# 11th International Conference on Developmental Coordination Disorder (DCD11): Developmental coordination disorder and other neurodevelopmental disorders: a focus on comorbidity

**DOI:** 10.15256/joc.2015.5.52

**Published:** 2015-06-23

**Toulouse, France, July 2–4, 2015**


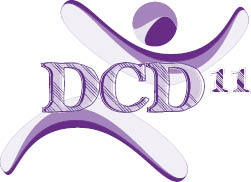


**DCD11 Organizing Committee**

Jean-Michel Albaret, University of Toulouse; Christophe Barré, University of Toulouse; Maëlle Biotteau, University of Toulouse; Mélody Blais, University of Toulouse; Yves Chaix, Neuropediatric Unit, University Medical Centre of Toulouse; Marianne Jover, Aix-Marseille University; Caroline Karsenty, University Medical Centre of Toulouse; Jennifer Lareng-Armitage, University of Toulouse; Agnès Laurent, University of Toulouse; Anaïs Mazella, University of Toulouse; Régis Soppelsa, University of Toulouse; Jessica Tallet, University of Toulouse

**DCD11 Scientific Committee**

Jean-Michel Albaret, University of Toulouse, France; Christine Assaiante, Aix-Marseille University, France; Anna L. Barnett, Oxford Brookes University, UK; Patrick Berquin, University of Amiens, France; Rainer Blank, University of Heidelberg, Germany; John Cairney, McMaster University, Hamilton, Canada; Chantal Camden, University of Sherbrooke, Canada; Yves Chaix, University of Toulouse, France; Jane E. Clark, University of Maryland, USA; Reint H. Geuze, University of Groningen, The Netherlands; Dido Green, Oxford Brookes University, UK; Sheila E. Henderson, Institute of Education University of London, UK; Marianne Jover, Aix-Marseille University, France; Marie-Laure Kaiser, University Hospital of Lausanne, Switzerland; Mitsuru Kashiwagi, Hirakata City Hospital, Japan; Amanda Kirby, University of Wales, UK; Livia C Magalhães, Federal University of Minas Gerais, Brazil; Anita E. Pienaar, North-West University, South Africa; Helen Polatajko, University of Toronto, Canada; Bouwien Smits-Engelsman, KU Leuven, Belgium; David Sugden, University of Leeds, UK; Jessica Tallet, University of Toulouse, France; Jean-Luc Velay, Aix-Marseille University, France; Hilde van Waelvelde, Artevelde University College & Ghent University, Belgium; Naomi Weintraub, Hebrew University of Jerusalem, Israel; Peter Wilson, Australian Catholic University, Australia; Jill G. Zwicker, University of British Columbia, Canada

Comorbidity refers to the presence of two or more disorders in the same person (especially DCD, dyslexia and attention deficit hyperactivity disorder in terms of developmental disorders). There has been growing interest in the presence of comorbidity in persons with neurodevelopmental disorders. Many recent studies suggest that up to half of all individuals diagnosed with a psychiatric or neurodevelopmental disorder have more than one condition. Comorbidity not only impacts patient outcomes but can also create a significant strain on both family and school life. It can also complicate diagnosis and healthcare organization. The 11^th^ congress on DCD aimed to address some of the important issues surrounding comorbidity in neurodevelopmental disorders. Three main topics were covered during oral and poster presentations: (1) assessment and diagnostic criteria, (2) underlying processes, causal factors, and prognostic markers, and (3) intervention and management of DCD and associated disorders.

## Keynote speakers

### Does improving motor skills or physical fitness improve cognitive skills?

**Adele Diamond**

*Division of Developmental Cognitive Neuroscience, Department of Psychiatry, The University of British Columbia (UBC), Vancouver, BC V6T 2A1 Canada. *adele.diamond@ubc.ca

Studies that look at the benefits of various forms of exercise for executive functions, that are not simply correlational, do not look at the effects of a single session only, and include a comparison group will be reviewed. There is a dearth of evidence that exercise without executive function (EF) components (e.g., aerobic walking or running or resistance training) improves executive functions. (EFs include ‘thinking outside the box’ and flexibly adjusting to unexpected change [cognitive flexibility], mentally relating ideas and facts or holding sequences in mind [working memory], and giving considered responses rather than impulsive ones, resisting temptations, resisting distractions, and staying focused [inhibitory control, including selective attention].) Results appear more promising for exercise with motor skill and cognitive components (e.g., soccer or traditional martial arts). Links between motor skills and cognitive skills will be briefly discussed

**Keywords:** Executive functions; Working memory; Aerobics; Resistance training; Martial arts; Sports; Prefrontal cortex; Cerebellum; Striatum.

### Neural basis of fine motor skill learning

**H. Forssberg**

Neuropediatric Unit, Karolinska Institutet, Astrid Lindgren Children’s Hospital, 17176, Stockholm, Sweden. hans.forssberg@ki.se

During the last decades there has been a growing interest in exploring the activity-dependent plasticity of the brain in order to develop new intervention programmes for children with motor disorders. Neuroimaging studies in humans have demonstrated both training associated alterations in brain volume (MRI), brain activity (fMRI) and changes in dopamine receptors (PET). Genetic studies have shown that some genotypes, including variation in dopamine genes and other plasticity genes, influence motor learning. Yet, there is a need to study animal models to understand the molecular mechanisms underlying learning induced plasticity.

Control of fine motor skills involves circuits in the frontal-striatal-cerebellar network and differs thus distinctly from the well-studied memory systems in the hippocampus and related structures. Studies in rodents have demonstrated that training in a forelimb skilled reaching task leads to rapid turnover and selective stabilization of “spines” on the dendrites of efferent pyramidal neurons in motor cortex. The dopamine system innervating the motor cortex seems to be necessary for learning fine motor skills shown by, e.g., i) chemical elimination of dopaminergic projections, ii) application of D1R and D2R antagonists in motor cortex, and recently iii) in animal models with genetic variations of the dopamine system.

The lecture will give a brief summary of previous studies on deficits in the neural control of the precision grip in children with DCD indicating a failure to form memory representations of objects used to scale the programmed grip force output. In the second part, results from studies on a skilled motor learning task in rodents will be presented, including genetic models, e.g., ADHD. Finally, recent findings on the molecular mechanisms involved in skilled motor learning in rodents will be presented. The results suggest that there are modifications of the intracellular dopamine signalling. The potential to utilize learning induced plasticity to improve motor functions in children with DCD will be discussed.

**References**:

Diaz Heijtz, R., & Forssberg, H. (2015). Translational studies exploring neuroplasticity associated with motor skill learning and the regulatory role of the dopamine system. *Developmental Medicine & Child Neurology, 57*, 10-14.

Qian, Y., Lei, G., Castellanos, F., Forssberg, H., & Heijtz, R. (2010). Deficits in fine motor skills in a genetic animal model of ADHD. *Behavioral and Brain Functions, 6*(1), 51.

Qian, Y., Chen, M., Forssberg, H., & Diaz Heijtz, R. (2013). Genetic variation in dopamine-related gene expression influences motor skill learning in mice. *Genes, Brain and Behavior, 12*(6), 604-614.

### Neuroimaging in Developmental Coordination Disorder: a review with a focus on comorbidity

M. Habib

Aix-Marseille University, UMR 7291 CNRS, Marseille, France. michel.habib@univ-amu.fr

Compared to other neurodevelopmental disorders, such as ADHD or dyslexia, the literature pertaining to the brain substrate of DCD is relatively scarce, since a complete review of relevant works in 2013 only identified seven separate relevant studies. This is probably due both to the complexity of the brain systems potentially involved, and to the difficulty identifying pure, non-comorbid cases of DCD. Since then, a handful of mainly brain MRI studies have been added allowing to draw a tentative panorama of the dysfunction and morphology of these individuals’ brain. In the present review, I will first consider brain regions potentially involved in motor coordination as derived from apraxic adults lesions and functional imaging studies of the intact brain (including during handwriting), then briefly review the main results of the few imaging studies of DCD cohorts. Among the latter, a special emphasis is put on the concept of comorbidity with other neurodevelopmental disorders suggesting that, not unlike the latters, brain changes in DCD might be mainly ascribed to a defect in connectivity between distant brain areas, including cerebellum, frontal and parietal regions. A comprehensive model of the neurodevelopmental disorders is proposed, featuring a general hypothesis of developmental disconnectivity of long-distance white matter tracts.

### Different profiles of children with developmental coordination disorder (DCD): challenges for research and intervention

Marie-Laure Kaiser

Health Department, University Hospital, Lausanne, Switzerland. Marie-Laure.Kaiser@chuv.ch

Both research and clinical intervention have to deal with a number of different profiles of children with DCD. In fact, these children may present either a general motor deficit, or a selective deficit touching fine motor or gross motor skills. They could also present deficiencies in proprioception, visual perception and/or tactile perception, which in turn impede motor skills. Moreover, children with DCD are more likely to have associated disorders such as dyslexia, attention deficit and/or hyperactivity, autism spectrum disorder or intellectual disability. These different children with one or more developmental disorders grow up in different environments, which also have an influence on their development. For example, if the teachers are well informed and trained to deal with these different profiles and know how to adapt their teaching, the children will have fewer secondary effects. If the family has received information and education concerning the disorders of their child, the child will grow up in a more favorable environment for his development. For the purpose of clinical practice, an exact profile of the child should be created. This evaluation provides a clear understanding of the different deficiencies and how they are interacted between them. The evaluation should also take into account the influence of these deficits on the daily life of the children. Only then, can a targeted intervention be provided including the evidence-based principles for the treatment of DCD and other disorders, if needed. For the purpose of research, one challenge is to identify the different profiles of children with DCD and to describe their specific features. Another challenge is to analyse the relationship between the different disorders, to identify when the motor coordination deficiencies result from another disorder. Lastly, in order to obtain valid results, it is essential to provide a good description of the profiles of the children included in research projects and to identify the influence of the environmental factors on the development of the children. Obviously, research and clinical practice need to interact with each other so as to offer a better understanding of the interactions between different deficits and the child’s environment. This is the best way to offer the most appropriate intervention.

### Considering a lifelong approach to DCD

A. Kirby

The Dyscovery Centre, Felthorpe House, Caerleon Campus, University of South Wales, NP18 3QR, UK. amanda.kirby@newport.ac.uk

DCD is a lifelong condition with a variable presentation depending on an individual’s ecology now and in their past. No adult presents with DCD alone. For example they are likely to have other developmental disorders to a greater or lesser degree, and are at greater risk of having anxiety and depression. Executive functioning difficulties commonly have an impact on their day-to-day lives. There is no typical presentation despite many adults displaying significant difficulties with motor functioning if assessed.

DCD needs to be considered as a multi-system complex and interwoven disorder. The secondary consequences of having DCD may result in reduced fitness and weight gain. This may have a longer-term impact on joints and cardiovascular functioning. Less participation in physical and social activities may also lead to greater social isolation, less social rehearsal, reduced confidence and further impact on development of close relationships and impact on wellbeing. Difficulties driving may make it harder to get out to participate as well. The reason individuals seek help or guidance may be for multiple reasons including for example potential loss of job, challenges with relationships, or difficulties in education.

The Dyscovery Centre at the University of South Wales, UK has been one of the few places seeing adults with DCD for more than fifteen years. This has presented a unique opportunity not only to follow children growing up with DCD, but also to see adults moving from education to employment, from parental supervision towards independence, and to witness the challenges that this presents. This paper will present a unique insight from both personal and professional perspectives, including a 29-year follow up single case study of an individual with DCD. The presentation will include what is currently known about DCD from emerging adulthood and the decades following including highlighting recent work by colleagues. It will discuss the potential direction for future research in an ageing population of individuals with DCD.

### Development of dyslexia: Procedural learning and delayed neural commitment

R.I. Nicolson

Department of Psychology, The University of Sheffield, Western Bank, Sheffield S10 2TP, UK. r.nicolson@sheffield.ac.uk

It is now evident that explanations of many developmental disorders need to include a network perspective. In earlier work we proposed that developmental dyslexia is well-characterised in terms of impaired learning within the procedural language networks. Here the analysis is extended to include the child’s developmental process of constructing these networks. The ‘delayed neural commitment’ framework proposes that, in addition to slower skill acquisition, dyslexic children take longer to build (and to rebuild) the neural networks that underpin the acquisition of reading. The framework provides an important link backwards in time to the development of executive function networks, and the earlier development of networks for language and speech. It is consistent with many theories of dyslexia, while providing fruitful suggestions for further research at the genetic, brain, cognitive and behavioural levels of explanation. It may also be a fruitful framework for the analysis of other developmental disorders, including ADHD, Specific Language Impairment and Developmental Coordination Disorder.

### Paradigm shift from a deficit to a task oriented point of view: Consequences for assessment and skill acquisition strategies in children with DCD

B. Smits-Engelsman

Department of Biomedical Kinesiology, KU Leuven, Leuven, Belgium; Department of Health and Rehabilitation Sciences, University of Cape Town, South Africa. bouwiensmits@hotmail.com

In the revised edition of the DSM-5, it is acknowledged that the acquisition and execution of coordinated motor skills is dependent on the availability of opportunities to learn and practice these skills (Criterion A). Consequently, a diagnosis of DCD will have to wait until a child has been exposed to a context where learning of a specific task is optimized. For how long and what has to been done in the mean time to meet this criterion, is not known. If we believe that training of children with DCD should be task oriented, which we do, the question rises how do you measure if the task performance is below the level that is expected, and how do we evaluate that the task has improved to a required level other than by using questionnaires or mere goal attainment. Although DCD is a disorder characterized by delayed acquisition of motor actions remarkably little research focused on how children with DCD learn new motor skills and what would be the best teaching strategy. Consequences of the paradigm shift for diagnosis, testing, and training and outcome measurement will be discussed.

## Oral Presentations

**Day 1: July 2, 2015**

### Psychometrics in every day clinical practice: do we test what we claim to test in children with DCD?

W.F.M. Aertssen^**1**^, G.D. Ferguson^**2**^ & B.C.M. Smits-Engelsman^**2,3**^

^1^Avansplus, University for Professionals, Breda, the Netherland. wendyverhoef@live.nl; ^2^Department of Health and Rehabilitation Sciences, Division of Physiotherapy, Faculty of Health Sciences, University of Cape Town, South Africa; ^3^Department of Biomedical Kinesiology, KU Leuven, Leuven, Belgium.

**Aim:** Children with DCD have, in addition to motor coordination problems, significantly lower levels of fitness than typically developing children including muscle strength and cardiovascular endurance. One explanation for the poor performance in physical fitness tests is that children with DCD participate less in sports and have low self-efficacy. As an alternative, it has also been suggested that poor coordination itself could explain the lower performance on these tests. In this study we will investigate the influence of coordination demands in the different sprint-, agility- and muscle endurance items in TD and DCD children.

**Method:** 35 DCD and 35 TD children aged 4-10 years from South Africa participated in this study. The Movement Assessment of children 2nd edition (MABC-2), the muscle endurance items and standing long jump of the Functional Strength Measurement (FSM), the running speed and agility items of the Bruininks Oseretsky Test-2nd edition (BOT2), the 10x5 sprint and the 10x5 sprint slalom were administrated. The Mann Witney U test was performed to calculate differences between the TD and DCD-group. We investigated if the differences increase if agility demands of a task increase. Factor analysis was used to evaluate the factors within the total dataset and if the loading was comparable between the DCD and TC children.

**Results:** The DCD children had significant lower performance on all tested items except for lifting a box. Difference between groups increased in tasks with higher agility demands. Factor analyses showed that there were 3 factors present in the dataset explaining 67% of the variance. The loading of the factors was different in DCD and TD children.

**Discussion:** Children with DCD have lower levels of fitness. This study also demonstrated that the construct of the different items in DCD children is different from TD children. Dimensions measured differ per population tested. The coordination/agility problems in DCD children influenced performance on muscle strength and anaerobic endurance tests. This information has to take in consideration when interpreting test results.

**Keywords:** Measurement; Functional strength; Anaerobic endurance; Construct.

### Reliability and validity of the Finnish version of Motor Observation Questionnaire for Teachers (MOQ-T-FI)

P. Asunta^**1**^, H. Viholainen^**2**^, T. Ahonen^**3**^, P. Rintala^**1**^, M. Cantell^**4**^ & M. Schoemaker^**5**^

^1^Department of Sport Sciences, University of Jyväskylä, Finland. piritta.asunta@gmail.com; ^2^Department of Education, Special Education Unit, University of Jyväskylä, Finland; ^3^Development Psychology, Department of Psychology, University of Jyväskylä, Finland; ^4^Department of Special Educational Needs, University of Groningen, The Netherlands; ^5^Centre for Human Movement Sciences, University Medical Centre Groningen, The Netherlands.

**Aim:** Despite the fact that DCD is so common, teachers lack tools to identify children who have motor learning problems. The Motor Observation Questionnaire for Teachers (MOQ-T) is an observational questionnaire for teachers developed in the Netherlands. The culture and language adaptation into Finnish was carried out and psychometric properties of this Finnish version (MOQ-T-FI) were studied. The aim of this study was to test the reliability and validity of the Finnish version of Motor Observation Questionnaire for Teachers (MOQ-T-FI).

**Method:** Item consistency, test-retest and inter-rater reliability, predictive and concurrent validity of the MOQ-T-FI were examined. Teachers were asked to complete the MOQ-T-FI. In this pilot study, teachers’ ratings were compared to students’ (n=193, aged 6−12 years) performance on the Movement Assessment Battery for Children-2. The factor structure (principal component analysis with varimax rotation) was examined in a standardization children’s sample (n=850, aged 6−9 years). Reliability was studied in two additional samples (n=25 and n=66).

**Results:** The psychometric properties of the Finnish version were compatible with those of the original MOQ-T. The internal consistency of MOQ-T-FI was excellent (Cronbach’s alpha α=.97). The same factor structure was found, the 18 items could be divided into two components: general motor functioning and handwriting. The components explained 77,2 % of the variation. Concurrent validity with MABC-2 test was moderate, but statistically significant (*r*=.33 p<.001). The original MOQ-T had better concurrent validity with MABC test (*r*=.57). Sensitivity 89,9 % and specificity 62,9 % were slightly better in MOQ-T-FI than in the original MOQ-T (Schoemaker et al. 2008). MOQ-T-FI test-retest was good [*r*=.616, p<.001; ICC (95 % CI) =.905(.71−.96)] and inter-rater reliability was moderate [*r*=.68, p<.001; ICC (95 % CI) = .67 (.51−.78)].

**Discussion:** The MOQ-T-FI can be considered as a valid and fast screening tool for Finnish children at risk of DCD. It has good psychometric properties, but further research is required to evaluate more specifically and with larger data sets the reliability of the Finnish version.

**References**:

Schoemaker, M. M., Flapper, B. C. T., Reinders-Messelink, H., & Kloet, A. (2008). Validity of the motor observation questionnaire for teachers as a screening instrument for children at risk for developmental coordination disorder.* Human Movement Science, 27*(2), 190-199.

**Keywords:** DCD; MOQ-T; Questionnaire; Validity; Reliability.

### Motor perseveration and variability as markers of learning difficulties of bimanual coordination in teenagers with Developmental Coordination Disorder

M. Blais^**1**^, Y. Chaix^**2,3**^, C. Baly^**1**^, M. Biotteau^**2**^, J.-M. Albaret^**1**^& J. Tallet^**1**^

^1^PRISSMH EA 4561, Université Toulouse III, UPS, 118 route de Narbonne 31062 Toulouse Cedex 9, France. melody.blais@univ-tlse3.fr; ^2^Inserm, Imagerie Cérébrale et Handicaps Neurologiques UMR 825, CHU Purpan, Toulouse, France & Université de Toulouse III, UPS, Toulouse, France; ^3^Unité de neurologie pédiatrie, Hôpital des Enfants, CHU Purpan, Toulouse, France.

**Aim:** There is a large consensus that developmental coordination disorder (DCD) presents an alteration of motor control, particularly in bimanual coordination. However, studies relative to motor learning are scarce and not consensual. We propose to test the learning of a new bimanual coordination in teenagers with DCD.

**Method:** Ten typically developed (TD, 13.49 +/- 1.76 yo) and 10 DCD (13.47 +/- 1.39 yo) teenagers were assessed with neuropsychological tests: WISC-IV, CPT-II and M-ABC. During Pre-test, teenagers were required to produce three coordination modes by tapping with their thumbs on a Xbox joystick in synchrony with visual stimulations specifying two pre-existing coordination: Inphase and Antiphase, and a New non pre-existing coordination. Then, teenagers were required to practice 25 trials of the New coordination only, they had to improve its accuracy and stability thanks to a visual feedback of the performance delivered after each trial. Finally, a Post-test assessed the three coordination modes. A Group × Test × Coordination ANOVA were carried out on (1) the Absolute Error (AE) of the produced coordination reflecting accuracy, (2) its Standard Deviation (SD) reflecting variability and (3) the number of additional taps of the both right and left thumbs (N), reflecting motor perseveration. Correlation between behavioural and neuropsychological results were tested (p<.05).

**Results:** Significant Coordination and Test × Coordination were found on AE: AE of the New coordination decreased whereas the AE of Antiphase increased between the Pre- and Post-test. Group and Coordination effects were found on SD which SD was higher for DCD compared to TD, and for Antiphase and New compared to Inphase coordination. Group, Coordination and Group × Coordination were significant on N: N was higher for Antiphase and New compared to Inphase coordination only for the DCD Group. Correlation tests revealed that teenagers with the highest M-ABC scores presented the highest N for the Antiphase and New coordination.

**Discussion:** Results reveal that the accuracy of the new coordination improved similarly in both groups. However, teenagers with DCD present more perseveration and variability without improvement with practice. This suggests that they preserve the ability to improve accuracy but alter the stabilization of a new bimanual coordination. Moreover, motor perseveration also represents a good marker of motor learning difficulties and are even more numerous that the general motor ability (M-ABC) is low.

**Keywords:** Synchronization; finger tapping; procedural learning; accuracy.

### Identification of Developmental Coordination Disorder by BOT-2 and M-ABC-2 (German versions)

R. Blank^**1,2**^, S. Vinçon^**3,4**^, J. Link^**2**^ & E. Jenetzky^**1,5,6**^

^1^Department of Pediatrics, Child Centre Maulbronn, 75433 Maulbronn, Germany. blank@kize.de; ^2^University of Heidelberg, Germany; ^3^Department of Occupational Therapy, Child Centre Maulbronn, Germany; ^4^Department of Sport and Health Sciences, Oxford Brookes University, United Kingdom; ^5^Divison of Clinical Epidemiology and Aging Research, German Cancer Research Center, Heidelberg, Germany; ^6^Department of Child and Adolescent Psychiatry, University Medicine Mainz, Germany.

**Aim:** In addition to the existing German version of the M-ABC 2 (M-ABC 2 G), the BOT-2 an alternative well known motor assessment battery was recently translated into German and completely re-standardized for German speaking countries (BOT-2 G). Both batteries have been recommended as diagnostic assessments in a recent German-Swiss clinical guideline for DCD. While the M-ABC 2 includes eight items in three subtests, the BOT-2 is more extensive with 53 items in eight subtests. The aim of this first pilot study is the comparison of both assessments (German speaking versions) for assisting diagnostic formulation of DCD.

**Method:** Up to know 56 children (age 4-12 years) were assessed with both assessments. Half of these children had been diagnosed with DCD by clinical judgement of an experienced pediatrician. The clinical judgement has been based on extensive history taking, report of the kindergarten or school and clinical observation. According to the EACD recommendations for DCD, cases with a performance below 16th percentile (one standard deviation) in the assessment were considered as possible DCD. Sensitivity and specificity and optimum sensitivity/specificity were calculated.

**Results:** Using the 16 th percentile, the M-ABC 2 G has a higher specificity (0.91) in comparison to the German BOT-2 G (0.64). In contrast, the sensitivity is with 0.89 higher in the BOT-2 G than the M-ABC-2 G (0.59).

The optimal sensitivity-specificity equation (Youden-Index) of the BOT-2 G is the 6^th^ percentile. At this cut-off it achieves combined and gender-specific norm values a high sensitivity (0.75; 0.79) and specificity (0.89; 0.86). In comparison the M-ABC 2 G demonstrated best sensitivity-specificity equation at the 25^th^ percentile with a sensitivity of 0.70 and specificity of 0.84 in combined norms. Using combined norms both test achieve a diagnostic odds ratio of 15, whereas usage of gender specific BOT 2 G norms achieved a diagnostic benefit with enhanced odds ratio of 22.

**Discussion:** The BOT-2 G gender-specific values seem to support the clinical judgement of the responsible pediatrician better than the M-ABC-2 G. The BOT-2 G version seems to be more sensitive than the M-ABC-2 G version; suggesting that a lower percentile may be more adequate when using the BOT-2 G. A possible explanation could be that the BOT-2 is based on more comprehensive motor dimensions and that the German standardization sample has achieved a higher average performance than the original American standardization sample. Further transcultural and diagnostic research is recommended.

**References**:

Blank, R., Smits-Engelsman, B., Polatajko, H., & Wilson, P. (2012). European Academy for Childhood Disability (EACD): recommendations on the definition, diagnosis and intervention of developmental coordination disorder (long version). *Developmental Medicine and Child Neurology, 54*, 54-93.

Blank, R., Jenetzky, E., & Vinçon, S. (Ed.) (2014). *Bruininks-Oseretzky Test der motorischen Fähigkeiten – Zweite Ausgabe (BOT-2) nach R.H. Bruininks und B.D. Bruininks*. Frankfurt: Pearson Assessment.

Bruininks, R. H., & Bruininks, B. D. (2005). *Bruininks-Oseretsky Test of Motor Proficiency – Second Edition (BOT-2)*. NCS Pearson, Inc.

Henderson, S. E., Sugden, D. A., & Barnett, A. L. (2007). *Movement Assessment Battery for Children-2. Second Edition (Movement ABC-2). Examiner’s manual.* London: Harcourt Assessment.

Petermann, F. (Ed.) (2008). *Movement Assessment Battery for Children, Second Edition. Deutschsprachige Adaptation nach S.E. Henderson, D.A. Sugden, A.L. Barnett*. Frankfurt: Pearson Assessment.

**Keywords:** BOT-2; M-ABC-2; Sensitivity; Specificity.

### Measurement properties of the Parents and Teachers Questionnaires of the ACOORDEM

A.A. Cardoso, L.C. Magalhães, M.B. Rezende & A.M.V.N. Van Petten

Department of Occupational Therapy, Federal University of Minas Gerais, Av. Antônio Carlos 6627, Belo Horizonte, MG, 31270-901, Brazil. anaameliato@yahoo.com.br

**Aim:** The Assessment of Motor Skills and Dexterity (Avaliação da Coordenação e Destreza Motora – ACOORDEM) is a Brazilian motor test created to provide professionals working with children with DCD, a valid and reliable instrument to detect the disorder (Criteria A and B) and to help defining intervention goals. ACOORDEM includes Parents’ and Teachers’Questionnaires that were designed to obtain information about the activities and social participation of children. The aim of this study was to examine the measurement properties and the quality of items of the ACOORDEM´s Questionnaires.

**Method:** The study included 426 children 4-8 years old from the metropolitan region of Belo Horizonte, whose parents answered the Developmental Coordination Disorder Questionnaire - DCDQ-Brazil and ACOORDEM’s Parents Questionnaire (54 items), and teachers completed the Teachers Questionnaire (30 items). The children were evaluated by the Movement Assessment Battery for Children (MABC-2) and the ones who presented scores below the 5th percentile both in the DCDQ-Brazil and the MABC-2 were considered as meeting criteria for DCD. The quality of the questionnaires was analyzed using Rasch analysis. Pearson correlation was used to investigate the concurrent validity between the DCDQ-Brazil and the subscales of ACOORDEM´sParents (i.e., Mobility, ADL, Student Role and Behavior) and Teachers (i.e., Motor and Behavior) Questionnaires.

**Results:** Rasch analysis showed the scales presented acceptable reliability (0.90 to 0.98) and internal consistency (Chronbach α = 0.86 to 0.96). Evidence of unidimensionality of the items in each scale, differentiation of children by age and diagnosis support the construct validity of the questionnaires. Considering the motor subscales of both Parents’ and Teachers’ Questionnaires, the easiest item was to go up and down stairs while maintaining good posture and writing items were the most difficult. Inthe Parents Questionnaire ADL subscale the most difficult item was using cutlery. Children with DCD score lower on behavioral subscales of both Questionnaires. The correlation between the ACOORDEM´s Parents and Teachers Questionnaires was significant, but low (0.338, p <0.001); between the Parents Questionnaire and the DCDQ-Brazil was even lower (0.196, p <0.001); and between the Teachers Questionnaire and the DCDQ-Brazil wasn’t significant.

**Discussion:** Both Questionnaires have acceptable measurement properties. Even though some items were easy for the sample, they differentiate around four ability levels. A few items that felt on the same ability level could be dropped, shortening both Questionnaires. Children with DCD tended to scored lower on all scales, including on the behavioral subscales, which gives support to the co-occurrence of behavioral and motor problems. Poor correlation with the DCDQ-Brazil suggests the instruments assess different aspects of motor behavior, with ACOORDEM also including behavioral factor.

**References**:

Henderson, S. E., Sugden, D. A., & Barnet, A. (2007). *Movement assessment battery for children 2nd ed (MABC-2)*. San Antonio, TX: The Psychological Corporation.

Magalhães, L. C. Rezende, M. B., Cardoso, A. A. (2014). *Avaliação da Coordenação e Destreza Motora – ACOORDEM - Versão 5*. Belo Horizonte: Departamento de Terapia Ocupacional, UFMG. Manuscrito não publicado.

Prado, M. S., Magalhães, L. C., & Wilson, B. N. (2009). Cross cultural adaptation of the Developmental Coordination Disorder Questionnaire for Brazilian Children. *Revista Brasileira de Fisioterapia,13*, 236-243.

**Keywords:** Questionnaires; Parents; Teachers; Diagnosis; ACOORDEM.

### The Developmental Coordination Disorder affects processing of action-related verbs

S. Del Signore^**1**^, F. Capozzi^**1**^, R. Averna^**1,2**^, R. Penge^**1**^& G. Mirabella^**3,4**^

^1^Department of Pediatrics and Neuropsichiatry of Children and Adolescents, University of Rome “Sapienza”, via dei Sabelli 108, 00185 Rome, Italy; ^2^IRCCS Children Hospital Bambino Gesù, Rome, Italy; ^3^Department of Physiology and Pharmacology, University of Rome La Sapienza, Piazzale Aldo Moro 5, 00185 Roma, Italy. giovanni.mirabella@uniroma1.it; ^4^IRCCS Neuromed, Pozzilli (IS), Italy.

**Aim:** A growing body of research has shown that the processing of action language affects the planning and execution of motor acts. These findings can be interpreted as evidence of the involvement of the cortical motor system in action-language understanding. However, at present, there is not yet an indubitable evidence in support of this claim. To tackle this issue, we compared the processing of action-verb information in children with Developmental Coordination Disorder (DCD) versus those with a non pathological development. The aim was to demonstrate that motor impairments typical of DCD, which are related to pathological functioning of the motor system, also affect language processing.

**Method:** We recruited 17 clinical subjects diagnosed with DCD, according diagnostic criteria of DSM-5 (APA, 2013) and a group of 20 healthy children, without motor impairment or behavioral disorders. All children were born in Italy, and attended the last three years of the primary school. To investigate the relationship between action execution and the processing of motor language material we administered two versions of a go/no-go task. In one version, participants were required to perform arm reaching movements toward a target when verbs expressing either hand or foot actions were shown, and to refrain from moving when abstract verbs were presented (“semantic task”, Mirabella *et al*., 2012). In the second version, (“color discrimination task”), participants had to move when the color of the printed verb was green and to refrain when it was red.

**Results:** In healthy children, similarly to what happen in young adults (see Mirabella *et al*., 2012), we found that, in the semantic task, reaction times (RT) increased when the verb involved the same effector used to give the response (i.e. the arm). However, when the color of the printed verb and not its meaning was the cue for movement execution the differences between RTs between verb categories disappeared, indicating that the phenomenon occurs only when the semantic content of a verb has to be retrieved. Differently DCD patients did not show any difference between verb categories irrespective of the task. This finding suggests that in DCD individuals the processing of verb semantic is compromised.

**Discussion:** Our results sustain the theory of embodied language, which hypothesizes that comprehending verbal descriptions of actions relies on an internal simulation of the sensory–motor experience of the action, and provide a new view of the interplay between action language, motor acts and the motor system.

**References**:

Mirabella G, S. Iaconelli, S. Spadacenta, P. Federico, V. Gallese (2012). Processing of Hand-Related Verbs Specifically Affects the Planning and Execution of Arm Reaching Movements. PlosOne 7(4):e35403

**Keywords:** Developmental Coordination Disorder; Embodied theory of language; Action language; Arm reaching movement; Semantics.

### Mental health and diagnosis in adults with Developmental Coordination Disorder (DCD): A qualitative study

L. Dockery^**1**^ & E. Hill^**1**^

^1^Department of Psychology, Goldsmiths, University of London, New Cross, London, SE14 6NW, United Kingdom. l.dockery@gold.ac.uk

**Aim:** Neurodevelopmental disorders are commonly associated with poor mental health. However, few studies have considered mental health in adults with developmental coordination disorder (DCD). Previous research suggests adults with DCD are less motivated to participate in physical activities and so are at higher risk of poorer physical and mental health. Adults with DCD may also experience difficulties obtaining a DCD diagnosis due to a lack of standardised diagnostic criteria. This qualitative study aims to investigate the effects of DCD in adulthood by considering feelings about diagnosis, social and emotional adjustment and participation in sports.

**Method:** Semi-structured qualitative interviews were carried out with 15 adults aged 18 years or over. Ten adults had a DCD diagnosis and five adults did not have a formal DCD diagnosis but strongly suspected they had DCD (and exceeded the ‘DCD at risk’ threshold on The Adult/Dyspraxia Checklist [ADC; Kirby, Edwards, Sugden, & Rosenblum, 2010]).

**Results:** Thematic analysis identified four main themes with several subthemes: childhood (ball/team sports, humour); adulthood (ball/team sports, humour, choice, coping mechanisms); diagnosis (positive retrospect, negative retrospect) and mental health (time, energy). The themes identified tentatively suggest differences between adults who had received a DCD diagnosis and those who had not. Adults with DCD who received a diagnosis at an earlier age tended to report more positive retrospective experiences from childhood. The interviews suggest these adults move through a process of understanding; naming; externalising and absolution which either happens later or not at all for adults who are left undiagnosed.

**Discussion:** Specific diagnostic criteria for adults with DCD are yet to be fully defined. Many adults with motor coordination impairments may not receive an official diagnosis and may experience difficulties in daily living skills, such as cooking, self-care and budgeting. It is essential that guidelines for the diagnosis of DCD in adulthood are fully developed in order to provide adequate support, treatment and intervention for these individuals.

**References**:

Kirby, A., Edwards, L., Sugden, D., & Rosenblum, S. (2010). The development and standardization of the Adult Developmental Co-ordination Disorders/Dyspraxia Checklist (ADC).* Research in Developmental Disabilities, 31*, 131-139.

**Keywords:** DCD; Adult; Mental health; Diagnosis; Qualitative.

### Cross-cultural adaptation of the “Here’s How I Write” a self-assessment of the handwriting for the Brazilian children

J.M.A. Flores^**1**^, A.V.M. Van Petten^**1**^, A.A. Cardoso^**1**^, C.R. Lages^**2**^, S.A. Costa^**2**^& L.C. Magalhães^**1**^

^1^Departamento de Terapia Ocupacional, Universidade Federal de Minas Gerais, Av. Antônio Carlos 6627, Belo Horizonte, MG, 31270-901, Brazil. jufloresto@gmail.com; ^2^Curso de Terapia Ocupacional, Universidade Federal de Minas Gerais, Belo Horizonte, MG, Brazil.

**Aim:** Handwriting is an essential component of school work and an important skill for academic success. Children with difficulties in handwriting may avoid writing which, in the long run, can compromise school performance. The “Here’s How I Write” (HHIW) is a new assessment tool which incorporates aspects of the client centered approach as it involves the perspective of both the teacher and the child. The instrument helps the child and the teacher to identify handwriting problems and collaborate to define goal and solutions to improve performance. The objective of this study was to conduct a transcultural adaption of the HHIW to the Brazilian Portuguese.

**Method:** The study was conducted in two steps: (1) process of transcultural adaptation of the HHIW, as proposed by Beaton *et al,* (2) examine validity and reliability for the Brazilian children. The sample consisted of 60 children from public and private schools, ages 8 to 10 years old, both genders, divided into two groups, identified by the teachers: G1 with handwriting difficulty and G2 no handwriting difficulty. The children from both groups were paired by gender, school level, age and social class. The children were assessed individually and the teachers answered the questionnaire, resulting in scores for the child (C) and teacher (T). The total and partial scores of the two groups was compared (Mann Whitney) and reliability indexes were calculates, test retest (ICC) and internal consistency (Cronbach Alfa).

**Results:** Children with poor handwriting (C = 68,87 ± 10,67; T = 65,57 ± 13,85) presented scores significantly lower than children with a good handwriting (C = 86,57 ± 10,67; T = 82,83 ± 12,94) (p<0,05). Test-retest reliability (consistency) was excellent for the total score for both the child (ICC=0,94) and the teacher (ICC=0,93) questionnaires. The internal consistency was also excellent (C =0,915 and T =0,953). The congruence (total agreement) between the total score for C and T was moderate (ICC>0,73,p=0,000) and on individual items, some did not show good congruence between C and T.

**Discussion:** There was significant difference between the groups regarding the children with and without handwriting difficulties. The psychometric qualities of HHIW were maintained with the adaptation, showing that this assessment tool can be used with Brazilian children to help in the identification of problems in calligraphy and in the definition of intervention goals focused on improving handwriting.

**References**:

Beaton, D. E., Bombardier, C., Guillemin, F. & Ferraz, M.B*.* (2000). Guidelines for the process of cross-cultural adaptation of self-report measures. *Spine*, 25(24), 3186-3191.

Goldstand, S., Gevir, D., Cermak, S., & Bissell, J. (2013). *Here’s How I Write: A child’s self-assessment and goal setting tool*. Framingham, MA: Therapro.

**Keywords:** Assessment; Handwriting; Validity; Occupational therapy.

### Numerical cognition of children with Developmental Coordination Disorder

A. Gomez^**1,2**^, M. Piazza^**1**^, A. Jobert^**1**^, G. Dehaene-Lambertz^**1**^, S. Dehaene^**1**^ & C. Huron^**1**^

^1^Cognitive Neuroimaging Unit, INSERM, U992, CEA/SAC/DSV/DRM/NeuroSpin, 91100, France. alice.gomez@isc.cnrs.fr; ^2^Center for Cognitive Neuroscience (CNC), UMR 5229, Université Claude Bernard Lyon 1 (UCBL), Ecole Supérieure du Professorat et de l’Enseignement (ESPE), Lyon, France.

**Aim:** Children with developmental coordination disorder (DCD) struggle with mathematics at school. However, little attention has been paid to numerical cognition of these children. We propose to use a psychophysical approach that taps elementary numerical processes to better understand difficulties with mathematics learning reported in DCD.

**Method:** To assess this hypothesis, twenty 7-to-10 years-old children with DCD were compared to twenty age –matched typically developing children in different numerical tasks. We used tasks assessing symbolic and non-symbolic number magnitude^1^, number line tasks^2^, arithmetic problems, counting and subitizing tasks^3,4^.

**Results:** Results showed that number acuity is severely impaired in DCD. Children with DCD were impaired in comparing dots and symbolic numbers. They were slower to solve simple arithmetic problems. Their counting skills and subitizing ability were reduced. Moreover, their numerical estimation in the number line task was less accurate and slower. Despite these numerical deficits, children with DCD were able to understand the mathematical concept of the linearity of numbers.

**Discussion:** These findings suggest that DCD impairs number processing but that abstract mathematical concepts can be grasped. A systematic assessment of numerical cognition in children with DCD could provide a more comprehensive picture of their deficits and help to propose specific remediation.

**References**:

1. Piazza, M., Facoetti, A., Trussardi, A. N., Berteletti, I., Conte, S., Lucangeli, D., … Zorzi, M. (2010). Developmental trajectory of number acuity reveals a severe impairment in developmental dyscalculia. *Cognition*, *116*(1), 33–41. doi:10.1016/j.cognition.2010.03.012

2. Siegler, R. S., & Booth, J. L. (2004). Development of numerical estimation in young children. *Child Development*, *75*(2), 428–44. doi:10.1111/j.1467-8624.2004.00684.x

3. Revkin, S. K., Piazza, M., Izard, V., Cohen, L., & Dehaene, S. (2008). Does subitizing reflect numerical estimation? *Psychological Science*, *19*(6), 607–14. doi:10.1111/j.1467-9280.2008.02130.x

4. Silverman, I. W., & Rose, A. P. (1980). Subitizing and counting skills in 3-year-olds. *Developmental Psychology*, *16*(5), 539–540. doi:10.1037//0012-1649.16.5.539

**Keywords:** Mathematics; Numerical cognition; Subitizing; Counting; Approximate number system.

### The relationship between motor and executive functioning in 3- to 5-year-old children

S. Houwen & M.H. Cantell

Department of Special Needs Education and Youth Care, Faculty of Behavioral and Social Sciences, University of Groningen, Groningen, The Netherlands. s.houwen@rug.nl

**Aim:** There is an emerging body of evidence showing that motor and executive functioning are intertwined. However, due to the limited research on the relationship between motor and executive functioning in pre-school children, we aimed to further investigate this potential relationship in 3- to 5-year-old children. Specifically, we addressed whether motor performance was associated with specific executive functions (EFs) in young children on a spectrum of motor functioning, thus including children with typical development and children at risk for motor difficulties.

**Method:** EFs were reported by parents of 112 children (mean age 4 years 7 months, SD 10 months; 61 male) by means of the Behavior Rating Inventory of Executive Function—Preschool version (BRIEF-P)1,2. In addition, the children performed the Movement Assessment Battery for Children 2nd Edition (MABC-2)3,4. We calculated partial correlation coefficients (controlling for child age and gender) for potential relationships between the scores of the MABC-2 and the BRIEF-P for the total group of children and separately for two subgroups of children: being at risk for motor difficulties (falling at or below the 16th percentile on the MABC-2; n = 47; mean age 4 years 9 months, SD 9 months; 25 male) and typically developing (above the 16th percentile on the MABC-2; n = 65; mean age 4 years 6 months, SD 10 months; 36 male).

**Results:** In the total group, four out of the five EF scales (Inhibit, Shift, Working Memory, and Plan/Organize) showed weak to moderate correlations (ranging from -.23 to -.48) with Manual Dexterity. Working Memory and Plan/Organize were weakly correlated (-.25 and -.23, respectively) to Ball Skills. Shift, Working Memory, and Plan/Organize had weak to moderate correlations (-.21 to -.35) with Balance. In the typically developing subgroup, no significant correlations were found. In the at risk for motor difficulties subgroup, moderate to strong correlations (ranging from -.36 to -.54) were found between three of the EF scales (Inhibit, Working Memory and Plan/Organize) and Manual Dexterity and Balance. No significant correlations were found between the EF scales and Ball Skills.

**Discussion:** This is one of the first studies to look at the interrelation among motor and executive functioning in such a young age group. It shows that the relationship between motor and executive functioning is complex in young children. Specific relationships were found between motor performance and EFs, but it is depended on whether motor difficulties are present. With regard to the method, it is important to note that the EF findings are based only on parent report of child behavior on one measure, which may differ from performance-based assessment of EFs.

**References**:

1. Gioia, G. A., Espy, K. A., & Isquith, P. K. (2003). *Behavior Rating Inventory of Executive Function Preschool version (BRIEF-P): Professional manual.* Lutz, FL: Psychological Assessment Resources.

2. Van der Heijden, B., Suurland, J., de Sonneville L. M. J. & Swaab, H. (2012). *BRIEF-P Executieve functies gedragsvragenlijst voor jonge kinderen*. Amsterdam, Nederland: Hogrefe Uitgevers.

3. Henderson, S. E., Sugden, D. A. & Barnett, A. L. (2007). *Movement Assessment Battery for Children-2. Second Edition (Movement ABC-2). Examiner’s manual.* London: Harcourt Assessment.

4. Smits-Engelsman, B. C., Henderson, S. E., Sugden, D. A., Barnett, A. L. (2010). *Movement Assessment Battery for Children-2, Second Edition, Manual, Nederlandse bewerking.* Amsterdam: Pearson Assessment and Information B.V*.*

**Keywords:** Motor skills; Executive functioning; Early childhood.

### Handwriting and graphomotor learning in children with developmental coordination disorder and/or dyslexia

A. Huau^**1**^, S. Bellocchi^**2**^, S. Ducrot^**3**^, J.-L. Velay^**4**^, J. Mancini^**5**^, F. ­Brun-Hénin^**5**^ & M. Jover^**1,2**^

^1^PsyCLE Center EA 3273, Aix-Marseille University, Aix en Provence, 13100, France. andrea.huau@etu.univ-amu.fr; ^2^Epsylon Rsearch Unit EA 4556, University of Montpellier & Paul Valéry University, Montpellier, France; ^3^Laboratory Parole and Language, UMR 7309, CNRS & Aix-Marseille University, Aix en Provence, France; ^4^Cognitive Neuroscience Laboratory, UMR 7291, CNRS & Aix-Marseille University, Marseille, France; ^5^CERTA (Centre of reference for learning disabilities), Salvator Hospital, APHM, Marseille, France.

**Aim:** Handwriting and graphomotor activities have been showed to be altered in children with developmental disorders as developmental dyslexia (DD)^1,2^ and developmental coordination disorder (DCD)^3,4,5^. However, children with those two disorders have seldom been compared in a same graphomotor task. Consequently, as far as we know, there isn’t any study demonstrating whether they difficulties rely on different or similar processes. This study aimed to analyse the respective influence of reading level and motor skills level on graphomotor product and process.

**Method:** Dyslexic and DCD children were contacted at the Marseille’s diagnostic center of reference for learning disabilities (CERTA) and children without dyslexia and DCD in a school. A total of 130 children aged between 8 and 12 years participated to the study (33 DD children, 18 DCD children, 14 DD and DCD children, 65 typical developing children. The M-ABC^6^, the Alouette^7^ and the BHK^8^ tests were administrated to the children. Two experimental tasks were proposed on a graphic tablet. A graphomotor learning task consisted in copy a new caracter with the presence of model, then without the model. For the second task, children were asked to write a known word “*lapin*” (rabbit) at self-paced and as fast as possible, it is the handwriting task. The amount of stops, the mean velocity, the number of strokes and the path length were measured for each trial.

**Results:** Linear models (GLM and GEE) were used to test the influence of the reading and the motor skill levels on the cinematic data and according to the learning trial. Data analysis is currently in progress.

**Discussion:** This study aimed for a better understanding of handwriting difficulties in children with DCD and/or DD in estimating the respective influence of reading and motor skills. Researches considering the degree of difficulties severity and the comorbidity in neurodevelopmental disorders should be pursued.

**References**:

1. Cheng-Lai, A., Li-Tsang, C. W. P., Chan, A. H. L., & Lo, A. G. W. (2013). Writing to dictation and handwriting performance among Chinese children with dyslexia: Relationships with orthographic knowledge and perceptual-motor skills. *Research in Developmental Disabilities, 34*, 3372-3383.

2. Lam, S. S. T., Au, R. K. C., Leung, H. W. H., & Li-Tsang, C. W. P. (2011). Chinese handwriting performance of primary school children with dyslexia. *Research in Developmental Disabilities, 32*, 1745-1756.

3. Rosenblum, S., & Livneh-Zirinski, M. (2008). Handwriting process and product characteristics of children diagnosed with developmental coordination disorder. Human Movement Science, 27, 200-214.

4. Rosenblum, S., Margieh, J. A., & Engel-Yeger, B. (2013). Handwriting features of children with developmental coordination disorder - Results of triangular evaluation. *Research in Developmental Disabilities, 34*, 4134-4141.

5. Smits-Engelsman, B. C. M., Niemeijer, A. S., & van Galen, G. P. (2001). Fine motor deficiencies in children diagnosed as DCD based on poor grapho-motor ability. *Human Movement Science, 20*, 161-182.

6. Henderson, S. E., & Sugden, D. A. (1992). *Movement Assessment Battery for Children*. London: The Psychological Corporation.

7. Lefavrais, P. (2005). *Alouette-R Test d’analyse de la lecture et de la dyslexie*. Paris: ECPA.

8. Hamstra-Bletz, L., & Blöte, A. W. (1993). A longitudinal study on dysgraphic handwriting in primary school. *Journal of Learning Disabilities, 26*(10), 689-699.

**Keywords:** Developmental dyslexia; Developmental coordination disorder; Learning; Handwriting; Graphic tablet; Comorbidity.

### Understanding executive functioning performance in Developmental Coordination Disorder: the importance of verbal and nonverbal task demands

H.C. Leonard^**1**^, M. Bernardi^**2**^, E.L. Hill^**1**^ & L.A Henry^**2**^

^1^Department of Psychology, Goldsmiths, University of London, London, SE14 6NW, UK. h.leonard@gold.ac.uk; ^2^Language and Communication Science, City University, London, UK.

**Aim:** The study was designed to assess a range of high-level abilities, known as executive functions, in children with DCD and motor difficulties (MD). These executive functions include tasks that affect activities of daily living and academic achievement, such as planning and multi-tasking, which are often reported to be difficult for individuals with DCD, as well as for those with a range of other neurodevelopmental disorders such as Attention Deficit-Hyperactivity Disorder. Specifically, this study aimed to assess a wide range of executive functions and to compare tasks with verbal and nonverbal task demands. It was predicted that children with motor impairments (both DCD and MD) would have greater difficulties with those tasks that involved a motor demand, but that performance in verbal tasks would be relatively less affected.

**Method:** Children aged 7-11 with a clinical diagnosis of DCD (*N*=23), and with motor difficulties identified through screening (MD: *N*=30; MABC-2 Total score <16th percentile), were compared to typically-developing children (TD: *N*=38) on verbal and nonverbal tests of executive-loaded working memory, inhibition, switching, planning and fluency. Nonverbal measures included tasks with a motor and/or visuospatial demand. In order to isolate the effect of motor impairments on executive functioning in these groups, children with co-occurring disorders were not included in the sample, and subclinical symptoms of inattention and hyperactivity and reading difficulties were taken into account in the analyses.

**Results:** Once differences in executive functioning relating to age, IQ, reading ability and subclinical inattention/hyperactivity symptoms were taken into account, significant group differences were revealed for all of the *nonverbal* measures except for switching. Children with MD and DCD had significantly lower scores or produced more errors than TD children in nonverbal tasks of executive-loaded working memory, inhibition, planning and fluency, but were similarly accurate to the TD children on all of the verbal tasks.

**Discussion:** Difficulties in nonverbal executive functions could have a significant impact on classroom functioning, particularly when tasks involve processing visuospatial information or a motor skill, such as handwriting. Future studies should include children with co-occurring neurodevelopmental disorders in order to assess the impact of other symptoms on executive functions in DCD across a clinical sample.

**Keywords:** Executive function; Verbal; Nonverbal; Motor difficulties; DCD.

### Is the Movement Assessment Battery for Children – 2nd edition (MABC-2) valid for brazilian children 4 to 8 years-old? A comparison between Brazil and UK

L.C. Magalhães^**1**^, C.Linhares^**2**^, A.L. Barnett^**3**^, B.L.C. Moraes^**4**^, C.G. Silva^**5**^, O.S. Agostini^**6**^ & A.A. Cardoso^**1**^

^1^Departamento de Terapia Ocupacional, Universidade Federal de Minas Gerais, Av. Antônio Carlos 6627, Belo Horizonte, MG, 31270-901, Brazil. liviacmag@gmail.com; ^2^Curso de Terapia Ocupacional, Instituto Federal do Rio de Janeiro, Brazil; ^3^Department of Psychology, Social Work & Public Health, Oxford Brookes University, United Kingdom; ^4^Curso de Terapia Ocupacional, Universidade Federal de Minas Gerais, Av. Antônio Carlos 6627, Belo Horizonte, MG, 31270-901, Brazil; ^5^Departamento de Terapia Ocupacional, Universidade Federal de Pelotas, Brazil; ^6^Departamento de Terapia Ocupacional, Universidade Federal do Rio de Janeiro, Brazil.

**Aim:** The MABC-2 is being used in Brazil and although there are two validity studies, none of them compared actual data. The aim of this study was to compare the performance of children ages 4 to 8 years old from Brazil (BR) and the United Kingdom (UK) on individual items of the motor test component of the Movement Assessment Battery for Children 2nd edition (MABC-2).

**Method:** 887 typically developing children (396 BR; 491 UK) stratified by age and gender were assessed with the MABC-2. Data on UK children were obtained from the MABC-2 normative data set. Brazilian children were recruited and assessed in public and private schools, according to standardized procedures. Means and standard deviation were calculated for the raw score of each individual item for the ages 4, 5, 6,7, and 8 years old. ANOVA was used for group comparison (p≤.05)

**Results:** There was no significant age difference between groups on each age group. Concerning motor performance, there were scattered differences in means for different items and ages. On manual dexterity, UK children were better on Placing Pegs with preferred hand at age 7 (p=.001) and they committed less mistakes on the Drawing Trail at ages 5 (p=.001), 6 (p=.001), 7 (p=.016), and 8 (p=.007); there were no differences on the Threading tasks, and 7-y-old BR children were better on Placing Pegs with non-preferred hand (p=.002). On Ball Skills, there were no significant differences on Catching, but UK children were better on Throwing at ages 7 (p=.001) and 8 (p=.001). There were no differences on the Jumping and Hopping tasks, but 6-y-old BR were better on preferred (p = .001) and non-preferred leg (p=.001) One-Leg Balance, and UK were better on Heel-to-Toe walking at ages 6 (p=.004) and 7 (p=.023). Further analysis will investigate gender and socioeconomic issues.

**Discussion:** There were differences in several motor items at different ages and, overall, UK children tended to outperform Brazilians when differences were found. These differences could be related to cultural as well as to socioeconomic factors as half of the Brazilian sample was recruited in public schools, which have higher representation of children from socially disadvantaged backgrounds. Although Brazilians are recognized for their ball skills, they did not perform better on the MABC-2, maybe because most children are not familiar with the tennis ball, used in the test. The impact of these differences on the MABC-2 cut off scores will be discussed.

**Reference**:

Henderson, S. E., Sugden, D. A., & Barnett, A. L. (2007). *Movement assessment battery for children 2nd ed (MABC-2)*. London: Harcourt Assessment.

**Keywords:** MABC-2; Children; Motor coordination; Cross cultural study.

### The relationship between motor coordination and internalising symptoms in a normative sample of 4 to 6 year old children

V. Mancini^**1**^, D. Rigoli^**1**^, L. Roberts^**1**^, J. Cairney^**2**^, B. Heritage^**1**^ & J. Piek^**1**^

^1^School of Psychology and Speech Pathology, Curtin University, Perth, 6102, Australia. vincent.mancini@postgrad.curtin.edu.au; ^2^Department of Family Medicine, McMaster University, Ontario, Canada.

**Aim:** Previous research has indicated a range of comorbid and secondary problems are associated with poor motor coordination. These findings have been established in both clinical and non-clinical samples. Recent empirical developments have moved beyond investigating only the comorbid neurodevelopmental comorbid and secondary problems, and have started to investigate the additional implications that poor motor coordination has for an individual’s mental health. A relationship between motor coordination and internalizing symptoms (e.g. anxious and depressive symptoms) is well supported by research. However, an understanding of the causal nature of this relationship remains conceptually underdeveloped. The recently proposed Elaborated Environmental Stress Hypothesis (EESH)^1^ provides a promising theoretical framework that allows the causal nature of this relationship to be empirically evaluated. The framework posits that the presence of poor motor coordination leads to the development of internalizing symptoms through several mediating constructs that are embedded within the individual and their surrounding environment (e.g. interpersonal conflict, peer social support, perceived self-competence). This study aims to investigate a subset of key pathways specified in the EESH, using a normative sample of West Australian children between 4 and 6 years of age. It is hypothesized that perceived self-competence and social acceptance will mediate the relationship between motor coordination and internalizing problems.

**Method:** Participants were 445 West Australian children between 4 and 6 years of age. Cognitive, physiological, psychosocial and demographic measures were collected as part of the pre-test phase of a universal school-based motor coordination intervention program. Motor coordination was measured using the Movement Assessment Battery for Children-II (MABC-2), and the short form of the Bruininks-Oseretsky Test of Motor Proficiency, Second Edition (BOT2-SF). Perceived self-competence and social acceptance was measured using the four subscales of the Pictorial Scale of Perceived Competence and Social Acceptance (PSPCSA). Internalizing problems were measured using the teacher-rated Strength and Difficulties Questionnaire (SDQ-T).

**Results:** Model testing using Path Analysis in LISREL is ongoing. Preliminary analysis indicated that, after controlling for age and verbal IQ, the MABC-2, the two PSPCSA subscales relating to social acceptance (maternal acceptance and peer acceptance) were not correlated with the outcome variable (internalizing problems) and subsequently excluded from the analysis. Similarly, preliminary analysis yielded results that indicated a small effect size, with the total model approximately accounting for a small, but significant 6% of variance in teacher rated levels of internalizing problems.

**Discussion:** The present study provides support for a link between motor coordination and internalizing problems. While a complete analysis is currently ongoing, there is preliminary evidence to suggest that in the current study, the relationship between motor coordination and internalizing problems was not mediated by perceived self-competence and social acceptance. It is important to recognize the limitations of this study may have impacted results; low levels of internal reliability in some measures may have attenuated the relationship between variables. The results of this study provide only partial support for the EESH, though there are several additional pathways and variables specified in the model that remain theoretically proposed, but empirically untested. This will be addressed in a series of future studies.

**References**:

Cairney, J., Rigoli, D., & Piek, J. (2013). Developmental coordination disorder and internalizing problems in children: The environmental stress hypothesis elaborated. *Developmental Review,* *33*, 224-238. doi: 10.1016/j.dr.2013.07.002

**Keywords:** Motor coordination; Motor development; Internalizing problems; Anxiety; Depression; Environmental stress hypothesis.

### Comparing the BOT-2 and MAND for identifying young adults with motor impairment

F. McIntyre^**1**^, A. Thornton^**2**^, B. Hands^**3**^, M. Licari^**2**^, J. Piek^**4**^ & D. Rigoli^**4**^

^1^School of Health Sciences, The University of Notre Dame, Fremantle, 6959, Australia. fleur.mcintyre@nd.edu.au; ^2^School of Sport Science, Exercise and Health, The University of Western Australia, Crawley, Australia; ^3^The Institute for Health Research, The University of Notre Dame, Fremantle, Australia; ^4^School of Psychology and Speech Pathology, Curtin University, Bentley, Australia.

**Aim:** Few studies have sought to understand motor coordination and health outcomes in young adults. To firstly identify those with poor motor proficiency, motor competence tests suitable for the adult population need to be identified. The purpose of this study was to compare discrimination accuracy of the McCarron Assessment of Neuromuscular Development (MAND) and the Bruininks Oseretsky Test of Motor Proficiency-2 (BOT-2) in a sample of young adults.

**Method:** *Participants:* Ninety-one adults (33 males, 58 females) aged between 18 to 30 years (M = 21.4 years) participated in the study. ­*Measures:* Participants completed the BOT-2 Short Form^1^ and the MAND^2^. The BOT-2 Short form comprises 14 items measuring four motor composite areas; fine manual control, manual coordination, body coordination and strength and agility. A scale score is calculated from raw scores. The scale scores for each subtest are summed and converted to a standard score. The Motor Area standard scores are summed and converted to a Total Motor Composite standard score (M=50; SD=10). Those with a score of 40 or less are considered to have motor impairment. The MAND involves five fine motor and five gross motor tasks. Raw scores are then scaled to the participant’s age and summed to indicate a Total Scaled score. This score is then standardised to a normal distribution (M=100; SD=15). Those with a standard score of 85 or less are considered to be at risk of a motor disability. *Data analysis:* Spearman rank order correlation examined the relationship between the BOT-2 Total standard scores and MAND Total standard scores. Level of agreement compared the MAND and BOT-2 identifying adults as having motor impairment. The discrimination accuracy of both measures was assessed by calculating the sensitivity, specificity, and negative and positive predictive values.

**Results:** A small, statistically significant correlation coefficient of .370 (*p* = .01) was found between the MAND and BOT-2 scores. Of the 91 adults in the sample, 6 were classified as having motor impairment on the MAND, and 12 on the BOT-2, with 4 cases identified by both measures. The overall decision agreement between the two tests was 84.6%. The BOT-2’s sensitivity was higher (66.7%) compared to the MAND (33%), and had a higher negative predictive value (97.3% compared to 90%), indicating it was a more accurate discriminator of adults with motor impairment.

**Discussion:** Results indicate high overall decision agreement between the two measures, however the BOT-2 is a more accurate discriminator of motor impairment with higher test sensitivity and negative predictive values. Pending results will examine the different samples identified by the tests, in particular those individuals with motor impairment, and their characteristics and scores on test items.

**References**:

1. McCarron. L. (1997). *MAND: McCarron Assessment of Neuromuscular Development*. Dallas, TX: Common Market Press.

2. Bruininks, R.H., & Bruininks, B.D. (2005). *The BruininksOseretsky Test of Motor Proficiency, 2nd ed*: *Manual.* Circle Pines, MN: AGS Publishing.

**Keywords:** Motor competence; BOT-2; MAND; Adults; Assessment.

### Suitability of the “Little DCDQ” for the identification of DCD in a selected group of 3 - 5 year old South African children

A.E. Pienaar, D. Coetzee & A. Venter

Physical activity, Sport and Recreation (PhASRec), Faculty of Health Sciences, Potchefstroom Campus, North-West University, Republic of South Africa. anita.pienaar@nwu.ac.za

**Aim:** Early identification of children who might run the risk of motor delays is important to ensure intervention as soon as possible to prevent such delays manifesting as larger problems^1,2^ (Majnemer, 1998; Malina, 2004). A primary goal of early motor intervention is to increase the skill levels in all the motor development domains to prevent or minimalize further delays and secondary consequences. In order to identify DCD as soon as possible we need validated screening instruments that can be used for the early identification of motor coordination delays. The aim of this study was to establish the suitability of the Little Developmental Coordination Disorder Questionnaire for the identification of DCD in a selected group of 3-5 year old South African children (N=53).

**Method:** Both reliability and validity of the Little DCDQ were assessed. Test items of the Little DCDQ, completed by the parents, were compared against the standardized Movement Assessment Battery for Children-2, in a group of 53 children aged 3 – 5 years.

**Results:** Correlations of r=0.3 were established between 2 of the test items and good internal consistency (Chronbach’s Alpha, r=>0.8) was established. The Little DCDQ showed poor sensitivity (57.14%) but reasonable specificity (81.25%).

**Discussion:** These results indicate that the Little DCDQ has potential as a screening instrument to detect possible DCD but a few adjustments need to be considered. The questionnaire should therefore be refined in order to identify children with possible DCD more accurately in South African children. Modifications are recommended to the types of questions and their level of difficulty to make it more understandable, especially for illiterate parents.

**References**:

1. Majnemer, A. (1998). Benefits of early intervention for children with developmental disabilities. *Seminars in Pediatric Neurology, 5*(1), 62–69.

2. Malina, R. M. (2004). Motor development during infancy and early childhood: Overview and suggested directions for research. *International Journal of Sport and Health Science, 2*, 50–66.

**Keywords:** Little DCDQ; MABC-2; DCD; Preschool children; Screening; Parents.

### Do measures of visual perception and visual motor integration help explain handwriting difficulties in children with DCD?

M. Prunty^**1**^, A.L. Barnett^**2**^, K. Wilmut^**2**^ & M. Plumb^**3**^

^1^Brunel University London, Division of Occupational Therapy, Uxbridge, Middlesex, UB8 3PH, U.K. mellissa.prunty@brunel.ac.uk; ^2^Oxford Brookes University, Dept. of Psychology, Social Work & Public Health, Oxford, U.K.; ^3^School of Health Sciences & Psychology, Federation University Australia, Ballarat, Australia.

**Aim:** Occupational therapists (OT) receive many referrals for handwriting difficulties, particularly in children with Developmental Coordination Disorder (DCD). In a recent study we found that children with DCD demonstrated a lack of automaticity in handwriting as measured by pauses during writing. Deficits in visual perception have been proposed in the literature as underlying mechanisms of handwriting difficulties in children with DCD. Indeed tests of visual perception and visual motor integration are some of the most widely used assessment tools in paediatric OT practice worldwide. However, despite their popularity, there is a lack of empirical evidence surrounding their ability to detect or explain handwriting difficulties in children with DCD. This leads to confusion surrounding best practice in approaches to assessment and intervention in this group. The aim of this study was to examine whether a relationship exists between measures of visual perception and visual motor integration with measures of the handwriting product and process in children with DCD.

**Method:** Twenty-eight 8-14 year-old children who met the DSM-5 criteria for DCD participated in the study, with 28 typically developing (TD) age and gender matched controls. The children completed the Beery-Buktenica Developmental Test of Visual Motor Integration (VMI) and the Test of Visual Perceptual Skills (TVPS). Group comparisons were made and bivariate correlations conducted between the visual perceptual measures and a range of handwriting process and product measures (pausing, words per minute and legibility). The sensitivity of the VMI in detecting handwriting difficulties was also examined.

**Results:** The DCD group performed significantly below the TD group for scores on the VMI (t(43) = -3.85, p <.001) and the TVPS (t(43) = -4.06, p <.001). However, there were no significant correlations between the VMI and TVPS measures with any of the handwriting measures in the DCD group. Analysed together, the only significant relationship was between the VMI and a measure of legibility (R^2^=.31, F(1,43)=19.18, p<.001). However, 14 children with handwriting difficulties were not detected by the VMI.

**Discussion:** The findings of this study suggest that clinicians should execute caution in using visual perception measures to inform them about handwriting skill in children with DCD. Direct measures of performance within handwriting tasks may be more useful in identifying those with difficulties and planning appropriate interventions.

**Keywords:** Handwriting; Writing pauses; Visual perception; Visual motor integration.

### Hot cognition in children with motor coordination problems: Insights using a go/no-go paradigm

S. Rahimi-Golkhandan^**1**^, B. Steenbergen^**1,2**^, J.P. Piek^**3**^, K. Caeyenberghs^**1**^ & P.H. Wilson^**1**^

^1^Australian Catholic University, Melbourne, Australia. shahin.rahimi@acu.edu.au; ^2^Behavioural Science Institute, Radboud University Nijmegen, Netherlands; ^3^Curtin University, Bentley, Australia

**Background:** Recent research shows that children with Developmental Coordination Disorder (DCD) have associated deficits in executive function (EF) that extend to both cold and hot EF. More specifically, our recent work shows an impaired ability to use inhibitory control in contexts that are motivationally salient.

**Aim:** The aim of the study presented here was to extend the study of EF by enlisting a modified version of a go/no-go paradigm, one particularly sensitive to developmental change, and using facial stimuli that are easily discriminable by children.

**Method:** A 2-step procedure was used to assess DCD. An 85-centile cut-off on the McCarron Assessment of Neuromuscular Development was used to confirm suspected DCD. Thirty-six children (12 with DCD) aged 7-12 years participated in this study. Children completed two blocks of an emotional go/no-go task in which neutral facial expressions were paired with either happy or sad faces. Each expression was used as both a go and no-go target in different runs of the task.

**Results:** Results showed no group differences in the ability to approach sad faces. However, the DCD group made significantly more commission errors to happy no-go faces. Analysis of reaction time, omission errors, and *d*’, which measures sensitivity to each facial expression, showed that this difference was not attributable to either emotion recognition difficulties in DCD or speed-accuracy trade-off.

**Discussion:** Taken together, the particular pattern of performance in DCD confirms earlier reports of (hot) EF deficits. Specifically, a problem of inhibitory control appears to underlie the atypical pattern of performance seen in DCD on both cold and hot EF tasks. This constellation of deficits may explain other issues in self-regulation that have been observed in DCD, particularly in the context of skill learning and social interaction. The implications of this hypothesis for a broader theoretical account of DCD are discussed, as are implications for intervention. We present a conceptual model that describes the possible mediating role of EF deficits in mapping causal links between poor motor coordination and its behavioural consequences. Strategies for cognitive training as a means of intervention are described and evaluated.

**Keywords:** Hot executive function; Delay of gratification; Emotion-regulation; Inhibitory control; Cognitive control.

### The relationship between motor coordination and mental health in young adults: A test of the Environmental Stress Hypothesis

D. Rigoli^**1**^, R.T Kane^**1**^, F. McIntyre^**2**^, A. Thornton^**3**^, B. Hands^**4**^, M. Licari^**3**^ & J. Piek^**1**^

^1^School of Psychology and Speech Pathology, Curtin University, Bentley, 6102, Australia. d.rigoli@curtin.edu.au; ^2^School of Health Sciences, The University of Notre Dame, Fremantle, Australia; ^3^School of Sport Science, Exercise and Health, The University of Western Australia, Crawley, Australia; ^4^The Institute for Health Research, The University of Notre Dame, Fremantle, Australia.

**Aim:** Growing evidence has highlighted the importance of movement ability for psychosocial outcomes including self-perceived competence and social support, self-worth, and emotional areas such as anxiety and depression. The Environmental Stress Hypothesis-elaborated (Cairney, Rigoli, & Piek, 2013) is a proposed theoretical framework for understanding these relationships. Recent studies have tested this model using child and adolescent samples. The extent to which the relationships exist, persist or change during early adulthood is currently unclear. **Aim:** The current study aimed to investigate the Environmental Stress Hypothesis in a sample of 82 young adults aged 18 to 30.

**Method:** The McCarron Assessment of Neuromuscular Development (McCarron, 1997) was used to assess motor skills, the Depression Anxiety Stress Scale (Lovibond & Lovibond, 1995) provided a measure of mental health outcomes, and the Physical Self Perceptions Profile (Fox & Corbin, 1989) was used to investigate the possible mediating role of perceived physical self-worth. Potential confounding variables such as age, gender and BMI were also considered in the analysis.

**Results:** Structural Equation Modelling provided support for an indirect relationship, that is, perceived physical self-worth mediated the relationship between motor skills and mental health. Therefore, the results provide support for part of the model pathways as described in the Environmental Stress Hypothesis.

**Discussion:** This study suggests an important relationship between motor skills and mental health in a young adult population. Specifically, the results support previous literature regarding the role of physical self-worth for mental well-being and therefore suggest that an intervention that targets motor ability may also indirectly influence mental health through its positive effects on an individual’s physical self-worth.

**References**:

Cairney, J., Rigoli, D., & Piek, J. (2013). Developmental coordination disorder and internalizing problems in children: The environmental stress hypothesis elaborated. *Developmental Review*, *33*(3), 224-238.

Fox, K. R., & Corbin, C. B. (1989). The physical self-perception profile: Development and preliminary validation. *Journal of Sport and Exercise Psychology, 11*, 408-430.

Lovibond, P. F., & Lovibond, S. H. (1995). The structure of negative emotional states: Comparison of the Depression Anxiety Stress Scales (DASS) with the Beck Depression and Anxiety Inventories. *Behaviour Research and Therapy*, *33*(3), 335-343.

McCarron, L. T. (1997). *McCarron Assessment of Neuromuscular Development* (3rd ed.). Dallas, TX: McCarron-Dial Systems Inc.

**Keywords:** Young adults; Motor coordination; Mental health; Physical self-worth.

### Play characteristics of children with Developmental Coordination Disorders (DCD)

S. Rosenblum^**1**^, P. Waissman^**2,3**^ & G.W. Diamond^**2**^

^1^The Laboratory of Complex Human Activity and Participation (CHAP), Department of Occupational Therapy, University of Haifa, Israel. rosens@research.haifa.ac.il; ^2^Klalit Medical Services HMO, Dan Region. Israel; ^3^University clinics, Ariel University. Israel.

**Aim:** There is evidence that motor coordination deficits that characterize children with DCD affect the quality of their participation in various areas of their functioning, including play. Nevertheless, there is a paucity of studies examining the patterns and behavior during play in children with DCD. The aim of the study was to assess the characteristics of play in children with Developmental Coordination Disorders (DCD) in comparison with a control group of children with typical development.

**Method:** 60 Israeli children, age 4-6 years were examined, 30 of whom were diagnosed by pediatrician as having DCD and another 30 who were age, gender and socioeconomic level-matched controls. Parents completed a demographic questionnaire, the Children Activity Scale for Parents (CHAS-P), the Children Leisure Assessment Scale for preschoolers (CLASS-Pre) and My Child›s Play Questionnaire (MCP).

**Results:** Daily performance characteristics of children with DCD as reflected by the CHAS-P were significantly different compared to controls. The CLASS-Pre results indicated less sociability and participation in a reduced variety of play activities among children with DCD in comparison to controls. Furthermore, children with DCD had significantly inferior performance in MCP factors such as interpersonal interaction and executive function during play, as well as for play choices and preferences in comparison to controls.

**Discussion**: Results indicate that use of both the CLASS-Pre and MCP questionnaires enables the detection of unique play characteristics of children with DCD, who differ significantly from typically developing children. As play constitutes a central aspect of health and well-being, implications for both clinical and academic fields will be discussed.

**References**:

Baerg, S., Cairney, J., Hay, J., Rempel, L., Mahlberg, N., & Faught, B. E. (2011). Evaluating physical activity using accelerometry in children at risk of developmental coordination disorder in the presence of attention deficit hyperactivity disorder. *Research in Developmental Disabilities*, *32*(4), 1343-1350.

Lifter, K., Foster-Sanda, S., Arzamarski, C., Briesch, J., & McClure, E. (2011). Overview of play: Its uses and importance in early intervention/early childhood special education. *Infants & Young Children, 24*(3), 225-245.

Missiuna, C., Moll, S., King, S., King, G., & Law, M. (2007). A trajectory of troubles: parents› impressions of the impact of developmental coordination disorder. *Physical & Occupational Therapy in Pediatrics, 27*(1), 81-101.

**Keywords:** Play; Evaluation; Pre-school children.

### Unique arabic writing characteristics of arab children with Developmental Coordination Disorder

S. Rosenblum^**1**^, J. Aassy Margieh^**2**^ & B. Engel-Yeger^**1**^

^1^The Laboratory of Complex Human Activity and Participation (CHAP), Department of Occupational Therapy, University of Haifa, Israel. rosens@research.haifa.ac.il; ^2^Ministry of Education, Matia Shfaaram

**Aim:** School–aged children spend between 30-50% of their school day performing written assignments. Writing Arabic is demanding, as 22 of the 28 letters of the alphabet encompass four different morphological forms in the beginning, middle and end of the word or when the letter appears following a non-joinable letter. The study aimed to characterize the handwriting performance of children with DCD who write in Arabic, based on triangular evaluation.

**Method:** The participants included 58 children aged 11-12 years, 29 of which were diagnosed with DCD, based on the *DSM-IV* criteria, the M-ABC, and 29 matched, typically developed controls. The children were asked to copy a paragraph onto a sheet of paper affixed to a digitizer, which supplied objective measures of the handwriting process (ComPET)^1^. The Handwriting Proficiency Screening Questionnaire (HPSQ)^2^ was completed by their teachers during observation of their performance and followed by an evaluation of their final written product by the Hebrew Handwriting Evaluation (HHE)^3^.

**Results:** The results indicated that compared to controls, children with DCD required significantly more on-paper and in-air time per stroke while paragraph copying. In addition, global legibility, unrecognizable letters and spatial arrangement measures of their written product were significantly inferior. Significant group differences were also found between the HPSQ subscales scores. Furthermore, 82.8% of all participants were correctly classified into groups based on one discriminate function which included two handwriting performance measures.

**Discussion:** These results strongly support the application of triangular standardized evaluation to receive better insight into the handwriting deficit features of individual children with DCD who write in Arabic. The results’ theoretical and clinical meaning are discussed.

**References**:

1. Rosenblum, S., Parush, S., & Weiss, P.L. (2003). Computerized temporal handwriting characteristics of proficient and poor handwriters. *The American Journal of Occupational Therapy, 57*, 129-138.

2. Rosenblum, S. (2008). Development, reliability, and validity of the handwriting proficiency screening questionnaire (HPSQ*). The American Journal of Occupational Therapy, 62*(3), 298–307.

3. Erez, N., & Parush, S.(1999). *The Hebrew handwriting evaluation*. Israel: School of Occupational Therapy, Faculty of Medicine, Hebrew University of Jerusalem.

**Keywords:** Handwriting process; Product; Evaluation.

### Modelling the real-time coupling between inhibitory systems and online control in Developmental Coordination Disorder: A 2-year longitudinal study

S. Ruddock^**1**^, J. Piek^**3**^, D. Sugden^**4**^, C. Hyde^**2**^, S. Morris^**3**^, K. Caeyenberghs^**1**^, P. Wilson^1*^

^1^Australian Catholic University, Melbourne, Australia, peterh.wilson@acu.edu.au; ^2^Deakin University, Melbourne, Australia; ^3^Curtin University, Bentley, Australia; ^4^University of Leeds, United Kingdom

**Aim:** Children with DCD show performance deficits in online motor control, which are exacerbated when tasks impose demands on inhibitory function. There is some suggestion, however, that these effects may dissipate with age into older childhood. Longitudinal data are required to clarify this issue. Here we modelled the development of online control in DCD using a cohort-sequential design and predicted a more protracted period of development in DCD as these children learn to couple fronto-executive and motor control systems.

**Method:** 196 school children aged between 7 and 12 years were assessed at 6-month intervals over a 2 year period. Motor ability (MAND) and online control (Double-Step Reaching Task—DSRT) were evaluated at each of the five time points. For the DSRT, inhibitory control was examined using an anti-jump condition where children were instructed to resist reaching for a cued target but instead reach toward a contralateral location. A standard jump condition was also examined. Growth curve analysis (GCA) was used to model the trajectory of change on key metrics in DCD and non-DCD cohorts.

**Results:** GCA revealed a gradual improvement in movement time for both DCD and control cohorts over the two years. Importantly, for the DCD group, the additional processing time required to complete anti-jump movements (MT*diff*) conformed to a linear trend with developmental age. A linear trend was also evident on the difference score between the first and second corrective movements (observed on anti-jump trails; ToC*diff*); this measure also assesses how well inhibitory control can be enlisted during the course of online corrections. By comparison, for controls, performance trends conformed to a quadratic function, with evidence of re-organisation during middle childhood.

**Discussion:** Modelling of this extensive dataset showed that the real-time coupling between online control and inhibitory systems follows an atypical pattern in DCD. Whereas children without motor impairment showed evidence of re-organisation (during middle childhood) in both online control and the coupling of inhibitory and motor control systems, children with DCD did not, indicating a more protracted period of development. Under the hypothesis of *interactive specialisation,* more time is required to integrate the function of different control systems in children with DCD. These findings have significant implications for later cognitive and motor development, which are discussed.

**Keywords:** Online control; Predictive modeling; Inhibition; Cohort sequential design; Growth curve modeling.

^*^Presenting author

### Incidence and impact of falls in adults with significant motor coordination difficulties

S. Scott-Roberts & C. Purcell

The Dyscovery Centre, University of South Wales, Felthorpe House, Lodge Road, Newport NP18 3QT, UK. sally.scott-roberts@southwales.ac.uk

**Aim:** Balance is the ability to maintain a weight-bearing posture or to move through a sequence of postures, without falling. Balance represents a critical component of most movement activities, yet is often taken for granted. Previous evidence looking at children with Developmental Coordination Disorder (DCD) suggests that their walking patterns differ compared to their typically developing peers as a consequence of for example, gait instability^1^. Given the evidence demonstrating greater postural instability and increased trunk inclination during the gait cycle among children with DCD, it seems likely that the incidence of falls amongst adults with significant motor difficulties will be elevated. The aims of this project were **t**o explore the frequency and severity of trips and falls in adults diagnosed with DCD following a full multi-team assessment and explore the adaptive responses they have made to minimize the risk of trips and falls.

**Method:** To explore the incidents of trips and falls, a questionnaire which asked participants to indicate the frequency and severity of falls over the past six months was distributed to eighteen adults with a diagnosis of DCD. In addition, to explore the adaptations utilised to minimise falls six of these participants volunteered to take part in a 40 minute semi structured interview, conducted via the telephone.

**Results:** The results showed that 43% of respondents had fallen more than ten times in the previous six months. Respondents reported being concerned about falling for example, when walking on slippery surfaces, uneven pavements, down stairs, particularly when carrying objects, as well as when completing a range of daily chores. Almost all participants had made adaptations to minimise the risk of falling, for example: choice of footwear, division of chores within their household. Interestingly many had felt that their adaptations had become so automatic that they no longer saw them as adaptions.

**Discussion:** Falls are a serious public health concern and the results from the survey indicate that tripping and falling is common amongst this particular group of adults. The results from this study suggest that safe mobility should be assessed and fall prevention interventions should be considered by OTs working with children and adults with significant motor difficulties. However, the efficacy of traditional approaches to falls management would need to be demonstrated for this group. Additionally, the adults who participated have adapted many tasks and their environment to manage the risk and there are lessons to be learnt from these ‘experts’ that potentially could be shared with others who have motor difficulties.

**References**:

1. Deconinck, F. J. A., De Clercq, D., Savelsbergh, G. J. P., Van Coster, R., Oostra, A., Dewitte, G., & Lenoir, M. (2006). Differences in gait between children with and without developmental coordination disorder.* Motor Control, 10*(2), 125-142.

**Keywords:** Significant motor difficulties; Adults; Trips and falls; Daily functioning.

### Prevalence of attention and executive function in young adults with and without Developmental Coordination Disorder- A comparative study

M. Tal Saban^**1**^, A. Ornoy^**2,3**^ & S. Parush^**1**^

^1^School of Occupational Therapy, Hebrew University Hadassah Medical School, Jerusalem, Israel. miri.tal-saban@mail.huji.ac.il; ^2^Hebrew University Hadassah Medical School, Jerusalem, Israel; ^3^Israeli Ministry of Health, Jerusalem, Israel.

**Aim:** The current study examined the prevalence of attention problems and the executive function (EF) of young adults with Developmental Coordination Disorder (DCD) in comparison to young adults without DCD.

**Method:** The study used a randomized cohort (*N*=429) of young adults with DCD (n= 135), Borderline DCD (*n*= 149) and control (*n*= 145), from a previous study. This initial cohort was asked to participate in the current study three to four years later. Twenty-five individuals with DCD (mean age = 24.35 years [SD=0.88]; 52% males); 30 borderline DCD (mean age = 24.48 years [SD=0.98]; 43.3% males) and 41 typical individuals (mean age = 25.82 [SD=1.91]; 48.8% males) participated in this study. Participants completed the *BRIEF-A* questionnaire, assessing EF abilities and the WURS questionnaire, assessing attention abilities.

**Results:** The DCD and borderline DCD groups had significantly lower EF profiles in comparison with the control group. While a high percentage of attention problems were found in both DCD groups, the executive functioning profiles remained consistent even when using the attention component as a covariate.

**Discussion:** The current study adds to the knowledge of motor coordination deficits in the young adult population. Our results show that young adults with DCD have a distinctive executive functioning profile as well as a high percentage of attention deficits. These findings are important in light of the fact that most studies in this area were performed only on children and not in young adults and did not control for attention. The findings emphasize the important role EF plays in young adults with DCD and the need to address this issue in the DCD population.

**References**:

Michel, E., Roethlisberger, M., Neuenschwander, R., & Roberts, C. M. (2011). Development of cognitive skills in children with motor coordination impairments at 12 month follow-up.* Child Neuropsychology, 17,* 151-172.

Rigoli, D., Piek, J., Kane, R., & Oosterlaan, J. (2012). An examination of the relationship between motor coordination and executive functions in adolescents.* Developmental Medicine & Child Neurology, 54,* 1025- 1031.

Tal- Saban, M., Ornoy, A., & Parush, S. (2014). Executive function and attention in young adults with and without developmental coordination disorder: A comparative study. *Research in Developmental Disabilities, 35,* 2644-2650**.**

**Keywords:** Executive Function; Attention; Young adults; Comparative study.

### The development and evaluation of the Adolescents Motor Competence Questionnaire (AMCQ): A self-report questionnaire for 12-18 years olds

A. Timler^**1**^, F. McIntyre^**2**^, S. Crawford^**3**^, M. Cantell^**4**^ & B. Hands^**1**^

^1^Institute for Health Research, University of Notre Dame Australia, 6160, Australia. Amanda.Timler1@my.nd.edu.au; ^2^School of Health Sciences, University of Notre Dame Australia, 6160, Australia; ^3^Epidemiologist, Alberta Perinatal Health Program, Calgary, T2A 3N4 Canada; ^4^Department of Special Educational Needs, University of Groningen, The Netherlands.

**Aim:** At present there is no self-report tool to identify adolescents with DCD at the population level. In this paper the development and evaluation of a questionnaire for 12 – 18 year old Australian youth will be presented.

**Methods and Results:** The Adolescent Motor Competence Questionnaire (AMCQ) was developed over 4 phases using a sample of 37 adolescents (12-17yrs) with a range of motor competence. *Phase 1: Content Development.* This stage involved designing 29 test items focusing on aspects of motor competence. These were informed by the DSM-5 diagnostic criteria, an extensive literature search, and interviews with key informants with DCD. Eight leading experts in the field of DCD reviewed the terminology and content of each item which led to the development of a further 19 items expanding the questionnaire to 48 items. *Phase two: Content Validation.* Individual or small group interviews were completed with 10 adolescents with DCD discussing each item for its ‘youthfulness’ and ease of comprehension. The questionnaire was also sent to 10 experts to rate the relevance of each item (1-4). As a result 12 items were removed leaving a total of 36 items. Analysis of the internal consistency of the 36-item questionnaire identified 10 items to be removed resulting in a 26-item questionnaire (α = 0.922). *Phase three: Concurrent validation and reliability.* Adolescents with a range of motor skills completed the AMCQ and the McCarron Assessment of Neuromuscular Development (MAND) in order to determine the concurrent validity. The test-retest reliability of the AMCQ over 7 days was r = 0.96. *Phase four: Establishment of cut score.* The cut scores to identify probable DCD were established using the decision of agreement proportions between the AMCQ and the MAND.

**Discussion:** The final 26-item questionnaire has evidence of validity and reliability. Preliminary evidence indicates that this questionnaire is suitable for use with a general population and is capable of identifying adolescents with probable DCD. In the next stage, the questionnaire will be administered to a population-based sample of 300 adolescents. There is also potential to validate this tool with populations from other countries.

**References**:

American Psychiatric Association (2013). *Diagnostic and Statistical Manual of Mental Disorders* (5th ed.). Arlington, VA: American Psychiatric Publishing.

Fitzpatrick, A., & Watkinson, J. (2003). The lived experience of physical awkwardness: Adults Retropsective views. *Physical Activity Quarterly, 20*(3), 279-297.

Kirby, A., Edwards, L., Sugden, D., & Rosenblum, S. (2010). The development and standardization of the Adult Developmental Co-ordination Disorders/Dyspraxia Checklist (ADC). *Research in Developmental Disabilities, 31*, 131–139.

McCarron, L. (1997). McCarron Assessment of Neuromuscular Development (3rd ed.). Dallas, TX: McCarron-Dial Systems Inc.

Tal-Saban, M. T., Ornoy, A., Grotto, I., & Parush, S. (2012). Adolescents and adults coordination questionnaire: Development and psychometric properties. *American Journal of Occupational Therapy, 66*(4), 406-413.

**Keywords:** Self-Report Questionnaire; Adolescence; Motor competence; Identification; Assessment.

### Multidimensional profile of children with learning disabilities, with and without Developmental Coordination Disorder according to the ICF

R.Traub Bar-Ilan^**1**^, S. Parush^**1**^ & N. Katz^**2**^

^1^School of Occupational Therapy, Faculty of Medicine, Hadassah and The Hebrew University of Jerusalem, Jerusalem, Israel. rutrazt@gmail.com; ^2^Director Research Institute for the Health & Medical Professions, Ono Academic College, Or-Yehuda, Israel.

**Aim:** The DSM-5 states that children with learning disabilities (LD) suffer from impaired functioning in school, at work and in ADL. The APA also linked between LD and developmental disorders such as Developmental Coordination Disorder (DCD). Yet, LD literature lacks research regarding non-academic functioning. Therefore, there’s a call to consider the multi-dimensional implications of this disorder since they affect different aspects of the child’s functioning and participation. The aim of the study was to create a functional profile of children with LD with and without DCD according to ICF dimensions (body functions, activity and participation) and to determine whether motor dysfunctions have unique predictive value beyond other aspects of functioning, such as academic and attentional functions, within and outside of the school environment.

**Method:** 90 children, ages 7.3 - 12.4 years, diagnosed with LD were recruited to this study. Based on the Developmental Coordination Disorder Questionnaire participants were divided to two groups: children with DCD (40) and without DCD (50). Different ICF dimensions measures administered to the total sample included academic performance test; Dynamic Occupational Therapy Cognitive Assessment for Children; Test of Everyday Attention for Children; the Conner’s Abbreviated Parent-Teacher Questionnaire; Child Behavioral Checklist and the School Function Assessment. A comparative study design (LD with and without DCD) was conducted. MANOVA was computed for between-group analyses on multiple outcome variables. Regression was conducted to predict degree of participation.

**Results:** MANOVA revealed significant differences between LD with and without DCD, on most variables, primarily in participation (P<.02, η²=.06) pointing to lower participation scores amongst the LD with DCD group. Regression analysis revealed that motor performance has a unique contribution to predicting the participation of the child within the school environment beyond that accounted for by reading, writing, math and ADHD symptoms.

**Discussion:** Children with LD and DCD have substantial difficulties on all ICF dimensions. Moreover, motor dysfunction has a significant impact on all ICF dimensions, and was found to predict the overall participation**.** The finding of a high percentage of motor deficits among children with LD, and the broad influence of this deficit on children’s participation, supports the importance of identifying these children and enhancing their health and participation, both within and outside of school settings.

**Keywords:** Learning disabilities; Developmental Coordination Disorder; Participation; ICF.

### Elaboration of the Environmental Stress Hypothesis – Results from a population-based 6-year follow-up

M.O. Wagner^**1**^, D. Jekauc^**2**^, A. Worth^**3**^ & A. Woll^**4**^

^1^Department of Sport Science, University of Konstanz, Universitaetsstr. 10, Konstanz, 78457, Germany. matthias.wagner@uni-konstanz.de; ^2^Department of Sport Psychology, Humboldt University Berlin, Germany; ^3^Department of Sport Pedagogy, University of Education Karlsruhe, Germany; ^4^Department of Sport Science, Karlsruhe Institute of Technology, Germany.

**Aim:** Children with Developmental Coordination Disorder (DCD) face a variety of physical and mental health problems; their complex interrelation was recently modeled in a heuristic framework named the Environmental Stress Hypothesis (ESH, see Cairney *et al*., 2013). Following Missiuna and Campbell (2014), longitudinal studies are necessary to establish temporal causation within such frameworks. The aim of this paper is the longitudinal elaboration of the ESH-framework on the basis of the MoMo study (Wagner *et al*., 2013). We assume that, in comparison to their typically developed peers, children with potential DCD show a higher risk for persistent grossmotor coordination problems (H1), overweight and obesity (H2), physical inactivity (H3), peer-relationship (H4) as well as internalizing (H5) problems in adolescents.

**Method:** MoMo (a) started with a population-based representative sample of 4,529 German children and adolescents aged between 4 and 17 years at baseline (2003-2006), (b) continued with a first follow-up (2009-2012) and (c) includes standardized motor tasks, a physical activity questionnaire, as well as various health-measures. We focus on children between 6 and 10 years at baseline (*N*=1,681; *mean age*=8.27, *SD*=1.48; 50.4% boys) who were re-examined between the ages of 12 and 16 years (*N*=940; *Response Rate*: 55.9%; *mean age*=14.37, *SD*=1.46; 49.1% boys). Children in the longitudinal sample diagnosed as having potential DCD at baseline (*N*=115; 47.8% boys) were identified on the basis of three common grossmotor coordination tasks using the age- and gender-specific 15th percentile cut off and under careful consideration of the subordinated European Academy of Childhood Disability-criteria. Data were analyzed with binary logistic regressions including the stability of the respective dependent variable.

**Results:** In comparison to their typically developed peers, children with potential DCD show a higher risk for (i) persistent grossmotor coordination problems (*OR*=7.98, *p*<.01), (ii) overweight and obesity (*OR*=1.75, *p*<.05), (iii) physical inactivity (*OR*=7.41, *p*<.05), (iv) peer-relationship (*OR*=1.52, *p*<.05) as well as (v) internalizing (*OR*=1.57, *p*<.05) problems in adolescents.

**Discussion:** Our results provide evidence for the developmental impact of childhood DCD. Subsequent analysis will be focused on the mediating and moderating role of personal and social resources using the data of two subsequent survey waves (2014-2016; 2018-2020).

**References**:

Cairney, J., Rigoli, D., & Piek, J. (2013). Developmental coordination disorder and internalizing problems in children: The environmental stress hypothesis elaborated. *Developmental Review 33*, 224-238.

Missiuna, C., & Campbell, W. N. (2014). Psychological aspects of Developmental Coordination Disorder: Can we establish causality? *Current Developmental Disorders Reports, 1*(2), 125-131.

Wagner, M. O., Bös, K., Jekauc, D., Karger, C., Mewes, N., Oberger, J., Reimers, A. K., Schlenker, L., Worth, A., & Woll, A. (2013). Cohort Profile: The Motorik-Modul (MoMo) longitudinal study - Physical fitness and physical activity as determinants of health development in german children and adolescents. *International Journal of Epidemiology, 43*(5), 1410-1416.

**Keywords:** Longitudinal study; Gross motor coordination; Physical and mental health.

### An examination of the impact of increasing writing task demand on the handwritten performance of children with Developmental Coordination Disorder

A. Webb, S. E. Henderson & M. Stuart

Department of Psychology and Human Development, UCL Institute of Education, London W1A 0AH. a.webb@ioe.ac.uk

**Aim:** Under-achievement in school has long been recognized in children with Developmental Coordination Disorder (DCD) and several studies have focused on handwriting as a link between poor motor control and academic success. For example, recent studies have found that children with DCD produced less text than their typically developing (TD) peers and paused for 60% of a free-writing task. The importance of this finding was highlighted in earlier studies which found a consistent relationship between the amount children wrote and the composition quality of the output, suggesting that being able to produce enough text is a pre-requisite to writing good composition. Interpreting these results through a theory of limited cognitive capacity, it was suggested that lack of automaticity in the production of the handwriting served to slow children down, but little is known about how the nature of the writing task interacts with the handwriting difficulty to increase the impact of the disorder. The aim of this study was to test capacity theory by manipulating the cognitive load of different writing tasks in English children with and without DCD and measuring the relative responses.

**Method:** Fifteen 9-14 year-old children with measured DCD participated in the study together with 15 TD age, verbal ability and gender matched controls. Participants completed a specifically designed writing task in which a less demanding short copying task of 24 words lead on to a more demanding free writing task for the same allocation of time. The tasks were conducted on a digitising writing tablet using software which measured temporal, spatial and force components of the handwriting, when the pen was ‘in air’ as well as ‘on paper’.

**Results:** Results indicated that on the initial copying task the children in the DCD group took significantly longer to copy than those in the TD group, though there was no significant group difference in the proportion of time spent ‘in the air’ or ‘on paper’. On the free writing part of the task the children in the DCD group produced significantly less text than those in the TD group. However, the main group difference of the impact of increase in task demand (from copying to self-generated text) was on the relative time the pen spent on the page. An interaction was noted between group and level of task demand: although, both groups spent less time ‘on paper’ when the task demand increased, this reduction was significantly greater in the DCD group.

**Discussion:** This finding suggests that although children with DCD may be able to perform simple classroom tasks similarly to their unaffected peers, when task demand increases, the impact of the impairment may be felt more acutely, as indicated by a reduction in output. Further analysis of the temporal, spatial and force measures recorded during the two tasks for the two groups are discussed together with the implications of these differences for purposes of intervention.

**References**:

Christensen, C. A. (2009). The critical role handwriting plays in the ability to produce high-quality written text. In R. Beard, D. Myhill, J. Riley & M. Nystrand (Eds.), *The handbook of writing development*. London: SAGE Publishers.

Connelly, V., & Hurst, G. (2001). The influence of handwriting fluency on writing quality in later primary and early secondary education. *Handwriting Today, 2*, 5-57.

Prunty, M., Barnett, A. L., Wilmut, K., & Plumb, M.(2013). Handwriting speed in children with Developmental Coordination Disorder: Are they really slower? *Research in Developmental Disabilities, 34*, 2927–2936.

Rosenblum, S., & Livneh-Zirinski, M. (2008). Handwriting process and product characteristics of children diagnosed with developmental coordination disorder. *Human Movement Science, 27*, 200–214.

Torrance, M., & Galbraith, D. (2006). The processing demands of writing. In C.MacArthur, S. Graham & J. Fitzgerald (Eds.), *The handbook of writing research*. London: Guildford Press.

**Keywords:** Developmental Coordination Disorder; Handwriting; Written task.

### Diagnosing Developmental Coordination Disorder: A novel model for a research-integrated clinic

J.G. Zwicker^**1,2,3,4**^, E.C.R. Mickelson^**2,4**^ & J. Shen^**2,3**^

^1^Department of Occupational Science and Occupational Therapy, University of British Columbia, Vancouver, Canada. jill.zwicker@ubc.ca; ^2^Department of Pediatrics, University of British Columbia, Vancouver, Canad; ^3^Child and Family Research Institute, Vancouver, Canada; ^4^Sunny Hill Health Centre for Children, Vancouver, Canada.

**Aim:** Despite being one of the most common disorders in childhood, developmental coordination disorder (DCD) is often under-recognized and under-diagnosed (Blank et al., 2012). To address this gap, we have established the first research-integrated DCD diagnostic clinic in Canada. The *primary purpose* of the clinic is to: (1) provide assessment by a developmental pediatrician and an occupational therapist for consideration of a diagnosis of DCD; and (2) provide educational materials and recommendations to families of children diagnosed with DCD. The *secondary purpose* of the clinic is to establish a research database and collect additional data (e.g. attention, psychosocial well-being, participation, quality of life) from families who consent/assent.

**Method:** Family physicians or pediatric specialists can refer 4-12 year old children with a history of motor difficulties that affect activities of daily living and/or academic achievement. Children with a history of medical conditions that better explain motor difficulties (e.g., cerebral palsy, muscular dystrophy, genetic syndromes) or a history of intellectual disability that would prevent the child from completing a standardized motor assessment are referred to a more appropriate clinic. Children up to age 18 years who have a DCD diagnosis can be referred to the DCD Clinic for research purposes.

**Results:** Our clinic began in January 2014. Measures of success include > 75 referrals from the community in the first year, and we intend to expand the clinic to meet the demand. Families have expressed their gratitude for receiving a diagnosis and recommendations to support their child, and the majority of families have consented/assented to participate in the research database. In addition, medical trainees participate in each clinic; thus, we are teaching the next generation of pediatricians, developmental pediatricians, and family physicians about DCD.

**Discussion:** Our clinic fills a critical gap in providing a diagnosis and recommendations to families with DCD. In building a research database, we can begin to better understand the issues facing these children and inform better ways of meeting the holistic needs of this under-served population. Our research-integrated DCD clinic serves as a model for establishing a similar clinic and offers the potential for national and international collaborations to systematically collect similar data, thereby increasing sample size and our collective understanding of and service for children with DCD.

**References**:

Blank, R., Smits-Engelsman, B., Polatajko, H., & Wilson, P. (2012). European Academy for Childhood Disability (EACD): Recommendations on the definition, diagnosis, and intervention of developmental coordination disorder (long version). *Developmental Medicine and Child Neurology, 54,* 54-93.

**Keywords:** DCD; Diagnosis; Clinic; Research; Medical training.

## Oral Presentations

**Day 2: July 3, 2015**

### Correlations between elbow and wrist kinematics and handwriting performance among children with and without Developmental Coordination Disorder (DCD)

A. Abu-Ata^**1**^, D. Green^**1,2**^, T.S. Portnoy^**1**^, R. Sopher^**1,3**^ & N.Z. Ratzon^**1**^

^1^Department of Occupational Therapy, Tel Aviv University, Tel Aviv, 69978, Israel; ^2^Centre for Rehabilitation, Oxford Brookes University, Oxford OX3 3FL, UK. dido.green@brookes.ac.uk; ^3^Department of Mechanical Engineering, Imperial College, London, UK.

**Aim:** Children with Developmental Coordination Disorder (DCD) often experience difficulties in both the legibility and speed of handwriting. This study investigates relationships between handwriting outcomes and limb kinematics to characterise movement patterns of children with DCD and typically developing children (TDC).

**Method:** Levels of motor ability (MABC-2) and function (DCDQ-07) of children with and without DCD, matched for age, gender and parent education, were compared across handwriting tasks using a standardized handwriting assessment of copying and dictation (A-A Handwriting). A 3-D motion capture system (Qualysis) was used to analyze upper limb kinematics and characterise movement patterns and contrasted with output in copying and dictated tasks.

**Results:** Thirty children (DCD, n=15 and non-DCD n=15; mean age 8.05; SD 11.1( participated in the study. Attention deficits and writing hours per week were significant factors correlating with outcome, with children with DCD more likely to show attention deficit (t=4.34,p<0.01). Significant differences were evident in productivity with children with DCD writing fewer legible letters in the first minute than TDC in both copying (mean difference -5.6; t=4.17,p<0.001) and dictation (mean difference -5.6;t=3.25,p<0.01) tasks. Partial correlations, controlling for attention, reflected differences in kinematic factors between groups. Comparisons of movement kinematics show higher values of maximal elbow extension associated with increased letters per minute in both groups in the first minute of writing (r>0.69;p<0.05) but only for children without DCD in the second minute (r=0.63,p<0.05). More atypical wrist postures of excessive wrist extension and flexion (larger movements), were associated with the production of fewer letters per minute for children with DCD (r=-0.77,p<0.001, r=-0.65,p<0.01 respectively). Children with DCD also showed poor maintenance of the right and left margins in both copying and dictation (t>5.5,p<0.001 all).

**Discussion:** Children with DCD showed differences in movement patterns between copying and dictation in handwriting; whereas TDC showed more consistency and less variability between the two handwriting tasks. Children with DCD also showed a lack of automisation of key writing concepts such as maintenance of margins in both copying and dictation. These results provide insights into the mechanisms underpinning handwriting problems in children with DCD and can promote our understanding of this condition and aid development of optimal treatment plans

**Keywords:** Developmental Coordination Disorder; 3-D motion analysis; Kinematics; Handwriting.

### A critical test of the internal modeling deficit in children with DCD via motor imagery, action planning and online control tasks

I.L.J. Adams^**1**^, J.M. Lust^**1**^, P.H. Wilson^**2**^ & B. Steenbergen^**1,2**^

^1^Behavioural Science Institute, Radboud University Nijmegen, PO Box 9104, 6500 HE Nijmegen, the Netherlands. i.adams@pwo.ru.nl; ^2^School of Psychology, Australian Catholic University, Melbourne 3065, VIC Australia.

**Aim:** Earlier studies (Wilson *et al*. 2013; Adams *et al*., 2014) have shown that the predictive control of movements is impaired in children with DCD, most likely due to a deficit in the internal modeling of movements. The aim of this study was to examine different parts of the internal modeling of movements in children with DCD by conducting tests of motor imagery, motor planning and rapid online control within one group of children with DCD. This study is the first to provide a comprehensive test of the internal modeling deficit hypothesis. The current study will increase fundamental insights into the etiology of this motor disorder and the aspects of disordered motor control that should be targeted in future intervention.

**Method:** Participants were 66 children; 33 children (26 boys and 7 girls) between the ages of 6 and 11 years who met the DSM-V diagnostic criteria for DCD and 33 individual gender and age-matched controls. Motor imagery was assessed with the hand rotation task (HRT) and the radial virtual guided pointing task (VGPT). Action planning was assessed with the dowel task that tested planning for end-state comfort. Rapid online control was tested with the double step pointing task.

**Results:** Results showed that children with DCD were slower and less accurate than the control group in the HRT. Surprisingly, both DCD and control group had difficulties with the imagery of movements in the VGPT. In the action planning task results showed that the control group ended significantly more often in a comfortable end position than the DCD group. The rapid online control task showed no difference between DCD and control group on all measures.

**Discussion:** Our results show that the content and execution of the internal model of a movement is impaired in children with DCD, evidenced by the results on motor imagery and action planning tasks. However, the rapid online control task showed no deficit in the detection of online perturbations and use of online feedback loops. Based on these results, an interesting avenue would be study whether motor imagery training can help children with DCD to improve their motor skills.

**References**:

Adams, I.L.J., Lust, J.M. Wilson, P.H., & Steenbergen, B. (2014). Compromised motor control in children with DCD: A deficit in the internal model? – A systematic review. *Neuroscience & Biobehavioral Reviews, 47C*, 225-244.

Wilson, P.H., Ruddock, S., Smits-Engelsman, B., Polatajko, H., & Blank, R. (2013). Understanding performance deficits in developmental coordination disorder: a meta-analysis of recent research. *Developmental Medicine and Child Neurology, 55*(3), 217-228.

**Keywords:** Developmental Coordination Disorder; Internal modeling deficit; Motor imagery; Action planning; Rapid online control.

### Locomotor behaviour of individuals with and without Developmental Coordination Disorder while navigating through apertures

A.L. Barnett, K. Wilmut & W. Du

Perception and Motion Analysis (PuMA) Lab, Department of Psychology, Social Work and Public Health, Oxford Brookes University, UK. abarnett@brookes.ac.uk

**Aim:** As we walk around our environment we are constantly faced with obstacles such as gaps or apertures, when these are narrow they force us to rotate our shoulders in order to pass through. In adults and children the decision to rotate the shoulders is based on both body size and movement control; where movement is more variable, walkers adopt a safer strategy rotating their shoulders for smaller apertures. It is generally observed that individuals with Developmental Coordination Disorder (DCD) have a tendency to bump into obstacles, yet exactly why this is the case is unclear. The aim of this study was therefore to determine the developmental pathway of how individuals with DCD make action judgments and movement adaptations while navigating apertures.

**Method:** 15 children (12-17 years) and 15 adults (18-30 years) were recruited in line with DSM-5 diagnostic criteria for DCD. Typically developing (TD) controls were individually matched for age and gender. Participants walked up to and passed through a series of apertures, which were scaled to their body size (from 0.9-2.1 times shoulder width). Reflective markers placed on the shoulders and trunk were tracked with a VICON motion analysis system. Spatial and temporal characteristics of movement speed and shoulder rotation were collected over the initial approach phase and while crossing the aperture threshold.

**Results:** The decision to rotate the shoulders was not scaled in the same way for the DCD and TD groups, with individuals with DCD turning their shoulders at larger apertures compared to the TD individuals when body size alone was accounted for. In terms of the movement adaptations, the individuals with DCD made an earlier and greater speed reduction compared to their typical peers when a shoulder rotation was required. A significant group (DCD and TD) by age (child and adult) interaction reflected greater improvement between the TD child and adult groups, compared to improvement between the DCD child and adult groups, with the latter showing an underdeveloped adaptive pattern.

**Discussion:** The findings provide a better understanding of the different navigation patterns in individuals with DCD. Their adaptive strategy, which results in them turning more often and to a greater degree than their typical counterparts, is coupled with a more cautious approach strategy giving them more time in which to make the necessary movement adaptations to cross the aperture. Therefore, when these individuals are in more complex environments this adaptive strategy may prove impossible to implement, when there is not additional time to allow for a slower movement and thus a collision may occur.

**Keywords:** Action capabilities; Locomotion; Obstacle avoidance; Movement adaptation.

### Oculomotor control in DCD children with and without developmental dyslexia: What is the impact of co-occurrence between neurodevelopmental disorders?

S. Bellocchi^**1**^, A. Huau^**2**^, M. Jover^**2,4**^, F. Brun-Hénin^**3**^, J. Mancini^**3**^ & S. Ducrot^**4**^

^1^Epsylon Research Unit EA 4556, University of Montpellier & Paul Valéry University, Montpellier, 34000, France. stephanie.bellocchi@univ-montp3.fr; ^2^Centre PsyCLE EA 3273, 13621, Aix-Marseille University, Aix en Provence, France; ^3^APHM, Salvator Hospital, CERTA (Centre of reference for learning disabilities), Marseille, France; ^4^Laboratoire Parole et Langage, UMR 7309, CNRS & Aix-Marseille University, Aix en Provence, France.

**Aim:** In previous studies, we showed that the perceptual low-level pre-processing step that is responsible for landing position pattern in oculomotor control is not yet implemented in dyslexic children without developmental coordination disorder (DCD). This result has been interpreted with the idea that saccade computation (i.e. *where* and *when* the eyes land in a word) is not dysfunctional but rather a mirror of their less reading exposure^1^ (e.g. Bellocchi et al., 2013). In the same vain, some studies suggest a delay in the maturation of low-level oculomotor skills in children with DCD (e.g. pursuit system, Robert et al., 2014)^2^. Considering that DCD and developmental dyslexia (DD) are often associated, the aim of the present study was to investigate the role of co-occurrence between these two disorders in saccade computation.

**Method:** We assessed the eye movements of four groups of children (12 DCD children with DD, 11 DCD children without DD, 20 DD children without DCD and 29 typical developing children – mean age: 117,3 months; SD: 10,2 months) using an oculomotor bisection task (participants were asked to move their eyes to a position they thought to be the middle of the stimulus). The type of stimulus - linguistic factor (words vs non-linguistic stimuli) and discreteness (lines vs strings of hashes) - in combination with the stimulus presentation side (left vs. right) were manipulated.

**Results:** Main results showed differences between groups with regard to saccade latency: “DCD + DD” and “DCD only” groups were slower compared to typical developing children in programming their first saccade. Moreover, in the LVF those two groups made shorter saccade size, suggesting that they struggled to reach the preferred viewing location (PVL) in this visual side. Furthermore, those two groups seem to suffer more for the absence of discreteness of the stimuli in order to compute their saccades and move their eyes in the middle of the stimulus. Finally, results seem to suggest differences between children with DCD (with or without DD) and children with DD only.

**Discussion:** Overall, it seems that no differences emerged between “DCD+DD” and “DCD only” groups, suggesting that co-occurrence in itself does not sharpen difficulties in saccade computation. However, the presence of a motor disorder seems to generate a dysfunction in saccade computation. These results strengthen the importance to take into account oculomotor control involved in visual word recognition for clinical assessment and intervention in children with DCD.

**References**:

1. Bellocchi, S., Mancini, J., Jover, M., Huau, A., Ghio, A., André, C., & Ducrot, S. (2013). *Dyslexic readers and saccade computation: effects of reading exposure and visuo-perceptual constraints.* Poster at the XVIII ESCoP Conférence. Budapest, Hungary, september.

2. Robert, M.P., Ingster-Moati, I., Albuisson, E., Cabrol, D., Golse, B., & Vaivre-Douret, L. (2014). Vertical and horizontal smooth pursuit eye movements in children with developmental coordination disorder. *Developmental Medecine & Child Neurology, 56*, 595–600.

**Keywords:** Saccade computation; Eye movements; Developmental dyslexia; Developmental coordination disorder; Co-occurrence.

### Manual function outcome measures in children with developmental coordination disorders: a systematic review

E. Bieber^**1**^, B. Smits-Engelsman^**2**^, G. Sgandurra^**1**^, G. Cioni^**1**^, H. Feys^**3**^, A. Guzzetta^**1**^ & K. Klingels^**3-4**^

^1^IRCCS Fondazione Stella Maris, Department of Developmental Neuroscience, Pisa, Italy. e.bieber@fsm.unipi.it; ^2^KU Leuven - University of Leuven, Department of Kinesiology, Leuven, Belgium; ^3^KU Leuven - University of Leuven, Department of Rehabilitation Sciences, Leuven, Belgium; ^4^REVAL Rehabilitation Research Center, BIOMED, Hasselt University, Diepenbeek, Belgium.

**Aim:** This study systematically reviewed the clinical and psychometric properties of manual function outcome measures for children with developmental coordination disorder (DCD).

**Method:** Three electronic databases were searched to identify studies (1980-2014), using outcome measures assessing manual function at the ICF-CY body function and/or activity-participation level in children with DCD aged 3-18 years old. Handwriting measures were excluded. Search strategy included terms identifying childhood, children with DCD and outcome measures for manual function. Study selection was conducted by two blind assessors. CanChild Outcome Measures Rating Form was used to report the psychometric properties and clinical utility.

**Results:** A total of 12 studies involving 16 assessment tools were identified including 7 clinical tests, 3 instruments using a naturalistic observation and 7 questionnaires. The tests measure main manual function components. The Hand-Held Dynamometer and the Functional Strength Measurement focus on strength while five other assessments (the Movement Assessment Battery for Children, mABC-2; the Bruininks-Oseretsky Test of Motor Proficiency-2, BOT-2; the McCarron Assessment Neuromuscular Development; the Zurich Neuromotor Assessment; the In-Hand Manipulation Test) evaluate aspects of fine motor control, manual dexterity, bimanual coordination and in-hand manipulation. Reliability and validity properties are reported with adequate results for the fine-motor subdomains of the mABC-2 and the BOT-2. The naturalistic observations (the Functioning in Children with Developmental Coordination Disorder, the Assessment of Children’s Hand Skills, and the “Do-Eat”) provide an assessment of daily manual tasks. Adequate reliability of the naturalistic observations has been shown but concurrent validity has been less evaluated. Finally four questionnaires (the Developmental Coordination Disorder Questionnaire’07, the Little Developmental Coordination Disorders Questionnaire, the Daily Functioning in Children with Developmental Coordination Disorder Questionnaire, the Children Activity Scale-Parents/Teachers) target daily general performance in children with DCD. The Children’s Hand-Skill ability Questionnaire focuses specifically on manual ability, but targets a broad range of pediatric populations. Reliability and validity of questionnaires was investigated into limited extent. Data on measurement error were only available for strength measures and one age band of mABC-2.

**Discussion:** This review shows that a combination of different tools might be useful for a comprehensive assessment of manual function in children with DCD. However, further investigation of psychometric properties of those tools in children with DCD are warranted. Also other tests adopted in other populations could be used for measuring manual dexterity.

**Keywords:** Assessment; Children; Manual function; Developmental Coordination Disorder.

### Cortical thickness in Developmental Coordination Disorder and/or Developmental Dyslexia

M. Biotteau^**1,2**^, Y. Chaix ^**1,2,3**^, P. Celsis^**1,2**^, N. Chauveau^**1,2**^, P. Péran^**1,2**^ & J.-M. Albaret^**4**^

^1^Inserm, Imagerie Cérébrale et Handicaps Neurologiques UMR 825, CHU Purpan, Place du Dr Baylac, F-31059 Toulouse Cedex 9, France. maelle.biotteau@inserm.fr; ^2^Université de Toulouse, UPS, Imagerie Cérébrale et Handicaps Neurologiques UMR 825, CHU Purpan, Toulouse, France; ^3^Hôpital des Enfants, Centre Hospitalier Universitaire de Toulouse, CHU Purpan, Toulouse, France; ^4^Université de Toulouse III, UPS, PRISSMH-EA4561, Toulouse Cedex 9, France.

**Aim:** Understanding the reasons of frequent comorbidity between Developmental Coordination Disorder (DCD) and Developmental Dyslexia (DD) is a real challenge. Their high association frequency (40 to 60%) suggests common etiological bases. Yet, however DCD and DD are associated with functional and structural brain alterations, their association has never been studied in terms of brain development. The purpose of this study is therefore to examine whether the pattern of changes for concurrent disorders is distinct from the alterations seen in single disorders and above all whether the effects of comorbidity influence cortical thickness. We aim to identify, amongst others, if the co-occurrence of DD and DCD is associated with a distinct global pattern of cortical thickness, in order to highlight the unique or associate neurobiology of comorbid neurodevelopmental disorders.

**Method:** 65 right-handed children (21♀-44♂- range 8–12y) with DCD or DD or DCD and DD in accordance with the DSM-IV-TR criteria (M-ABC test for motor skills and L’Alouette and ODEDYS-2 test for reading skills) were recruited into the study. They had no history of neurological or psychiatric disorder and no cons-indication for a MRI. Children with Specific Language Impairment, Attention Deficit/Hyperactivity Disorder and an IQ less than 70 were excluded. Participants were scanned one time and 9 children were lost due to incomplete scans or excessive motion. The remaining 56 participants (19 DD, 20 DCD and 17 DD+DCD) composed our total sample of children for MRI data analysis.

**Results:** Cortical thickness analysis was performed to determine the patterns of cortical thickness/thinning in each disorder separately and in association. The patterns of cortical thickness were then compared across groups. First results already denote significant differences in cortical thickness in the motor, language and attentional areas (frontal, parietal, and temporal lobes) depending on the considerate group. Data analysis is currently in progress.

**Discussion:** Cortical thinning has been recently been reported in the literature for DCD + ADHD (Langevin, 2014). Our results should be compared to these findings and helpful to better understand comorbidity and may provide clues to both the isolated and the shared neurobiological origins of motor and language disorders.

**References:**

Langevin, L. M., MacMaster, F.P., & Dewey, D. (2014). Distinct patterns of cortical thinning in concurrent motor and attention disorders. *Developmental Medicine & Child Neurology, 57*(3), 257-264.

**Keywords:** Comorbidity; Developmental Coordination Disorder; Developmental Dyslexia; MRI; Cortical thickness.

### Examining relationships between online control of reaching-to-grasp and standardized motor tests in healthy adults and children

**C. Blanchard**^1^, M. **Younas**^2^, R. **Sperring**^3^ & NP. **Holmes**^1^

^1^School of Psychology, University of Nottingham, University Park, Nottingham NG7 2RD, UK. Caroline.Blanchard@nottingham.ac.uk; ^2^School of Psychology and Clinical Language Sciences, University of Reading, UK; ^3^School of Psychology, University of New South Wales, Sydney, Australia.

**Aim:** We address individual variability in rapid visuo-motor control among healthy adults and children (7-10 years old), and how this variability in behaviour corresponds with abilities on standardised motor tests.

**Method:** We used a ‹double-step› reaching and grasping task to measure how long it took children (n=32) and adults (n=21) to alter their movement when reaching to grasp an illuminated ball. At the start of each trial, a single, central target ball was illuminated. On 60% of trials, the central ball remained lit throughout, but on the remaining 40% of trials, at the onset of movement the illumination switched from one ball to another in a centre-out configuration (left, right, up, or down). We measured the position of the index finger and thumb, and extracted the latency to initiate a movement correction from velocity profiles. We combined this task with a series of standardised tests of motor coordination skills (i.e., the MABC-2), cognitive performance (BAS-II), autistic traits (AQ-child) and attention deficit/hyperactivity disorder (Conners 3). In the adult sample, we used the MABC-2 (11-16 years) test along with standardised manual dexterity tasks - the Grooved and Perdue pegboards.

**Results:** Our novel task resulted in trajectory correction times longer than those typically reported in the adult literature, which may be due to the increased cognitive and biomechanical complexity of switching movements in four different directions. Our initial analyses show that, in adults, the latency to initiate trajectory corrections was very well-correlated (across participants) between the four different movement directions. Between tasks, correction latency was well correlated with the Purdue task, particularly the assembly task, and less well correlated with grooved pegboard performance. Perhaps surprisingly, correction latency did not correlate with MABC-2 total score or any subscale. In children, latencies to correct movements were ~60ms longer than in adults, and correction latency correlated significantly with the aiming and catching subscale of the MABC-2, but not with the other subscales or the total score. Corrections towards the left or right targets were made significantly earlier than towards the upper or lower targets, in both adults (26ms), and children (24ms).

**Discussion:** The novel centre-out reaching task may provide a measure of hand-eye coordination skills that is independent from other standardized tasks. It provides a continuous and high-resolution scale of measurement, and is directly comparable between adults and children, which makes it a promising task for further study.

**Keywords:** MABC-2; Hand; Motor Control; Pegboard.

### Symposium: Retrospective on the work of Dr. Dawne Larkin

M.H. Cantell^**1**^, D. Dewey^**2**^, B. Hands^**3**^ & E. Rose^**3**^

^1^Department of Special Needs Education and Youth Care, Faculty of Behavioral and Social Sciences, University of Groningen. m.h.cantell@rug.nl; ^2^Department of Pediatrics, University of Calgary, Alberta, Canada; ^3^Institute for Health Research, Notredame University, Australia. beth.hand@nd.edu.au

**Aim and Method:** This paper is a retrospective of the remarkable contribution of Dr. Dawne Larkin to the field of Developmental Coordination Disorder between the years of 1980 and 2013. We will draw on numerous sources including over 50 papers, 2 books, 9 book chapters, 30 student theses, over 80 conference papers and a screening test. What was unique about Dawne was her multidisciplinary interest in issues related to movement development. She collaborated with researchers from around the world in neurology, pediatrics, psychology, occupational therapy, physiotherapy, speech pathology.

**Results and Discussion:** Six main themes characterize Dawne’s teaching and research in the DCD field. Her early research focused on the* mechanisms of DCD* relating to laterality, motor asymmetry and the interaction of structure and function. She was also interested in identifying subtypes of DCD, involving kinaesthesia and soft motor signs, as well as DCD children with overlapping disorders such as ADHD. The second area in which she was very active was *identification, screening and assessment*. She developed a well-used screening tool, Stay in Step (Larkin & Revie, 1994) and spent considerable time examining the psychometric properties and protocols of motor skill assessments, in particular the McCarron Assessment of Neuromuscular Development (MAND). A third interest area related to *physical activity and fitness* in children and adolescents with DCD. She was especially skillful in understanding the constraints and benefits of fitness based on her broad theoretical and practical knowledge of anatomy, biomechanics, motor control and exercise physiology. A fourth interest involved *the daily activities* and routines of children with DCD and how motor problems constrained their participation, and that of their families in activities of daily life. The fifth theme she was well-known for was *intervention*. Her development of the Unigym program was based on a task-specific framework which has since been extensively applied around the world. Throughout her career she was also particularly passionate about the influence of *gender* and had a strong belief that assessment and intervention models should pay attention to the underlying biological, psychosocial and environmental differences that are unique to each person. In sum, Dr. Larkin made a remarkable contribution to the scientific literature not only in DCD but also in the more general area of child development.

**Selected references**:

Armitage, M., & Larkin, D. (1993). Laterality, motor asymmetry and clumsiness in children. *Human Movement Science, 12*, 155-177.

Cantell, M., Kooistra, L. & Larkin, D. (2001). Approaches to intervention for children with Developmental Coordination Disorder. *New Zealand Journal of Disability Studies, 9*, 106-119.

Cermak, S. A., & Larkin, D. (2002). *Developmental coordination disorder*. Albany, NY: Delmar Thomson Learning.

Hands, B., & Larkin, D. (2006). Physical activity measurement methods for young children: A comparative study. *Measurement in Physical Education and Exercise Science, 10*, 203-214.

Hands, B., & Larkin, D. (2006). Physical fitness differences in children with and without motor learning difficulties. European *Journal of Special Needs Education, 21*, 447-456.

Hands, B. P., Chivers, P. T., Parker, H. E., Beilin, L., Kendall, G., & Larkin, D. (2011). The associations between physical activity, screen time and weight from 6 to 14 yrs: the Raine Study*. Journal of Science and Medicine in Sport, 14*, 397-403.

Hoare, D., & Larkin, D. (1991). Kinaesthetic abilities of clumsy children. *Developmental Medicine & Child Neurology, 33*, 671-678.

Larkin, D., & Hoare, D. (1991). *Out of Step: Coordinating kids’ movement*. Active Life Foundation.

Larkin, D., & Hoare, D. (1992). The movement approach: A window to understanding the clumsy child. *Approaches to the Study of Motor Control and Learning, 84*, 413-439.

Licari, M., Larkin, D., & Miyahara, M. (2006). The influence of developmental coordination disorder and attention deficits on associated movements in children. *Human Movement Science, 25*, 90-99.

Männistö, J. P., Cantell, M., Huovinen, T., Kooistra, L., & Larkin, D. (2006). A school-based movement programme for children with motor learning difficulty. *European Physical Education Review, 12*, 273-287.

O’Beirne, C., Larkin, D., & Cable, T. (1994). Coordination problems and anaerobic performance in children. *Adapted Physical Activity Quarterly, 11*, 141-141.

Revie, G., & Larkin, D. (1993). Task-specific intervention with children reduces movement problems. *Adapted Physical Activity Quarterly, 10*, 29-29.

Rose, B., Larkin, D., & Berger, B. G. (1997). Coordination and gender influences on the perceived competence of children. *Adapted Physical Activity Quarterly, 14*, 210-221.

Summers, J., Larkin, D., & Dewey, D. (2008). Activities of daily living in children with developmental coordination disorder: dressing, personal hygiene, and eating skills. *Human Movement Science, 27*, 215-229.

Tan, S. K., Parker, H. E., & Larkin, D. (2001). Concurrent validity of motor tests used to identify children with motor impairment. *Adapted Physical Activity Quarterly, 18*, 168-182.

**Keywords:** DCD; Mechanisms; Identification; Intervention; ADL; Fitness; Gender.

### Gestures and related skills in Developmental Coordination Disorder and Developmental Dyspraxia: A production-system deficit?

O. Costini^**1,2**^, A. Roy^**1,3,4**^, S. Faure^**5**^, C. Remigereau^**1,3**^, E. Renaud^**3**^, L. Blanvillain^**6**^, C. Fossoud^**2**^& D. Le Gall^**1,7**^

^1^Laboratory of Psychology EA4638, University of Angers, LUNAM, Angers cedex 01, 49045, France. orianne.costini@gmail.com; ^2^Pediatric Unit for Learning Disabilities, University Hospital of Nice, France; ^3^Reference Center for Learning Disabilities, University Hospital of Nantes, France; ^4^Neurofibromatosis Clinic, University Hospital of Nantes, France; ^5^University of Nice-Sophia Antipolis, EA7278, Nice, France; ^6^Regional Center for Functional Rehabilitation of Angers, France; ^7^Neuropsychology Unit, Department of Neurology, University Hospital of Angers, France.

**Aim:** The present study analyzed the nature of praxis difficulties in children with Developmental Coordination Disorder (DCD) by comparing their performance with that of typically developing children across tasks and conditions that allow exploring distinct components of the functional architecture hypothesized by cognitive models of praxis processing in adults^1,2^. Within this theoretical framework, the study investigated the extent to which the gestural difficulties of children with DCD are related to a deficit of the production system. According to this hypothesis, DCD children perform significantly less well than do typically developing controls on all gesture production tasks, but have spared performance on conceptual tasks. We also examined the impact of other cognitive functions involved in gesture production.

**Method:** Tasks were selected in order to enable a comprehensive assessment of gestures, including conceptual tasks (knowledge about tool functions and about actions; recognition of transitive gestures), representational (transitive, intransitive), and non-representational gestures (imitation of finger, hand, and bimanual postures). We realized an additional assessment of constructional abilities and cognitive domains known to be involved in gestural development: intellectual efficiency (Intelligence Quotient), executive functions, visual-perceptual and visuospatial functions. Participants between the ages of 7 and 13 years included 30 DCD children and 30 typically developing children matched for age, gender, handedness and socio-economic status.

**Results:** The DCD children exhibited impaired performance for both representational transitive and nonrepresentational gestures, with impaired visuospatial skills. Differences between groups remained significant when controlling for a measure of visuospatial skill only for representational transitive gestures. This dysfunction could not be related to a semantic deficit, an impairment of sensorimotor knowledge (gesture engrams), or a neuromotor dysfunction, since the clinical examinations were normal.

**Discussion:** The fact that children with Developmental Coordination Disorder were impaired for both representational transitive and nonrepresentational gestures suggests a deficit of the production system. When considering an explanation in terms of a dysfunction of visuospatial processing, the main question being whether the underlying deficit is specific to gestures and motor actions. Further researches are needed to explore whether another line of explanation could concern the mechanisms of predictive motor control for both manual and oculomotor activities.

**References**:

Rothi, L.J.G., Ochipa, C., & Heilman, K. M. (1991). A cognitive neuropsychological model of limb praxis. *Cognitive Neuropsychology, 8,* 443–458.

Roy, E. A., & Square, P. A. (1985). Common considerations in the study of limb, verbal and oral apraxia. In E. A. Roy (Ed.), *Neuropsychological studies of apraxia and related disorders* (pp. 111–161). Amsterdam: Elsevier.

**Keywords:** Gestures; Visuospatial processing; Developmental Coordination Disorder; Developmental dyspraxia.

### Very preterm birth and developmental coordination disorder at school age: results from the area-based Italian ACTION follow-up study

M. Cuttini^**1**^, I. Croci^**1**^, M. Lacchei^**1**^, G. Riccio^**1**^, C. Giorno^**1**^, C. Rosa^**1**^ & G. Giana^**1**^

^1^Research Unit of Perinatal Epidemiology, Pediatric Hospital Bambino Gesu’, 00146, Rome, Italy. marina.cuttini@opbg.net

**Aim:** This study was aimed at screening for DCD in a large area-based population of school-age children born very preterm (i.e. before 32 weeks gestation), and exploring comorbidities and risk factors.

**Method:** Very preterm children born in Lazio region in 2003-04 and survived to school age were included. The Kaufman Assessment Battery second edition (KABC-II) and selected items of NEPSY-II were used to assess cognitive and neuropsychological development. Parents completed the Conners’ Parent Rating Scale Revised-long form (CPRS-R:L) and the ICAP questionnaire to assess ADHD traits and eating problems respectively. Screening for DCD was carried out through the Developmental Coordination Disorder Questionnaire (DCDQ, Italian version1). 427 children participated in the study (response rate 70.6%). In 27 cases, the DCDQ was not completed. For this analysis, children with cerebral palsy (n.36), vision (n.7) and hearing (n.5) problems were excluded, together with cases of severe cognitive impairment (KABC-MPI < 2SD, n.24). Thus, 328 cases were analyzed. Multiple logistic regression analysis was used to assess the relation between perinatal and infancy risk factors and DCD score.

**Results:** Mean gestational age was 29.1 weeks (SD 1.8). 144 children were females (43.9%) and 87 (26.5%) multiples. 49 (14.9%) were born from non Italian mothers. Mean age at assessment was 9 years (SD 0.97). Overall DCD score had a mean of 57.3 (SD 10.9). 146 children (44.5%) had a DCD score indicating/suggesting DCD. These children were significantly more likely to screen positive for ADHD (p=0.026) and eating problems (p=0.017). At multivariable analysis, factors significantly associated with positive DCD screening results were male gender, older maternal age (≥ 35 years), higher illness severity score (combining base deficit and oxygen requirements in the first 12 hours of life) and lower cognition measured at 2 years of age corrected for prematurity2.

**Discussion:** In this large area-based sample of very preterm children, almost half screened positive for DCD. Male gender, older maternal age, early neonatal respiratory and cognitive problems appeared to be significant predictive factors, whose relevance should be tested after DCD diagnosis confirmation.

**Acknowledgements:** The ACTION follow-up project was funded by the Italian Ministry of Health and by Chiesi Farmaceutici S.p.A. (unrestricted grant).

**References**:

1. Caravale, B., Baldi, S., Gasparini, C., & Wilson, B. N. (2014). Cross-cultural adaptation, reliability and predictive validity of the Italian version of Developmental Coordination Disorder Questionnaire (DCDQ). *European Journal of Paediatric Neurology, 18*(3), 267-272.

2. Cuttini, M., Ferrante, P., Mirante, N., Chiandotto, V., Fertz, M., Dall’Oglio, A. M. Cognitive assessment of very preterm infants at 2-year corrected age: Performance of the Italian version of the PARCA-R parent questionnaire. *Early Human Development, 88*(3), 159-163.

**Keywords:** Very preterm births; Area-based cohort; Developmental Coordination Disorder; Risk Factors.

### The neurobiology of co-occurring Developmental Coordination Disorder and Attention-Deficit/Hyperactivity Disorder provides clues for genetic research

D. Dewey^**1,2,9,12**^, L.M. Langevin^**1,9**^, K. McLeod^**4,9**^, S.K. Thornton^**5**^, K. Ten Eckye^**1,9,12**^, S. Bray^**1,6,9**^, F. MacMaster^**1,7,9,10**^, C. Lebel^**1,7,9**^, B.G. Goodyear^**6,7,8,10,11**^, F.P. Bernier^**3,9**^

^1^Department of Pediatrics, University of Calgary, Alberta, Canada. dmdewey@ucalgary.ca; ^2^Department of Community Health Sciences, University of Calgary, Alberta Canada; ^3^Department of Medical Genetics, University of Calgary, Alberta, Canada; ^4^Department of Medical Sciences, University of Calgary, Alberta, Canada; ^5^Department of Biological Sciences, University of Calgary, Alberta Canada; ^6^Department of Radiology, University of Calgary, Alberta, Canada; ^7^Departments of Psychiatry, University of Calgary, Alberta, Canada; ^8^Department of Clinical Neurosciences, University of Calgary, Alberta, Canada; ^9^Alberta Children’s Hospital Research Institute, University of Calgary, Alberta, Canada; ^10^Hotchkiss Brain Institute, University of Calgary, Alberta, Canada; ^11^Seaman Family MR Research Centre; ^12^Behavioural Research Unit, Alberta Children’s Hospital, Calgary, Canada.

**Aim:** Developmental coordination disorder (DCD) and attention-deficit/hyperactivity disorder (ADHD) co-occur in up to 50% of cases^1^. Previous imaging studies have typically looked at these disorders in isolation. This research investigated whether children with isolated or co-occurring DCD and ADHD display differing brain structure and function.

Methods: All imaging took place on the same GE 3T system. The imaging protocol consisted of anatomical imaging (T1-weighted), diffusion tensor imaging (DTI), resting-state fMRI and task-based fMRI, Children with DCD only, ADHD only, co-occurring DCD/ADHD and controls participated. Cortical thickness, diffusion tensor imaging, resting state fMRI and task-based fMRI were conducted. Motor, cognitive and neuropsychological functions were assessed using standardized tests and correlated to fMRI measures.

**Results:** Children with co-occurring DCD+ADHD demonstrate significant widespread decreases in cortical thickness in the frontal, parietal and temporal lobes, whereas children with DCD only demonstrate the cortical thickness reductions in regions of the temporal lobe associated with attention and spatial memory^2^. Analysis of DTI data revealed reductions in fractional anisotropy (FA) in the posterior region of the corpus in children DCD and comorbid DCD and ADHD. Abnormalities in the frontal region of the corpus callosum were found in children with ADHD and co-occurring DCD and ADHD. Finally, higher radial diffusivity values were found in in the callosal region subserving the primary and supplementary motor areas in the DCD+ADHD group^3^. Resting state fMRI revealed that the bilateral inferior frontal gyri, the right supramarginal gyrus, angular gyri, insular cortices, amygdala, putamen and pallidum exhibited similar alterations in functional connectivity (FC) in children with DCD and/or ADHD compared to controls. However, compared to children with only DCD or ADHD, the DCD+ADHD group exhibited greater FC between the primary motor cortex and brain regions involved in motor control (bilateral caudate, left premotor cortex, right inferior frontal gyrus), speech processing and prosody (bilateral anterior superior temporal gyri), sensorimotor processing (left postcentral gyrus, right parietal operculum cortex, bilateral precuneus cortices, angular gyri), and attention and error detection (bilateral anterior cingulate cortices)^4^. Finally, on task-based fMRI (i.e., GO-NOGO) children with co-occurring DCD+ADHD showed decreased brain activation compared to controls in the bilateral inferior parietal lobules, superior parietal lobules, right angular gyrus, left precentral gyrus and left inferior frontal gyrus. No significant differences were found between the DCD only, ADHD only and control groups^5^.

**Discussion:** The co-occurrence of DCD+ADHD was associated with distinct alterations in brain structure and function. The global pattern of cortical thickness decrease seen in children with co-occurring DCD and ADHD highlights the unique neurobiology of comorbid neurodevelopmental disorders. The novel neurobiological features associated with co-occurring disorders may inform diagnostic definitions and provide clues to both the shared and the isolated neurobiological and genetic origins of motor and attention disorders^6^.

**References:**

1. Kaplan, B. J., Wilson, B. N., Dewey, D., & Crawford, S. G. (1998). DCD may not be a discrete disorder. *Human Movement Science, 17*, 471-490.

2. Langevin, L. M., MacMaster, F. P., & Dewey, D. (2015). Distinct patterns of cortical thinning in concurrent motor and attention disorders. *Developmental Medicine & Child Neurology, 57*(3), 257-264.

3. Langevin, L. M., MacMaster, F. P., Crawford, S., Lebel, C., & Dewey, D. Common white matter microstructure alterations in pediatric motor and attention disorders. *The Journal of Pediatrics, 164*(5), 1157-1164.

4. McLeod, K. R., Langevin, L. M., Goodyear, B. G., & Dewey, D. (2014). Functional connectivity of neural motor networks is disrupted in children with developmental coordination disorder and attention-deficit/hyperactivity disorder. *NeuroImage: Clinical, 4*, 566-575.

5. Thornton, S.K., Bray, S., Langevin, L., & Dewey, D. (2015). Functional brain activation during a motor inhibition task in children with developmental coordination disorder and attention deficit/ hyperactivity disorder. *Journal of the International Neuropsychological Society. 21*.

6. Bernier, F. P., Mosca, S. J., Langevin, L., Innes, A. M., Loinel, A. C., Marshall, C. C., Scherer, S.W., Parboosingh, J. S., & Dewey, D. (2015). Copy-number variation in canadian children with Developmental Coordination Disorder implicates neurodevelopmental genes. *Journal of the International Neuropsychological Society. 21*.

**Keywords:** Co-occurring Disorders; Developmental Coordination Disorder; Attention Deficit/Hyperactivity Disorder; Neurobiology; Neuroimaging; Genetics.

### Level walking in individuals with and without Developmental Coordination Disorder: an analysis of movement variability

W. Du, K. Wilmut & A.L. Barnett

Perception and Motion Analysis (PuMA) Lab, Department of Psychology, Social Work and Public Health, Oxford Brookes University, UK. wdu@brookes.ac.uk

**Aim:** As an important everyday locomotor skill, walking has been examined in individuals with DCD and is anecdotally reported to be poorly executed in this population. However, studies have produced mixed results, with some reporting a shortened stride length or shorter double support, and some finding no quantitative differences between children with DCD and their typically developing peers. Thus the standard measures of foot placement used in previous studies do not give a clear picture of how gait in DCD is different from that seen in typical development. In ageing research, measures of velocity and acceleration patterns of the centre of mass (CoM), as well as the variability of these measures have been used as an indicator of stability when walking. These measures might be useful in understanding the poor stability (e.g. tripping and bumping into things) reported in DCD. The aim of this study was therefore to examine the developmental pathway of foot placement and CoM velocity and acceleration, as well as the movement variability during level walking in children and adults with and without DCD.

**Method:** 15 primary school aged children (7-11years), 15 secondary school aged children (12-17years), and 15 adults (18-30years) were recruited in line with DSM-5 diagnostic criteria for DCD. Typically developing (TD) controls were individually matched for age and gender. Participants walked barefoot at a natural pace up and down an 11m walkway for one minute. Reflective markers placed on the sacrum (to mimic CoM) and feet were tracked with a VICON motion analysis system. Spatial and temporal characteristics of foot placement and velocity and acceleration of the CoM were calculated and recorded, as well as measures of movement variability.

**Results:** The DCD groups showed similar gait patterns to the TD groups in terms of foot placement measures, including step length, step width, double support time and stride time. The DCD groups also showed similar CoM velocity and acceleration to their peers. However, the DCD groups exhibited greater variability in some of the measures.

**Discussion:** The finding that individuals with DCD have a reduced ability to produce consistent movement patterns will be discussed in relation to postural control limitations and compared to variability of walking measures, which have been found to underlie the increased incidence of falling in elderly populations. Effects of age will also be discussed.

**Keywords:** Gait; Locomotion; Movement variability; Acceleration.

### Sensory processing difficulties among children with DCD

B. Engel-Yeger

Department of Occupational Therapy, University of Haifa, Haifa, 3498838, Israel. bengel@univ.haifa.ac.il

**Aims:** (1) To profile sensory processing difficulties (SPD) among children with DCD (2) To compare their prevalence to those of children with typical development and (3) To examine the relationship between SPD severity, DCD severity and socio-demographic parameters.

**Method:** The study group included 38 children diagnosed with DCD (by their pediatrician and by their score in the Movement Assessment Battery for Children (MABC) (Henderson & Sugden, 1992) (below the 15th percentile). The control group included 53 children with typical development. Children in this group scored above the 15th percentile on the MABC. The parents of all children completed a health status/socio-demographic questionnaire and the Short Sensory Profile (SSP) which evaluates the child’s sensory processing abilities as expressed in daily life in regard to all sensory modalities. The SSP has good internal consistency: α=.70-.90 and discriminant validity of >95% in identifying children with and without SPD (McIntosh et al., 1999).

**Results:** SPD were significantly more prevalent among children with DCD with no relation to DCD severity level. This was mainly expressed in greater auditory and visual sensitivity (χ²_2_=8.38, p=.02); in lower energy level (χ²_2_=16.71, p≤.001); and in seeking for sensations (χ²_2_=6.75, p=.03). In the study group SPD was not correlated with child’s age or with familial socio-economic level. However, a significant correlation was found between SPD and mothers’ education.

**Discussion:** SPD may be more prevalent among children with DCD. The expression of SPD in specific modalities and behaviors such as greater sensitivity and lower energy level may partially explain motor difficulties in this population. SPD should be screened among children with DCD and when diagnosed, should be included in the intervention program in order to focus intervention on child’s specific needs. The intervention process should elevate child and parents’ awareness to the possible involvement of SPD in child’s motor abilities and in daily life. This attitude may enhance child’s performance and participation and elevate child’s well being.

**References**:

Henderson, S. E., & Sugden, D. A. (1992). *The movement assessment battery for children*. San Antonio, TX: The Psychological Corporation.

McIntosh, D.N., Miller, L.J., & Shyu, V. (1999). Development and validation of the Short Sensory Profile. In W. Dunn (Ed.), *Sensory Profile manual* (pp. 59-73). San Antonio, TX: Psychological Corporation.

**Keywords:** DCD; Children; Sensory Processing Disorders.

### Exploring the internal modelling deficit hypothesis in DCD

G.D Ferguson^**1**^ & B.C.M Smits-Engelsman^**1,2**^

^1^Department of Health and Rehabilitation Sciences, University of Cape Town, Cape Town 8000, South Africa. Gillian.Ferguson@uct.ac.za; ^2^Department of Kinesiology, Katholieke Universiteit Leuven, Heverlee, Belgium.

**Aim:** To determine whether South African children with Developmental Coordination Disorder (DCD) perform differently to typically developing (TD) children when assessed on paradigms that are presumed to tap into internal models of motor control.

**Method:** In the first study, we compared the performance of TD (n=30) and children with DCD (n=30) on a manual task executed on a digitizing tablet (age range 6-10 years). In the second study, we compared the performance of 40 children with DCD and 40 TD children (age range 6-8 years) who were required to track a target moving along a circular path presented on a monitor by moving an electronic pen on a digitizing tablet. The task was performed under two visibility conditions (target visible throughout the trajectory and target intermittently occluded) and at two different target velocities (30° and 60° per second).

**Results:** In the first study, we show that the TD group had fewer velocity changes during the movement trajectory compared with the DCD group. The DCD group was less accurate and ended a further distance from the targets compared to the TD group who made fewer endpoint errors. Amplitude error was significantly greater for the DCD group as was directional error. In the second study, results showed that children with DCD were less proficient in tracking a moving target. Their performance deteriorated even more when the target was occluded and when the target speed increased. The mean tracking speed of the DCD group exceeded the speed at which the target rotated.

**Discussion:** The perception, interpretation and use of visual cues are essential elements that guide our behaviours in daily life, and any maladaptive decision or choice made in a specific situation might have serious consequences. Understanding how specific task constraints affect the way tasks are executed should be an important consideration when developing more effective and targeted intervention strategies. The kinematic profiles on the digitizing tablet show that children with DCD make more corrective movements when aiming at an array of distinct targets. We hypothesize that the presence of increased neuromotor noise is likely to constrain the ability to build accurate internal (forward) models. The poor tracking performance of the DCD group adds evidence of impairments in predictive control and poor dynamic internal representation of target or hand motion.

**References**:

Ferguson, G. D., Duysens, J., & Smits-Engelsman, B. C. M. (2015). Children with Developmental Coordination Disorder are deficient in a visuo-manual tracking task requiring predictive control. *Neuroscience, 286*(0), 13-26.

**Keywords:** Mental chronometry; Visuo-manual tracking; Pursuit; Predictive control.

### Fronto-parietal differences in brain activations of children with DCD in a calculation and a saccades task: an fMRI study

A. Gomez^**1,2**^, A. Jobert^**1**^, G. Dehaene-Lambertz^**1**^, S. Dehaene^**1**^ & C. Huron^**1**^

^1^Cognitive Neuroimaging Unit, INSERM, U992, CEA/SAC/DSV/DRM/NeuroSpin, 91100, France. alice.gomez@isc.cnrs.fr; ^2^Center for Cognitive Neuroscience (CNC), UMR 5229, Université Claude Bernard Lyon 1 (UCBL), Ecole Supérieure du Professorat et de l’Enseignement (ESPE), Lyon, France.

**Aim:** The neural correlates of Developmental Coordination Disorder (DCD) remain understudied^1^. DCD does not only affect the coordination of movements, it also impairs other cognitive abilities, crucial for academic achievement, such as mathematical learning. Given the hypothesis of a common parietal mechanism underlying calculation and eye-movements^2^, we hypothesized that a parietal dysfunction could play a role into mathematical and saccadic impairments reported in children with DCD.

**Method:** To assess this hypothesis, 17 8-9 years-old children with DCD and 15 typically developping (TD) children, performed an approximate calculation and a saccade task during a 3T fMRI scanning. Main contrasts of interests, conjunction analysis across groups, inter-group analyses (p<0.001, k=5) and Region Of Interest (ROI) analyses (based on developmental studies of numerical cognition^3^) were performed to identify specific and common activity in both groups.

**Results:** In the saccade task, the group conjunction analysis showed a classical fronto-parietal network in the saccades vs. central fixation task contrast. However, the left superior parietal lobule (BA 7), middle frontal gyrus (BA 6), precentral gyrus (BA 4), and thalamus were significantly more activated in TD children than in children with DCD. In the calculation task, the group conjunction analysis showed a common activation of the right inferior frontal gyrus (BA 47). Moreover, the right middle frontal gyrus (BA 9) was significantly more activated in children with DCD than in TD children. In contrast, ROI analysis showed higher activations of the left inferior parietal lobule (BA 40) in TD children than in children with DCD.

**Discussion:** Our results suggest parietal dysfunctions in DCD.

**References**:

1. Peters, L., Maathuis, C. G. B., & Hadders-Algra, M. (2013). Neural correlates of developmental coordination disorder. *Developmental Medicine and Child Neurology*, *55*(4), 59–64. doi:10.1111/dmcn.12309

2. Knops, A., Thirion, B., Hubbard, E. E. M., Michel, V., & Dehaene, S. (2009). Recruitment of an area involved in eye movements during mental arithmetic. *Science*, *324*(5934), 1583–5. doi:10.1126/science.1171599

3. Kaufmann, L., Wood, G., Rubinstein, O., & Henik, A. (2011). Meta-analyses of developmental fMRI studies investigating typical and atypical trajectories of number processing and calculation. *Developmental Neuropsychology*, *36*(6), 763–87.

**Keywords:** fMRI; Parietal; Frontal; Cognition; Saccades; Mathematic; Numerical; DCD.

### Maternal preeclampsia negatively effects long term motor development

T. Grace^**1**^, B. Hands^**2**^, M. Bulsara^**2**^ & C. Pennell^**3**^

^1^Institute for Health Research, The University of Notre Dame Australia, Fremantle, 6959, Australia. tegan.grace1@my.nd.edu.au; ^2^Institute for Health Research, The University of Notre Dame Australia, Fremantle, Australia; ^3^School of Women’s and Infants’ Health, The University of Western Australia, Australia.

**Aim:** To determine if maternal hypertensive diseases during pregnancy are a risk factor for compromised motor development at 10, 14, and 17 years.

**Method:** Offspring (N=2868) from the Raine Study (1) were classified by their mothers pregnancy blood pressure profile: normotension (n=2133), hypertension (n=626) and preeclampsia (n=109). Offspring motor development, at 10 (n=1622), 14 (n=1584), and 17 (n=1221) years were measured by the Neuromuscular Developmental Index (NDI) (M=100, SD=15) of the McCarron Assessment of Motor Development (MAND) (2). Linear mixed models, controlling for sociodemographic and obstetric risk factors, were used to compare outcomes between pregnancy groups. Doppler waveform data were examined to explore the theory of restricted placental blood flow as a potential mechanism (3).

**Results:** Offspring from pregnancies with preeclampsia had poorer motor outcomes at all ages than offspring from mothers with normotension (p ≤ 0.001) or hypertension (p = 0.002). The preeclampsia group contained a higher percentage of individuals (46.8%) who fell below the cutoff (≤85) used to determine motor disability compared to the hypertension (27.9%) and normotension (24.6%) groups (p = <0.001). Mothers with preeclampsia and restricted placental blood flow were more likely to be diagnosed with early onset preeclampsia, considered by some researchers as a more severe type of preeclampsia with greater associated health risks (4,5).

**Discussion:** Preeclampsia, especially early onset preeclampsia with associated placental blood flow restrictions was related to lower motor outcomes. Abnormal placental morphology may interrupt the development of neurological pathways, resulting in a long term deficit of motor development.

**References**:

1. Newnham, J. P., Evans, S. F., Michael, C. A., Stanley, F. J., & Landau, L. I. (1993). Effects of frequent ultrasound during pregnancy: A randomised controlled trial. *Lancet, 342*(8876), 887-891.

2. McCarron, L. T. (1997). *MAND McCarron Assessment of Neuromuscular Development.* Dallas, TX: Common Market Press.

3. Matsuo, K., Malinow, A. M., Harman, C. R., Baschat, A. A. (2009). Decreased placental oxygenation capacity in preeclampsia: Clinical application of a novel index of placental function performed at the time of delivery. *Journal of Perinatal Medicine, 37*(6), 657-661.

4. Leeson, P. (2013). Invited Plenary Orals: Long term cardiovascular outcomes for mother and child. *Pregnancy Hypertension: An International Journal of Women’s Cardiovascular Health, 3*, 57-61.

5. Egbor, M., Ansari, T., Morris, N., Green, C. J., Sibbons, P. D. (2006). Morphometric placental villous and vascular abnormalities in early-and late-onset pre-eclampsia with and without fetal growth restriction. *British Journal of Obstetrics and Gynaecology, 113*(5), 580-589.

**Keywords:** Preeclampsia; Pregnancy; Motor development; Raine Study; Adolescence.

### Speech and motor lateralisation in adults with Developmental Coordination Disorder: a functional Transcranial Doppler imaging study

J.C. Hodgson & J.M. Hudson

School of Psychology, University of Lincoln, Brayford Pool, Lincoln, LN6 7TS, UK. jhodgson@lincoln.ac.uk

**Aim:** Research using clinical populations to explore the relationship between hemispheric speech lateralisation and handedness has focussed on individuals with language disorders, such as dyslexia or specific language impairment (SLI). Results from such work reveal atypical patterns of cerebral lateralisation and handedness in these groups compared to controls. There are few studies that examine this relationship in people with motor coordination impairments but without speech or reading deficits, which is a surprising omission given the prevalence of theories suggesting a common neural network underlying both functions. This study fills that gap by using an emerging imaging technique in cognitive neuroscience; functional Transcranial Doppler (fTCD) sonography, and an electronic peg moving task, to assess the relationship between speech lateralisation and motor control in participants with Developmental Coordination Disorder (DCD).

**Method:** 12 adult control participants (5 males; aged 18-28 yrs, *mean* = *20yrs*) and 12 adults with DCD and no other developmental/cognitive impairments (4 males; aged 18-43 yrs, *mean* = *25.3yrs*), performed a word generation task whilst undergoing fTCD imaging to establish a hemispheric lateralisation index for speech production. DCD status was confirmed via the Adult Developmental Co-ordination Disorders /Dyspraxia Checklist (ADC) (Kirby, Edwards, Sugden & Rosenblum, 2010). All participants completed a handedness questionnaire, an electronic peg moving task to determine hand skill, a shortened version of the Raven’s Matrices non-verbal reasoning test and the phonological processing section of the York Adult Assessment Battery (YAA-R).

**Results:** As predicted the DCD group showed a significantly reduced left lateralisation pattern for the speech production task compared to controls (mean laterality indices = 1.89 and 3.77 respectively, *t*(22) = -2.2, *p* < .05). Results from the Ravens Matrices test were equivalent across groups (*t*(22) = .008, *p* = .993) and the groups did not differ significantly on the handedness classification. Performance on the motor skill task showed a clear preference for the dominant hand across both groups (*t*(23) = -4.472, *p* < .001), however the DCD group showed significantly slower mean movement times for the non-dominant hand compared to controls (*t*(22) = 2.270, *p* < .05).

**Discussion:** This is the first study of its kind to assess hand skill and speech lateralisation in individuals with DCD. The results reveal a reduced leftwards asymmetry for speech and a slower motor performance specific to the non-dominant hand. This fits alongside previous work showing atypical cerebral lateralisation in DCD for other cognitive processes (e.g. executive function and short term memory). The findings are clinically relevant for understanding the profile of handedness in DCD and speak to debates on theories of hemispheric specialisation and language lateralisation.

**References**:

Kirby, A., Edwards, L, Sugden, D. & Rosenblum, S. (2010). The development and standardization of the Adult Developmental Co-ordination Disorders/Dyspraxia Checklist (ADC). *Research in Developmental Disabilities, 31*, 131–139. doi:10.1016/j.ridd.2009.08.010

**Keywords:** DCD; Speech lateralisation; Functional transcranial Doppler (fTCD); Motor skill; Handedness.

### Neural mechanisms supporting observational motor learning in children with Developmental Coordination Disorder - An EEG study

J. Lust^**1**^, H. van Schie^**1**^, J. van der Helden^**2**^, P. Wilson^**3**^ & B. Steenbergen^**1,3**^

^1^Behavioural Science Institute, Radboud University, Nijmegen, PO Box 9104, The Netherlands. j.lust@pwo.ru.nl; ^2^University of applied sciences Saxion, Enschede, The Netherlands; ^3^School of Psychology, Australian Catholic University, Melbourne, Australia.

**Aim:** Children with Developmental Coordination Disorder (DCD) experience problems in the learning and execution of motor actions. Learning a new movement by action observation is very important in for example classroom settings and entails the transposition of the observed action to the existing internal motor representations of the observer. The automatic activation of motor representations during action observation, as well as the functional coupling between perception and action have been hypothesized as important neural processes supporting observational action learning. In the present study we aim to study these brain mechanisms of observational motor learning in children with and without DCD.

**Method:** Motor neural network activation will be measured by electroencephalography (EEG), specifically event-related desynchronization (ERD) of mu rhythms and fronto-parietal coherence. We will include 15 children with DCD and 15 matched controls in two different tasks conditions: action observation to learn (imitate the action afterwards) and action observation to detect (report a deviant movement afterwards). Children with DCD are expected to imitate the action with a lower level of accuracy than controls. As mu-suppression has been suggested as a measure of the activation of internal motor representations, children with DCD are expected to show less mu-suppression compared to the controls. Moreover, as individual differences in fronto-parietal coherence have been found to be positively related to imitation accuracy, children with DCD are expected to show decreased coherence during the observation to learn condition.

**Results:** Results from adult participants without motor problems performing this task, reported by co-authors HvS and JvdH^1^, have shown that mu-suppression is maximal during pause intervals in the action observation condition, consistent with rehearsal of movement sequences using motor imagery. Importantly, individual differences in the strength of fronto-parietal coherence were found to predict imitation accuracy. Results in children with and without DCD will be presented on the conference.

**Discussion:** Findings will be discussed in the context of the internal modeling deficit hypothesis for children with DCD and will be related to existing knowledge from other developmental disorders associated with high levels of clumsiness such as autism spectrum disorder (ASD).

**References**:

1. Van der Helden, J., Van Schie, H. T., & Rombouts, C. (2010). Observational learning of new movement sequences is reflected in fronto-parietal coherence. *PLoS ONE, 5*(12): e14482. doi: 10.1371/journal.pone.0014482

**Keywords:** Observational learning; Motor resonance; Fronto-parietal coherence; Developmental Coordination Disorder (DCD); Internal modeling deficit hypothesis (IMD).

### Gait pattern under the different availability of visual information in children at a risk of DCD

R. Psotta, M. Palomo-Nieto & R. Abdollahipour

Department of Natural Sciences in Kinanthropology, Palacky University, Olomouc, 771 11, Czech Republic. rudolf.psotta@upol.cz

**Aim:** The aim of the study was to investigate how the availability of visual information sources can affect the motor control of walking in children at risk of DCD.

**Methods:** Sixteen children at risk of DCD (DCDR) (8.7 ± 0.8 years) indicated by the total test score TTS ≤ 15th percentile in the MABC-2 test, and sixteen typically developing children (TD) (9.1 ± 1.0 years) with TTS ≥ 16th percentile participated in the experiment. Each child walked along a 10m walkway under four vision conditions: full vision (FV), non-vision (NV), and limited vision consisting of 2000 ms intervals of vision interrupted by 150 ms and 100 ms of non-vision (LV150 and LV100), with the use of Plato portable liquid-crystal goggles. While walking, the Optojump Next Microgate device was used to measure the kinematic measurements of the gait cycle.

**Results:** Two-way ANOVA 2 (TD vs. DCDR) x 4 (FV, LV150, LV100 & NV) (α = 0.05) carried out for each variable of a gait cycle revealed that the DCDR children manifested a significantly shorter stride length (cm), shorter single support time and stance time (s), longer pre-swing time (s; %) and load response time (s; %), and lower speed of stride scaled to functional leg length (FLL) as compared to the TD children. In addition, the stride length (cm), single support time (s; %), stance time (s; %) and speed of stride/FLL were significantly affected by visual conditions.

**Discussion:** The results provided evidence that children at risk of DCD produce the slower walk with shorter steps than their TD peers. Differences in the gait cycle between these groups were higher when performed with more limited visual information. The study has provided support for the notion that children with DCD rely more on visual information than children without a deficit in motor coordination.

**References**:

Deconinck, F. J. A., DeClerq, D., Savelsbergh, G. J. P., VanCoster, R., Oostra, A., Dewitte, G., & Lenoir, M. (2006). Differences in gait between children with and without developmental coordination disorder. *Motor Control, 10,* 125-142.

Henderson, S. E., Sugden, D. A., & Barnett, A. L. (2007). *Movement Assessment Battery for Children-2.* London: Harcourt Assessment.

Williams H. (2002). G. Motor control in children with developmental coordination disorder. In S. A. Cermak & D. Larkin (Eds). *Developmental Coordination Disorder* (pp. 117-137). Albany, NY: Thomson Learning.

**Keywords:** Developmental coordination disorder; Motor control; Walk; Vision; Children.

### Do children with DCD accurately predict their action gaps in a road crossing situation?

C. Purcell^**1**^, K. Wilmut^**2**^ & J. Wann^**3**^

^1^The Dyscovery Centre, University of South Wales, Felthorpe House, Lodge Road, Newport NP18 3QT, UK. catherine.purcell@southwales.ac.uk; ^2^Department of Psychology, Oxford Brookes University, Oxford, UK; ^3^Department of Psychology, Royal Hollway, University of London, Egham, Surrey, UK.

**Aim:** Previous research has demonstrated that children with Developmental Coordination Disorder (DCD) have reduced perceptual sensitivity in their ability to detect approaching vehicles (Purcell *et al*., 2012) and are unable to accurately discriminate between the approach speeds of two vehicles (Purcell *et al*., 2011). However, the ability to safely cross a road is a perceptual-motor skill which involves the coordination of a pedestrian’s perception of the time available and their locomotive capability. The road crossing task therefore requires the observer to ensure that the size of the gap is related to the time needed to cross safely. The aim of this study was to systematically measure the temporal gaps that children with DCD accept in the context of road crossing.

**Method:** The temporal crossing gaps that 11 children with DCD aged 6-11 years and 11 age and gender matched typically developing children accepted was measured, using a psychophysical procedure in a virtual road crossing environment, where multiple vehicles, in three speed conditions, approached from only the near-side lane or bi-directionally. In addition, to assess the sufficiency of their temporal gaps, the difference between gap acceptance thresholds and their crossing time was calculated.

**Results:** In line with previous research (e.g. Connelly *et al.*, 1998), the temporal gaps accepted within each group decreased as approach speed increased. However, unlike their typically developing peers, for vehicles approaching at speeds greater than 20mph, children with DCD accepted temporal gaps that were significantly insufficient and would have resulted in collision.

**Discussion:** These findings suggest that the temporal gaps that children with DCD accept would result in collision if translated to the roadside. These findings will be discussed in relation experience at the roadside.

**References**:

Connelly, M. L., Conaglen, H. M., Parsonson, B. S., & Isler, R. B. (1998). Child pedestrians’ crossing gap thresholds.* Accident Analysis & Prevention, 30*(4), 443-453. doi:10.1016/S0001-4575(97)00109-7.

Purcell, C., Wann, J. P., Wilmut, K., & Poulter, D. (2011). Roadside judgments in children with developmental co-ordination disorder. *Research in Developmental Disabilities*, doi:10.1016/j.ridd.2010.12.022.

Purcell, C., Wann, J. P., Wilmut, K., & Poulter, D. (2012). Reduced looming sensitivity in primary school children with developmental co-ordination disorder.* Developmental Science, 15*(3), 299-306. doi:10.1111/j.1467-7687.2011.01123.x.l

**Keywords:** Developmental Coordination Disorder; Road crossing; Temporal gaps; Margin for error.

### Perceptual and sensorimotor timing in children with Attention Deficit Hyperactivity Disorder with or without Developmental Coordination Disorder

F. Puyjarinet^**1**^, J.-M. Albaret^**2**^ & S. Dalla Bella^**1,3,4**^

^1^Movement to Health Laboratory (M2H), EuroMov, Université de Montpellier, 34000 Montpellier, France. f.puyjarinet@hotmail.fr; ^2^PRISSMH-EA 4561, Université de Toulouse III, UPS, Toulouse, France; ^3^Institut Universitaire de France, Paris, France; ^4^International Laboratory for Brain, Music, and Sound Research (BRAMS), Montreal, Canada.

**Aim:** There is a considerable body of studies showing that subjects with Attention Deficit Hyperactivity Disorder (ADHD) have deficits in a range of timing functions (Noreika, Falter, & Rubia, 2013). Nevertheless, little is known whether comorbidities influence those timing disorders. Fifty percent of children suffering from ADHD show severe motor disabilities leading to a diagnosis of Developmental Coordination Disorder or DCD (Martin, Piek, & Hay, 2006). Timing deficits may also occur in certain subtypes of DCD population. Yet, it is unclear whether deficits are associated or not with motor factors. The aim of the present work is to examine perceptual and sensorimotor timing abilities in children with ADHD with and without associated DCD. We expect to find more severe deficits in sensorimotor timing tasks DCD+ADHD as they involve motor performance. However, differences are not expected in purely perceptual timing tasks.

**Method:** 23 DCD+ADHD children, 20 ADHD children, and 14 healthy age-matched controls, all from 7 to 14 years of age, took part in the experiment. They were submitted to the Battery for the Assessment of Auditory Sensorimotor and Timing Abilities (BAASTA, Benoît *et al*., 2014) which consists of both perceptual timing and sensorimotor timing tasks. Each measure was submitted to a one-way ANOVA, with a significance level set at p < .05. Then, post hoc analyses using Tukey tests were performed.

**Results:** Data analysis is currently in progress. Preliminary results suggest a greater impairment in timing abilities in the DCD+ADHD group as compared to ADHD and control groups, even when the tasks do not require a motor response. However, this difference is not visible with all timing measures. The ADHD group also tends to show worse timing capacities than the controls.

**Discussion:** The goal of this study was to examine the potential effect of DCD+ADHD condition on timing abilities. Our results will pave the ground to remediation methods aimed to improve perceptual, motor and sensorimotor abilities among children with neurodevelopmental disorders.

**References**:

Benoit, C. E., Dalla Bella, S., Farrugia, N., Obrig, H., Mainka, S., & Kotz, S.A. (2014). Musically cued gait-training improves both perceptual and motor timing in Parkinson’s disease. *Frontiers in Movement Neuroscience, 8*, 494.

Martin, N., Piek, J. P., & Hay, D. (2006). DCD and ADHD: A genetic study of their shared aetiology. *Human Movement Science, 25*, 110-124.

Noreika, V., Falter, C.M., & Rubia, K. (2013). Timing deficits in attention-deficit /hyperactivity disorder (ADHD): Evidence from neurocognitive and neuroimaging studies*. Neuropsychologia, 51*, 235-266.

**Keywords:** Timing abilities; DCD; ADHD; Comorbidities.

### Mirror neuron activation in children with probable developmental coordination disorder: a functional MRI study

J. Reynolds^**1**^, M. Licari^**1**^, J. Billington^**2**^, J. Williams^**3**^, C. Elliott^**4**^, B. Lay^**1**^, A.M. Winsor^**5**^, L. Codd^**5**^ & M. Bynevelt^**5**^

^1^School of Sport Science, Exercise and Health, The University of Western Australia, Perth, 6009, Australia. jessica.reynolds@research.uwa.edu.au; ^2^Institute of Psychological Sciences, University of Leeds, Leeds, United Kingdom; ^3^College of Sport and Exercise Science, Victoria University, Melbourne, Australia; ^4^Faculty of Health Sciences, Curtin University, Perth, Australia; ^5^Neurological Intervention and Imaging Service of Western Australia, Sir Charles Gairdner Hospital, Perth, Australia; ^6^Department of Diagnostic Imaging, Princess Margaret Hospital for Children, Perth, Australia.

**Aim:** It has recently been hypothesized that a deficit in the functioning of the mirror neuron system (MNS) may contribute to the motor impairments associated with DCD. The aim of this study was to reveal cortical areas that may contribute to the movement difficulties seen in children with probable DCD (pDCD), and to examine suspected deficits in the MNS using functional magnetic resonance imaging (fMRI).

**Method:** Twenty-four right handed boys participated in this study, 12 with pDCD (mean age = 10.06yrs ± 1.47) and 12 typically developing controls (mean age = 10.23yrs ± 0.98). Prior to scanning, children were tested on the MABC-2, with children in the pDCD group ≤16th percentile and controls ≥20th percentile. MR imaging was conducted on a 3T Philips Achieva TX scanner using a 12 channel head coil. High resolution anatomical images were acquired first (T1-weighted 3D FFE 160 slices 1x1x1 mm), followed by two functional studies (T2-weighted gradient echo, TR/TE = 3000/35ms, flip angle 90°, 25 axial slices with a thickness of 4mm, no gaps). A randomized block design was used for functional runs, during which a right index finger adduction/abduction tapping task was performed under four MNS activation conditions: observation, execution, imagery and imitation. Visual and auditory stimuli were used to prompt and coordinate each task. Active tasks (18secs) were interspersed with rest periods (12secs). Cortical activations of mirror neuron regions, including posterior inferior frontal gyrus (IFG), ventral premotor cortex, anterior inferior parietal lobule and superior temporal sulcus were examined. Preprocessing and analysis was performed using SPM12.

**Results:** Full results will be presented at DCD-11. Preliminary results indicate children with pDCD have decreased cortical activation in MNS related regions, including the precentral gyrus and IFG, as well as in the posterior cingulate and precuneus (PCC/Pcu) complex.

**Discussion:** Initial evidence supports a deficit in the MNS in children with pDCD. Due to its key role in imitation, motor imagery, and visual learning, dysfunction of the MNS is likely to result in a reduced ability to develop motor programs, impacting functionally on activities of daily living, sport participation and social situations. A greater understanding of MNS function in children with DCD has the potential to inform and tailor intervention programs for this population.

**Keywords:** Mirror Neuron System (MNS); Functional Magnetic Resonance Imaging (fMRI); Cortical Function; Imitation; Motor Imagery.

### Do they ‘look’ the same? Comparing gaze patterns in school-aged children with and without developmental coordination disorder during a visual-motor task

L.M. Rivard^**1**^, T.D. Lee^**2**^, L.R. Wishart^**3**^ & C. Missiuna^**4**^

^1^School of Rehabilitation Science and CanChild Centre for Childhood Disability Research, McMaster University, 1400 Main St. W., Hamilton, Ontario L8S 1C7, Canada. lrivard@mcmaster.ca; ^2^Department of Kinesiology, McMaster University, Canada; ^3^School of Rehabilitation Science, McMaster University, Canada; ^4^School of Rehabilitation Science and CanChild Centre for Childhood Disability Research, McMaster University, Canada.

**Aim:** To compare gaze patterns in children with developmental coordination disorder (DCD) and typically developing (TD) children using an eye tracker during a fine motor pouring task performed in a natural setting.

**Method:** Using a mobile eye-tracker and gaze analysis paradigm, we compared visual fixation patterns in children with DCD and their TD peers during a serial motor task. Wearing eye tracking glasses, children poured water from each of 3 ‘pouring’ cups into 3 colour-matched ‘filling’ cups in sequence. All participants completed 12 trials; the order/position of the filling cups was altered on every 3rd trial (pouring cups stayed in the same position). Gaze overlay videos were extracted. An eye-tracking video observation coding scheme was developed and intra-rater reliability determined. First-person perspective videos (with a forward-facing view of the task scene) with superimposed gaze provided measures of: 1) total fixation time on a) pouring cups, b) filling cups, and c) dominant upper extremity; 2) proportion of total time spent visually fixating on ‘objects of importance’ (pouring and filling cups); and 3) average fixation time on pouring and filling cups, either before or during 3 functional task phases (forward transport, pour, back transport/set down/release).

**Results:** 12 TD children (10M, 2F), mean age 9.7 years, and 12 children with DCD (11M, 1F), mean age 10.5 years, participated. Children with DCD visually fixated for significantly more time on their dominant upper extremity than TD children (p<.05, ES=0.5). TD children visually fixated for a significantly longer proportion of total time on pouring/filling cups than children with DCD (p<.05, ES=0.4). Children with DCD fixated for a significantly longer average duration time on the pouring cups during the pour phase (p<.05, ES=0.6). A similar trend was noted for visual fixations on pouring cups during forward transport but was non-significant.

**Discussion:** Previously, children with DCD have been noted to depend on vision during motor performance, suggesting poorly developed or integrated internal models of movement control. Our results suggest that children with DCD may use vision specifically to monitor their body position rather than other important aspects of a motor task. In addition, they do not appear to use predictive ‘look-ahead’ visual fixations, with a need to visually monitor their motor movements throughout a motor task, supporting the notion of an internal-model deficit.

**Keywords:** Eye-tracking; Vision; Gaze analysis; Prediction; Internal models.

### Cognitive-motor interference during walking in children with Developmental Coordination Disorder

N. Schott & I. El-Rajab

Institute for Sport and Exercise Science, University of Stuttgart, Germany. nadja.schott@inspo.uni-stuttgart.de

**Aim:** While typically developing children produce relatively automatized postural control processes, children with DCD exhibit an automatization deficit (Tsai *et al*., 2009). Dual tasks with various cognitive loads seem to be an effective way to assess the automatic deficit hypothesis. The aims of the study were to (1) examine the effect of a concurrent task on walking in children with DCD; and (2) to evaluate if easy cognitive tasks can lead to performance improvements in the motor domain.

**Method:** We examined dual-task performance of a cognitive and a sensorimotor task (walking) in 20 children with DCD (boys, n = 12; girls, n = 8; mean age, 8.10 ± 1.07 years) and 39 typically developing children (boys, n = 18; girls, n = 21; mean age, 8.44 ± 1.19 years). Based on the idea of the paper-and-pencil Trail Making Test, participants walked along a fixed pathway (TWT-A), stepped on targets with sequentially increasing numbers (i.e., 1-2-3; TWT-B), and sequentially increasing numbers and letters (i.e., 1-A-2-B-3-C; TWT-C; Schott, in press). The dual-task costs (DTC) were calculated for each task. Additionally the following items were assessed: Movement Assessment Battery for Children, test and checklist (MABC-2), and the Trail-Making Test (TMT).

**Results:** In the primary *walking* task condition (trail tracing), the differences were not statistically significant (p = 0.215) between children with and without DCD; however, we found significant differences for the *seated condition* of the trail tracing task (TMT 1; p=.003). A concurrent cognitive task increased times significantly in all three groups, with the effect greater in children with DCD. Increased cognitive task complexity resulted in greater slowing of gait: gaits DTCs as well as handwriting DTCs were least for the simplest conditions and greatest for the complex conditions in children with DCD more so than in comparison children. Additionally, gait DTCs were significantly lower than handwriting DTCs.

**Discussion:** These results support previous studies suggesting that children with DCD are more cognitively dependent and may have an automatization deficit, especially in the fine motor control task.

**References:**

Schott, N. (in press). The Trail-Walking-Test (TWT-D): Development and Evaluation of the psychometric properties of the assessment of the motor-cognitive interference in older adults. *Zeitschrift fuer Gerontologie und Geriatrie.*

Tsai, C. L., Pan, C. Y., Cherng, R. J., & Wu, S. K. (2009). Dual-task study of cognitive and postural interference: a preliminary investigation of the automatization deficit hypothesis of developmental co-ordination disorder. *Child Care Health Development, 35,* 551–60.

**Keywords:** Dual task costs; Trail-Walking-Test; Walking; Developmental Coordination Disorder; Children.

### Does viewing an object elicit internal motor programs for children with Developmental Coordination Disorder?

E. Sumner, H.C. Leonard & E.L. Hill

Department of Psychology, Goldsmiths, University of London, New Cross, London, SE14 6NW, United Kingdom. e.sumner@gold.ac.uk

**Aim:** Affordance theory takes the view that objects are perceived not only in terms of their size and shape, but also in relation to the possibilities for action. Research has shown that adults activate internal representations related to the execution of power or precision grips when viewing compatible objects. The aim of this study was to explore whether children with and without Developmental Coordination Disorder (DCD) elicit motor programs that support possible actions towards an object in their visual field.

**Method:** Data collection is ongoing at the time of writing. Thirty children, aged 7-10 years, with a diagnosis of DCD (DSM-5) are being recruited alongside 30 typically developing age-matched children. In one hand children hold a cylinder device that they press using a power grip, and in the other hand is a small button mimicing a precision grip. Children view individually photographed objects (8 neutral object, 8 affording power grips, 8 affording a precision grip) on a laptop and are told the aim of the task is to respond quickly when they see a colour appear over the object; using the two devices, pressing one device if orange and the other if purple. Colour allocation is counterbalanced and each object is shown four times, resulting in two object grip/colour compatible, and two incompatible possibilities. Children responded to colour rather than the object because a range of objects were presented that did not have one obvious theme, aside from their associated grip. Children were told to stay focused on the objects as they would be asked questions about them on completion of the task. Halfway through the experiment, children swap the devices to the other hand to control for hand preference.

**Results:** Preliminary analyses of 16 participants in each group showed that children with DCD made a higher number of errors and had slower reaction times than the control group. Children were able to successfully identify the objects they saw. A compatibility effect was evident for both children with DCD and the control group when viewing objects that afford a power grip, meaning they were quicker to respond when objects matched the colour and device grip they had been assigned. However, this compatibility effect was only evident for the control group on the precision grip objects, and not for children with DCD. A significant negative correlation (*r* = -.46, *p* < .001) was found between manual dexterity performance and precision compatible reaction times.

**Discussion:** Initial findings show an interaction between type of response and object for both groups, when the object afforded a power grip. These reaction time findings imply that a motor plan is generated after object presentation. Reasons for a lack of a compatibility effect for the precision grip in the DCD group may be attributed to weaker precision (manual dexterity) skills in this group.

**References**:

American Psychiatric Association. (2013). *Diagnostic and statistical manual of mental disorders* (DSM, 5^th^ ed.). Arlington, VA: American Psychiatric Association.

**Keywords:** Affordances; Developmental Coordination Disorder; Manual dexterity; Power grip; Precision grip.

### Early motor assessment of very preterm infants is predictive of Developmental Coordination Disorder at 4.5 years

J.G. Zwicker^**1,2,3,4**^, M. Mackay^**5**^, J. Shen^**2,3**^, R. Brant^**3,6**^, S.P. Miller^**3,7**^, R.E. Grunau^**2,3,5**^ & A. Synnes^**2,3,5**^

^1^Department of Occupational Science and Occupational Therapy, University of British Columbia, Vancouver, Canada. jill.zwicker@ubc.ca; ^2^Department of Pediatrics, University of British Columbia, Vancouver, Canada; ^3^Child and Family Research Institute, Vancouver, Canada; ^4^Sunny Hill Health Centre for Children, Vancouver, Canada; ^5^British Columbia Women’s Hospital, Vancouver, Canada; ^6^Department of Statistics, University of British Columbia, Vancouver, Canada; ^7^Hospital for Sick Children and Department of Pediatrics, University of Toronto, Toronto, Canada.

**Aim:** Very preterm infants are at high risk of developing developmental coordination disorder (DCD), which is often undiagnosed before school-age. Although early identification of DCD is recommended, the course of motor performance from infancy to early childhood and the presence of DCD at the age of school entry is largely unknown. The purpose of this study was to examine the relationship between motor assessments conducted early in life [4, 8, 18 months corrected age (CA) and 36 months] with motor outcomes of very preterm children identified with DCD at 4.5 years.

**Method:** In a retrospective cohort of 320 very preterm infants (median gestational age: 26 weeks; median birth weight: 788 grams), the Movement Assessment of Infants (4 and 8 months CA) and Peabody Developmental Motor Scales-2 (18 months CA, 36 months), and Movement Assessment Battery for Children 1 or 2 (4.5 years) were conducted. Children with cerebral palsy and global developmental delay were excluded. DCD was defined as MABC ≤ 5th percentile. Differences between children with and without DCD were determined using Fisher’s exact test for categorical variables and the Mann-Whitney U test for continuous variables. Step-wise logistic regression and receiver operating curves used to determine if serial motor assessments were predictive of DCD at 4.5 years, adjusting for sex, birth weight, and days of ventilation.

**Results:** 85/320 (27%) of children were diagnosed with DCD. Children with DCD were primarily male, born earlier, had smaller birth weight, and scored more poorly on motor assessments at each time point compared to children without DCD (all p’s ≤ 0.001). MAI at 4 months significantly predicted DCD (OR 1.09, p=0.02; AUC=0.77), but not when MAI at 8 months was entered into model (8 month MAI: OR 1.13, p=0.001, AUC=0.81). Peabody total motor scores (TMQ) at 18 months CA (OR=0.89, p<0.001, AUC=0.81) and combined with 36 month scores (OR=0.88, p<0.001, AUC=0.84) also predicted DCD. The best predictive model included 8 month MAI and both TMQ scores, with the 8 month MAI (p=0.009) and 36 month TMQ (p=0.002) being significant (AUC=0.86).

**Discussion:** Pattern of motor performance on the MAI at 8 months and the TMQ at 36 months is predictive of DCD in very preterm children at 4.5 years. This is the first study to suggest that early motor performance of preterm infants is predictive of DCD and may allow for earlier identification of these high-risk infants.

**Keywords:** DCD; Prematurity; Early identification; Motor assessment.

## Oral Presentations

**Day 3: July 4, 2015**

### Effects of the Cognitive Orientation to Daily Occupational Performance (CO-OP) on Brazilian children with Developmental Coordination Disorder

C.R.S. Araujo^**1**^& L.C. Magalhães^**2**^

^1^Department of Occupational Therapy, Health Sciences Center, Federal University of Paraiba, Campus I, s/n - Castelo Branco, João Pessoa, PB, 58051-900, Brazil. clariceribeiro@hotmail.com; ^2^Department of Occupational Therapy, Federal University of Minas Gerais, Belo Horizonte, Brazil.

**Aim:** To explore the use of CO-OP protocol in treatment of Brazilian children with DCD; to investigate the effects of CO-OP in the performance of activities; to examine whether, after CO-OP, children were able to transfer and generalize the strategies and skills acquired to improve functional performance in other activities and contexts.

**Method:** Single subject designs with eight children with DCD, ages between six and ten years old, assessed with the Developmental Coordination Disorder Questionnaire (DCDQ-Brazil), the Child Behavior Checklist (CBCL), the Swanson, Nolan and Pelham Scale IV (SNAP IV), answered by their parents, the Movement Assessment Battery for Children-2 (MABC2), the Wechsler Intelligence Scale for Children-III (WISC-III) administered by external evaluators, the Perceived Efficacy and Goal Setting System (PEGS), the Canadian Occupational Performance Measure (COPM) and Performance Quality Rating Scale (PQRS), to identify goals and to measure changes in occupational performance. The children participated in 13 treatment sessions based on a CO-OP adapted protocol.

**Results:** Even though a number of children did not show progress in motor performance, as measured by MABC-2, occupational performance of all the children improved significantly, seven children were able to generalize learning and six children used the strategies and skills acquired in therapy to learn other tasks.

**Discussion:** There were significant differences on individual and group performance after CO-OP according to parents, children and external evaluators. There were also gains in performance of activities that were not trained in therapy, demonstrating the ability of some children to generalize and transfer the acquired skills to other tasks. Although the small sample, results were robust and consistent with previous studies, reaching statistical significance. Parents’ engagement in therapy is an important variable - children, whose parents were mostly engaged, achieved higher improvements in performance and satisfaction levels. Future studies should focus on parents training, a key component of CO-OP, and it is important to develop strategies to monitor objectively parents’ participation.

**References**:

American Psychiatry Association (2014). *Manual diagnóstico e estatístico de transtornos mentais: DSM-V*. 5 ed. Porto Alegre, RS: Artes Médicas.

Araújo, C. R. S., Magalhães, L. C., & Cardoso, A. A. (2011). Uso da *Cognitive Orientation to Daily Occupational Performance* (CO-OP) com crianças com transtorno do desenvolvimento da coordenação. *Revista Brasileira de Terapia Ocupacional da Universidade Federal de São Paulo, 22*(3), 245-253.

Henderson, S. E., Sugden, D. A., & Barnett, A. L. (2007). *The Movement Assessment Battery for Children*. *Second Edition*. London, UK: Harcourt Assessment.

Jarus, T., Lourie-Gelberg, Y., Engel-Yeger, B., & Bart, O. (2011). Participation patterns of school-aged children with and without DCD. *Research in Developmental Disabilities, 32*(4), 1323-1331.

Missiuna, C., Pollock, N., & Law, M. (2004). *Perceived Efficacy and Goal Setting System (PEGS)*. San Antonio, TX: Psychological Corporation.

Polatajko, H. J., & Mandich, A. (2004). *Enabling occupation in children: The Cognitive Orientation to Daily Occupational Performance (CO-OP) approach*. Ottawa, ON: CAOT Publications ACE.

**Keywords:** Motor skills disorders; Intervention studies; Activities of daily living; Occupational therapy.

### Effects of two distinct group motor skill interventions in psychological and motor skills of children with Developmental Coordination Disorder

P. Caçola, M. Romero, M. Ibana, & J. Chuang

Department of Kinesiology, The University of Texas at Arlington, Arlington, TX, 76019, USA. cacola@uta.edu

**Aim:** Children with Developmental Coordination Disorder (DCD) have an increased risk for mental health difficulties. The present study aimed to determine whether two distinct group intervention programs (in size and approach) for children with DCD improved several psychological variables (anxiety; adequacy and predilection for physical activity; participation, preferences, and enjoyment for activities) and motor skills from a child perspective as well as parental perceptions of motor skills, rate of function, and strengths and difficulties of their children.

**Method:** Both programs involved 10 sessions of one hour each. Eleven children participated in Program A and thirteen children participated in Program B. Program A focused on task-oriented activities in a large group (all participants) that involved motor skill training as well as collaboration and cooperation among children, and Program B was composed of three smaller groups with a direct goal-oriented approach for training of skills chosen by the children. Before and after each program, children were assessed with the Movement Assessment Battery for Children – 2nd ed. (MABC-2), Children’s Self-Perceptions of Adequacy in and Predilection for Physical Activity Scale (CSAPPA), Children’s Assessment of Participation and Enjoyment (CAPE) and Preferences for Activities of Children (PAC), and the Spence’s Child Anxiety Scale (SCAS), while parents answered the Developmental Coordination Disorder Questionnaire (DCD-Q), Strengths and Difficulties Questionnaire (SDQ), and the Children Activity Scale (CAS).

**Results:** Results indicated that children improved motor skills after both programs, but showed distinct results in regards to other variables – after Program A, children showed significantly higher anxiety and lower levels of enjoyment, even though parents detected an improvement in rate of function and a decrease in peer problems of their children. With Program B, children significantly decreased anxiety levels, and parents noted a higher control of movement of their children.

**Discussion:** We conclude that regardless of the group approach, children were able to improve their motor skills. However, it is possible that the group size and approach may influence parents’ perception of their children’s motor and psychological skills, as well as children’s perception of anxiety.

**Keywords:** Developmental Coordination Disorder; Group intervention; Motor skills; Psychological skills; Mental health

### The Coordination and Activity Tracking in Children (CATCH) Study: A new prospective cohort study to examine DCD and the activity-deficit in early childhood

J. Cairney^**1**^, C. Missiuna^**2**^, B.W. Timmons^**3**^, C. Rodriguez^**1**^, S. Veldhuizen^**1**^, S. King-Dowling^**1**^, S. Wellman^**1**^ & T. Le^**1**^

^1^Department of Family Medicine, McMaster University, Hamilton, L8P 0A1, Canada. cairnej@mcmaster.ca; ^2^School of Rehabilitation Science, McMaster University, Hamilton, Canada; ^3^Department of Pediatrics, McMaster University, Hamilton, Canada.

**Aim:** Past studies have found that children with Developmental Coordination Disorder (DCD) engage in less physical activity than typically developing children. This “activity deficit” may result in children with DCD being less physically fit and more likely to be overweight or obese, potentially increasing later risk for poor cardiovascular health. Unfortunately, the majority of DCD research has been limited to cross-sectional designs, leading to questions about the complex relationships among motor ability, inactivity and health-related fitness. Of the few longitudinal studies on the topic, determining precedence amongst these factors is difficult because study cohorts typically focus on mid to late childhood. By this age, both decreased physical fitness and obesity are often established. The purpose of the Coordination and Activity Tracking in CHildren (CATCH) study is to examine the pathways connecting DCD, physical activity, physical fitness, and body composition from early to middle childhood.

**Methods:** The CATCH study is a prospective cohort study. We aim to recruit a cohort of 600 children aged 4 to 5 years (300 children at risk for DCD (DCDr) and 300 controls) and test them once a year for 4 years. At Phase 1 of baseline testing, we assess motor skills, cognitive ability (IQ), basic anthropometry, flexibility and lower body muscle strength, while parents complete an interview and questionnaires regarding family demographics, their child’s physical activity, and behavioural characteristics. Based on the motor skill assessment, some children move on to Phase 2 (longitudinal cohort) and have their body fat percentage, foot structure, aerobic and anaerobic fitness assessed. An accelerometer to measure physical activity is then given to the child and interested family members. The family also receives an accelerometer logbook and 3-day food dairy. At years 2 to 4, children in the longitudinal cohort will have all baseline assessments repeated (excluding the IQ test). Parents will complete an ADHD index twice within the follow-up period. To assess the association between DCDr, fitness and adiposity, our primary analysis will involve longitudinal growth models with fixed effects.

**Discussion:** The CATCH study will provide a clearer understanding of pathways between DCD and health-related fitness necessary to determine the types of interventions children with DCD require.

**Keywords:** Underlying process; Early childhood; Fitness; Body composition; Health.

### Implementing the “Partnering for Change” service delivery model: What do stakeholders say?

C. Camden^**1,2**^, W. Campbell^**2**^, D. Stewart^**2**^, C. Hecimovich^**3**^, L. Dix^**2**^, K. Floyd^**3**^, D. Haughton^**4**^, E. Graham^**2**^, D. McCauley^**2**^ & C. Missiuna^**2**^

^1^Centre de recherche du CHUS & School of Rehabilitation, Sherbrooke University Park, Sherbrooke, J1H 5N4, Canada. Chantal.camden@usherbrooke.ca; ^2^CanChild Center for Childhood Disability Research, McMaster University, Hamilton, Canada; ^3^Central West Community Care Access Centre, Peel, Canada; ^4^Hamilton Niagara Haldimand Brant, Community Care Access Centre, Hamilton, Canada.

**Aim:** Partnering for Change (P4C) is a school-based, occupational therapy service delivery model for children with Developmental Coordination Disorder (DCD). In 2013, P4C was implemented in 40 schools in Ontario, Canada. The goal of this study was to identify stakeholders’ perceptions of the implementation process and outcomes, with an emphasis on the factors that influence the model’s sustainability.

**Method:** Between December 2013 and June 2014, individual interviews were conducted with 5 occupational therapists (OTs), 14 school board managers and principals, 12 health care managers, and 3 research team members. Audio files were transcribed verbatim and entered into QSR NVivo 10 ©. Content analysis was completed using an implementation science framework. Comments relating to the implementation and sustainability of P4C were extracted to identify recommendations for expansion of P4C. In addition, 15 OTs completed daily logs to document services provided to children with DCD as well as requests to provide services to other populations of children.

**Results:** All stakeholder groups perceived P4C to be an effective service delivery model for fostering participation of children with DCD and, potentially, for other children with disabilities. For health care managers, expanding P4C to other populations of children was identified as an equity issue and was recommended to financially sustain P4C. Partnership with schools was also a key factor for success. School stakeholders commented that it was helpful to have an OT as part of their team – to have someone who could problem-solve quickly without requiring a formal referral or the constraint of a diagnosis. OTs were asked to see children with varied needs; they offered 3329 individual sessions to 592 children with coordination difficulties, and were requested to see 435 children with other conditions (70% of whom did receive service). OTs described the supports needed to become part of the school team, to implement all of the P4C components (e.g. universal design learning and accommodation), and to manage large numbers of referrals/requests.

**Discussion:** Stakeholders perceived P4C to be an efficient service delivery model and suggested that effective and sustainable service delivery should be responsive to the needs of all children. Their comments highlighted the many changes and the support required at different levels to implement this kind of model.

**Keywords:** Management; Service delivery model; Stakeholder engagement; Implementation; Intervention.

### School-based educators and occupational therapists collaborating to support students with Developmental Coordination Disorder (DCD)

W. Campbell^**1,2**^, S. Bennett^**3,2**^, C. Camden^**4,2**^, L. Dix^**1,2**^, N. Pollock^**1,2**^, K. Wlodarczyk^**2**^ & C. Missiuna^**1,2,**^

^1^School of Rehabilitation Science, McMaster University, Hamilton, ON, Canada; ^2^CanChild Centre for Childhood Disability Research, McMaster University, Hamilton, ON, Canada; ^3^Department of Teacher Education, Brock University, St. Catharines, ON, L2S 3A1, Canada. sbennett@brocku.ca; ^4^Centre de recherche du CHUS & School of Rehabilitation, Université de Sherbrooke, Quebec, Canada.

**Aim:** An evidence-based occupational therapy (OT) service for children with DCD was introduced in 40 schools to facilitate educators’ capacity to manage children’s needs and to support children’s participation. Using best-practices in educator professional development and job-embedded learning, occupational therapists (OTs) work 1 day/week in the school to: 1) learn school culture; 2) develop relationships with educators; 3) exchange contextual knowledge from the educational setting to facilitate collaboration; 4) increase understanding of DCD; and 5) coach educators in a way that enhances application of new knowledge, skills, and behaviours. The intent is to empower educators to support children with DCD more effectively but also support all children’s motor skill development. Building educator capacity may impact educators’ ability to work successfully with children with DCD and thus reduce secondary disabilities. This presentation will describe educators’ experiences with this service.

**Method:** Educators completed a questionnaire at the start and end of the 1st year of the new OT service that captured educators’ acquisition of knowledge of motor development and ability to identify children with motor delays; transfer knowledge to others; and use information to problem-solve and promote participation. Pre-post questionnaires were matched to measure individual change over time. Semi-structured interviews conducted with a convenience sample of school principals, special educators, and school board administrators further informed the findings.

**Results:** Year 1 pre-post questionnaires showed an increase in educators’ perceptions of their levels of competency and skills working with children with DCD in their classrooms. Interview findings confirmed principals and educators were highly satisfied with this model of service and valued the support and collaborative learning offered. Responses from the 1st year will be matched to questionnaires currently being collected for the 2nd year to further understand educators’ experiences and change in knowledge and skills.

**Discussion:** Findings show that providing collaborative OT services in context can change educators’ knowledge and skills to support children with DCD and enhance the skillset of OTs to consider important contextual school based knowledge, structures and practices when providing services. Implications for health and educational policy, especially with respect to inclusive education, will be discussed.

**Keywords:** Intervention; School-based management; Collaboration; Knowledge translation; Occupational therapy.

### Evidence-based management of children with DCD: What can implementation science offer?

W. Campbell^**1,2**^, C. Camden^**3,2**^, D. Stewart^**1,2**^, E. Graham^**2**^, L. Dix^**1,2**^& C. Missiuna^**1,2**^

^1^School of Rehabilitation Science, McMaster University, Hamilton, ON, L8S 1C7, Canada. campbelw@mcmaster.ca; ^2^CanChild Centre for Childhood Disability Research, McMaster University, Hamilton, ON, Canada; ^3^Centre de recherche du CHUS & School of Rehabilitation, Université de Sherbrooke, Sherbrooke, Quebec, Canada.

**Aim:** Evidence-based management of Developmental Coordination Disorder (DCD) in school-age children requires putting into practice the best available and most current research findings, including evidence that early identification, self-management, prevention of secondary disability, and enhanced participation are the most appropriate foci of school-based occupational therapy. Partnering for Change (P4C) is new model of service delivery based upon these principles that is currently being evaluated in Ontario, Canada. Our experience to date indicates that its implementation in schools is highly complex with many factors influencing its success. This presentation will introduce attendees to a new field called implementation science, which focuses on the study of factors that facilitate uptake of evidence-based practices. We will use the “Active Implementation Driver’s Framework” (Fixsen *et al*., 2005) to examine key factors or “drivers” that impact implementation of P4C, including: competency drivers related to the individuals who deliver the intervention; organization drivers related to how systems and institutions change their practices, processes, and work environment; and leadership drivers related to how well leaders are able to put in place the supports needed to make and sustain change.

**Method:** Focus groups with therapists and individual interviews with stakeholders from health, education, and the research team were conducted between December 2013 and June 2014. Audio files were transcribed verbatim and entered into QSR NVivo 10 ©. Content analysis was conducted by team members to extract macro-level “lessons learned” about implementation from the first year of the P4C evaluation study.

**Results:** Lessons learned about implementation included: (1) systematic training and regular opportunities for mentorship and networking are critical to people and systems making and sustaining change; (2) people in organizations need to dedicate time to building relationships and developing strong communication processes; (3) consistent and ongoing communication with all stakeholders at all levels facilitates change; (4) change needs to be gradual – too much at once may overwhelm individuals and systems; and (5) strong leadership is needed at every level within and across organizations.

**Discussion:** Findings will be related to the Active Implementation Drivers Framework. Implications for implementing new evidence-based practices for children with DCD will be discussed.

**Keywords:** Management of DCD; Evidence-based practice; Implementation science; Research to practice; Qualitative research.

### Feeling better now: The impact of an exercise intervention on self-concept and happiness in adolescents with low motor competence.

E. Clark^**1**^, F. Farringdon^**1**^, B. Hands^**2**^ & F. McIntyre^**1**^

^1^School of Health Sciences, The University of Notre Dame, Fremantle, 6959, Australia. ellouise.clark1@my.nd.edu.au; ^2^The Institute for Health Research, The University of Notre Dame, Fremantle, Australia.

**Aim:** The purpose of the study was to investigate changes in happiness and self-concept in adolescents with low motor competence as a result of being involved in an exercise intervention. Specific aspects of the exercise intervention that may contribute to any changes were also identified.

**Method:** Seventeen adolescents including 12 boys and 5 girls between the ages of 13 and 18 involved in a larger research project (Adolescent Movement Program; AMPitup) participated in the study. The AMPitup program involved two 90-minute exercise sessions a week over 13 weeks, the current study focused on 16 sessions. Participants completed the Short Depression-Happiness scale (SDHS; Joseph, Linley, Harwood & Lewis, 2004) to measure feelings of happiness and the Harter-Self Perception Profile for Adolescents (SPP; Harter, 2012) to measure self-concept before and after the intervention. The AMPitup Environment Assessment Scale (EAS) measured short-term changes in happiness pre and post each session and the impact of contributing factors (exercise, the group and the relationship with trainer). The Wilcoxon Signed Rank Test was used to test significant differences in overall happiness and self-concept. A Chi Square Test, paired T Test and Linear Mixed Model were used to test significant changes in short term happiness and the impact of contributing factors.

**Results:** No significant difference was found pre and post intervention for overall happiness (*p*= .843) or self-concept sub domains. A Chi Square Test revealed significant increases in happiness in 9 out of 16 sessions. Paired T Tests found a significant increase in feelings of total happiness over 16 sessions (*p*=.013). A Linear Mixed Model revealed that the exercise (*p*=.001), group (*p*=.001) and trainer (*p*=.007) were all significant factors in predicting happiness.

**Discussion:** Due to the quantitative method of data collection and small sample size overall happiness and self-concept showed no significant differences. However, significant changes were found in short term happiness for each session and cumulatively across 16 sessions, with the exercise, group and trainer contributing to the increase in happiness. Future research should include qualitative interviews with participants and parents. This will provide further insight into overall happiness and self-concept and what particular aspects of the program they enjoy the most, so that exercise interventions can be individually tailored for adolescents with low motor competence.

**References:**

Harter, S. (2012). *Self perception profile for adolescents: Manual and questionnaires.* Denver, CO: University of Denver.

Joseph, S., Linley, P.A., Harwood, J., Lewis, C.A., & McCollam, P. (2004). Rapid assessment of well-being: The short depression happiness scale (SDHS). *Psychology and Psychotherapy: Theory, Research and Practice, 77*, 463-478. doi: 10.1348/1476083042555406

**Keywords:** Adolescence; Intervention; Exercise; Self-concept; Happiness.

### Diagnosing Developmental Coordination Disorder: The parents’ perspective

E.L. Hill^**1**^, L. Crane^**2**^ & C. Alonso Soriano^**1**^

^1^Department of Psychology, Goldsmiths, University of London, London, SE14 6NW, United Kingdom. e.hill@gold.ac.uk; ^2^Division of Language and Communication Science, City University London, United Kingdom.

**Aim:** Receiving a diagnosis of a developmental disorder has a major impact on a family. The aim of the current research was to highlight the views and experiences of parents who had received a diagnosis of developmental coordination disorder (DCD) for their child in the United Kingdom. Specific aims were: (1) To examine the common ways that DCD presents (e.g. nature of initial concerns regarding their’s child development), and the journey that the parents go through in order to obtain a diagnosis for their child; (2) To evaluate parents’ satisfaction levels with different aspects of the diagnostic process and subsequent support; (3) To investigate which factors affect parental satisfaction, in order to determine areas in which improvements would be beneficial.

**Method:** A total of 228 parents completed an online survey about their experiences of obtaining a diagnosis of DCD for their child. The survey was adapted from a recent study exploring parental experiences of receiving a diagnosis of autism spectrum disorder (Crane *et al*., in press) and questioned parents on: their child’s early development; the age of their child when they first sought professional help; the professionals seen and the outcome of each consultation; the disclosure of the diagnosis; the time taken to receive a diagnosis; their satisfaction with various aspects of the diagnostic process; and the support (if any) that they subsequently received.

**Results:** On average, a diagnosis was confirmed two and a half years after parents initially sought professional help in relation to their child’s motor difficulties. Satisfaction with the diagnostic process was mixed: 45% of parents were dissatisfied and 39% were satisfied. Three factors were predictive of parental satisfaction with the diagnostic process: levels of stress during the process; the manner of the disclosing professional; and satisfaction with post-diagnostic support. Post-diagnostic provision was the area in which parents reported most dissatisfaction, which was not surprising given that 43% of parents were not offered any practical help or support during the diagnostic process or in follow up appointments. A total of 61% of parents reported that their child had a co-occurring condition (often autism, ADHD, or learning/physical disabilities). However, parental satisfaction with the diagnostic process did not vary as a function of whether the child had a comorbid disorder.

**Discussion:** Based on these findings, we propose three key areas in which improvements in the diagnostic process for DCD are needed: (1) Greater awareness about DCD in order to facilitate early recognition; (2) Implementation of clear referral pathways; and (3) Increased post-diagnostic support within health and educational systems.

**References**:

Crane, L., Chester, J. W, Goddard, L., Henry, L. A., & Hill, E. L. (in press). Experiences of autism diagnosis: a survey of over 1000 parents in the United Kingdom. *Autism: The International Journal of Research and Practice*

**Keywords:** Diagnosis; Parents; Support; Satisfaction; Comorbidity.

### The Autonomous Nervous System modulation in children with Developmental Coordination Disorders during motor performance

C.Y. Hiraga, D.T. Gama, P.H. Rocha & A.M. Pellegrini

Laboratory of Motor Development and Learning, Department of Physical Education, University of Sao Paulo State, Av. 24 A, n. 1515, 13506-900, Rio Claro/SP - Brazil. cyhiraga@rc.unesp.br

**Aim:** The aim of the present study was to investigate the modulation of the SNA in children with DCD through Heart Rate Variability (HRV) analyses, during free practice and under timing and precision pressure practice of dominoes lined up setting motor task.

**Method:** Thirty children from 8 to 12 years old participated in this study. Following the American Psychiatric Association (APA-DSM V) criteria for DCD, 30 children were selected to participate in the present study, grouped as follow: 15 DCD children (9 boys and 6 girls, mean age 10.8 y) and 15 Typically Developed (TD) children (9 boys and 6 girls, mean age 10.5 y). Groups were paired by age, gender, Peak Volume of Oxygen uptake (VO2 peak) and Body Mass Index (BMI) outcomes. Spectral, symbolic and conditional entropy HRV analyses of DCD children were compared to Typically Developed (TD) children during resting, free and under pressure practice.

**Results:** As expected, DCD children motor task performance was poorer than TD children`s performance during free and under pressure practice. HRV results indicated that during free practice TD children showed parasympathetic modulations significantly higher than the ones showed by DCD children. During practice under pressure, TD children reduced parasympathetic modulation closer to the level of modulation showed by children with DCD.

**Discussion:** Children with Developmental Coordination Disorders (DCD) show many executive functions difficulties in decision-making, learning, control and memory involved by the Central Nervous System (SNC). Those difficulties impact negatively DCD children development. The Autonomous Nervous System (SNA) modulation is in the context of SNC executive functions. However, during motor performance, the SNA modulation in children with Developmental Coordination Disorder (DCD) has not been focus of empirical systematic investigation. Furthermore, the literature has strongly confirmed that parasympathetic modulation of SNA is positively related to the activation of cerebral centers of executive functions. Therefore, DCD children`s neurophysiologic systemic modulation during motor task free practice and both groups (DT and TDC) neurophysiologic systemic modulation during motor task under timing and precision pressure were not ideal to the motor performance demands throughout executive functions process.

**References**:

Thayer, J., Hansen, A., Saus-Rose, E., & Johnsen, B. (2009). Heart Rate Variability, Prefrontal Neural Function, and Cognitive Performance: The Neurovisceral Integration Perspective on Self-regulation, Adaptation, and Health. *Annals of Behavioral Medicine, 37*(2), 141-153. doi: 10.1007/s12160-009-9101-z

Yoshie, M., Kudo, K., Murakoshi, T., & Ohtsuki, T. (2009). Music performance anxiety in skilled pianists: effects of social-evaluative performance situation on subjective, autonomic, and electromyographic reactions. *Experimental Brain Research, 199*(2), 117-126. doi: 10.1007/s00221-009-1979-y

Zwicker, J. G., Missiuna, C., Harris, S. R., & Boyd, L. A. (2010). Brain activation of children with Developmental Coordination Disorder is different than peers. *Pediatrics, 126*(3), e678-686. doi: 10.1542/peds.2010-0059

**Keywords:** Developmental Coordination Disorder (DCD); Autonomous nervous system; Central nervous system; Heart Rate Variability (HRV); Motor learning.

### Participation and needs of school-aged children with DCD in their living environments: Perceptions of children, parents and teachers

E. Jasmin^**1**^, S. Tétreault^**2**^ & J. Joly^**3**^

^1^Department of Occupational therapy, Université de Sherbrooke, Sherbrooke, Quebec, J1H 5N4, Canada. Emmanuelle.Jasmin@USherbrooke.ca; ^2^Department of Occupational therapy, Haute École spécialisée de Suisse occidentale, Lausanne, Switzerland; ^3^Department of Psychoeducation, Université de Sherbrooke, Sherbrooke, Québec, Canada.

**Aim:** To our knowledge, no study has exhaustively examined the perceptions of children with DCD, their parents and their teachers. However, to be able to offer the best services, a needs assessment should be done, which includes probing the stakeholders (Rossi *et al*., 2004). This study aimed to explore and compare their perceptions regarding the participation and needs of school-aged children with DCD in their living environments.

**Method:** Data were derived from an ecosystemic needs assessment based on a multiple case study design (Jasmin *et al*., 2014). A multiple case study design was chosen because it can be used to describe and analyze, in-depth, a particular social phenomenon in its real context (Yin, 2009). Participants included ten children with DCD, their parents (n = 12) and their teachers (n = 9). Individual semi-structured interviews were conducted with each participant. Qualitative data were analyzed according to Miles and Huberman (2003).

**Results:** Results describe the participation and needs of children with DCD at home, at school and in the community, from the perspective of children, parents and teachers. More specifically, results show that all participants perceived difficulties with school activities. However, participation and needs at home, at school and in the community varied for each child. Children rarely expressed the same expectations as their parents and teachers. Contrary to their parents and teachers, most children did not want more services. Parents and teachers mainly requested additional services at school and training.

**Discussion:** Programs for children with DCD should consider consulting all stakeholders as well as offering diversified services, including indirect interventions. Future studies should examine the effects of consulting and training on DCD for parents and teachers.

**References**:

Jasmin, E., Tetreault, S., & Joly, J. (2014). Ecosystemic needs assessment for children with DCD in elementary school: Multiple case studies. *Physical & Occupational Therapy in Pediatrics,* Early only (20 March 2014).

Miles, M. B., & Huberman, A. M. (2003). *Analyse des données qualitatives* (2^nd^ ed.). Paris: De Boeck Université.

Rossi, P.H., Lipsey, M.W., & Freeman, H.E. (2004). *Evaluation: A systematic approach.* (6th ed.). Thousand Oaks, CA: Sage.

Yin, R.K. (2009). *Case study research: Design and methods* (4^th^ ed.). Los Angeles, CA: Sage Publications.

**Keywords:** needs assessment, perceptions, participation, school-aged children.

### Changes in dynamic balance control over time in children with and without Developmental Coordination Disorder

L.D. Jelsma^**1**^, B.C.M. Smits-Engelsman^**2**^ & R.H. Geuze^**1**^

^1^Clinical and Developmental Neuropsychology, University of Groningen, Grote Kruisstraat 2-1, 9712 TS Groningen, the Netherlands. l.d.jelsma@rug.nl; ^2^Faculty of Kinesiology and Rehabilitation Sciences, KU Leuven, Heverlee Belgium.

**Aim:** Many studies report balance problems in children with Developmental Coordination Disorder (DCD). Dynamic balance, when evaluated as the control of Centre of Pressure (CoP), differs from typical developing to children with DCD. The latter group shows an increase of variability mainly during tasks under complex or changed circumstances. How children with DCD learn to control dynamic balance in dynamic conditions is unknown. This study examines changes over time in a task requiring high level of dynamic balance control (the Wii-Fit ski slalom game). Our hypotheses are 1) children with DCD will show larger and more variable CoP excursions than control children; 2) balance will improve after repetition as measured by a reduction of the variability of the Centre of Pressure (CoP); 3) differences in CoP excursions reflect differences in movement strategies.

**Method:** Twenty eight children with DCD between the age of 6-12 year, participated in this study. Twenty one typically developing children (TD) matched for age and with a score >16th percentile on the Movement Assessment Battery for Children 2 (MABC2) were included as the control group. The Wii balance board was placed on an AMTI force plate. The children played the Wii Fit ski slalom game for ten consecutive runs before (T0) and after (T1) a period of six weeks. The force plate data were analysed for CoP variability and total path length.

**Results:** The control group outperformed the DCD group with an average of 4.5 gates versus 10.5 gates missed. Preliminary analyses of the CoP reveal differences between groups at T0 on variability of the lateral CoP (*F*=7.19, *p*=.011) pathlength (*F*=10.35, *p*=<.01) and counts of change of direction in the anterior-posterior (*F*=15.47, *p*=<.001) and lateral (*F*=4.28, *p*=.047) CoP. Remarkably, the pathlength was longer in the TD group (*M*=.51*,* *SD* 15.8) compared to the DCD group (*M*=.35, *SD* 12.3). After six weeks (T1) both groups had further improved (controls missed 3.4 gates; DCD missed 8.8 gates) and pathlength had become near equal (TD: *M*=.46 (12.7) and DCD: *M*=.40, *SD* 11.5) (see left Figure). Repeated measures ANOVA revealed a difference between groups (*F*(5,28)=2.9, *p*=.03), with a significant interaction effect between group and time for the variability of lateral CoP (*p*=.048), the counted changes in anterior-posterior direction (*p*=.043), and a trend for pathlength (*p*=.066).

**Discussion:** The TD group seems to gain efficiency over time in reducing path length; the BP group seems less active at first, gaining more efficiency over time by increasing path length. As the change of group differences at T0 and T1 does not match the change in performance (# missed gates) we conclude that inaccurate timing may be a better predictor of performance than control of dynamic balance during the first phase of learning.

**Keywords:** Balance control; DCD; Force plate; Centre of Pressure (CoP).

### DCD risk and aerobic and anaerobic fitness in early childhood: Preliminary results from the CATCH Study

S. King-Dowling^**1**^, C. Missiuna^**2**^, B.W. Timmons^**3**^ & J. Cairney^**1**^

^1^Department of Family Medicine, McMaster University, Hamilton, L8P 0A1, Canada; kingds@mcmaster.ca; ^2^School of Rehabilitation Science, McMaster University, Hamilton, Canada; ^3^Department of Pediatrics, McMaster University, Hamilton, Canada.

**Aim:** Poor motor skills have been associated with lower aerobic and anaerobic fitness, but the few studies on preschool children have relied on field-based physical fitness assessments. The purpose of this study was to determine if 4- and 5-year old children at risk for Developmental Coordination Disorder (DCDr) exhibit lower short term muscle power (STMP), and decreased aerobic fitness compared to their typically developing (TD) peers.

**Methods:** Participants include 4- to 5-year olds enrolled (N=112; n= 66 DCDr; n=73 boys) into the longitudinal component of the Coordination and Activity Tracking in CHildren (CATCH) study to date. Motor skills were assessed using the *Movement Assessment Battery for Children version 2* (MABC2). STMP, including peak power (PP/kg) and mean power (MP/kg), was evaluated using a 30-second Wingate protocol on a pediatric cycle ergometer. Time to exhaustion on a maximal treadmill test (Bruce Protocol) and 2 min heart rate recovery (HRR) following the test were used as indicators of aerobic fitness. Only children who could pedal >25 rpm and who reached a max HR of at least 180 beats per minute (bpm) were included in the analysis (n=101). Independent t-tests were conducted to evaluate differences between DCDr (≤16th percentile on the M-ABC-2) and TD children.

**Results:** DCDr children had lower relative PP (4.9±1.3 vs. 5.4±1.1 W/kg, p=.044), and MP (3.1±1.2 vs. 3.8±1.2 W/kg, p=.008) on the Wingate test and reached exhaustion faster on the treadmill (578±105 vs. 646±91 s, p=.002). At 2-minutes following exercise, HRR did not differ between groups (82.9±12.9 vs. 79.4±14.0 bpm, p=.216).

**Discussion:** Preschool children at risk for DCD have decreased anaerobic and aerobic fitness performance compared with children without movement difficulties. Although children fatigued faster on the maximal aerobic fitness test, their ability to recover following exercise was not impaired. Poorer performance on the tests in DCDr children may be due to coordination difficulties with the task rather than fitness levels per se. The failure to find significant differences in HRR may be due to the early developmental age of subjects: poor aerobic fitness may not yet be present in young children with DCD, but develops over time given chronic inactivity and other risk factors.

**Keywords:** Early childhood; Aerobic fitness; Anaerobic fitness.

### Parental influences on physical activity behavior in children at risk for DCD

M. Kwan^**1,2**^, M. Greenway^**2**^, C. Missiuna^**3**^, B. Timmons^**4**^ & J. Cairney^**1**^

^1^Department of Family Medicine, McMaster University, Hamilton, L8P 0A1, Canada. kwanmy@mcmaster.ca; ^2^Michael DeGroote School of Medicine Niagara Regional Campus, McMaster University, St. Catharines, Canada; ^3^School of Rehabilitation Science, McMaster University, Hamilton, Canada; ^4^Department of Pediatrics, McMaster University, Hamilton, Canada.

**Aim:** In addition to academic and self-care difficulties, there is compelling research showing children with Developmental Coordination Disorder (DCD) engage in less physical activity (PA) than their typically-developing (TD) peers^1^. There is very little in the research literature, however, which examines PA behaviours and the psychosocial factors related to PA in young children with motoric difficulties. The purpose of the current study was to compare objectively assessed PA behaviours of children at risk for DCD (DCDr) and TD children, and the relationships between PA and salient parental influences.

**Methods:** Children recruited as a part of the Coordination and Activity Tracking in CHildren (CATCH) study were included (N=86; 60% DCDr; 57% boys). Parents completed a questionnaire measuring their perceived importance of PA, enjoyment of PA, parental support for PA, and perceptions of their child’s competence. Additionally, each child wore an RT3 Triaxial Actical Accelerometer for seven consecutive days.

**Results:** Univariate analysis of variance found no significant differences in time spent in moderate-to-vigorous PA between DCDr (*M*= 76.32 +20.62) and TD children (*M*= 74.42 +19.73). Similarly, no differences were found in light PA or sedentary behaviours. Correlation analyses did not find significant relationships between children’s PA and parental influences, but there was a significant difference in parents’ perceived competence for DCDr and TD children (F(1,82) = 21.86, p<.001).

**Discussion:** Findings suggest that there are no differences in PA behaviours between DCDr children and their TD peers during early childhood. As expected, parents of DCDr children reported lower perceived competence for their child; however, parental enjoyment, support, and perceived importance of activity were unrelated to a young child’s PA. Overall, preliminary findings suggest that these young children tend to be fairly active, working toward the progression of 60 minutes of energetic activity by age 5, as per PA recommendations. While PA behaviours do not appear to be influenced by motoric capabilities and parental influences during this early life stage, future research will examine how these patterns of PA and its relationships change over time, potentially identifying the critical periods and targets for intervention efforts.

Reference:

1. Rivilis, I., Hay, J., Cairney, J., Klentrou, P., Liu, J., & Faught, B. E. (2011). Physical activity and fitness in children with developmental coordination disorder: A systematic review. *Research in Developmental Disabilities, 32,* 894-910.

**Keywords:** Early childhood; Physical activity; Parental influences; Accelerometers.

### Gender differences in physical fitness and overweight between children with and without developmental coordination disorder

N. Lifshitz^**1**^, S. Raz-Silbiger^**2,3**^, N. Weintraub^**2**^, S. Steinhart^**3**^, S.A. Cermak^**4**^ & N. Katz^**5**^

^1^Department of Occupational Therapy, Faculty of Health Professions, Ono Academic College, 104 Zahal St., Kiryat ono, 55000, Israel. niritlif@gmail.com; ^2^School of Occupational Therapy, Hebrew University Jerusalem, Israel; ^3^Alyn Hospital, Pediatric & Adolescent Rehabilitation Center, Jerusalem, Israel; ^4^Mrs. T.H. Chan Division of Occupational Science and Occupational Therapy, University of Southern California, Los Angeles, USA; ^5^Research Institute for Health and Medical Professions, Ono Academic College, Israel.

**Aims:** 1) To examine gender differences in physical fitness and overweight in a sample of DCD children in comparison to typical children in Israel; 2) To examine the same differences within a comparable US sample.

**Method:** DCD was identified through total scores on the Movement Assessment Battery for Children 2 (MABC-2) equal to or less than the 16th percentile as well as parents’ report that the child’s deficits in motor skills interfered with at least two daily life activities. Participants from Israel included children with DCD [n=22, M age =8.70(1.36) years, 16 boys, 6 girls] and typical children [n=47, M age=8.90(1.52), 34 boys, 13 girls]. From the US DCD [n=31, M age=9.3(1.5) 24 boys, 7 girls] and typical [n=44, M age=9.5(1.4) 26 boys, 18 girls]. Measures included the Strength subtest of the Bruininks-Oseretsky Test of Motor Proficiency (BOT-2), the Six Minute Walk Test (6MWT) with heart rate measure, BMI and the percentage of body fat.

**Results:** Significant differences between DCD and typical children were found on all variables of physical fitness (BOT-2 and 6MWT) in both cultural samples. A two-way analysis of variance (ANOVA) (group/ gender) within the Israeli sample revealed significant interactions for the percentage of body fat (F=8.51, p<.005) and BMI (F=4.50, p<.038) meaning that less fit children were more obese. Within the DCD group significant differences were found between girls and boys, where girls had higher % body fat (t = 2.36, p < .028); and approached significance for BMI (t=1.89, p<.074) in the same direction. However, in the US none of these interactions were significant.

**Discussion:** The current study supports previous findings that children with DCD are less physically fit and more overweight compared to typically developing children. Moreover, in comparing between the genders within the Israeli sample, the girls weighed more and had a significantly higher percentage of body fat, this was not found for boys. Thus, it is important to further our understanding of the relationships between obesity, physical fitness and gender among children with and without DCD.

**References**:

Cermak, S. A., Katz, N., Weintraub, N., Steinhart, S., Raz-Silbiger, S., Munoz, M., Must, A, Rakaczky, S., Bandini, L., Curtin, C., Gleason, J., & Lifshitz, N. (Accepted). Participation in physical activity, fitness, and risk for obesity in children with Developmental Coordination Disorder: A cross cultural study. *Occupational Therapy International*.

**Keywords:** Gender; Physical fitness; Overweight.

### Kinematic movement analysis in handwriting: A diagnostic tool to enhance training in writers with and without DCD

C. Marquardt & M. Diaz-Meyer

Schreibmotorik Institut e.V., Schwanweg 1, 90562 Heroldsberg, Germany. christian.marquardt@schreibmotorik-institut.com

**Aim:** Kinematic analysis of writing movements allows differentiating between disturbed and undisturbed movement components and thereby various patterns of impairment. Application includes diagnosing writing impairments and planning therapy in DCD, as well as documenting training success and prevention. In the education sector, the respective performance level when learning to write can be measured. Based on the results specific and personalised training programmes can be tailored. Such an approach may prevent future writing problems from occurring early on.

**Method:** The CSWin programme registers writing movements using graphic tablets (Marquardt, 2012a). An assessment lasting approximately 10 minutes measures a sample of normal writing, simple letters and basic movements (isolated wrist and finger movements, loops). The subsequent analysis of e.g. writing frequency, degree of automation and writing pressure makes it possible to evaluate the performance in tasks of graduated difficulty and to define specific problem areas.

**Results:** Studies from the therapy sector have proven that inadequate compensation and control strategies of the motor system may cause pathological cramps. With an appropriately aligned, therapeutic approach to systematically detecting and using preserved movement potentials, it was possible to achieve good treatment results in patients with writer’s cramp (Mai & Marquardt, 1994).

In the same line of thinking, we examined if children can call upon existing movement competences in form-focussed writing classes. A recent study with 130 pupils in the first grade showed good results in simple coordination movements, but at the same time often poor performance in writing tasks. A training study demonstrated that pupils (aged 6 and over) can benefit substantially from specific motor writing exercises (Marquardt, 2012b).

**Discussion:** Kinematic analyses can be used to objectify and quantitatively describe observations of writing movements. This information is suitable for a differentiated diagnosis of writing problems and for planning customised training. In many cases, however, existing movement skills can also be discovered that can then be used systematically in training and therapy of DCD, as well as in the education sector for preventative purposes.

**References**:

Mai, N., & Marquardt, C. (1994). Treatment of writer’s cramp. Kinematic measures as an assessment tool for planning and evaluating training procedures. In C. Faure, P. Keuss, G. Lorette, & A. Vinter (Eds.), *Advances in handwriting & drawing: a multidisciplinary approach* (pp. 445-461). Paris: Europia.

Marquardt, C. (2012a). *CSWin – Kinematic analysis of handwriting movements, computer programme*. Munich: MedCom.

Marquardt, C. (2012b). *Effects of motor training on writing performance in grade one pupils*. Unpublished pilot study, Vienna.

**Keywords:** Diagnosis; Handwriting; Motor skills; Kinematic writing analysis; CSWin programme.

### A quiet eye intervention to improve the catching performance of children with Developmental Coordination Disorder

C.A.L. Miles^**1,2**^, G. Wood^**2**^, S.J. Vine^**1**^, J.N.Vickers^**3**^ & M.R. Wilson^**1**^

^1^Department of Sport and Health Science, University of Exeter, St. Luke’s Campus, Heavitree Rd, Exeter, EX1 2LU, UK. milesc@hope.ac.uk; ^2^Department of Health Sciences, Liverpool Hope University, Hope Park, Liverpool, UK; ^3^Faculty of Kinesiology, University of Calgary, 2500 University Drive NW, Calgary, Canada.

**Aim:** Our pilot study^1^ found that teaching a specific gaze strategy - quiet eye training (QET) - was more effective than traditional training (TT) methods for teaching a throw and catch task to typically developing 8-10 year old children. The current study aimed to apply the technique to children with Developmental Coordination Disorder (DCD).

**Method:** 30 children diagnosed with DCD were randomly allocated into TT or QET intervention groups. The training consisted of 3 instructional videos, each followed by a set of catching practices with verbal reinforcement of the training points from an experimenter. Both groups were given standard catching instructions taken from the UK Physical Education Curriculumn, but the QET group received additional emphasis on increasing their pre-throw (QE1) and pre-catch (QE2) QE durations. Quantitative and qualitative catching performance, Elbow angle and QE durations were collected at pre/post-training and 6-week retention.

**Results:** The QET group significantly increased QE durations from pre-training to delayed retention (QE1 = +247ms, QE2 = +19%) whereas the TT group experienced a reduction (QE1 = -74ms, QE2 = -4%). QET participants showed significant improvement in the quality of their catch attempts and increased elbow flexion at catch compared to the TT group (QET = +28°, TT = +1°).

**Discussion:** QET may be an effective adjunct to traditional instructions, for therapists teaching visuomotor skills to children with DCD.

**References**:

1. Miles, C. A. L., Vine, S. J., Wood. G., Vickers, J. N., & Wilson, M. R. (2014). Quiet eye training improves throw and catch performance in children. *Psychology of Sport and Exercise, 15*, 511-515.

**Keywords:** DCD; Motor control; Visuomotor; Prediction; Children.

### Finding the children who “fall through the cracks”: Describing children with DCD who are identified using the Partnering for Change service delivery model

C. Missiuna^**1,2**^, W. Campbell^**1,2**^, N. Pollock^**1,2**^, C. DeCola^**2**^, C. Camden^**3,2**^ & D. McCauley^**2**^

^1^School of Rehabilitation Science, McMaster University, Hamilton, ON, L8S 1C7, Canada. missiuna@mcmaster.ca; ^2^CanChild Centre for Childhood Disability Research, McMaster University, Hamilton, ON, Canada; ^3^Centre de recherche du CHUS & School of Rehabilitation, Université de Sherbrooke, Sherbrooke, Quebec, J1H 5N4, Canada.

**Aim:** Partnering for Change (P4C) is an innovative, tiered model for providing school-based occupational therapy. An occupational therapist (OT) spends one day per week providing classroom-based services in which the goal is to build educators’ capacity to support children with DCD. In the current study, P4C was implemented in 40 schools in which hundreds of children who had probable DCD had been on a waitlist for up to 2 years. OTs provided P4C service to these children and also identified new children who they suspected had DCD. This presentation will describe the characteristics of both groups.

**Method:** In the 2013-2014 school year, 241 children waiting for OT services were transferred from waitlists to the P4C service; OTs identified an additional 351 children who had not been previously recognized. From this pool, the families of 392 children agreed to participate in the research study (305 boys and 87 girls): 171 came from waitlists and 221 were identified by OTs. This presentation will report data from the 244 families (62%) who returned demographic and health questionnaires and completed the DCD-Q.

**Results:** The 171 children referred from waitlists were, on average, one year older than those children identified directly by OTs (mean age = 8.78 vs. 7.79 years). Children referred from waitlists were more likely to have multiple diagnoses than those identified by OTs (64% vs. 57%). Regardless of referral source, the most frequent comorbidities were attention difficulties, learning disabilities, and speech delay. Interestingly, children identified by OTs were 3 times more likely to have anxiety problems than children on waitlists. Perhaps most notably, children from the waitlists were more likely to have a formal individualized education plan at school (49% vs. 28%). DCD-Q scores indicated that over half of the children in both referral groups met criteria for probable DCD; however, while only 18% of the children on waitlists were younger than 8 years, 41% of those referred by OTs were less than age 8.

**Discussion:** Delivering health services at a whole school level leads to much earlier identification of children with DCD and successfully identifies many children who otherwise would “fall through the cracks.” Implications for enhanced equity and accessibility of school-based therapy services will be discussed.

**Keywords:** Comorbidity; School-based interventions; Early identification; Early intervention; Description of DCD.

### Meta review of systematic and meta analytic reviews on the intervention outcome for children with Developmental Coordination Disorder

M. Miyahara^**1**^, M. Lagisz^**2**^ & S. Nakagawa^**2**^

^1^School of Physical Education, Sport and Exercise Sciences, University of Otago, POBox 56, Dunedin, 9011, New Zealand. motohide.miyahara@otago.ac.nz; ^2^Department of Zoology, University of Otago, Dunedin, New Zealand.

**Aim:** Systematic and meta analytic reviews have been conducted to evaluate and summarise existing empirical evidence. Because the findings from the reviews are often used for informed decision-making, it is critical to ensure that the reviews are undertaken properly and that the conclusions drawn from the reviews are trustworthy. To date, no attempt has been made to appraise the quality of systematic review and meta analysis on the effectiveness of intervention for children with developmental coordination disorder (DCD). This meta review, or a review of the reviews, is the first of its kind for this population.

**Method:** We conducted a review of published systematic and meta-analytic reviews on the intervention outcome for children with DCD between 1806 and October 2014. The methodological quality of the identified systematic and meta-analytic reviews was independently assessed by two assessors with the assessment of multiple systematic reviews (AMSTAR)^1^. Disagreements of the assessment were resolved by discussion. The methodological quality score of the AMSTAR was expressed as a percentage of full score.

**Results:** The literature search yielded a total of four reviews^2–5^ on the intervention outcome for children with DCD from 1996 until 2014. The AMSTAR percentage quality scores of the four reviewed studies ranged from 0 to 55 percent. No previous review has provided *a priori* review design, a search for “grey literature” or unpublished studies, a thorough homogeneity analysis with forest plots, an assessment of publication bias, or adequate disclosure of conflict of interest.

**Discussion:** The quality scores of the reviews progressively improved over the years. However, there is a room for further improvement. By fully addressing the shortcomings, future reviews would become of higher quality, thus offering more trustworthy and useful findings for evidence-based practice.

**References**:

1. Shea, B. J., Grimshaw, J. M., Wells, G. A., Boers, M., Andersson, N., Hamel, C., Porter, A.C., Tugwell, P., Moher, D., & Bouter, L. M. (2007). Development of AMSTAR: a measurement tool to assess the methodological quality of systematic reviews. *BMC Medical Research Methodology 7,* 1471-2288.

2. Miyahara, M. (1996). A meta-analysis of intervention studies on children with developmental coordination disorder. *Corpus, Psyche et Societas, 3,* 11-18.

3. Pless, M., & Carlsson, M. (2000). Effects of motor skill intervention on developmental coordination disorder: a meta-analysis. *Adapted Physical Activity Quarterly, 17,* 381–401.

4. Hillier, S. L. (2007). Intervention for children with developmental coordination disorder: a systematic review. *The Internet Journal of Allied Health Science and Practice, 5,* 1–11.

5. Smits-Engelsman, B. C. M., Blank, R., Van Der Kaay, A., et al. (2013). Efficacy of interventions to improve motor performance in children with developmental coordination disorder: a combined systematic review and meta-analysis. *Developmental Medicine and Child Neurology, 55,* 229–237.

**Keywords:** Evidence based practice; Systematic review; Meta analysis; Developmental Coordination Disorder; Intervention outcome.

### Playful learning to write with a particular Teacher: feasibility of the digital writing program Teacher in a Box in education and rehabilitation

A. Overvelde^**1**^, M. Jongbloed-Pereboom^**2**^, & M.W.G. Nijhuis-van der Sanden^**1**^

^1^Radboud university medical center, Radboud Institute for Health Sciences, IQ healthcare, Postbus 9101, 6500 HB Nijmegen, The Netherlands. Anneloes.Overvelde@radboudumc.nl; ^2^Radboud University, Behavioural Science Institute, Nijmegen, the Netherlands.

**Aim:** A serious game –Teacher in a Box (TiaB; in Dutch Juf-in-a-Box, www.jufinabox.com)- was developed to train young children with fine motor skills disorder to practice preparatory writing movements with a stylus on an interactive tablet, built into a classroom desk. TiaB is based on principles of neuromotor task training^1^. In this exploratory study, feasibility and preliminary efficacy of TiaB training on motor handwriting skills were investigated.

**Method:** Three groups of children (6-8 yrs old) participated: children in regular education (RE; n=23), children with motor handwriting problems, who were treated by a pediatric physical therapist (PPT; n=22), and children in special education (SE; n=16). Children were instructed to train on TiaB for 2 times/week, 25 minutes during 6 weeks^2^. Feasibility was investigated after training using semi-structured interviews with children, teachers, and therapists. Time on task and the amount of different tasks were recorded during each training session. Quality of motor performance was assessed pre- and post training with a testing module within TiaB.

**Results:** Mean grades for the children for TiaB were 7.9 (RE) and 8.6 (SE and PPT) on a 10 point scale. Most of the children preferred practice with TiaB above traditional paper-pencil practice. Feasibility was graded with a mean of 7.5 by the teachers and therapists. Rating for independent practice differed between teachers (mean 8.9) and therapists (mean 2.6). Points of criticism were mainly focused on technical problems and logistics. Mean practice time was only 147.7 minutes (SD 78.8) with a significant difference between groups (p<0.001): the PPT practiced the most 204.4 min (SD 82.3), and the SE the least with 97.9 min (SD 47.2). Over 88% of mean practice time was spent on the basic level of exercises. No differences were found on accuracy and velocity between pre- and post training. The effect of training differed significantly between groups: The PPT group improved on velocity with unchanged level of accuracy. In both pre- and post test, accuracy was significantly higher for the RE group with a lower velocity compared to the PPT and SE group.

**Discussion:** This particular Teacher speaks to the children. Preliminary effect on motor performance was proved in the PPT group. To improve practice time, the development of an application in a handheld tablet was recommended. Release of this app-version Monster Zoo is planned in April 2015.

**References**:

1. Blank, R., Smits-Engelsman, B., Polatajko, H., & Wilson, P. (2012). European Academy for Childhood Disability (EACD): Recommendations on the definition, diagnosis and intervention of developmental coordination disorder (long version). *Developmental Medicine & Child Neurology, 54*, 54–93.

2. Hoy, M. M. P., Egan, M. Y., & Feder, K. P. (2011). A systematic review of interventions to improve handwriting. *Canadian Journal of Occupational Therapy, 78*(1), 13-25.

**Keywords:** Developmental Coordination Disorder; Handwriting; Intervention; Serious gaming; Learning to write.

### Development and reliability of the Motor Teaching Principles Taxonomy - a tool to study the use of motor teaching principles in intervention

M.M. Schoemaker^**1**^, H.A. Reinders-Messelink^**2,3**^, A. Peek^**1**^, M. Okken^**1**^, I. van der Veer^**4**^ & B.C.M. Smits-Engelsman^**5,6**^

^1^University of Groningen, University Medical Centre Groningen, Centre for Human Movement Sciences, Groningen, The Netherlands. m.m.schoemaker@umcg.nl; ^2^Rehabilitation Centre Friesland, Beetsterzwaag, The Netherlands; ^3^University of Groningen, University Medical Center Groningen, Department of Rehabilitation Medicine, Groningen, The Netherlands; ^4^Avans+, University for Professionals, Master Pediatric Physical Therapy, Breda, The Netherlands; ^5^Faculty of Kinesiology and Rehabilitation Sciences, Katholieke Universiteit Leuven, Leuven, Belgium; ^6^Department of Health and Rehabilitation Sciences, Faculty of Health Sciences, University of Cape Town, Groote Schuur Hospital, Cape Town, South Africa.

**Aim:** Physical and occupational therapists aim to improve motor performance in children with Developmental Coordination Disorder (DCD) by using motor teaching principles. Examples of motor teaching principles are how to instruct children, how to guide practice, how to provide feedback about their performance, and how to impart knowledge. It is known from literature that some motor teaching principles are more effective than others. In 2003, a pilot version of the Motor Teaching Principles Taxonomy (MTPT) was developed (Niemeijer, Smits-Engelsman, Reynders & Schoemaker, 2003). Aim of the current study was to adapt this taxonomy in order to include recent knowledge about motor teaching, and to investigate the utility and reliability of the taxonomy. The ultimate goal of this study is to use the Motor Teaching Principles Taxonomy as a tool to describe which motor teaching principles are used in interventions of children with DCD.

**Method:** Several steps were taken to adapt the original MTPT. First, relevant textbooks and papers about motor learning were studied to update the original MTPT. Second, a Delphi round was held with pediatric physical therapists to incorporate their practical knowledge about motor teaching principles in the taxonomy. This process resulted in an updated version of the original MTPT. Two intervention sessions of about 10 children with DCD treated by pediatric physical therapists were recorded. Next, master students in Human Movement Science observed these recordings and classified all verbal actions aimed to enhance motor learning using the taxonomy. Both inter- and intra-reliability were determined by calculating Cohen’s Kappa.

**Results:** The new MTPT covers four categories: Instructing, Sharing knowledge before a task is completed, Providing feedback, and Sharing knowledge after a task is completed. The results regarding intra-rater reliability and inter-rater reliability will be presented during the conference, as data gathering is still ongoing.

**Discussion:** The implications of the results for further research and clinical practice will be discussed during the conference.

**References**:

Niemeijer, A.S., Smits-Engelsman, B.C.M., Reynders, K., & Schoemaker, M.M. (2003). Verbal actions by physiotherapists to enhance motor learning of children with DCD. *Human Movement Science, 2*(4-5), 567-581*.*

**Keywords:** DCD; Intervention; Motor learning; Taxonomy.

### Sleep disturbance in children with and without Developmental Coordination Disorder

M. Sparrowhawk^**1**^, A. Barnett^**1**^ & L. Wiggs^**1**^

^1^Department of Psychology, Social Work & Public Health, Oxford Brookes University, Headington Campus, Oxford, OX3 0BP, U.K. m.sparrowhawk@brookes.ac.uk

**Aim:** Impaired sleep is associated with a range of negative effects on children’s quality of life and their behavioural, emotional and cognitive functioning. Previous research has shown a higher rate of sleep disturbance in children with a range of developmental disorders in comparison to the general population. In our previous work parents of children with and without DCD completed the Children’s Sleep Habits Questionnaire (CSHQ). This showed significantly higher sleep disturbance for children with DCD compared to controls. Sub-scale scores indicated particular problems with bedtime resistance, parasomnias and daytime sleepiness. These results suggested that sleep patterns of children with DCD should be investigated further. The aim of the current study was to examine sleep in DCD using more extensive and objective measures.

**Method:** Two groups of 15 children with DCD were recruited, one from primary school (7-11 years), one from secondary school (11-16 years). Typically developing children were individually matched on age and gender to the children with DCD. Sleep behaviour was assessed using the CSHQ plus actigraphy (movement sensors), which provided an objective assessment of the children’s sleep-wake patterns over one week. Aspects of self-rated child functioning which may be associated with the presence of sleep disturbance were assessed with various questionnaires (Pre-sleep Arousal Scale, Pediatric Daytime Sleepiness Scale, PedsQL Multidimensional Fatigue Scale). The Maternal Cognitions about Infant Sleep Questionnaire was used to assess parents’ thoughts about their child’s sleep.

**Results:** The DCD group scored significantly higher than controls on the CSHQ, indicating greater overall parent-reported sleep disturbance. In particular, sleep duration, parasomnias and daytime sleepiness were problematic. Actigraphy indicated that the nocturnal sleep of children with DCD was objectively more fragmented than controls’. The DCD group reported feeling more cognitively aroused before sleep and feeling more sleepy during the day than the control children.

**Discussion:** The children with DCD had more problematic sleep than typically developing children, according to both objective measures and parent report. The underlying cause of this disturbance cannot be ascertained from this study but is an important area for future research. There seems to be a link between the sleep problems and daytime tiredness. Awareness of sleep problems in DCD is important for early identification and implementation of support to potentially ease tiredness during the day, which might enable children to cope better during the daytime.

**Keywords:** Sleep; Actigraphy; Daytime sleepiness; Parasomnias; Pre-sleep cognitive arousal.

## Poster Presentations

### Evaluating motor performance and the presence of signs of inattention/hyperactivity in 6-years-old Brazilian children

O.S. Agostini^**1**^, C.R.S. Araújo^**2**^ & L.C. Magalhães^**3**^

^1^Departamento de Terapia Ocupacional, Universidade Federal do Rio de Janeiro, Brazil, Rua Prof. Rodolpho Paulo Rocco, s/n - Prédio CCS - Cidade Universitária - Ilha do Fundão, Rio de Janeiro, RJ, 21910-590, Brazil. oliviagostini@gmail.com; ^2^Departamento de Terapia Ocupacional, Universidade Federal da Paraíba, João Pessoa, Brazil; ^3^Departamento de Terapia Ocupacional, Universidade Federal de Minas Gerais, Belo Horizonte, Brazil.

**Aim:** To investigate the motor performance and the presence of signs of inattention and hyperactivity/impulsivity in 6-years-old Brazilian children.

**Method:** Six year old Brazilian children randomly selected from private and public schools were evaluated with the Movement Assessment of Battery for Children 2nd Edition (MABC-2)^1^, the Developmental Coordination Disorder Questionnaire (DCDQ-Brazil)^2^ and the Swanson, Nolan and Pelham IV Scale (SNAP-IV)^3^. An occupational therapist and a research student assessed all children with the MABC-2 and their parents and teachers completed the DCDQ - Brazil and SNAP-IV, respectively.

**Results:** A total of 85 children (42 girls - 49.4%) considered as typically developing participated in the study, their mean age was 77.91 (± 3.17) months and 77 (90, 6%) were enrolled in the first grade of elementary schools and eight (9.4%) were enrolled in Childhood Education (preschool). According to the results of the SNAP-IV, nine (10.6%) children showed signs of inattention, six (7.1%) showed signs of hyperactivity/impulsivity, one (1.2%) showed signs of inattention and hyperactivity/impulsivity; one (1.2%) questionnaire was not returned. Regarding performance on the MABC-2, two children (2.4%) had results bellow the 5th percentile, three (3.5%) had suspect results and 80 children performed above the 15th percentile. Based on the DCDQ-Brazil, the scores of 8 (8.4%) children indicated possible DCD. Combining results, on the SNAP-IV, of the eight children who showed signs of DCD on the DCDQ-Brazil, half had symptoms of inattention (3) and combination of inattention and hyperactivity (1). Among the five children with results suggestive of DCD on the MABC-2, one showed signs of inattention and other presented signs of inattention and hyperactivity/impulsivity on the SNAP-IV. There was agreement between the MABC-2 and DCDQ-Brazil on only two children regarding the presence of motor problems.

**Discussion:** Even though the study was conducted with typically developing children, 5.9% children were found to present signs of motor coordination problems. Children with probable DCD, as defined by the MABC-2 and DCDQ-Brazil, showed more signs of hyperactivity and inattention, however, the motor test and questionnaire identified motor problems in different children. Future studies should further investigate the relationship between the DCDQ-Brazil and the MABC-2 as well as the co-occurrence of motor performance and attentional problems among Brazilian children.

**References**:

Henderson, S. E., Sugden, D. A., & Barnet, A. (2007). *Movement assessment battery for children (MABC-2)*. (2^nd^ ed). San Antonio: The Psychological Corporation.

Mattos, P., Serra Pinheiro, M. A., Rodhe, L. A., & Pinto, D. (2006). Apresentação de uma versão em português para o uso no Brasil do instrumento MTA-SNAP IV de Avaliação de sintomas de transtorno do déficit de atenção/hiperatividade e sintomas de transtorno desafiador e de oposição. *Revista de Psiquiatria, 28*(3), 290-7.

Prado, M. S. S., Magalhães, L. C., & Wilson, B. N. (2009). Cross-cultural adaptation of the Developmental Coordination Disorder Questionnaire for brazilian children. *Revista Brasileira de Fisioterapia, 13*(3), 236-243.

**Keywords:** Assessment; Comorbidity; Children; DCD; ADHD.

### Visual perception’s comparison of term and preterm children with Developmental Coordination Disorder

T. Altunalan^**1**^ & A.M. Çalışkan^**2**^

^1^Department of Early Intervention Unit, Turkey’s Spastic Children Foundation, 34750, Turkey. turgayaltunalan@yahoo.com; ^2^Department of Pediatric Neurology, Istanbul Medical Faculty, The University of Istanbul, Turkey.

**Aim:** Prevalence of Developmental Coordination Disorder (DCD) is 5-6% for term infants, and 40-50% for preterm infants^1^. Children with DCD may also have attention deficit disorder, hyperactivity, speech and learning problems^2,3^. This study aims to compare visual perception of preterm born children with DCD to term born children with DCD.

**Method:** Initially, 60 children (thirty of them born under 32 weeks of gestational age and the others were term) between ages of 4-6, were included in the study. We excluded children whose diagnoses are cerebral palsy, intrauterine growth restriction, loss of hearing and vision, mental retardation. Movement Assessment Battery-Second Edition and Frostig Developmental Test of Visual Perception have been used for assessment of motor skills and visual perception. We continued the study with 18 children (7 term, 11 preterm) who had <15th centile in Movement ABC-2 test. Due to Frostig test <60th centile was accepted as a visual perception problem. Sub parameters of Frostig test (eye motor coordination, figure-ground, form constancy, position in space, spatial relations) and Frostig total point were compared to the term and preterm groups who diagnosed as DCD. There isn’t any significant difference between children according to preschool education and social-economical status of their families (p>0,05).

**Results:** As a result of Frostig test, visual perception problem was detected as 91.9% (n:10) for the preterm group with DCD, and 14% (n:1) for the term group with DCD. When the groups were compared, hand-eye motor coordination and Frostig total score of preterm group with DCD were found significantly lower than term group with DCD (p=0.005 and p=0.001). Although no statistically significant difference were detected for other parameters of Frostig test (figure-ground, form constancy, position in space, spatial relations), average scores were found lower for the preterm group with DCD.

**Discussion:** The motor, learning, social and speech ability problems caused by prenatal, perinatal and postnatal risk factors that affect the immature brain of preterm infants are still discussed. When the children with DCD are evaluated and treated it is needed to demonstrate particular attention to the accompanying visual perception and learning problems.

**References**:

1. Blank, R., Smits-Engelsman, B., Polatajko, H., & Wilson, P. (2012). European Academy for Childhood Disability (EACD): Recommendations on the definition, diagnosis and intervention of developmental coordination disorder (long version). *Developmental Medicine & Child Neurology, 54*, 54–93.

2. Visser, J. (2003). Developmental coordination disorder: a review of research on subtypes and comorbidities. *Human Movement Science, ­22*(4-5), 479-493.

3. Santosuosso, J., Strand, K., Surran, B. B., Rosman, N. P., & Augustyn, M. (2012). Developmental Coordination Disorder plus: A diagnosis by exclusion? *Journal of Developmental & Behavioral Pediatrics, 33*(9), 746-748.

**Keywords:** Developmental coordination disorder; Preterm; Visual perception; Term; Frostig.

### Parents’ perception of the effects of Cognitive Orientation to daily Occupational Performance on participation and performance of children with Developmental Coordination Disorder out of therapy

C.R.S. Araújo^**1**^, L.C. Magalhães^**2**^, T.B. Pontes^**3**^, A.S. Franca^**1**^ & E. Oliveira^**1**^

^1^Department of Occupational Therapy, Health Sciences Center, Federal University of Paraiba, Campus I, s/n - Castelo Branco, João Pessoa, PB, 58051-900, Brazil. clariceribeiro@hotmail.com/ clarice@ccs.ufpb.br; ^2^Department of Occupational Therapy, Federal University of Minas Gerais, Belo Horizonte, Brazil; ^3^Ceilandia Faculty, University of Brasilia, Brasilia, Brazil.

**Aim:** To investigate parents’ perception about the effects of occupational therapy on children’s activities and participation.

**Method:** Qualitative study with in-depth interviews with eight parents of children who were engaged in occupational therapy based on the Cognitive Orientation to Daily Occupational Performance (CO-OP). After thirteen occupational therapy sessions using CO-OP, mothers were interviewed and asked about the effects of CO-OP on performance on activities and participation of their children. The interviews were analyzed using Lawrence Bardin’s content analysis.

**Results:** Parents perceived improvements on activities and participation of their children, and improvements in other abilities and activities that were not the focus on therapy – transfer of learning. Parents indicated relevant factors such as the process of learning, the influence of the occupational therapist, the puppet use (Mr. Goal-Plan-Do-Check) and the amount of information provided during therapy.

**Discussion:** Participation of parents was determinant for children’s progress. Limitations were the absence of more comprehensive measure of characterization of families. Research which addresses the influence and the level of participation of parents in the treatment of their children should be encouraged.

**References**:

1. American Psychiatry Association (2014). *Manual diagnóstico e estatístico de transtornos mentais: DSM-V*. 5.ed. Porto Alegre, RS: Artes Médicas.

2. Araújo, C. R. S., Magalhães, L. C., & Cardoso, A. A. (2011). Uso da *Cognitive Orientation to Daily Occupational Performance* (CO-OP) com crianças com transtorno do desenvolvimento da coordenação. *Revista Brasileira de Terapia Ocupacional da Universidade Federal de São Paulo*, 22(3), 245-253.

3. Henderson S. E., Sugden, D. A., & Barnett, A. L. (2007). *The Movement Assessment Battery for Children*. Second Edition. London, UK: Harcourt Assessment.

4. Jarus, T., Lourie-Gelberg, Y., Engel-Yeger, B., & Bart, O. (2011). Participation patterns of school-aged children with and without DCD. *Research in Developmental Disabilities, 32,* 1323-1331.

5. Missiuna, C, Moll, S, Law, M, King, S, & King, G. (2006). Mysteries and mazes: parents’ experiences of children with developmental coordination disorder. *Canadian Journal of Occupational Therapy, 73*(1), 7-17.

6. Missiuna, C., Moll, S., King, S., King, G., & Law, M. (2007). A trajectory of troubles: parents’ impressions of the impact of developmental coordination disorder. *Physical and occupational Therapy in Pediatrics, 27*(1), 87-101.

7. Missiuna, C., Pollock, N., & Law, M. (2004). *Perceived Efficacy and Goal Setting System (PEGS)*. San Antonio, TX: Psychological Corporation.

8. Polatajko, H. J., & Mandich, A. (2004). *Enabling occupation in children: The Cognitive Orientation to Daily Occupational Performance (CO-OP) approach*. CAOT Publications ACE, Ottawa, Ontario.

**Keywords:** Motor skills disorders; Parents; Perception; Intervention studies; Occupational therapy.

### Physical fitness and body mass index in children with Developmental Coordination Disorder

J.C.S.M. Arcay, P.R.H. Rocha, A.C. Ricco, L.M. Nakamura, L.E. Malvezzi & C.Y. Hiraga

Department of Physical Education, São Paulo State University, Rio Claro, 13506-900, Brazil. cyhiraga@rc.unesp.br

**Aim:** A number of studies have shown that children with developmental coordination disorder (DCD) show poor performance on health-related physical fitness components^1^. Specifically, physical fitness performance is lower than that of their typically developing (TD) peers^2^. Many of the studies comparing physical fitness performance between children with and without DCD have not taking into account the body mass index (BMI) status of their participants. The present study examines (1) whether overweight/obesity influences on physical fitness components of children with and without DCD; and (2) whether different BMIs (normal BMI vs. overweight/obesity) influence on physical fitness of children with DCD.

**Method:** A total of forty-nine children aged between 7 to 10 years participated in the study. Movement Assessment Battery for Children 2 (MABC-2) was used to identify children with DCD and TD children. The assessment of physical fitness of all participants was done by the sit and reach, long jump, modified pull-up, curl-up and 9-min run tests. Comparison analyses on physical fitness components between children with DCD and overweight/obesity (DCD_o/o) vs. typically developing overweight/obesity children (TD_o/o) were done. Furthermore, comparisons between children with DCD and normal BMI (DCD_nBMI) vs. children with DCD and overweight/obesity (DCD_o/o) were carried out. For a fair comparison between groups, participants were matched by age, gender and BMI status.

**Results:** The results of comparisons between DCD_o/o vs. DT_o/o indicated that physical fitness of DCD_o/o group was significantly lower than that of the TD_o/o group for the standing long jump test, t(34) = -3.8, p < .01, modified pull-up test, Z(34) = -2.0, p < .05 and curl-up t(34) = -2.9, p < .01. There was no significant difference between groups for sit and reach test and 9-min run test. The results of comparisons between DCD_nBMI vs. DCD_o/o, showed that there were no significant differences on physical fitness components (sit and reach, modified pull-up, curl-up and 9-min run tests) between children with DCD_nBMI and DCD_o/o, except for the long jump test, t(24) = 3.1, p < .01.

**Discussion:** Overall, the results of the present study suggest that BMI, overweigh/obese, appears not to determine the performance of physical fitness components of children with DCD, but their own coordination problem seems to be decisive to low physical fitness.

**References**:

1. Rivilis, I., Hay, J., Cairney, J., Klentrou, P., Liu, J. A., & Faught, B. E. (2011). Physical activity and fitness in children with developmental coordination disorder: a systematic review. *Research Developmental Disabilities, 32*, 894-910.

2. Haga, M. (2009). Physical fitness in children with high motor competence is different from that in children with low motor competence. *Physical Therapy, 89*, 1089-1097.

**Keywords:** Infancy; Health; Motor coordination; Motor difficulty.

### Development of an adult motor skills questionnaire

A. Barnett^**1**^, G. Illingworth^**1**^, R. Egloff^**1**^, S.E. Henderson^**2**^ & D.A. Sugden^**3**^

^1^Department of Psychology, Social Work & Public Health, Oxford Brookes University, Headington Campus, Oxford, OX3 0BP, U.K. abarnett@brookes.ac.uk; ^2^Department of Psychology and Human Development, UCL Institute of Education, University College London, U.K.; ^3^School of Education, University of Leeds, U.K.

**Aim:** Some adults experience motor difficulties that have a negative impact on their ability to perform everyday life tasks at home, work and/or in education. Developmental Coordination Disorder (DCD) often persists into adulthood, with continuing difficulties with a range of self-care tasks, handwriting, sports, recreational activities and driving. For others, motor difficulties are associated with known physical or neurological deficits. Whatever the nature of the motor difficulties, it is important to identify individuals and assess their motor difficulties so that appropriate support can be provided by educators and employers. Self-report questionnaires can provide useful information as part of a broader assessment. The aim of the current study was to develop a self-report, online questionnaire for young adults, focused on everyday life motor skills.

**Method:** *Item Generation:* Items were generated using the DSM-5 criteria for DCD, a review of the literature on adult motor difficulties, our own experience and existing adult and child questionnaires. The questionnaire comprised a short section on difficulties in childhood and a section on current difficulties in adulthood. The latter had six parts: Daily Living skills; Everyday Movement; Study/Work skills; Sports/Recreational skills; Participation and Driving. A 4-point scoring scale similar to that in the MABC-2 Checklist was used. *Piloting items:* Minor amendments were made to wording, following feedback from eight adults. *Expert Validity:* Ten experts in the field, with experience of developing and using tests and of working with individuals with motor difficulties, were asked to provide feedback on the content and clarity of the questionnaire. The experts included psychologists, occupational therapists, teachers and student disability officers. Revisions to items and wording were made on the basis of information received from this panel of experts. *Online formatting:* The revised questionnaire was prepared for online completion, using Qualtrics software. *Validity & Reliability:* Preliminary aspects of validity and reliability were investigated by asking 120 adults aged 18-35 to complete the questionnaire online. Factor analysis was used to explore the number of constructs assessed. Internal consistency and test-retest reliability were also examined.

**Results and Discussion:** Findings from this preliminary study will be presented and planned future work on the instrument outlined.

**Keywords:** Adults; Motor assessment; Questionnaire; Self-report.

### The persistence of motor difficulties and its effect on participation in school-aged children

O. Bart^**1**^, M.T. Cohen^**1**^ & A. Mimouni-Bloch^**2**^

^1^Department of Occupational Therapy, Sackler Faculty of Medicine, Tel Aviv University, Tel Aviv 69978, Israel. oritbert@post.tau.ac.il; ^2^Department of Child Development, Loewenstein Hospital Rehabilitation center, Raanana Israel.

**Aim:** Disabilities limit opportunities for children. Occupational therapy seeks to improve a child’s physical, cognitive, and social emotional health by increasing participation in daily living activities. This study examined two questions: Do motor difficulties diagnosed in kindergarten age children continue into school age? How does participation of children with motor difficulties differ from that of typical children?

**Method:** Sixty children age 7-12 years comprised the research group. Children were previously diagnosed with motor difficulties, when they were 3-6 years old, by the Child Development Unit at Loewenstein Hospital. The control group comprised 30 typical children matched for age and gender. Parents were contacted by telephone by the primary investigator and were asked to complete four standardized questionnaires: The Children Participation Questionnaire (CPQ), The Child Performance Skills Questionnaire (CPSQ), and The Developmental Coordination Disorder Questionnaire (DCDQ).

**Results:** Children formerly identified with motor difficulties in kindergarten continued to score lower on the DCDQ compared to their typical peers and they had lower scores in motor, organization, and communication skills. In addition, significant differences were found between the groups in two measures of participation; child independence in favor for typically developed children and child enjoyment in favor for the study group.

**Discussion:** Motor difficulties do not disappear with age. Significant differences in performance skills suggest more effort required for participation, which may affect its quality. Children with motor difficulties may benefit from intervention to improve skills and participation.

**Keywords:** DCD; Participation; Motor skills; Process skills; Motor ability.

### Response inhibition in children with Developmental Coordination Disorder and motor difficulties

M. Bernardi^**1**^, H.C. Leonard^**2**^, E.L. Hill^**2**^& L.A. Henry

^1^Division of Language and Communication Science, School of Health Sciences, City University London, London, EC1V 0HB, UK. Marialivia.Bernardi@city.ac.uk; ^2^Department of Psychology, Goldsmiths, University of London, UK.

**Aim:** The study investigated response inhibition (RI) in children with impaired and typical motor skills. It was expected that children with poor motor skills would be less accurate and slower at inhibiting motor responses than children without motor impairments, while performing appropriately when inhibiting verbal responses.

**Method:** Participants were 91 children aged 7-11 years, split into three groups: children with a clinical diagnosis of Developmental Coordination Disorder (DCD), children with motor difficulties (MD) but no diagnosis, and typically-developing (TD) children. Children with co-occurring neurodevelopmental disorders were not included in the DCD group in order to assess the extent of the impact of motor impairments *only* on RI. RI was measured using the Verbal Inhibition Motor Inhibition test, in which the response to inhibit was either a word (verbal task) or a gesture (motor task). The Total Error and the Total Completion Time were used as measures of RI accuracy and speed, respectively, and processing speed measures were also included in the analyses.

**Results:** In the motor RI task children with DCD and MD produced more errors than TD children but did not take longer to complete the task. In the verbal task, children with DCD and MD were as accurate as TD children but the DCD group was significantly slower than the other groups at inhibiting verbal responses. These differences were evident even after subclinical symptoms of inattention and hyperactivity were taken into account. When processing speed was controlled for in the analyses, the DCD group remained slower than the other groups on the verbal RI task.

**Discussion:** Children with poor motor skills (both DCD and MD) demonstrated significant difficulties in inhibiting motor responses. It may be that the motor demand of the task impacted on their ability to respond accurately and discouraged them from taking extra time to attempt to perform well. Children with DCD and MD performed as accurately as TD children in RI tasks once the motor demand was removed. However, the slower performance of children with DCD in the verbal RI task may reflect inefficiency with the process of inhibiting a response, such that typical levels of accuracy can only be obtained at the expense of very slow and careful responding. Future studies should include children with DCD and co-occurring disorders in order to compare RI across a clinical sample.

**Keywords:** Response inhibition; Processing speed; Motor difficulties; Developmental Coordination Disorder; Executive functioning.

### Concurrent motor and language disorders: What status for this specific group? Synthesis of three studies

M. Biotteau^**1,2**^, J.-M. Albaret^**3**^& Y. Chaix^**1,2,4**^

^1^Inserm, Imagerie Cérébrale et Handicaps Neurologiques UMR 825, CHU Purpan, Place du Dr Baylac, F-31059 Toulouse Cedex 9, France. maelle.biotteau@inserm.fr; ^2^Université de Toulouse III, UPS, Imagerie Cérébrale et Handicaps Neurologiques UMR 825, CHU Purpan, Place du Dr Baylac, F-31059 Toulouse Cedex 9, France; ^3^Université de Toulouse III, UPS, PRISSMH-EA4561, 118 Route de Narbonne, F-31062 Toulouse Cedex 9, France; ^4^Hôpital des Enfants, Centre Hospitalier Universitaire de Toulouse, CHU Purpan, Place du Dr Baylac, F-31059 Toulouse Cedex 9, France.

**Aim:** Motor impairments in dyslexia were observed long ago (Denckla, 1985; Haslum, 1989) and overlap between Developmental Coordination Disorder (DCD) and Developmental Dyslexia (DD) is substantial, with over half of dyslexic children having DCD and conversely. However, few studies give details on such association. To attempt to bridge this gap, our team members have conducted three studies based on the same sample composed of children with only dyslexia, or only DCD or dyslexia and DCD. Those three levels of analysis have allowed us to compare the intellectual, behavioural and neural characteristics of these three populations (DD, DCD and DD+DCD). We especially paid a particular concern to the profiles of children with co-occurrence.

**Method:** A group of 65 children (21♀-44♂) were recruited: 20 DD (8♀-12♂), 22 DCD (6♀-16♂) and 23 DD+DCD (7♀-16♂). Inclusion criteria were: 8 to 12 years old, with DCD or DD or DCD and DD, no known psychiatric or neurological disorder and an IQ score greater than 70. Diagnosis DD and DCD were in accordance with the DSM-IV-TR criteria with M-ABC test for motor skills and L’Alouette and ODEDYS-2 test for reading skills. Children with Specific Language Impairment and/or Attention Deficit/Hyperactivity Disorder according to the DSM-IV-TR criteria were excluded. Participants were submitted to the same evaluation, comprising an assessment of intellectual abilities (WISC-IV), procedural learning (Finger Tapping Task) and a functional brain imaging (FMRI).

**Results:** Firstly, none of our three studies showed any evidence of compounding effects of dual-diagnosis. Comorbidity does not seems to constitute an aggravating factor. There is no additive effect, never on intellectual abilities, nor behavioural skills or brain organization. Secondly, there was no differences between DD and DD+DCD groups, but important difference was observed between DCD children and the two other groups. Surprisingly, characteristics which were clearly typical of the DCD group (visuospatial deficiency, procedural learning difficulties or additional activations in the attentional circuit), did not appear in the comorbid group.

**Discussion:** The results from the children with dual association are particularly relevant, highlighting a feature of the comorbid group, which behaves like the DD group, and on several levels (behavioural, neurological and cognitive aspects). All together, this raises the question of the status of the comorbid group: as a sub-group of DD or an independent disorder, closer to DD than to DCD? This finding is a real improvement and constitutes an important basis for further reflection. Now, the question is naturally what will happen to this specific status, given the specificities of their motor and functional skills, and what therapy and medical care can best help them overcome their difficulties?

**Keywords:** Comorbidity; Developmental Coordination Disorder; Developmental Dyslexia.

### Neural correlates of early motor learning in adults with and without DCD

D. Brady, J. Van Velzen & E. Hill

Department of Psychology, Goldsmiths, University of London, New Cross, London, SE14 6NW, UK. Dan.brady@gold.ac.uk

**Aim:** There is increasing evidence that the primary motor cortex (M1) plays a role in the early stages of motor learning^1,2,3^, and there are genetic and environmental factors influencing M1 plasticity which may affect motor learning^4,5^. There is also evidence that the degree of connectivity between M1 and other areas can be used to predict subsequent motor learning^6^. Despite this there has been little research looking at the potential contribution of the primary motor cortex to the motor learning difficulties experience by individuals diagnosed with DCD^7^. Therefore the aim of this study is look at the neural correlates of movement during the early stages of motor learning and from these correlates attempt to determine whether the primary motor cortex could be implicated in the disorder. A secondary aim was to look at the connectivity between M1 and other areas in adults with and without DCD to determine if there were any significant and reliable differences.

**Method:** Participants: Right-handed adults, aged 18-35, diagnosed with DCD or synonymous condition (e.g. dyspraxia), and age-matched, typically-developing controls. All participants will undertake the modified Movement ABC^8^ to differentiate those with DCD and those without.

**Task:** Participants practice a simple motor learning task involving responding to numbers presented on screen by pressing the corresponding key on a numerical keypad. This will be divided into several blocks.

**Measures:** Behavioural performance (accuracy and reaction time) for the task was recorded. Resting state EEG was recorded before and after the task, and EEG was also recorded throughout the task.

**Results:** Data collection is on-going and preliminary results will presented at the conference. Behavioural data will be analysed to determine if participants showed an improvement over the course of the blocks. The EEG recorded during the task will be analysed looking at changes in Movement related event related potentials, specifically the amplitude and onset, as well as changes in event-related mu desynchronisation over the course of the blocks. Both of these neural correlates have been shown to be localised to the primary motor cortex^9,10^. In addition the resting state data will be analysed to look at differences in connectivity between the participants diagnosed with DCD and the typically developing participants, and any differences in connectivity before and after completion of the task.

**References**:

1. Floyer-Lea, A., & Matthews, P. M. (2005). Distinguishable brain activation networks for short- and long-term motor skill learning. *Journal of Neurophysiology, 94*(1), 512–518. doi:10.1152/jn.00717.2004

2. Muellbacher, W., Ziemann, U., Wissel, J., Dang, N., Kofler, M., Facchini, S., … Hallett, M. (2002). Early consolidation in human primary motor cortex. *Nature, 415*(6872), 640–644. doi:10.1038/nature712

3. Steele, C. J., & Penhune, V. (2010). Specific increases within global decreases: a functional magnetic resonance imaging investigation of five days of motor sequence learning. *The Journal of Neuroscience, 30*(24), 8332–8341. doi:10.1523/JNEUROSCI.5569-09.2010

4. Missitzi, J., Gentner, R., Geladas, N., Politis, P., Karandreas, N., Classen, J., & Klissouras, V. V. (2010). Plasticity in human motor cortex is in part genetically determined. *The Journal of Physiology, 589*(Pt 2), 297–306. doi:10.1113/jphysiol.2010.200600

5. Pitcher, J. B., Schneider, L. A., Burns, N. R., Drysdale, J. L., Higgins, R. D., Ridding, M., … Robinson, J. S. (2012). Reduced corticomotor excitability and motor skills development in children born preterm. *The Journal of Physiology, 590*(Pt 22), 5827-5844. doi:10.1113/jphysiol.2012.239269

6. Wu, J., Srinivasan, R., Kaur, A., & Cramer, S. C. (2014). Resting-state cortical connectivity predicts motor skill acquisition. *NeuroImage, 91*, 84–90. doi:10.1016/j.neuroimage.2014.01.026

7. Zwicker, J. G., Missiuna, C., & Boyd, L. A. (2009). Neural correlates of developmental coordination disorder: a review of hypotheses. *Journal of Child Neurology, 24*(10), 1273–81.

8. Cousins, M., & Smyth, M. M. (2003). Developmental coordination impairments in adulthood. *Human Movement Science, 22*(4-5), 433–459.

9. Cui, R. Q., Huter, D., Lang, W., & Deecke, L. (1999). Neuroimage of voluntary movement: topography of the Bereitschaftspotential, a 64-channel DC current source density study. *NeuroImage, 9*(1), 124–34.

10. Taniguchi, M., Kato, A., Fujita, N., Hirata, M., Tanaka, H., Kihara, T., … Yoshimine, T. (2000). Movement-related desynchronization of the cerebral cortex studied with spatially filtered magnetoencephalography. *NeuroImage, 12*(3), 298–306. doi:10.1006/nimg.2000.0611

**Keywords:** EEG; Connectivity; ERP; Motor learning; Adults; DCD.

### Differences in motor milestone attainment in 4- and 5-year old children at risk for Developmental Coordination Disorder

E. Bremer^**1**^, C. Missiuna^**2**^, B.W. Timmons^**3**^ & J. Cairney^**1**^

^1^Infant and Child Health Lab, Department of Family Medicine, McMaster University, Hamilton, L8P 0A1, Canada. bremeree@mcmaster.ca; ^2^School of Rehabilitation Science, McMaster University, Hamilton, Canada; ^3^Department of Pediatrics, McMaster University, Hamilton, Canada.

**Aim:** Attaining motor milestones such as standing and walking are important aspects of a child’s overall motor development^1.^ Children with Developmental Coordination Disorder (DCD) experience significant movement difficulties^2^; however, it is not well understood when children with DCD begin to fall behind their peers with typical development (TD) developmentally. We examined the average age motor milestones were attained in children at risk for DCD (DCDr) and TD children. The relationship between average age of motor milestone attainment and current level of motor coordination was also examined.

**Method:** The sample was a part of the larger Coordination and Activity Tracking in CHildren (CATCH) study, comprised of 133 children 4-to-5 years of age (n= 64 DCDr; n= 87 boys). All participants were assessed with the *Movement Assessment Battery for Children version 2* (MABC2), and parents provided information regarding the age (in months) that their child achieved the following milestones: sitting without support, standing with assistance, crawling on hands and knees, walking with assistance, standing alone, and walking alone. Independent t-tests were conducted to evaluate differences in the average age the milestones were achieved between DCDr (≤16th percentile on the MABC2) and TD children. Pearson product correlations were conducted between age of motor milestone attainment and MABC2 scores.

**Results:** TD children achieved 3 motor milestones at significantly younger ages than DCDr children: sitting without support (t(131)=-2.207, p=.029), crawling on hands and knees (t(131)=-2.109, p=.037), and walking alone (t(131)=-2.335, p=.021). MABC2 scores were significantly negatively correlated with sitting without support (r=-0.173, p=.046), walking with assistance (r=-0.176, p=.043), and walking alone (r=-0.197, p=.023).

**Discussion:** The results suggest that DCDr children achieve motor milestones such as sitting, crawling, and walking at a later age than TD children; however, they still often achieve these milestones within typical time frames. We also found that children who achieve their motor milestones earlier may be more likely to have better motor coordination in early childhood.

**References**:

1. Payne, V. G., & Isaacs L. D. (2002). *Human motor development: A lifespan approach* (5^th^ ed.). Boston, MA: McGraw Hill.

2. Cairney, J. (Ed.) (2015). *Developmental Coordination Disorder and its consequences*. Toronto, ON: University of Toronto Press.

**Keywords:** Early childhood; Motor milestone; Motor coordination.

### Children with Developmental Coordination Disorder demonstrate a spatial mismatch when using coincident-timing ability with tools

P. Caçola^**1**^, M. Ibana^**1**^, M. Ricard^**1**^ & C. Gabbard^**2**^

^1^Department of Kinesiology, The University of Texas at Arlington, Arlington, TX, 76019, USA. cacola@uta.edu; ^2^Department of Health and Kinesiology, Texas A&M University, College Station, TX, 77843, USA

**Aim:** Compared to typically developing (TD) children, those with Developmental Coordination Disorder (DCD) have difficulties estimating reach to a stationary target with a long tool (e.g., 40 cm). Here, we compared estimation accuracy of children with DCD and TD children when using a dynamic action representation of coincident-timing ability with their hand and various tool lengths. Coincident-timing, or interception ability, is defined as performing a motor response at the same time (coincident) that a moving object arrives at a designated intercept point.

**Method:** Forty-seven participants performed in this study; 25 DCD (8.92 ± 1.47 years) and 22 TD (9.41 ± 1.94 years). DCD diagnosis complied with the DSM-5 and all children scored below the 15th percentile for motor skills with the MABC-2. The task involved estimating reach and intercepting a target moving towards the subject along the midline under 5 conditions (hand, and tools of 10-, 20-, 30-, and 40 cm length). Participants were instructed to imagine themselves reaching out with their hand or tool and press the center button of a keypad when they believed the target had arrived at their interception point.

**Results:** A 2x5 repeated measures ANOVA was used to compare “match” (stop distance – actual distance) values in the five conditions. The group main effect was significant indicating that children with DCD were significantly less accurate than TD children in all conditions (*ps*<.05). Overall, all participants overestimated their reach ability, with children with DCD overestimating an average of 6.65 cm more than TD children.

**Discussion:** These results indicate that children with DCD are less accurate in a coincident-timing context and show greater overestimation with TD children. It appears that children with DCD have problems with spatially representing a motor action that requires the use of coincident-timing; an ability that may apply to common motor activities such as catching a ball or striking a baseball pitch. The results also support the notion that children with DCD do not have an efficient strategy for terminal accuracy, and tend to move earlier (faster) than their peers. Furthermore, that ability does not seem to be constrained by the length of the tool used.

**Keywords:** Developmental Coordination Disorder; Tool; Coincident-timing; Interception; Action representation; Motor imagery.

### Increasing families’ perceived knowledge and competency for managing DCD: Working with parents and clinicians to share an online evidence-based workshop

C. Camden^**1**^, D. Anaby^**2**^, F. Leger^**3**^, M-C. Rheaume^**4**^, M-E. Langevin^**5**^, C. Remillard^**4**^, C. Gauthier-Boudreault^**1**^, V. Foley^**1**^, K. ^­^Shikako-Thomas^**2**^, L. Rivard^**6**^, A. Sylvestre^**7**^, R. Gaines^**8**^ & C. Missiuna^**6**^

^1^Centre de recherche du CHUS, Université de Sherbrooke, Sherbrooke, Québec, J1H 5N4, Canada. chantal.camden@usherbrooke.ca; ^2^Centre de recherche interdisciplinaire en Réadaptation, McGill University, Montréal, Québec, Canada; ^3^Centre de réadaptation de l’Estrie, Sherbrooke, QuébecCanada; ^4^Association québécoise pour les enfants dyspraxiques (AQED), Sherbrooke, Québec, Canada; ^5^Institut de réadaptation en déficience physique de Québec (IRDPQ), Québec, Canada; ^6^CanChild, McMaster University, Hamilton, Canada; ^7^Centre Interdisciplinaire de Recherche en Réadaptation et Intégration Sociale (CIRRIS), Laval University, Québec, Canada; ^8^Children’s Hospital of Eastern Ontario, Ottawa University, Ottawa, Canada.

**Aim:** Developmental Coordination Disorder (DCD) is a chronic and prevalent health condition that impacts on daily functioning of children and increases the risk for preventable secondary health issues. Few tools are available to transfer knowledge to families about how to effectively manage DCD. The goal of this study was to evaluate change in parents’ perceived knowledge and competence managing their own children and explore the impact on daily life following completion of an online evidence-based workshop translated into French.

**Method:** This collaborative research used a pre-post mixed methods design. A working committee including representatives of two clinical centers and a DCD parent association met monthly to overview the study and facilitate recruitment. Participants were invited to complete online questionnaires before and after completion of the workshop, and three months later. Descriptive and thematic analyses were performed.

**Results:** 170, 106 and 90 participants (mostly mothers of a child with DCD in Québec) completed the pre, post and follow up questionnaires, respectively. Most participants heard about the study by the DCD association or through Internet (92%). Most items (79%) evaluating self-reported knowledge and skills increased significantly (p<0.001) following workshop completion. Three months later, this increase was maintained. Participants reported sharing information with others and modifying attitudes (e.g., being more supportive) and daily routines (e.g., homework) to better support children with DCD.

**Discussion:** Clinicians and the parent association appreciated having access to this tool to share with families, and reported that it helped them supporting parents to understand and manage better DCD.

**Keywords:** Knowledge translation; Participatory-action research; Developmental Coordination Disorder.

### Localisation of touch in children with developmental coordination disorder (DCD): the ‘crossed hands effect’

J.S. Camp^**1**^, J. Begum Ali^**1,2**^, E.L. Hill^**1**^ & A.J. Bremner^**1**^

^1^Sensorimotor Development Research Unit, Department of Psychology, Goldsmiths, University of London, New Cross, London SE14 6NW, UK. j.camp@gold.ac.uk; ^2^Centre for Brain and Cognitive Development, Department of Psychological Sciences, Birkbeck, University of London, London, UK.

**Aim:** Skilled action, which children with developmental coordination disorder (DCD) experience difficulties with in everyday life (DSM-5, 2013)^1^, requires a representation of the body and limbs. Here we report an investigation of one aspect of body representation, tactile localisation, in children with DCD. When localising touches to the hands, TD adults show a “crossed hands effect” whereby identifying which hand received a tactile stimulus is less accurate when the hands are crossed than uncrossed^2^. This effect has been taken to indicate the use of an external frame of reference for locating touches, and has been demonstrated in children and infants as young as 6 months of age^3,4^. However, it is not apparent in congenitally blind individuals, suggesting that the use of an external reference frame is reliant on visual development^5^. As children with DCD are thought to rely more heavily on vision than TD children for some tasks^6^, we hypothesised that they would show a greater crossed hands effect than controls.

**Method:** Children with DCD aged 7-11 years are currently completing a tactile localisation task^3^. Participants feel a gentle vibrotactile stimulus on one of two fingers, each situated underneath a cuddly toy, and report whether the “penguin” or the “hedgehog” tickled their finger. Across four blocks, posture (uncrossed, crossed) and view (seen, unseen) are varied systematically. TD control and DCD participants were individually matched on the Raven’s Coloured Progressive Matrices (Raven, 2004) to within two points. Children in the DCD group had received a previous DCD/dyspraxia diagnosis. Existing Movement-ABC (2nd edition) data was available for a subset of 12: of these, two were on the 16th percentile overall while the remaining children scored below the 15th percentile.

**Results:** Preliminary findings (n=14 per group; target ns=20) indicate lower percentage accuracy for the DCD group (M=95.5%) than the TD group (M=98.6%) overall (Mann-Whitney U = 47.0, *p*=.019). Crossed conditions (M=95.2%) were completed with lower accuracy than uncrossed conditions (M=98.9%), and this applied to both groups (TD: Wilcoxon Z=-2.12, *p* =.034; DCD: Wilcoxon Z=-2.22, *p* =.026). There was neither a significant main effect of View nor any reliable interactions.

**Discussion:** These preliminary findings demonstrate: a) poorer performance of children with DCD when locating touch than controls matched for nonverbal ability and chronological age, and b) a crossed hands effect in the DCD group. Comparisons with the younger motor-matched group, available by the time of the conference, should facilitate a description of these abilities with respect to motor development.

**References**:

1. American Psychiatric Association. (2013). *Diagnostic and statistical manual of mental disorders* (5^th^ ed.). Washington, DC: APA.

2. Shore, D. I., Spry, E., & Spence, C. (2002). Confusing the mind by crossing the hands. *Cognitive Brain Research, 14*(1), 153-163.

3. Begum Ali, J., Cowie, D., & Bremner, A. J. (2014). Effects of posture on tactile localization by 4 years of age are modulated by sight of the hands: evidence for an early acquired external spatial frame of reference for touch. *Developmental Science, 17*(6), 935-943.

4. Bremner, A. J., Holmes, N. P., & Spence, C. (2008). Infants lost in (peripersonal) space?. *Trends in Cognitive Sciences, 12*(8), 298-305.

5. Röder, B., Rösler, F., & Spence, C. (2004). Early vision impairs tactile perception in the blind. *Current Biology, 14*(2), 121-124.

6. Deconinck, F. J. A., De Clercq, D., Savelsbergh, G. J., Van Coster, R., Oostra, A., Dewitte, G., & Lenoir, M. (2006). Visual contribution to walking in children with developmental coordination disorder. *Child: Care, Health and Development, 32*(6), 711-722.

**Keywords:** Developmental Coordination Disorder; Tactile localisation; Body representation.

### Children with suspected Developmental Coordination Disorder: What do parents and teachers say about their social-emotional and behavioral functioning?

W. Campbell^**1,2**^, S. Bennett^**3,2**^, C. Camden^**4,2**^, R. Gaines^**5,2**^, N. Pollock^**1,2**^, D. McCauley^**2**^, C. DeCola^**2**^, J. Cairney^**6,2**^ & C. Missiuna^**1,2**^

^1^School of Rehabilitation Science, McMaster University, Hamilton, ON, L8S 1C7, Canada. campbelw@mcmaster.ca; ^2^CanChild Centre for Childhood Disability Research, McMaster University, Hamilton, Canada; ^3^Department of Teacher Education, Brock University, St. Catharines, Canada; ^4^Centre de recherche du CHUS & School of Rehabilitation, Université de Sherbrooke, Sherbrooke, Quebec, Canada; ^5^Children’s Hospital of Eastern Ontario Research Institute, Ottawa, ON, Canada; ^6^Department of Family Medicine, McMaster University, Hamilton, ON, Canada.

**Aim:** Children with Developmental Coordination Disorder (DCD) are at risk for developing comorbid social and emotional problems, especially if they are not recognized early and do not receive adequate support. *Partnering for Change (P4C)* is an evidence-informed occupational therapy intervention that uses a health promotion approach coupled with a focus on knowledge translation to enhance early identification, build capacity of others to manage DCD, and prevent secondary consequences. In the current study, we are following 392 children with suspected DCD over two school years to examine the impact of the P4C service on two key outcomes: (1) social-emotional and behavioral functioning and (2) school functioning and participation. This presentation will compare parents’ and teachers’ initial evaluation of children’s social-emotional and behavioral functioning.

**Method:** Of 392 participants, 171 children were referred from waitlists, with another 221 children identified by the P4C occupational therapists (OTs). Parents and educators were asked to complete the Strengths and Difficulties Questionnaire (SDQ) to document children’s initial levels of emotional, conduct, hyperactivity, and peer problems. From the original sample, 188 (48%) had SDQs completed by both parents and teachers at the initial evaluation (94 from waitlists and 94 referred by OTs).

**Results:** In general, parents and educators agreed with respect to their ratings of children on the SDQ. Issues with hyperactivity were viewed by both parents and educators to be the most noticeable problem for children irrespective of how they had been referred to the P4C service. Interestingly, parents whose children were referred to P4C by the OT (whose problems had gone unnoticed by the school system prior to this study), were more likely to report concerns about emotional problems than teachers.

**Discussion:** Our results indicate that parents of children whose motor coordination needs have not been previously recognized by the school system are noticing that their children are struggling emotionally. This suggests that secondary social-emotional consequences may already be developing in these children. Implications for the benefit of P4C in the early identification of children with DCD will be discussed. Consideration should be given to the potential role of this type of service delivery in reducing secondary complications associated with DCD.

**Keywords:** Comorbidity; Social-emotional problems; Secondary consequences; Intervention; Prevention.

### Psychometric investigation of the Dutch version of the Little Developmental Coordination Disorder Questionnaire (Little DCDQ-NL)

M.H. Cantell^**1**^, S. Houwen^**1**^ & M.M. Schoemaker^**2**^

^1^Department of Special Needs Education and Youth Care, Faculty of Behavioral and Social Sciences, University of Groningen; ^2^Centre for Human Movement Sciences, University Medical Centre Groningen University of Groningen, Groningen, The Netherlands. m.h.cantell@rug.nl

**Aim:** The aim of this study was to investigate the validity and reliability of the Dutch version of the Little Developmental Coordination Disorder Questionnaire (Little DCDQ-NL).

**Method:** A convenience sample of 192 parents of preschool aged children (79 girls, 113 boys; 40 three year olds, 57 four year olds and 95 five year olds) was recruited in the North of the Netherlands. In addition to the Little DCDQ-NL, the parents filled out a set of questionnaires, and children were tested with the Movement ABC2-NL (M-ABC2-NL; Henderson, Sugden, Barnett, & Smits-Engelsman, 2010). The internal consistency of the 15 items of the Little DCDQ-NL was determined by Cronbach’s alpha to measure reliability. Construct validity was investigated using factor analyses. Concurrent validity was measured by calculating correlations between the Little DCDQ-NL and the M-ABC2-NL and the Early Years Movement Skill Checklist (EYMSC; Chambers & Sugden, 2006).

**Results:** The internal consistency of the Little DCDQ-NL calculated by Cronbach’s alpha was .926. The factor analyses resulted in three factor solutions explaining about 66% of the variation. The solution for the 3-4 year old age range included Factor I Fine motor control, Factor II Gross motor control, and Factor III Ball skills. The Little DCDQ-NL had a moderate correlation with the M-ABC2-NL (*r* = .551) and the EYMSC (*r* = -.626). The Little DCDQ-NL showed a significantly lower score in the at-risk group (<16th percentile in the M-ABC2) in comparison to the group with age typical development (p <.001). Most of the parents (62%) found the Little DCDQ-NL easy or really easy to fill out, a third found it just right, and 6% evaluated it as difficult to fill out.

**Discussion:** In sum, the current Dutch version of the Little DCDQ meets most of the necessary psychometric criteria of an early movement skills screening instrument. Based on the findings, this tool is able to discriminate between young typically developing children and children at risk of DCD.

**References**:

Rihtman, T., Wilson, B. N., & Parush, S. (2011). Development of the Little Developmental Coordination Disorder Questionnaire for preschoolers and preliminary evidence of its psychometric properties in Israel. *Research in Developmental Disabilities, 32*, 1378-1387.

**Keywords:** Developmental Coordination Disorder; Little DCDQ; Psychometrics.

### Daily motor characteristics in children with Developmental Coordination Disorder and in children with Specific Learning Disorder

B. Caravale^**1**^, S. Baldi^**2**^, F. Presaghi^**1**^, R. Penge^**3**^, I. Salvadore^**3**^ & M. Nunzi^**2**^

^1^Department of Developmental and Social Psychology, Sapienza University of Rome, Italy. barbara.caravale@uniroma1.it; ^2^Associazione Melograno Psicologia Cinica e Riabilitazione, Rome, Italy; ^3^Department of Paediatrics and Child Neuropsychiatry, Sapienza University of Rome, Italy?

**Aim:** An association between reading difficulties and coordination problems has been reported in several studies over the last decade. The aim of the present study was to investigate daily motor characteristics in two groups of children with developmental disorders, developmental coordination disorder (DCD) and specific learning disorder (SLD), compared to typically developing controls.

**Method:** 96 children participated in the study, 33 with diagnosis of DCD, 29 with diagnosis of SLD (excluding children with dysgraphia) and 34 typically developing children. The Italian version of ‘Developmental Coordination Disorder Questionnaire’ (Wilson *et al*., 2009 and Caravale* et al*., 2014) was used for the evaluation of the daily motor characteristics. DCDQ is a 15-item questionnaire designed to be self-administered by parents of children aged 5–15 years. Parents are asked to answer on a five-point Likert scale when comparing motor performance between their child and peers. The Total DCDQ scores and the three DCDQ sub-scores of the three groups of children were systematically compared in four distinct ANOVAs. When a significant main effect was found, the Tuckey Post-hoc tests were performed.

**Results:** The three groups compared with regard to DCDQ total showed significant differences (F(2, 93)=42.3; p <0.01) with the DCD group scoring significantly lower than both SLD and controls (both p<0.01). With regard to sub-scores, SLD reached significantly lower mean scores than controls in ‘Fine motor/hand writing’ skills and in the ‘General coordination’ area (both p<0.01) while they obtained significantly higher scores than DCD in ‘Control during movement’ and ‘General coordination’ areas (both p <0.01).

**Discussion:** Children with DCD showed more difficulties in daily motor performance compared to the other groups. Children with diagnosis of SLD diverged from typically developing children in general coordination skills and fine motor abilities. Our data suggest that although children with DCD present a more impaired global motor development, SLD may highlight difficulties in certain motor areas. These results are consisted with previous studies on motor difficulties in poor readers (Iversen *et al*., 2005) and encourage the routinely evaluation of motor skills in children with learning problems.

**References**:

Caravale, B., Baldi, S., Gasparini, C., & Wilson, B. N. (2014). Cross-cultural adaptation, reliability and predictive validity of the Italian version of Developmental Coordination Disorder Questionnaire (DCDQ). *European Journal of Paediatric Neurology, 18*(3), 267–272.

Iversen, S., Berg, K., Ellertsen, B., & Tønnessen, F. E. (2005). Motor coordination difficulties in a municipality group and in a clinical sample of poor readers. *Dyslexia, 11*(3), 217-231.

Wilson, B. N., Crawford, S. G., Green, D., Roberts, G., Aylott, A., & Kaplan, B. J. (2009). Psychometric properties of the revised developmental coordination disorder questionnaire. *Physical & Occupational Therapy in Pediatrics, 29*(2), 182–202.

**Keywords:** Parent questionnaire; Motor characteristics; DCD; Specific learning disorder.

### Little DCDQ-US in typically developing children and children with motor concerns with and without ASD

S.A. Cermak^**1**^ & A. Foran Jozjkowski^**2**^

^1^Department of Occupational Science and Occupational Therapy, University of Southern California, Los Angeles, California, USA. sharon.cermak@gmail.com; ^2^Department of Occupational Therapy, Towson University, Towson, MD, USA.

**Aim:** To examine whether there are differences between children with motor concerns with and without a diagnosis of ASD on the Little Developmental Coordination Disorder Questionnaire- US.

**Method:** Participants included children ages three and four years old, 85 children who were typically developing: 75 children who were receiving services that included motor concerns. The inclusion criteria for children with motor concerns included having gross motor or/and fine motor skills below average based on parent or therapist report and receiving one or more services of the following services related to motor needs: speech therapy, occupational therapy or physical therapy, psychological services. These children were not required to have a diagnosis of DCD; it was sufficient for children to have a general complaint of motor developmental problems. Children diagnosed with neurological or physical impairment such as cerebral palsy or muscular dystrophy, were excluded. Children with suspected or diagnosed ASD were not excluded. Of the 75 children with motor concerns, 27 parents reported that their child had a confirmed or suspected diagnosis of ASD. Parents completed the Little Developmental Developmental Coordination Disorder Questionnaire-US (LDCDQ-US), a 15 item questionnaire designed to screen young children at risk for DCD.

**Results:** There were significant differences between the group receiving services for motor concerns (n=75) and the typically developing group (n=85). For the total score, the three component scores (Control during movement; Fine motor: General coordination), and the individual items (all p<.001). Of the group with motor concerns, there were no significant differences between children with and without ASD.

**Discussion:** The Little DCDQ-US distinguishes between children with and without motor concerns but does not distinguish between children with motor concerns with and without ASD; both groups show impairments in motor performance based on parent-report. This finding is consistent with other investigators who have reported that children with ASD have motor concerns similar to children with DCD.

**Keywords:** Developmental Coordination Disorder; Autism Spectrum Disorder; Questionnaire.

### Motor impairment in children with Language Disorders: Influence of the language profile

Y. Chaix^**1,2**^, L. Beringer^**2**^, N. Faure Marie^**2**^, C. Chignac^**2**^, I. Barry^**2**^, C. Karsenty^**2**^ & J.-M. Albaret^**3**^

^1^Inserm; Imagerie Cérébrale et Handicaps Neurologiques UMR 825; CHU Purpan, Place du Dr Baylac, F-31059 Toulouse Cedex 9, France. chaix.y@chu-toulouse.fr; Université de Toulouse; UPS; Imagerie Cérébrale et Handicaps Neurologiques UMR 825; CHU Purpan; ^2^Unité de neurologie pédiatrie, Hôpital des Enfants, CHU Purpan, Toulouse, France; ^3^Université Toulouse III, UPS, PRISSMH EA 4561, Toulouse, France.

**Aim:** Recently the terminology of language problems in children has changed (DSM-5). Language Disorder (LD) is a broad and new category that does not differentiate between the pure expressive subtype and the mixed receptive-expressive subtype. Nevertheless these two subtypes have different outcomes that suggest two different entities. The aim of the current study was to compare motor impairment in children with pure expressive subtype or with mixed receptive-expressive subtype. The comorbidity with motor disorders could differentiate these two groups of LD and be an argument against this new classification.

**Method:** This was a retrospective monocentric study and we reviewed all records of children seen between January 2002 and December 2012 at the Reference Center for language and learning disabilities in Toulouse Children Hospital and diagnosed with Specific Language Impairment. We included all the children aged between 5 to 12y12 months and for which motor performances were assessed with the same version of M-ABC and the Purdue Pegboard test. We reported general cognitive level and more specifically FIQ and some subtests of Wechsler scales (vocabulary and block design). We have noted the presence of speech sound disorder (SSD) or not associated to the language disorder. Children with SSD only were excluded.

**Results:** 205 children have been included in this study with diagnosis of Specific Language Impairment: 92 with Pure Expressive LD and 113 with Mixed LD. We found difference on cognitive variables, with FIQ and score for vocabulary sub-test lower in Mixed LD compared to Pure Expressive LD group. Motor impairment (we have considered motor impairment when the total M-ABC score was < 5th percentile) was found in 45 % in Mixed LD group and in 31% in Pure Expressive LD group and the difference was statistically significant (p = 0.047). The total score for M-ABC was significantly higher in the Mixed LD group compared to the Pure Expressive LD group (10.51 versus 7.99; p=0.014) but only the sub test balance was different between the two groups (4.19 versus 2.61; p=0.003). No difference for manual dexterity (Purdue Pegboard test) was found between the two groups. The presence of speech sound disorder (SSD) with LD did not change the results.

**Discussion:** The two LD groups have different cognitive abilities and associated motor disorders: Mixed LD group had poorer IQ and more frequent and more severe motor impairment than Pure Expressive LD group. These findings suggest the existence of two different entities on a physiopathological point of view. The role of the cerebellum for the former group is possible due to the involvement of balance disorder.

**Keywords:** Motor impairment; Comorbidity; Language disorders.

### Motor impairment in children with Neurofibromatosis type 1: Effect of the comorbidity with the language disorders

Y. Chaix^**1,2**^, C. Chignac^**2**^, N. Faure Marie^**2**^, I. Barry^**2**^, S. Iannuzzi^**2**^, C. Karsenty^**2**^ & J.-M. Albaret^**3**^

^1^Inserm; Imagerie Cérébrale et Handicaps Neurologiques UMR 825; CHU Purpan, Place du Dr Baylac, F-31059 Toulouse Cedex 9, France. chaix.y@chu-toulouse.fr; Université de Toulouse; UPS; Imagerie Cérébrale et Handicaps Neurologiques UMR 825; CHU Purpan; ^2^Unité de neurologie pédiatrie, Hôpital des Enfants, CHU Purpan, Toulouse, France; ^3^Université Toulouse III, UPS, PRISSMH EA 4561, Toulouse, France.

**Aim:** There is a body of evidence demonstrating comorbidity of motor and cognitive deficit in « idiopathic » developmental disorders. These associations are also found in developmental disorders secondary to monogenic disorders as in Neurofibromatosis type 1 for which the principal complication during childhood is learning disabilities. The comparison of motor impairment between developmental disorders either idiopathic or secondary as in NF1 could help us to better understand the cause of the combined language/motor deficit in these populations. The current study investigated motor impairment in children with NF1 for which oral language had been specified and then to compare the motors skills of the NF1 group to motor performance of children with Specific Language Disorder (SLD).

**Method:** Children with a diagnosis of NF1 according to the conference consensus criteria, were included from the Toulouse Children Hospital Reference Center for NF1. Children were age between 5 to 12 years olds and motor performances were assessed with the M ABC and Purdue Pegboard tests. Cognitive general level, oral language and attentional skills were assessed with French standardized tests adapted for age. The SLD group was constituted with children diagnosed at the Reference Center for Language and Learning Disabilities of the same hospital. Children (NF1 and SLD groups) were matched for sex, age and IQ.

**Results:** 98 children have been included in this study with 49 children with NF1 and 49 children with SLD. In the NF1 group, 43% of children exhibited a Speech and/or language disorders. Motor impairment with a total M ABC score < 15th percentile was found in 53 % in NF1 group and in 33% in SLD group and the difference was statistically significant (p=0.041). Otherwise, the total score for M-ABC was significantly higher in the NF1 group compared to the SLD group (11.01 versus 8.21; p=0.046) but only the sub test balance was different between the two groups (5.18 versus 3.40; p=0.023). Nevertheless the comparison of motor scores of NF1 children with or without language impairment was not statistically different. Correlations studies show differences between the 2 groups. For NF1 Group, none correlation was found between motor scores and cognitive variables. Conversely, for SLD Group 1) significant negative correlations were found between total score for M-ABC and FSIQ (r=-0.30, p=0.035) and balance score M-ABC and Block design (r=-0.35, p=0.014) 2) significant positive correlation was found between Purdue Pegboard Right Hand and FSIQ (r=0.37, p=0.015).

**Discussion:** In NF1 group, motor impairment was more frequent and more severe and concerned specifically balance rather than manual dexterity or ball skills, compared to a group of children with SLD. This motor impairment was independent of language status in the NF1 group. These results and the difference between correlations according the groups could suggest the implication of different cerebral networks for motor impairment in these two conditions: the cortico-cerebellar network in NF1 and the cortico-striatal network in SLD. Nevertheless recent studies do not support an impaired cerebellar mechanism. Researches on procedural learning in children with NF1 could help to resolve these issues.

**Keywords:** Neurofibromatosis Type 1; Motor impairment; Comorbidity; Language disorders.

### Dual diagnosis of DCD and deaf children leading to intervention strategies

M.E. Chambers^**1**^, D.A. Sugden^**1**^, V.A. McQuillan^**1**^ & R.A. Swanwick^**1**^

^1^School of Education, University of Leeds, Leeds, LS2 9JT, UK. M.E.Chambers@education.leeds.ac.uk

**Aim:** Current thinking in the field of developmental disorders such as DCD is that not only are co-occurring characteristics important when diagnosing children but also that a dual diagnosis should not be ruled out. For the past two years we have been conducting research work on motor ability in deaf children and in particular how their motor skills impact on daily living. This is an under researched area compared to other developmental disorders (eg ASD; ADHD)and their relation to DCD. This paper draws upon case studies of children with a dual diagnosis of varying degrees of deafness and DCD and aims to illustrate how the dual diagnosis leads to different intervention strategies.

**Method:** The children have a current diagnosis of DCD peformed locally by occupational and physiotherapists, and in order to be consistent they have also been given both the Checklist and the Test from the Movement ABC-2 (Henderson, Sugden, & Barnett, 2007). In addition, parent and teacher interviews have been conducted and assessment of representational and non representational gestures have been adminstered. This assessment of gestures is to asceratain the hand functions of the children with respect to how they may learn sign language. All children have profiles constructed noting strengths and weaknesses, together with objectives and priorities for action. Each child profile shows a plan of action for a period of 10 weeks. The intervention is based on an ecological perspective and on the principle of the accumulation of marginal gains, now much employed in the sporting arena. This implies specific and specialist contributions from different individuals, parents, teachers, health professionals and friends, each one making small but significant contributions.

**Results:** The results show how the dual diagnosis affects the intervention strategies with each child working on different priorities, and how different individuals play their role.

**Discussion:** These results are discussed within the wider framework of an ecological approach to intervention involving an examination of both participation and learning variables.

**References**:

Henderson, S. E., Sugden, D. A., Barnett, A. (2007). Movement Assessment Battery for Children – Second Edition. London: Pearson.

**Keywords:** Dual diagnosis; DCD; Deaf; Intervention strategies.

### Phonological awareness and rapid automatic naming performance in preschool children with Developmental Coordination Disorder

H.-C. Cheng^**1**^, M.-L. Shen^**2**^ & R.-J. Cherng^**3**^

^1^Department of Physical Therapy, HungKuang University, Taichung, Taiwan. stpt@sunrise.hk.edu.tw; ^2^Department of Early Childhood Education, Asia University, Taichung, Taiwan; ^3^Department of Physical Therapy, National Cheng Kung University, Tainan, Taiwan.

**Aim:** Developmental Coordination Disorder (DCD) refers to a delay in motor development that does not have any known medical causes. Studies conducted in English speaking societies have found that children with DCD display a higher co-occurrence rate of learning difficulties (e.g., problems in reading and writing) than typically developing (TD) children. The purposes of this study were to examine the rapid automatic naming and phonological awareness ability of preschool children with DCD; and to examine the co-morbidity rate of cognitive deficit and motor impairment.

**Method:** Participants were recruited from 15 preschools at Taichung cities. They were first physically examined**,** received Developmental Coordination Disorder Questionnaire-Chinese version (DCDQ-C), attention-deficit/hyperactivity disorder test and measured with IQ test (C-TONI) and a language test. Then they were further measured with a motor test (Movement Assessment Battery for Children 2nd edition, Movement ABC 2) and categorized into two groups: DCD and TD based on the test results. A total of 692 children at the ages of 5-6 years participated in the study. The cognitive performance was examined by appropriate test and measurement (included rapid automatic naming test and phonological awareness test). Regression analysis that controlled for IQ showed that manual dexterity of M-ABC was predictive of all scores on the phonological awareness and rapid automatic naming tests. To determine a deficit on a test, a score at or below the 25th percentile of the norm or a score at or below 2 SD from the group mean was established as the cutoff.

**Results:** Sixty-two children (9.0%) were identified to have Developmental Coordination Disorder (DCD), and 630 children (91.0%) were without. Eighteen out of 62 children with DCD (29%) were noted to have co-morbid phonological awareness deficit. Sixteen to 29 children with DCD (26% to 47%) were noted to have either one or all subtypes of rapid automatic naming impairment. Chi-square analysis revealed a significant correlation between cognitive problem and DCD. The odds ratio of cognitive problem was higher (by about three-fold) among the children with DCD than among the children without (0.26 vs. 0.08).

**Discussion:** Co-morbid motor, phonological awareness and rapid automatic impairments in preschool children appear to be a significant clinical condition that requires the attention of the therapeutic community. Manual dexterity, in particular, seems to be an important clue for understanding the shared mechanism of motor and cognitive impairments.

**References**:

Asonitou, K., Koutsouki, D., Kourtessis, T., & Charitou, S. (2012). Motor and cognitive performance differences between children with and without developmental coordination disorder (DCD). *Research in Developmental Disabilities, 33(4),* 996 -1005*.*

Wolff, U. (2014). RAN as a predictor of reading skills, and vice versa: results from a randomized reading intervention. *Annals of Dyslexia, 64*, 15 -165.

**Keywords:** Phonological awareness; Rapid automatic naming; Co-morbidity; Developmental Coordination Disorder.

### The cognitive profile and reading and writing performance in children identified with Developmental Coordination Disorder at preschool age

H.-C. Cheng^1^, M.-L. Shen^2^ & R.-J. Cherng^3^

^1^Department of Physical Therapy, HungKuang University, Taichung, Taiwan. stpt@sunrise.hk.edu.tw; ^2^Department of Early Childhood Education, Asia University, Taichung, Taiwan; ^3^Department of Physical Therapy, National Cheng Kung University, Tainan, Taiwan.

**Aim:** Developmental coordination disorder (DCD) refers to a delay in motor skill development and learning that significantly interfere with children’s academic achievement and daily activities. Studies have found that DCD is not an isolated problem, but with a high risk of co-occurrence rate of cognitive processing deficit, such as phonological awareness and rapid automatic naming and further with reading and learning deficit. However, such information is obtained from studies done in the western countries but at Taiwan is unclear yet. Learning English and Chinese is very different. English is characterized with spelling but Chinese is orthographic. Therefore, the relationship of DCD and learning difficulties may not be the same in Chinese society as in English ones. The purposes of the study were: 1) to examine the cognitive profile (rapid automatic naming and phonological awareness ability) in preschool children with DCD; 2) to examine the co-occurrence rate of cognitive deficit (CD) and DCD; 3) to compare the reading and writing performance among children with DCD, children with CD, children with both CD and DCD and typically developing (TD) children when they get into primary school.

**Method:** Participants were recruited from preschools at Taichung cities. They first received physical examination, attention-deficit/hyperactivity disorder test, developmental coordination disorder questionnaire-Chinese version (DCDQ-C) and measured with IQ test, a language test. Then they were further measured with a motor test (Movement Assessment Battery for Children- 2nd edition, Movement ABC 2 and categorized into two groups: DCD and TD based on the test results. (2)The cognitive profile was examined with rapid automatic naming test and phonological awareness test. (3)At the first grade of elementary, the Chinese Reading Achievement Test (CRAT) and the Basic Reading and Writing Test Battery (BRWTB) were used to measure the children’s reading and writing performances.

**Results:** The participants were 61 DCD and 630 TD in preliminary study. The naming performance and phonological awareness ability in preschool children with DCD were significant lower than TD. The rate of phonological awareness deficit in children with DCD and TD were 29.5% and 14.1%, the digit, color and object naming deficit in children with DCD and TD were 25.8% and 8.2%, 40.3% and 16.8%, 46.8% and 17.9%. The co-occurrence rate of DCD and cognitive deficit in preschool children were higher than children with TD (72% & 33%). The children with CD showed significant poorer in the Chinese reading achievement test and the basic reading and writing test battery than TD children. The incidence of dyslexia in children with CD, children with DCD, children with both CD and DCD and TD children is 55.6%, 0%, 33.3% and 11.1% and 55.6%, 0%, 22.2% and 22.2% in writing difficult respectively.

**Discussion:** The children with DCD have high risk of co-occurrence rate of cognitive deficit (phonological awareness deficit and rapid naming deficit). Children with cognitive deficit will higher risk being dyslexia than children only with DCD or TD children. The cognitive performance is the predictive factor of reading disability and writing difficulty.

**References**:

Asonitou, K., Koutsouki, D., Kourtessis, T., & Charitou, S. (2012). Motor and cognitive performance differences between children with and without developmental coordination disorder (DCD). *Research in Developmental Disabilities, 33(4),* 996-1005*.*

Wolff, U. (2014). RAN as a predictor of reading skills, and vice versa: results from a randomised reading intervention. *Ann. of Dyslexia, 64*, 151–165.

**Keywords:** Developmental Coordination Disorder; Rapid automatic naming; Phonological awareness; Co-morbidity.

### Peripheral quantitative computed tomography (pQCT) reveals low bone mineral density in adolescents with motor difficulties

P. Chivers^**1**^, B. Hands^**1**^, F. McIntyre^**2**^, F. Bervenotti Filho^**1**^, T. Blee^**2**^, B. Beeson^**3**^, F. Bettenay^**3**^ & A. Siafarikas^**1,4**^

^1^Institute for Health Research, The University of Notre Dame Australia, Fremantle, W.A., 6959, Australia. paola.chivers@nd.edu.au; ^2^School of Health Sciences, The University of Notre Dame Australia, Fremantle, Australia; ^3^Department of Diagnostic Imaging, Princess Margaret Hospital, Subiaco, Australia; ^4^Department of Endocrinology and Diabetes, Princess Margaret Hospital, Subiaco, Australia.

**Aim:** Poor bone health is an emerging health risk in children and adolescents with possible long-term consequences due to an increased risk of osteoporosis, fractures and related complications in adulthood^1^. Little is known about bone strength and structure in adolescents with motor difficulties, yet low levels of physical activity and fitness are reported to be associated with poor bone strength and structure in those without motor difficulties. This study analysed local bone mineral density (BMD), assessed parameters of fracture risk (Stress Strain Index: SSI) and reported history of fractures in adolescents with motor difficulties.

**Method:** Thirty-three adolescents (20 males, 13 females), ranging in age from 12.5 years to 17.6 years (M=14.3 SD=1.5 years), with motor difficulties were included in the study. Motor performance was screened using the McCarron Assessment of Neuromuscular Development (MAND)^2^. Participants were eligible for the program if they had a Neuromuscular Development Index (NDI) of 85 or below (≤ 1SD) (mild motor disability) and/or a history of movement difficulties (such as poor coordination or clumsiness, slowness and inaccuracy of motor skills that negatively impact daily living, school, leisure and play activities^3^). Eligible participants underwent peripheral quantitative computed tomography (pQCT) measurements at proximal (66%) and distal (4%) sites of the non-dominant radius (R4 and R66) and tibia (T4 and T66). Results were compared to standardized norms using Z-scores for total BMD, trabecular density, cortical density and stress strain index (SSI) using one-sample t-tests.

**Results:** Significant differences were present at R4: BMD t=-6.96 (p<.001), R66: cortical density t=-2.16 (p=.038), R66: SSI t=5.32 (p<.001) and T66: SSI t=-4.02 (p<.001). There was a higher incidence of fractures (26.9%) compared to the normal population (3-9%) with more than half of caregivers identifying a tendency for their child to trip or fall more than their peers.

**Discussion:** This study extends previous pilot data, presenting local bone densiometric data based on pQCT analysis for adolescents with motor difficulties. We identified a previously unreported risk factor – low motor competence, for low BMD and fracture based on a detailed analysis of pQCT measurements including trabecular and cortical density. We found lower than average z-scores across most bone density measurements, which were more pronounced on the forearm, even when considering puberty and BMI. In addition, the radial and tibial SSI z-scores were in the low range indicating low bone strength and increased risk of fracture. Our results identified a higher incidence of reported fractures in this group compared to the normal population. We conclude that poor coordination should be considered a risk factor for below average bone strength and structure, and fracture risk. Strategies that improve bone health in this high-risk-group should be considered in intervention programs for developing motor competence.

**References**:

1. Ebeling, P. R., Daly, R. M., Kerr, D. A., & Kimlin, M. G. (2013). An evidence-informed strategy to prevent osteoporosis in Australia. *Medical Journal of Australia, 198*(2), 90-91.

2. McCarron, L. (1997). *McCarron Assessment of Neuromuscular Development* (3^rd^ ed.). Dallas, TX: McCarron-Dial Systems Inc.

3. American Psychiatric Association. (2013). *Diagnostic and statistical manual of mental disorders* (5th ed.). Arlington, VA: American Psychiatric Publishing.

**Keywords:** Adolescents; Motor difficulties; Bone health; pQCT; Fracture risk.

### Functional fitness can be improved and sustained over time in adolescents with DCD

P. Chivers^**1**^, F. McIntyre^**2**^ & B. Hands^**1**^

^1^Institute for Health Research, The University of Notre Dame Australia, Fremantle, W.A., 6959, Australia. paola.chivers@nd.edu.au; ^2^School of Health Sciences, The University of Notre Dame Australia, Fremantle, Australia

**Aim:** To determine if an individually designed fitness and strength program can improve functional fitness in adolescents with Developmental Coordination Disorder (DCD) and if these improvements can be sustained over time.

**Method:** Twenty adolescents identified with DCD based on the McCarron Assessment of Neuromuscular Development^1^ (15 males, 5 females), ranging in age from 11 years to 17 years (M=13.6 SD=1.5 years) participated in a 90 minute individualised exercise program twice a week (two 13 week programs per year) for up to four years. Performance on the Multistage Fitness Test, Curl-ups, Grip Strength and Standing Broad Jump were recorded pre and post each 13-week program along with anthropometric measures. Linear Mixed Modelling (LMM) was used to determine changes over time whilst controlling for age, gender, Neuromuscular Development Index (NDI), Body Mass Index (BMI), and the number of sessions attended. LMM has the advantage over repeated measures ANOVA in that they are more flexible in fitting and testing covariance structures, provide the opportunity to investigate within-person and between-person change over time, permit individuals to have missing data points, and allow the inclusion of time-varying measures^2^.

**Results:** Participants attended on average 21 sessions (SD=3) and were involved in an average of 6 (SD=4) 13-week program cycles over the reported period. Only significant model predictors are reported for the LMM. Multistage Fitness Test performance improved over time (p<.001), was higher with increasing NDI (p<.001), increasing age (p=.004), and with decreasing BMI. Males reported higher Multistage Fitness Test scores compared to females (p=.004). Curl up performance improved over time (p<.001) and with increased adherence to the 13-week program (p=.024) and higher NDI (p<.001), with females out performing males (p<.001). Grip strength performance also improved over time (p<.001), was higher with increasing BMI (p<.001), increasing NDI (p<.001) and increasing age (p<.001). Standing Broad Jump performance did not improve over time, with performance scores higher with increasing NDI (p<.001), increasing age (p<.001) and decreasing BMI (p=.001).

**Discussion:** The LMM provided the opportunity to investigate within-person and between-person change over time, controlling for differences in the number of sessions attended. This study found that an individually targeted exercise program can achieve sustainable improvement in fitness outcomes in adolescents with DCD. Importantly, sustained participation over time (total sessions attended) is more important than specific program adherence.

**References**:

1. McCarron, L. (1997). *McCarron Assessment of Neuromuscular Development* (3^rd^ ed.). Dallas, TX: McCarron-Dial Systems Inc.

2. West, B. T., Welch, K. B., & Galecki, A. T. (2007). *Linear mixed models. A practical guide using statistical software*. Boca Raton, FL: Chapman.

**Keywords:** Adolescents; Motor difficulties; Fitness; Longitudinal study; Exercise intervention.

### The influence of support surface rigidity and visual information on postural control in children with Developmental Coordination Disorder

H.C. Chung^**1**^

^1^Department of Physical Education. Kunsan National University, Gunsan-si, Jellabook-do, 573-701, Republic of Korea. hcx@kunsan.ac.kr

**Aim:** Little is known about the dynamics of perceptual motor coupling in postural control of DCD children to use dynamic visual information (known as optic flow) of the perception and control of stance. Previous study^1^ has shown that DCD children exhibit robust postural responses to optic flow in a moving room. The study further investigated the differential sensitivity of TD and DCD children to imposed optic flow using the moving room paradigm. Using the moving room paradigm, we evaluated the dynamic of postural response induced by cyclic oscillation of visual and cutaneous stimulus for children with DCD compared to typically developing children.

**Method:** A total of twenty children, ten with DCD (< 5th percentile in M-ABC test) and ten matched controls, in the age of 10-11 years (mean age = 9.5 ± 0.6 yr) participated in this study. We evaluated the postural sway of TD and DCD children while providing and reducing sensitivity of support surface with the vision or without during the cyclic oscillation of visual stimulus. Participants were asked to maintain erect posture (standing on a force plate) while facing along the axis of room oscillation. The room was oscillated with magnitude 2 cm at three different frequencies (0.1 Hz, 0.2 Hz, 0.3 Hz). Variation in the frequency of room oscillation was crossed with variation in the support surface (foam vs. no foam), for both TD and DCD children. We measured the motion of the body in the anterior-posterior axis of center of pressure (COPap), that is, along the line of sight.

**Results:** The postural sway of DCD children was less stable than TD children with eyes closed without room motion, *F (1, 19)* =* 6,85, p*<*.01*. We found a significant interaction between frequency and supports surface on the coherence of COP motion relative to room motion, *F(1,22*)= 5.91, *p* < .05. In addition, there was a significant 3 way interaction between group, frequency, and supports surface on room-COP gain, *F(1,22*)= 3.40, *p* < .05.

**Discussion:** These results revealed that the responses of DCD children to impose optic flow while standing supports surface qualitatively different from the responses of TD children. We concluded that DCD children may have problem in integrating multi-sensory information and use of optic flow differently from TD in controlling their posture.

**References**:

*1.* Chung, H.C. & Stoffregen, T.A. (2011). Postural responses to a moving room in children with and without developmental coordination disabilities.* Research in Developmental Disabilities,* 2011, *32*(6), 2571-2576.

**Keywords:** Postural control; Development Coordination Disorder; Moving room; imposed optic flow.

### The extent and nature of motor dysfunction based on age, socio-economic status, gender and race of a selected group of 3- to 5-year old children

D. Coetzee^**1**^, A.E. Pienaar^**1**^ & A. Venter^**1**^

^1^Physical activity, Sport and Recreation (PHASRec) focus area, Faculty of Health Science, Potchefstroom campus, North West University, South Africa. 12129941@nwu.ac.za

**Aim:** The aim of this study was to establish the nature and extent of motor dysfunction based on socio-economic status, gender and race in 3- to 5-year old children.

**Method:** A convenience sample of 53 participants aged 3.0-4.11 years from 5 kindergarten schools from different Quintile 1 – Quintile 5 schools (1 = low socio-economic and 5 = high socio-economic) in the Potchefstroom area, North West Province in South Africa, were assessed with the Movement Assessment Battery for Children-2.

**Results:** A percentage of 11.32% of the group (5 girls, 1 boy) were classified with severe DCD. These results indicated that the high socio-economic class (22.73%), girls (15.63%), black children (18.18%) and the 3-year old group (12.50%) had the most toddlers in the severe DCD group. The 3.0 year group performed significantly better (p≤0.05) than the 4.0 year group in catching and throwing. White children outperformed black children in fine motor skills and boys outperformed (p≤0.05) girls in catching and throwing.

**Discussion:** No statistical significant differences were found between the different socio-economic groups. These results confirm motor dysfunction problems in 3- to 5-year old children with gender and age differences to be considered when addressing these problems.

**Keywords:** Preschool children; Motor development; Developmental Coordination Disorder; Socio-economic status; Gender.

### The relationship between visual motion sensitivity and motor competency: the importance of group selection

F. Corbett^**1**^, J. Atkinson^**1,2**^ & O. Braddick^**2**^

^1^Department of Developmental Science, University College London, Gower Street, London, WC1E 6BT, United Kingdom. fleur.corbett.10@ucl.ac.uk; ^2^Depatment of Experimental Psychology, University of Oxford, Oxford, United Kingdom.

**Aim:** Global visual motion sensitivity is crucial for decoding optic flow to successfully navigate the environment. Across several developmental disorders, reduced visual motion sensitivity and poor motor competency have been found. However, there are mixed findings regarding the status of visual motion sensitivity in DCD^1,2,3^ and whilst a meta-analysis^4^ identified visual perceptual impairments in DCD, their contribution to symptomatology is unclear. The current study sought to examine the relationship between visual motion sensitivity and motor competency.

**Method:** Motor and verbal abilities of 6-14 year olds (n=85) from two primary schools were assessed with the Movement ABC-2 & Checklist and BPVS-2. All participants had normal or corrected-to-normal vision and were free from developmental disorders. Sensitivity to radial, rotational and translational motions as well as an equivalent static pattern was measured separately using random dot kinematograms with a 2AFC psychophysical task. Participants had to detect the location of a coherent motion or pattern embedded in noise, with coherence level varied across trials to determine sensitivity.

**Results:** Global visual motion sensitivity was significantly poorer than static pattern sensitivity (p<0.001) but could not predict motor competency in this typically developing sample (p>0.500). Verbal ability was the only reliable predictor of motor competency (p=0.005). When the sample was divided according to Movement ABC-2 score, no difference in motion or static pattern sensitivity was found (p=0.789) for high (n=14) and low (n=14) motor ability groups, matched for age and verbal ability.

**Discussion:** Whilst a longer maturation period and reduced sensitivity were found for visual motion, there was no evidence of a relationship between visual motion sensitivity and motor competency in this typically developing sample. As verbal ability could predict motor competency, this raises the concern that relationships found previously^2^ may have been mediated by between-group differences unrelated to DCD. To assess visual motion sensitivity and its contribution to motor competency in DCD, we are currently using novel visuomotor tasks in individuals diagnosed with DCD with a range of mental ability.

**References**:

1. O’Brien, J., Spencer, J., Atkinson, J., Braddick, O. & Wattam-Bell, J. (2002). Form and motion coherence processing in dyspraxia: evidence of a global spatial processing deficit. *NeuroReport, 13*, 1399-1402.

2. Sigmundsson, H., Hansen, P.C. & Talcott, J.B. (2003). Do ‘clumsy’ children have visual deficits. *Behavioural Brain Research, 139*, 123-129.

3. Wilmut, K. & Wann, J. (2008). The use of predictive information is impaired in the actions of children and young adults with Developmental Coordination Disorder. *Experimental Brain Research, 191*, 403-418.

4. Wilson, P.H., Ruddock, S., Smits-Engelsman, B., Polatajko, H. & Blank, R. (2012). Understanding performance deficits in developmental coordination disorder: a meta-analysis of recent research. *Developmental Medicine & Child Neurology,* *55*, 217-228.

**Keywords:** Visual motion sensitivity; Group matching.

### Nature and specificity of gestural disorder in children with Developmental Coordination Disorder: a multiple case study

O. Costini^**1,2**^, A. Roy^**1,3,4**^, S. Faure^**5**^, C. Remigereau^**1,3**^ & D. Le Gall^**1,6**^

^1^Laboratory of Psychology UPRES EA4638, University of Angers, LUNAM, Angers, 49045, France. orianne.costini@gmail.com; ^2^Pediatric Unit for Learning Disabilities, University Hospital of Nice, France; ^3^Reference Center for Learning Disabilities, University Hospital of Nantes, France; ^4^Neurofibromatosis Clinic, University Hospital of Nantes, France; ^5^University of Nice-Sophia Antipolis, EA7278, Nice, France; ^6^Neuropsychology Unit, Department of Neurology, University Hospital of Angers, France.

**Aim:** Praxis skills are commonly defined as the ability to perform purposeful motor actions and to use tools. The lack of theoretical framework to explore praxis in children has been problematic for clinical/experimental tasks design and consequently for understanding the underlying deficits to gestural difficulties. Although praxis assessment in developmental coordination disorder (DCD) children mostly used adult’s clinical tests, including comparisons across types of gestures and input modalities, the cognitive models of adult praxis processing are rarely used in a comprehensive interpretation. These models^1,2^ generally involve two systems: a conceptual system (semantic knowledge about tool function or about actions, and sensorimotor knowledge about manipulation), and a production system (execution of a gesture). Whereas heterogeneity of deficits is consistently reported, few researches have investigated the implication of other cognitive skills assumed to be involved in DCD, such as executive or visual-perceptual and visuospatial functions^3,4^. Our study aimed at discussing the nature and specificity of the gestural deficit in children with DCD using a multiple case study approach.

**Method:** We elaborated a comprehensive assessment of gestures that allows exploring distinct levels hypothesized by adult models of praxis processing. We also examined constructional abilities, executive functions, visual perception, and visuospatial processing. We recruited 27 children diagnosed with DCD by Pediatric Wards of University Hospitals, based on a multidisciplinary assessment following DSM-IV-R criteria. Neuropsychological profiles were classified using an inferential clinical analysis based on the modified t-test^5^, and compared with 100 typically developing children divided into 5 age groups.

**Results:** Among the 27 DCD children, we first classified profiles that are characterized by impairment in tasks assessing perceptual visual or visuospatial skills (n = 8). Children with a weakness in executive functions (n = 6) were then identified, followed by those with an impaired performance in conceptual knowledge tasks (n = 4). Among the 9 remaining children, 6 could be classified as having a visual spatial/visual constructional dyspraxia as proposed by Vaivre-Douret et al. (2011). Gestural production deficits were variable between and within profiles.

**Discussion:** This study confirmed the heterogeneity of gestural production deficit among children with a diagnosis of DCD, at both intra- and inter-individual levels. The contribution of other cognitive deficits in most of the profiles allows discussing the specificity of gestural difficulties, and the current conception of praxis in children.

**References**:

1. Rothi, L. J. G., Ochipa, C., & Heilman, K. M. (1991). A cognitive neuropsychological model of limb praxis. *Cognitive Neuropsychology, 8,* 443–458.

2. Roy, E. A., & Square, P. A. (1985). Common considerations in the study of limb, verbal and oral apraxia. In E. A. Roy (Ed.), *Neuropsychological studies of apraxia and related disorders* (pp. 111–161). Amsterdam: Elsevier.

3. Rahimi-Golkhandan, S., Piek, J. P., Steenbergen, B., & Wilson, P. H. (2014). Hot executive function in children with Developmental Coordination Disorder: Evidence for heightened sensitivity to immediate reward. *Cognitive Development, 32*, 23-37.

4. Tsai, C.-L., Wilson, P. H., & Wu, S. K. (2008). Role of visual-perceptual skills (non-motor) in children with developmental coordination disorder. *Human Movement Science, 27*(4), 649–664.

5. Crawford, J. R., & Howell, D. C. (1998). Comparing an Individual’s Test Score Against Norms Derived from Small Samples. *The Clinical Neuropsychologist (Neuropsychology, Development and Cognition: Section D), 12*(4), 482–486.

6. Vaivre-Douret, L., Lalanne, C., Ingster-Moati, I., Boddaert, N., Cabrol, D., Dufier, J.-L., … Falissard, B. (2011). Subtypes of developmental coordination disorder: research on their nature and etiology. *Developmental Neuropsychology, 36*(5), 614–43.

**Keywords:** Praxis; Gestures; Developmental Coordination Disorder; Specificity; Dyspraxia.

### Movement adaptation strategies in an unpredictable environment: A comparison between individuals with and without Developmental Coordination Disorder (DCD)

W. Du, K. Wilmut & A.L. Barnett

Perception and Motion Analysis (PuMA) Lab, Department of Psychology, Social Work and Public Health, Oxford Brookes University, UK. wdu@brookes.ac.uk

**Aim:** The ability to locomote through the environment without falling or bumping into objects is a fundamental everyday skill, which is typically assessed using static obstacles in the lab. In everyday life, however, we are often faced with a dynamic environment, such as a busy street, where obstacles appear and move across our path in an unpredictable fashion. This is usually performed effortlessly but actually involves complex skills to visually perceive the obstacle and adapt our own body movements. For individuals with Developmental Coordination Disorder (DCD), who are reported to find adjustments to ongoing movements difficult, such an environment presents a real challenge and can have a negative impact on safe participation in daily life activities. The aim of the current study was therefore to determine whether movement adaptations in an unpredictable environment differ in individuals with DCD compared to typically developing individuals.

**Method:** A new paradigm was developed for this study, to create a simple yet unpredictable environment to be negotiated. 15 adults with DCD (assessed in line with DSM-5 criteria) and 15 age and gender matched controls walked along a 10m walkway. On some trials a ‘gate’ closed in front of them blocking part of the pathway. Reflective markers placed on the trunk and feet were tracked with a VICON motion analysis system. Spatial and temporal characteristics of their movements were collected over the approach phase, and while they walked around the obstacle. In ‘gate closed’ trails, the number of adjustments to step width and step length were calculated to determine the individual strategies employed to negotiate the obstacle in the pathway.

**Results:** Individuals with DCD employed different types of adaptive strategies compared to the control group. Effects of group will also be reported in terms of approach speed, timing of the decision to alter the walking path, the type of adaptive strategies employed, and the distance maintained from the gate.

**Discussion:** The different adaptive strategies of individuals with DCD might reflect their poor coordination skills. These findings will provide a better understanding of the navigation patterns in individuals with DCD in a dynamic environment.

**Keywords:** Action capabilities; Locomotion; Obstacle avoidance; Movement adaptation.

### Dual diagnosis: Autism Spectrum Disorder & Developmental Coordination Disorder

C. Dunstan

Owner of Dunstan Pediatric Services, Plymouth, NH, 03264, United States of America. chris@dunstanpediatric.com

**Aim:** This research will explore the differentiating sensory-motor movements of a single case study with the dual diagnosis of autism Spectrum Disorder (ASD) and Developmental Coordination Disorder (DCD). The quality of movements will be articulated in catogories of praxis versus dyspraxic movements influenced by a DCD.

**Method:** Three students were selected for this research, all of whom retain an ASD. The case study holds a secondary diagnosis of DCD. The two children without a DCD were selected based on their ability to evoke the praxis of movement. Video samples of each child engaged in novel motor skills will be used to identify the intensity, discovery, and fluency of movement patterns. Using a likert scale to qualify movements, neurodevelomental immaturies will be identified and valued for the impact on functional performance.

**Results:** A the time of this abstract submission, the results are not fully discovered.

**Discussion:** This simple attempt to search for and identify sensory motor movement patterns can be valuable to the therapist supporting a child’s developmental and functional needs. By identifying variations in the quality of movements from a child with a DCD with the uninterested nature of the child with an ASD, therapy services have the opportunity to provide more focued interventions and realistic expectations for students with this dual diagnosis.

**References**:

American Psychiatric Association (APA) (2013). *DSM-5: Diagnostic and statistical manual of mental disorders* (5^th^ ed.). Washington, DC: American Psychiatric Association.

**Keywords:** Developmental Coordination Disorder; Autism Spectrum Disorder; Sensory-motor movements; Praxis; Dyspraxic.

### Importance of Developmental Coordination Disorder definitions: Evolution from DSM-IV to DSM-5

M. Farmer^**1,2**^, B. Échenne^**2**^ & M. Bentourkia^**1**^

^1^Department of Nuclear Medicine and Radiobiology, and ^2^Department of Paediatrics, Faculty of Medicine and Health Sciences, Sherbrooke (Qc), J1H 5N4, Canada. Marie.Farmer@USherbrooke.ca

**Aim:** Since 1987, Developmental Coordination Disorder (DCD) is reported and defined in the DSM (Diagnostic and Statistical Manual of Mental Disorders) as a neurodevelopmental disorder present in 5-6 % of the school-age population, preferentially affecting boys. Several publications on DCD contributed to expand the definition of DCD to shape the latest edition of the DSM, DSM-5.

**Method:** Based on the literature and on the definitions in DSM-IV and DSM-5, we discuss in this work the characteristic of slowness in DCD children.

**Results:** Two aspects now appear as part of DCD: “slowness and inaccuracy of motor skills (e.g., catching an object, using scissors or cutlery, handwriting, riding a bike, or participating in sports)”, sometimes isolated among DCD subjects. These are cited in the first paragraph of the definition and allows the inclusion of subjects with relatively simple measures of school facilities who are able to compensate for their difficulties. Furthermore, the previous DCD definition was inherently referring to a school-age population. Adding in the former a sentence concerning “professional” activities (prevocational and vocational activities) now implies that DCD persists over time and is also found in adult subjects.

**Discussion:** Several publications have described the delays encountered in DCD characteristics as well as the persistence of the disorder in adulthood. DSM is a reference tool, and for DCD, changes to the definition contribute to better recognition and management of the disorder.

**Keywords:** Developmental Coordination Disorder; DSM-IV; DSM-5; Paediatrics; Brain development.

### Phenotype selection in DCD patients prior to quantitative PET imaging

M. Farmer^**1,2**^, B. Échenne^**1**^, M. Bentourkia^**1**^

^1^Department of Nuclear Medicine and Radiobiology, Faculty of Medicine and Health Sciences, Sherbrooke (Qc), J1H 5N4, Canada. Marie.Farmer@USherbrooke.ca; ^2^Department of Paediatrics, Faculty of Medicine and Health Sciences, Sherbrooke Canada.

**Aim:** The aim of the study was to propose a means to select a cohort of patients affected by Developmental Coordination Disorder (DCD) based on their phenotypes.

**Method:** The study was carried out in 129 paediatric patients addressed to our outpatient 3rd line clinic dedicated to DCD. A questionnaire with 33 items concerning the developmental stages, education, social interactions and specific clinical examination was developed according to an exhaustive review of the literature. This questionnaire was administered to each new patient. To confirm DCD diagnoses, complementary measurements were performed with magnetic resonance imaging, electroencephalography and other standard psychological tests.

**Results:** Among the 200 patients, 129 were confirmed having DCD including 47% who were treated for Attention Deficit Hyperactivity Disorder, in conformation with DSM-5 definitions. In these DCD subjects, among other results, 100 % had a slow implementation of actions in requested tasks, 47% were left-handed or ambidextrous, 76% were male, 95% showed handwriting difficulties, 56% moved in a sitting position before acquiring normal walking.

**Discussion:** From this study and in an attempt to homogenize groups of patients for further experimental procedures, we classified our DCD subjects into 3 categories: 1) slow and clumsy; 2) slow without awkwardness; and 3) slow with “language impairment” (verbal dyspraxia and/or oral -facial dyspraxia).

**Keywords:** Developmental Coordination Disorder; DSM-5; Paediatrics; Brain development.

### Motor impairment in children with specific language impairment: Effect of comorbidity on school outcomes

N. Faure Marie^**1**^, I. Barry^**1**^, C. Chignac^**1**^, N. Lafin^**1**^, C. Karsenty^**1**^ & Y. Chaix^**1,2**^

^1^ Unité de neurologie pédiatrie, Hôpital des Enfants, CHU Purpan, 330, avenue de Grande-Bretagne, 31059 Toulouse cedex 9, France. faure-marie.n@chu-toulouse.fr; ^2^Inserm, Imagerie Cérébrale et Handicaps Neurologiques UMR 825, CHU Purpan, Toulouse, France.

**Aim:** Similarly to specific language impairment (SLI), developmental coordination disorder (DCD) is a disability that has long-term consequences on child’s learning, development and socialization. The combination of these two disorders is frequent, and varies between 30 and 90 % according to the authors. The comorbidity has a negative impact on school outcomes, and subsequently restricts the choice of professional activities. The aim of this study is to assess whether children with both DCD and SLI experience more difficulties with education, behavior and self-esteem than children with isolated SLI.

**Method:** In this retrospective study, we contacted 32 children who attended the language school of the Reference Center for language and learning disabilities in Toulouse Children Hospital between 2003 and 2014. At the time of the assessment, these children were between 6 and 18 years old. Of all the children with SLI (assessed by French standardized tests such as TCG, Ecosse, NEEL, O52), 12 (37%) additionally have a DCD diagnosed with the M-ABC test (score < 15^e^ percentile) and with the impact on the daily living estimate at the time of discussions with the parents. Three questionnaires were sent, covering repectively generalities, schooling and activities. The psycho-behavior profile was evaluated using the Child Behavior Check List (CBCL). Self-esteem was assessed using the Echelle Toulousaine d’Estime de Soi (ETES).

**Results and discussion**: Data collection and analysis are in progress. At the moment 27 participants, 9 of whom are girls, completed the first questionnaire. A third of the children were sent to a specific education school (CESSDA) after the language school and 74,1% have repeated a grade. For the other questionnaires, we hypothesise that the psycho-behavior profile and the self-esteem will differ between the two groups (SLI, SLI+DCD). The disorder consequences should be more pronounced on internalizing clinical profiles and ‘physical’ self-esteem for the SLI+DCD group.

**Keywords:** Developmental Coordination Disorder; Comorbidity; Specific language impairment; Education; Psycho-behaviour; Self-esteem.

### Modeling executive functioning by a dimensional approach in rodents: toward an animal model of DCD?

A. Fitoussi, P. Renault, C. Le Moine, E. Coutureau, C. Blondeau, M. Cador & F. Dellu-Hagedorn

Institut des Neurosciences Cognitives et Intégratives d’Aquitaine (INCIA), UMR 5287 CNRS, F-33000 Bordeaux, France. francoise.dellu@u-bordeaux.fr

**Aim:** A major challenge in biomedical psychiatric research is to model the main symptoms of mental disorders in animal models with good construct and predictive validities. The heterogeneous nature of the Developmental Coordination Disorder (DCD) and the absence of established pathophysiological etiology are many obstacles for establishing such models. However, animal models of DCD and related comorbid troubles, like ADHD remain necessary for investigating easily and rapidly the related neurochemical, neuropathological and genetical alterations and to develop new therapeutic strategies. A promising approach, based on natural behavioral inter-individual differences, consists in identifying within a « normal » population, some individuals with extreme or atypical behaviors that mimic the symptoms of the disorder for identifying causal factors. This approach presents the advantage to avoid preconceived etiological hypothesis. It is based on a dimensional vision of the trouble and considers that most of disorders related to executive dysfunction are an extreme manifestation of a continuum including unimpaired individuals.

**Method:** Based on this approach, we have explored the neural bases of executive dysfunction (attention^1^ or working memory deficits^2^, impaired inhibition^3^, poor decision-making^4,5^) and more recently, we focused on one particular symptom of DCD related to the impaired ability to process the transitions between goal-directed and automated motor skill. This process can be addressed in rats through the capacity to adapt actions in instrumental conditioning, when decreasing the contingency between the action and earning the outcome. We explored brain activity within the fronto-striatal network using immunodetection of Zif268 protein.

**Results:** Using the contingency degradation paradigm, we showed large inter-individual differences in goal-directed behavior, some rats demonstrating impaired capacity to adapt to the environmental changes. We showed that these inter-individual differences were related to the balance of activity between the medio prefrontal cortex and the dorsal striatum.

**Discussion:** These findings indicate that a rat model is suitable to assess differences in the ability to adapt motor skills to changes in environmental contingencies. This model allows identifying the specific neural network involved within the fronto-striatal circuitry. Further investigations will be necessary to establish a valid animal model of DCD, notably by combining several other symptoms and comorbid troubles, like deficits in timing, inhibition, working memory or attention.

**References**:

1. Blondeau, C., & Dellu-Hagedorn, F. (2007). Dimensional analysis of ADHD subtypes in rats. *Biological Psychiatry, 61*(12), 1340-1350.

2. Grégoire, S., Rivalan, M., Le Moine, C., & Dellu-Hagedorn, F. (2012). The synergy of working memory and inhibitory control: behavioral, pharmacological and neural functional evidences. *Neurobiology of Learning and Memory, 97*(2), 202-212.

3. Rivalan, M., Grégoire, S., & Dellu-Hagedorn, F. (2007). Reduction of impulsivity with amphetamine in an appetitive fixed consecutive number schedule with cue for optimal performance in rats. *Psychopharmacology (Berl), 192*(2), 171-182.

4. Rivalan, M., Ahmed, S. H., & Dellu-Hagedorn, F. (2009). Risk-prone individuals prefer the wrong options on a rat version of the iowa gambling task. *Biological Psychiatry, 66*(8), 743-749.

5. Fitoussi, A., Le Moine, C., De Deurwaerdere, P., Laqui, M., Rivalan, M., Cador, M. et al. (2014). Prefronto-subcortical imbalance characterizes poor decision-making: neurochemical and neural functional evidences in rats. *Brain Structure and Function,* (in press).

**Keywords:** Animal model; Executive functions; Inter-individual differences; Goal-directed behaviour; Prefronto-striatal network.

### Impact of self-movement generation on the discrimination of human body posture: a perception-action coupling study in children from 7 to 10 years old

A. Fontan, F. Cignetti, M. Vaugoyeau & C. Assaiante

Laboratoire de Neuroscience Cognitive, Aix-Marseille Université, CNRS, LNC UMR 7291, 13331 Marseille. aurelie.fontan@etu.univ-amu.fr

**Aim:** Body schema is an internal representation of the body in action. As executing and perceiving human movements share common representations, the body schema could be involved in the ability to perceive and understand other’s action. Its construction during ontogenesis is based on sensory inputs. An early sensitivity to biological aspects of movement was reported in infants, as well as a robustness of the perception of human movement during adolescence^1^. Also, many studies based on motor planning and execution during development confirm the late maturation of multisensory integration for central motor control^2^. The aim of this study is to evaluate the body schema construction during childhood via the functional link between the sensory representations activated during action perception and the motor representations used during action execution.

**Method:** 20 adults (28.9±7.4 y; 10♀) and 20 TD children from 7 to 10 years participated to this study. The task consisted in a sequential “same-different” visual matching task^3^. Two types of stimuli were presented: pictures of bodies’ posture and Lego’s shapes. In both cases, the stimulus was presented first in a front view and secondly with a rotation of 45° with or without modification in the body or object configuration. While viewed the second stimulus, participants had to decide whether its shape was identical or different as quickly and accurately as possible. During this task participants had to execute unrestrictive movements or to imitate the human posture. A control condition for both stimuli without movement was also executed. All conditions were presented in blocks randomly counter-balanced between participants. Reaction time and response accuracy were recorded.

**Results:** Whatever the conditions, adults presented a high-level of body perception performances with significantly higher correct answers in human posture than in object. Preliminary results in children showed that specificity to detect human posture variation is already present at 7 years.

**Discussion:** This finding supports that posture recognition is specifically subtended by the body schema. By contrast with adults, movement involvement during posture discrimination is expected to influence children performances. These findings would provide a simple tool for better understanding perception-action coupling and body schema construction in individuals with DCD.

**References**:

1. Cignetti, F., Caudron, S., Vaugoyeau, M., Assaiante, C. (2013). Body schema disturbance in adolescence: From proprioceptive integration to the perception of human movement. *Journal of Motor Learning and Development, 1*, 49-58.

2. Assaiante, C., Cignetti, F., Fontan, A., Nazarian, B., Roth, M., Anton, J. L., Coulon, O., & Vaugoyeau, M. (2014). *Developmental changes in the cerebral network of proprioceptive processing from adolescent to adulthood*. Poster Neuroscience, Washington.

3. Reed, C. L., & Farah, M. J. (1995). The psychological reality of the body schema: A test with normal participants. *Journal of Experimental Psychology: Human Perception and Performance, 21*(2), 334-343.

**Keywords:** Body perception; Body schema; Perception-action coupling; Children.

### Motor coordination and attentional problems in school-aged children in a low-income population from northeastern Brazil

A.S. Franca^**1**^, R. Matias^**1**^, M.H.C. Real^**1**^, A.D.L. Vale^**1**^, M.A.V. Pacheco^**1**^, J.P.D. Clase^**1**^, O.S. Agostini^**2**^ & C.R.S. Araujo^**1**^

^1^Department of Occupational Therapy, Health Sciences Center, Federal University of Paraiba, Campus I, s/n - Castelo Branco, João Pessoa, PB, 58051-900, Brazil. clariceribeiro@hotmail.com; ^2^Department of Occupational Therapy, Federal University of Rio de Janeiro, Brazil.

**Aim:** To investigate the prevalence of children with motor problems with 7 and 8 years old attending public schools of João Pessoa/PB - Northeastern Brazil.

**Method:** A total of 535 children were assessed with a questionnaire of the history of child’s development, the Developmental Coordination Disorder Questionnaire (DCDQ), the Economic Classification Questionnaire Criterion Brazil - to investigate the economic profile of families - and the Swanson, Nolan and Pelham Scale IV - SNAP-IV. The descriptive data analysis was performed using the Statistical Package for Social Sciences 18.0 (SPSS).

**Results:** In total, 47.2% of children were identified with motor problems. According to the analysis of the Economic Classification Criterion Brazil, 79% of these families were classified in economic classes C, D and E which means that they live with an income of $500 or less per month; 23% showed signs of inattention, hyperactivity, or both, associated with motor problems; 5% were born premature according to data collected in the child’s notebook – a commonly used instrument in primary health care to monitor child’s growth and development.

**Discussion:** Prevalence of motor problems are reported to be between 5 and 19% in the literature, but the present study found that 47.2% of children received scores indicative of motor problems/possible TDC, according to the cutoff points from DCDQ used in other studies, including in Brazil. Seventy nine percent of the families are among the economic level classes C, D and E, whose purchasing power is low. More studies should be done to investigate the environmental and social contexts these children live. It is extremely important to carry out a more thorough assessment of these children with motor performance and sustained attention tests, as indicated in the DSM-V to confirm how many of these children are in fact DCD. Another point to be raised is the possibility of difficulty in understanding the DCDQ-Brazil test, which requires caregivers compare your child with another of the same age and give you a score for your performance on certain tasks. As these children are identified earlier, it can be developed school programs, reducing the damage caused in these children’s daily life.

**References**:

Cairney, J., Faught, B. E., Hay, J. A., Corna, L. M., & Flouris, A. D. (2006). Developmental Coordination Disorder, age and play: a test of the divergence in activity-deficit with age hypothesis. *Adapted Physical Activity Quarterly, 23*, 261-276.

Cardoso, A. A., & Magalhães, L. C. (2012). Análise da validade de critério da Avaliação da Coordenação e Destreza Motora – ACOORDEM para crianças de 7 e 8 anos de idade. *Revista Brasileira de Fisioterapia, 16*(1), 16-22.

França, C. de. (2008). *Desordem Coordenativa Desenvolvimental em Criança de 7 e 8 anos de Idade*. Dissertação de Mestrado, Universidade do Estado de Santa Catarina, Florianópolis, SC, Brasil.

Kirby, A., Sugden, D., & Purcell, C. (2014). Diagnosing developmental coordination disorders. *Archives of Disease in Childhood, 99*(3), 292-296.

**Keywords:** Developmental Coordination Disorder; Attention Deficit Hyperactivity Disorder; Children; Identification.

### Reliability of the modified Resistance Training Skills Battery for children with movement impairments

B.J. Furzer, A.L. Thornton, M. Bebich-Philip, K.E. Wright & S.L Reid

UWA Paediatric Exercise Health Research Group, School of Sport Science, Exercise and Health, The University of Western Australia, WA, Australia. bonnie.furzer@uwa.edu.au

**Aim:** Muscle strength is a key component of fitness and research in typically developing (TD) children suggests up to 70% of the variability of performance, across a range of motor skills, could be due to muscular strength and physical development variables. Recent research has reported that children with probable Developmental Coordination Disorder (p-DCD) have lower muscular strength than TD children; however, strength assessments have traditionally included exercises that require a high level of coordination, a potential limitation for children with pDCD. Therefore, the aim of this research was to develop a modified version of the Resistance Training Skills Battery (RTSB) for children aged 7-12 years with potential movement impairments and determine the inter rater reliability of the modified-RTSB for the field-based assessment of competence and proficiency related to muscle strength.

**Method:** 10 children aged 7-12 years with pDCD were recruited prior to entry into a community based exercise program. Children with musculoskeletal impairments were excluded. Age appropriate amendments were made to the RTSB in consultation with a panel of experts in paediatric exercise. Movement proficiency was assessed using the Movement Assessment Battery for Children-2 (MABC-2), with children below the 15th percentile classified as pDCD. For each individual skill of the RTSB participants observed the movement, received verbal instructions and were given a trial attempt to ensure instructions were understood. General encouragement was provided but no skill-specific feedback. Participants completed two trials of four repetitions for the skills that included body weight squat, push-up, step-up, suspended row, standing overhead press and front support with chest touches. All skills were recorded using a digital video camera positioned to capture the technical aspects of movement in both the sagittal and frontal plane. Video recordings of the participants performing the RTSB were used post-hoc to assess the quality of movement and determine inter-rater reliability of the scoring protocol.

**Results & Discussion:** Given the potential role of resistance training in improving movement for children with pDCD, valid and reliable assessments to determine competency and proficiency are required. Data collection and analysis for the modified-RTSB is currently in progress, with full results and conclusions from this study presented at the conference.

**Keywords:** Developmental Coordination Disorder; Resistance training; Movement impairments; Muscle strength; Strength assessment.

### Effect of tool choice and length on reach distance estimation in children with Developmental Coordination Disorder

C. Gabbard^**1**^, M. Ricard^**2**^ & P. Caçola^**2**^

^1^Department of Health and Kinesiology, Texas A&M University, 77845, USA, c-gabbard@tamu.edu; ^2^Department of Kinesiology, University of Texas at Arlington, USA.

**Aim:** Research indicates that children with Developmental Coordination Disorder (DCD) may have difficulty with action planning. With the task of estimating reach distance to objects via tool use, typically developing (TD) children and those with DCD are similar with a shorter tool (20 cm) but TD children are more accurate with a longer tool (40 cm). One similarity with previous work is that participants were explicitly instructed to use a specific tool to estimate reach. Here, we increased the cognitive demands of the task by providing a choice between 2 different lengths.

**Method:** Children with DCD (*n* = 25; MABC-2 < 9th percentile; mean age 9.5 years) and TD children (*n* = 23; > 16th; 10.2 years) were measured for maximum seated reach with their dominant arm and tools of 10-, 20-, 30-, and 40 cm length. For each length, seven 2 cm targets were displayed: 3 below and 3 above maximum reach. Participants had to choose, based on reach estimation using motor imagery, arm and/or tool length that would be closest to the target: two choices were provided in 4 conditions (A-D): Arm or 10 cm, 10- or 20 cm, 20- or 30 cm, and 30- or 40 cm. Targets were presented in the participants’ midline and responses given via keypad. Participants were trained in motor imagery and familiarized with arm and tool lengths. A 2 (Group) x 4 (Condition) mixed-method analysis on total accuracy was conducted.

**Results:** No overall Group effect, however, there was a significant effect for Condition and an interaction (*p* < .01). There was no difference between conditions A-C, with all being different from D (30-40 cm) (less accurate). Furthermore and most interesting, the DCD group scored significantly higher than the TD group in condition D. Although not significant, TD group scores were higher in all other conditions.

**Discussion:** Whereas this result was unexpected and additional research needed, we can only speculate that due to the cognitive demands of choice, children with DCD may have relied primarily on visual information, rather than use of motor imagery, which was apparently advantageous with the longest tool length. As noted earlier, both groups had more difficulty with the longer length condition.

**References**:

Adams, I. L. J., Lust, J. M., Wilson, P. H., & Steebergen, B. (2014). Compromised motor control in children with DCD: A deficit in the internal models?-A systematic review. *Neuroscience and Biobehavioral Reviews, 47*, 225-244. http://dx.doi.org/10.1016/j.neubiorev.2014.08.011

Caçola, P., Gabbard, C., Ibana, M., & Romero, M. (2014). Tool length influences reach distance estimation via motor imagery in children with Developmental Coordination Disorder, *Journal of Clinical and Experimental Neuropsychology, 36*(5), 596-606. doi:10.1016/j.neubiorev.2014.08.011

**Keywords:** Tool use; Motor imagery; Reach.

### Social participation and interventions supporting teenagers and young adults living with DCD: Results from a scoping review

M. Gagnon-Roy^**1**^, E. Jasmin^**1**^ & C. Camden,^**1,2**^

^1^Department of Readaptation, University of Sherbrooke, Sherbrooke, J1K 2R1, Canada. mireille.gagnon-roy@usherbrooke.ca; ^2^Centre de Recherche du CHUS, Sherbrooke, Canada.

**Aim:** Developmental Coordination Disorder (DCD) is a neurodevelopmental disorder affecting approximately 5-6% of children^1^. Coordination impairments impact on motor planning causing children to struggle to perform various activities and to participate in daily living activities. Because DCD is a life-long condition and teenagers and young adults are requested to perform new life habits, DCD could cause new disabling situations as children grow and want to become independent. However, the impact of DCD on youths’ participation, and the interventions that could help them, are not well documented. The aim of this project is to synthesize the actual knowledge on the social participation challenges faced youths (15-25 years old) living with DCD.

**Method:** A scoping review following Levac et al. proposed steps^2^ was performed and the scientific and grey literature was searched. A combination of keywords (DCD, teenagers, young adults, participation) was used in databases (PubMed, CINAHL, PsycINFO). The Internet was also searched and emails were sent to experts in the field to identify all potential relevant documents. The Disability Creation Process (DCP)^3^, composed of 12 life habits categories influencing social participation, has been used as a framework to extract the information from the documents and present the results.

**Results:** Few documents were retrieved; most talked about the social participation challenges faced by youths with DCD and very little interventions or resources were proposed to help them. Some of the challenges experienced by youths with DCD are similar to the ones faced by children, such as difficulties with interpersonal relationship (bullying) and leisure (poor implication in group sports, avoidance of social activities like dancing). Education is also affected by DCD (difficulties in manual writing), but challenges are different. Likewise, mobility is affected differently, particularly because youths normally learn to drive. Driving, that can cause safety challenges since youths with DCD have a slower response to stimulus. Responsibilities change, and financial management is particularly challenging.

**Discussion:** Many life habits are challenging for teenagers and young adults living with DCD. Interventions and resources are needed to support social participation for these youths. Results will be used to develop some of these in collaboration with a parents’ association.

**References**:

1. American Psychiatric Association (2013). *Diagnostic and statistical manual of mental health disorders* (5^th^ ed). Washington: American Psychiatric Association.

2. Levac, D., Colquhoun, H. & O’Brien, K. (2010). Scoping studies: advancing the methodology. *Implementation Science, 5*(69). doi:10.1186/1748-5908-5-69

3. Fougeyrollas, P. (1998). *Classification québécoise: processus de production du handicap*. Lac-St-Charles (Québec): Réseau international sur le processus de production du handicap.

**Keywords:** Developmental Coordination Disorder; Adults; Teenagers; Participation; Life habits.

### Evaluating the Partnering for Change service delivery model: Teachers’ pre-intervention evaluation of school functioning in children with Developmental Coordination Disorder?

R. Gaines^**1,2**^, W. Campbell^**2,3**^, C. DeCola^**2**^, N. Pollock^**2,3**^, S. Bennett^**4,2**^, C. Camden^**5,2**^, D. McCauley^**2**^ & C. Missiuna^**2,3**^

^1^Children’s Hospital of Eastern Ontario Research Institute, Ottawa, ON, K1H 8L1, Canada. gaines@cheo.on.ca; ^2^CanChild Centre for Childhood Disability Research, McMaster University, Hamilton, ON, Canada; ^3^School of Rehabilitation Sciences, McMaster University, Hamilton, Canada; ^4^Department of Teacher Education, Brock University, St. Catharines, Canada; ^5^Centre de recherche du CHUS & School of Rehabilitation, Université de Sherbrooke, Sherbrooke, Québec, Canada.

**Aim:** Partnering for Change (P4C) is an evidence-informed occupational therapy service for school-aged children with Developmental Coordination Disorder (DCD). It aims to: 1) increase early identification; 2) build teacher capacity to manage DCD; 3) improve participation; and 4) prevent secondary disability. Currently, we are studying the impact of P4C on outcomes of children with probable DCD over 2 school years. The poster describes teachers’ pre-evaluation of the functioning of children with DCD at school, including the provision of assistance and adaptive supports.

**Method:** 392 children receiving the P4C service participated. 171 children were referred from waitlists, with another 221 children identified by the occupational therapists. Teachers completed the School Function Assessment (SFA) to document pre-P4C participation in 5 different school settings. The extent to which the children required assistance and/or modifications to perform 9 physical tasks and 9 cognitive/behavioral tasks was also rated.

**Results:** On average, children were rated as participating in all school settings with either no or some modifications. Some children were judged to have limited participation or require assistance. Teachers also reported that children required, on average, minimal to no assistance and modifications when completing most physical or cognitive/behavioral tasks. Statistical comparisons of the extent to which teachers provided assistance versus modifications indicated that teachers consistently reported relying on assistance more frequently, although this was a small effect.

**Discussion:** Evidence shows that DCD is under-recognized and that children with DCD often do not receive adequate support. Pre-intervention teacher ratings support this observation: in general, teachers did not perceive children with suspected DCD as being limited in their participation or needing assistance or modifications with school tasks. We hypothesize that teachers may not recognize the needs of the children with DCD; hence, their provision of little support. We anticipate the knowledge translation component of P4C will highlight the many unrecognized needs of this population. Comparisons to year 2 data will allow us to test this hypothesis. Of note, when teachers do provide support they are slightly more likely to provide overt assistance rather than modify the environment. We anticipate that their ability to make environmental modifications will be strengthened by the P4C service.

**Keywords:** Comorbidity; Management; Teacher awareness of DCD; School participation.

### Elementary visuospatial perception impairment in a group of children with developmental coordination disorder

S. Gonzalez-Monge^**1**^, C. Caton^**2**^, E. Luc-Pupat^**1**^, A. Barriere^**1**^, D. Chatelus^**1**^, D. Jacquemot^**1**^, M.A.Rocher^**1**^, Y. Rossetti^**2**^& L Pisella^**2**^

^1^Department of Pediatric rehabilitation, Hospices Civils de Lyon, Groupement Hospitalier Est, Bron, France. sibylle.gonzalez-monge@chu-lyon.fr; ^2^ImpAct – Lyon Neuroscience Research Center, Inserm U1028, CNRS UMR 5292, Université de Lyon, Biologie Humaine, Bron, France.

**Aim:** Children with DCD (Developmental Coordination Disorder) constitute a heterogeneous group in which the possible impairment of visuospatial perception should be evaluated in order to orient toward specific therapies (visual or motor). However, visuospatial perception is usually assessed in clinical routine by multifactorial tests, involving in particular motor responses. In a previous study, we normalized a simple, fast and specific test of elementary visuospatial perception (whose responses do not require action, multi-choice, object recognition or language) on 96 children between 4 and 12 years old^1^. The aim of this study is therefore to assess the presence of visuospatial perception impairment in children with DCD, using this test.

**Method:** We tested visuospatial perception in a group of 43 DCD children using this test to compare with age-matched controls. We also assessed the correlation of these scores of visuospatial perception with scores in other tests evaluating praxic, cognitive and writing abilities (Wechsler, NEPSY-II and BHK).

**Results:** The visuospatial perceptual performance in DCD children significantly differed from age-matched controls, establishing that this test can be discriminant to diagnose DCD (Mann Whitney test and ROC curve), with 60% of DCD children lying below the interquartile range of controls. Moreover, the score of visuospatial ability was significantly correlated (Spearman test) to the scores of the Wechsler PIQ, to the sub-scores Cube and Copy of the NEPSY-II, but neither to the writing scores of the BHK (neither the speed nor the quality) nor to the Wechsler scores VIQ and Processing speed.

**Discussion:** Based on these results, we expect that the test of visuospatial perception can become an interesting tool for an early and large detection of potential DCD and also for a differential care of visuo-spatial and non-visuospatial forms of DCD (with dysgraphia probably rather associated with the latter).

**References**:

Pisella L., André V., Gavault E., Le Flem A., Luc-Pupat E., Glissoux C., Barrière A., Vindras P., Rossetti Y., & Gonzalez-Monge S. (2013) A test revealing the slow acquisition and the dorsal stream substrate of visuo-spatial perception. *Neuropsychologia, 51*(1):106-113.

**Keywords:** Evaluation; Visuo-spatial perception; Elementary; Child; DCD (Developmental Coordination Disorder).

### The relationship between motor coordination, cognitive abilities, and academic achievement in Japanese children with developmental disorders

T. Higashionna^**1**^, A. Tokunaga^**1**^, A. Nakai^**2**^, K. Tanaka^**1**^, H. Nakane^**1**^, G. Tanaka^**1**^ & R. Iwanaga^**1**^

^1^Department of Occupational Therapy, Nagasaki University Graduate School of Biomedical Sciences Health Sciences, Nagasaki, Japan. h_takuya_3515@yahoo.co.jp; ^2^Hyogo Children’s Sleep and Developmental Medical Research Center, Kobe, Japan.

**Aim:** Because movement impairment may arise from neurological impairment (Green et al., 2009), and motor and cognitive development are fundamentally interrelated. The aim of this study is to examine the relationship between motor coordination and cognitive abilities, and academic achievement in children with developmental disorders.

**Method:** Forty-one children with developmental disorders (34 males, 7 females; age range 6-17 years old; mean age 10.22±3.12 years) participated. Cognitive abilities and academic achievement were assessed using the Kaufman Assessment Battery for Children, Second Edition (KABC-II) and motor coordination was assessed with the Movement Assessment Battery for Children 2 (MABC-2).

**Results:** There was a positive significant correlation between total cognitive score of KABC-II and Manual dexterity of MABC-2 (r=.370, p=.017). There were also positive significant correlations between total academic achievement, and Manual dexterity(r=.357, p=.022) and Balance(r=.312, p=.047). In the cognitive abilities, Simultaneous processing(r=.447; p=.003) and Planning ability(r=.336, p=.032) significantly correlated with the Manual dexterity. In academic achievement, Mathematical achievement significantly correlated with Manual dexterity (r=.410, p=.008) and Balance (r=.336, p=.032). In Ball skills, only Aiming & Catching 2 (A&C2) negatively, significantly correlated with the Crystallized ability (r=-.359, p=.021). On Cattell-Horn-Carroll (CHC) model, Visual processing (r=.453, p=.003), Fluid reasoning (r=.334, p=.033), Quantitative knowledge (r=.410, p=.008) and total CHC score (r=.365, p=.019) positively, significantly correlated with Manual Dexterity.

**Discussion:** Our findings suggest that there is a relationship between motor problems, cognitive abilities and academic achievement in children with developmental disorders. These motor coordination problems have a negative impact on accuracy and speed, which further inhibits the ability to integrate spatial stimulus and planning ability. And since fine motor ability is also associated with Visual processing, Fluid reasoning and Quantitative knowledge, fine motor skills might be related to visual information processing, reasoning capability and mathematical knowledge.

**References**:

Green D, Charman T, Pickles A, et al. Impairment in movement skills of children with autistic spectrum disorders. Developmental Medicine & Child Neurology 2009; 51: 311-316.

**Keywords:** Developmental disorders; Motor coordination; Cognition; Academic skill; MABC-2.

### Longitudinal change in motor skills in children with autism spectrum disorders

S. Hirata^**1**^, Y. Kita^**2**^, K. Suzuki^**2**^, H. Okuzumi^**3**^, M. Kokubun^**3**^& A. Nakai^**4**^

^1^Department of Elementary Education, Ibaraki Christian University, Hitachi, 319-1295, Japan. r093002g@st.u-gakugei.ac.jp; ^2^Department of Developmental Disorders, National Institute of Mental Health, National Center of Neurology and Psychiatry, Kodaira, Japan; ^3^Faculty of Education, Tokyo Gakugei University, Koganei, Japan; ^4^Department of Pediatric Neurology, Department of Pediatrics & Department of Child and Adolescent Psychiatry, Hyogo Children’s Sleep and Development Medical Research Center, Kobe, Japan.

**Aim:** In recent years, empirical studies have revealed the existence of motor skill impairments in children with autism spectrum disorders (ASD), using standardized assessment batteries such as the Movement Assessment Battery for Children-2 (MABC-2). In general, children with ASD show a lower total score with this test. However, since longitudinal change in motor skills in children with ASD has not been fully investigated, we examined this point in the present study.

**Method:** Participants were 22 children with ASD (mean age 11.6±2.6 years, mean IQ 96.0±6.0). We conducted the MABC-2 for three years, with an interval of one year between measurements. Thus, we obtained three MABC-2 total scores. The MABC-2 has not been standardized in Japan yet. The norm attached to the test manual was used when raw scores were converted to percentile and standardized score (M=10, SD=3). Lower standard scores indicate greater severity of motor skill impairments.

**Results:** In order to clarify individual differences in motor skill impairments among children with ASD, we classified each percentile of MABC-2 total score into three groups according to the MABC-2 manual: ‘definite motor impairment (below the 5th percentile),’ ‘borderline impairment (6th–15th percentile inclusive),’ and ‘no impairment (above the 16th percentile).’ In the first measurement, 46% of the children with ASD had at least below the 15th percentile in their total score. In the third measurement, however, this proportion was decreased, and 36% of the children with ASD scored below the 15th percentile. The ANOVA also revealed that the MABC-2 total standard score was significantly raised with age in children with ASD whose MABC-2 total score was below the 15th percentile in the first measurement. On the other hand, the MABC-2 total standard score was not significantly changed with age in children with ASD whose MABC-2 total score was above the 16th percentile in the first measurement.

**Discussion:** Results of this study indicated the possibility that lower MABC-2 scores in children with ASD slightly improved with age. Because of the relatively small sample size, we couldn’t have the robust conclusion. But, several factors which affect the performance of MABC-2 were identified. One is the executive dysfunction, i.e., the problem of adaptation to novel motor tasks. Further investigation focused on the relationship between motor skills and executive function in children with ASD is therefore warranted.

**Keywords:** Autism spectrum disorders; MABC-2; Longitudinal change.

### Children with Developmental Coordination Disorder show abnormal intra-limb coordination patterns in jumping and hopping but not in walking

M.J. Hung^**1**^, C.Y. Cho^**1**^, J.F. Yang^**1**^, & R.J. Cherng^**1,2**^

^1^Department of Physical Therapy, National Cheng Kung University, Tainan, Taiwan. rjc47@mail.ncku.edu.tw; ^2^Institute of Allied Health Sciences, National Cheng Kung University, Tainan, Taiwan.

**Aim:** Studies showed that children with developmental coordination disorder (DCD) had poorer and more variability locomotion performance than typically developing (TD) children. However, such comparisons of the locomotion performance were focused on global temporal distance parameters rather than intra-limb coordination. The purposes of this study were to examine the differences of locomotion performance between children with DCD and TD children from the perspectives of intra-limb coordination patterns.

**Method:** Eighteen children with DCD (6.8 ± 1.7 years) and 18 age-matched TD children (6.9 ± 1.6 years) participated in the study. A six-camera Qualisys motion capture system (ProReflex) was used to collect participants’ lower limb kinematic information during three locomotion tasks: 1) walking, 2) consecutive forward jumping and 3) consecutive forward hopping on the preferred leg. Following the law of the planar covariation^1^, the study used the principal component analysis method to analyze the interdependencies of each segment of lower limb (thigh, shank, and foot) and their elevation angles and to determine their contributions to planarity configurations. Three variables were analyzed to explain the intra-limb coordination performance: the limb elevation angle, the orientation of the covariation plane, and the planarity index (the sum of variance in PC1 and PC2: λ1+λ2). Repeated-measure analysis of variances and post-hoc analyses with SPSS version 17.0 were used to examine the effects of the group, task, and their interaction on the coordination variables. Statistical significance was set at *p* < 0.05.

**Results:** The results showed that the minimum foot elevation angle was larger in children with DCD than TD children in jumping and hopping (jumping, DCD: 8.38 ± 11.00, TD: 1.07 ± 9.23, *p* = 0.04; hopping, DCD: 20.37 ± 10.64, TD: 13.01 ± 7.00,* p* = 0.02). That is, the amplitude of foot elevation angle was smaller in children with DCD than TD children in jumping and hopping. No significant differences between the groups in the other limb segments during walking, jumping and hopping. No significant interaction between group and task condition, neither the effect of group on the orientation of covariation plane. Only the effect of task condition was significant (F_2,68_ = 9.23, p < 0.001). The group, task, and interaction effects on planarity index were all significant. Post-hoc analyses showed that children with DCD had a smaller planarity index than TD children in jumping (97.44 ± 0.95% vs. 98.43 ± 0.64%,* p* = 0.001) and hopping (94.86 ± 1.24% vs. 96.54 ± 1.22%, *p* < 0.001).

**Discussion:** Walking is a basic and the first upright locomotion task in children. It emerges and develops at approximately 10-15 months of age. Jumping and hopping are more advanced skills which require adequate timing and spatial coordination control of multiple limbs. TD children do not present the skill of jumping and hopping until the at age of 2-4 years and 3-5 years respectively. Children with DCD who are characterized with delayed motor development do not show different intra-limb coordination from TD children in walking but evident in jumping and hopping.

**References**:

1. Borghese, N. A., Bianchi, L., & Lacquaniti, F. (1996). Kinematic determinants of human locomotion. *Journal of Physiology, 494*(3), 863-879.

**Keywords:** Developmental coordination disorder; Intra-limb coordination; Locomotion; Task complexity.

### Pilot study: Characteristics of motor impairment in children with autism spectrum disorder

R. Iwanaga, A. Tokunaga, T. Higashionna, G. Tanaka, H. Nakane & K. Tanaka

Department of Health Sciences, Nagasaki University Graduate School of Biomedical Sciences, 1-7-1 Sakamoto, Nagasaki, 852-8520 Japan. iwanagar@nagasaki-u.ac.jp

**Aim:** DSM-IV did not allow DCD diagnosis concurrent with autistic disorder or Asperger’s disorder. However, co-occurrence of DCD and autism spectrum disorder (ASD) is common (APA, 2013). Previous studies reported relationships between some motor impairments and autistic symptoms, and similarities, and differences between motor impairment in children with ASD, and DCD. To make treatment plans for children with ASD, we need to fully understand their specific motor impairments, and the relationships between motor impairment, and autistic tendency and intelligence abilities. The aim of this study was to clarify the characteristics of motor impairment in children with ASD from various perspectives and to analyze the relationship between these impairments, and autistic tendency and intelligence abilities.

**Method:** The participants were 23 children with ASD aged 5 to 10 years old (19 boys and 4 girls; mean age was 8 years 5 months ± 14 months). All of them were diagnosed with ASD. IQs measured by WISC-III ranged from 61 to 110 (mean 79 ± 14.7). We conducted the 6 motor tests from the Southern California Sensory Integration Tests (SCSIT), the Prone extension posture test, and the Supine flexion posture test, as well as measuring participants’ grasping power. Z scores of each test were calculated. And correlations between the motor tests’ Z scores, and IQ, and Childhood Autism Rating Scale (CARS) scores were analyzed.

**Results:** Z scores of each tests were as follows: Motor accuracy test right and left were -1.9 and -1.9 respectively, Imitation of posture was -0.4, Crossing midline was -1.6, Bilateral motor coordination was -1.1, Standing balance eyes open was -2.0, Standing balance eyes closed was -1.7, Prone extension was -1.9, Supine flexion was -1.3, and grasping power was -1.7. However there were no significant correlations between motor tests and CARS scores, correlation between grasping power and CARS scores was marginally significant. IQ significantly correlated with MAC-R right and grasping power, but did not with other tests.

**Discussion:** This study demonstrated that children with ASD have problems with balance, maintaining anti-gravity posture, griping power, hand-eye coordination, rhythmic bilateral hand movement, and crossing midline movement. Sine Crossing midline test scores reflect not only crossing midline movement skills but also goal-less movement skill, its lower scores might indicate difficulty in goal-less movement that was demonstrated in previous studies for children with ASD. This study suggests that grasping power and hand-eye motor coordination of the right hand were related to intelligence abilities in children with ASD. This study has limitations resulting from the small sample size, so further study should be conducted with large samples on motor impairment of children with ASD.

**References**:

American Psychiatric Association (2013). *Diagnostic and Statistical Manual of Mental Disorders. 5th edition.* Washington, DC: American Psychiatric Association.

**Keywords:** Autism Spectrum Disorder; Southern California Sensory Integration Tests; Motor impairment.

### Reliability of peak power output in countermovent jumps in children aged 9 to 11 years

C.M. Jones, N.J. Owen, J. Watkins & A. Foulkes

Applied Sports Technology, Exercise and Medicine Research Centre, Swansea University, Swansea, SA2 8PP, UK. 551514@swansea.ac.uk

**Aim:** The diagnosis of developmental coordination disorder (DCD) is derived, in part, from standardised motor coordination tests, but the reliability of the tests is acknowledged to be poor^1^. A high level of peak power output (PPO) in countermovement vertical jumps (CMJ) is a key determinant in successful athletic performance^2^, conversely it is reasonable to assume that a low level of PPO in a CMJ would be associated with poor athletic performance, characterised by poor coordination. Jumping is a common activity for children and consequently has potential as a measure of coordination. There are however no valid data on the reliability of PPO in children. Therefore the purpose of this study was to determine the reliability of PPO in a CMJ in 9-11 year olds.

**Method:** School children (n = 24, age = 10.4 ± 0.5 years, height = 1.43 ± 9.2 cm, mass = 41.0 ± 11.4 kg) performed 6 CMJ’s, separated by periods of rest, off a force platform, on the same day. The CMJ protocol excluded the use of arms to isolate the lower body. Force-time histories were collected and the impulse momentum relationship was used to determine a criterion measure of peak lower body power output (Pp)^3^. Pps were expressed relative to body mass. Intraclass correlation coefficient (ICC) was used to determine reliability.

**Results:** Average Pp ranged from 33.5 to 34.4 W/kg for females and from 35.8 to 37.0 W/kg for males. The highest and lowest Pps achieved were 20.8 W/kg and 46.9 W/kg, both by males. The ICC between attempts ranged from 0.841-0.969 and 0.924-0.987 for males and females with the peak attempt mean ICC reaching 0.990 and 0.991 (p < 0.05). There was no significant difference between sexes (p > 0.05).

**Discussion:** The average ICC value between 6 attempts of 0.929 was indicative of vertical jumping being a well-practiced skill allowing the CMJ to be classified as a reliable test of Pp in children of both sexes between the ages of 9 and 11 years. Results suggest that 3 CMJs is a sufficient and reliable protocol for measuring Pp in children this age. Proposed future research is to collect normative data (ND) for Pp in children in this age range and compare values of Pp for DCD children to ND.

**References**:

1. Brown, T., & Lalor, A. (2009). The Movement Assessment Battery for Children - Second Edition (MABC-2): a review and critique. *Physical & Occupational Therapy in Pediatrics, 29*(1), 86-103.

2. Kawamori, N., Crum, A. J., Blumet, P. A., Kulik, J. R., Childers, J. T., Wood, J. A., Stone, M. H., & Haff, G. (2005). In?uence of different relative intensities on power output during the hang power clean: Identi?cation of optimal load. *Journal of Strength and Conditioning Research, 19*, 698-706.

3. Owen, N. J., Watkins, J., Kilduff, L. P., Bevan H. R., & Bennett, M. A. (2014). Development of a criterion method to determine peak mechanical power output in a countermovement jump. *Journal of Strength and Conditioning Research, 28*(6), 1552-1558,

**Keywords:** Power; Coordination; Vertical jumping; Children; Force platform.

### Evaluation of motor coordination in children with epilepsy, using a japanese version of the DCDQ

M. Kashiwagi^**1**^, T. Tanabe^**2**^, C. Ooba^**1**^, E. Wakamiya^**3**^, A. Nakai^**4**^ & H. Tamai^**5**^

^1^Department of Pediatrics, Hirakata City Hospital, Hirakata City, Japan. dbs003@art.osaka-med.ac.jp; ^2^Department of Pediatric Neurology, Tanabe Pediatric clinic, Hirakata City, Japan; ^3^Faculty of Nursing and Rehabilitations, Aino University, Ibaraki City, Japan; ^4^Hyogo Children’s Sleep and Development Medical Research Center, Kobe City, Japan; ^5^Department of Pediatrics, Osaka Medical College, Takatsuki City, Japan.

**Aim:** There is various examination about the cognitive function of the epilepsy child, but there is little study about the motor coordination. Using a Developmental Coordination Disorder Questionnaire Japanese edition (DCDQ-J: Nakai A, et al. 2011), we evaluated the motor coordination of children with epilepsy.

**Method:** Forty-nine epilepsy children (29 boys and 20 girls) in our hospital were evaluated. The average age of DCDQ-J is 10 years 8 months old (6 years 6 months old-15 years and 6 months old). The average age of onset of epilepsy is 6 years 11 months old (2 years old-13 years and 10 months old). The average duration of epilepsy is 3 years 4 months old (6 months -11 years and 10 months). The average duration of anti-epileptic drugs is 2 years 9 months old (1 month -10 years). The average full IQ of 46 cases (94%) is 89.2. Fourteen cases (29%) were symptomatic epilepsy. Twelve cases (24%) took more than two anti-epileptic drugs. Twelve cases (24%) took more than two anti-epileptic drugs. Five cases (10%) had more than monthly seizure frequency. In DCDQ-J, child with score less than 15 percentile was suspected motor problem. This study was approved by the ethics committee of Hirakata City hospital.

**Results:** Sixteen cases (32%) in total score, 17 cases (34%) in control during movement score, 16 cases (32%) in fine motor score and 14 cases (29%) in general coordination score were less than 15 percentile. Total score was correlated with the age of onset of epilepsy, the duration of drugs and full IQ. Fine motor score was correlated with the age of onset of epilepsy, the duration of epilepsy, the duration of drugs and full IQ. Control during movement score was correlated with the duration of epilepsy, the duration of drugs and full IQ. The age of DCDQ-J, the age of onset of epilepsy, the duration of drugs and full IQ were not significantly different between 16 cases of suspected motor problem and the other 33 cases. The rates of symptomatic epilepsy, more than two anti-epileptic drugs and more than monthly seizure frequency in 16 cases of suspected motor problem were significantly higher than the other 33 cases.

**Discussion:** Approximately 30% of children with epilepsy were suspected motor problem. Motor problem was correlated with symptomatic epilepsy, more than two anti-epileptic drugs and more than monthly seizure frequency. In epilepsy medical care, we should pay more attention to the motor problem, and the support is necessary.

This study was supported, in part, by a Grant-in-Aid for Scientific Research from the Japan Society for the Promotion of Science

**References**:

Nakai, A., Miyachi, T., Okada, R., Tani, I., Nakajima, S., Onishi, M., … Tsujii, M. (2011). Evaluation of the Japanese version of the Developmental Coordination Disorder Questionnaire as a screening tool for clumsiness of Japanese children. *Research in Developmental Disabilities, 32*(5), 1615-1622.

**Keywords:** Epilepsy; Motor problem; Symptomatic epilepsy; Anti-epileptic drugs; Seizure frequency.

### Longitudinal prediction of psychosocial maladaptation and educational achievement in elementary school based on development assessment of fine and gross movement by nursery teachers

M. Katagiri^**1**^, H. Ito^**1**^, Y. Murayama^**1**^, M. Hamada^**1**^, A. Uemiya^**1**^ & M. Tsujii^**1,2**^

^1^Research Center for Child Mental Development, Hamamatsu University School of Medicine, 1-20-1 Handayama, Higashi-ku, Hamamatsu, Shizuoka, 431-3192, Japan. mitty1024@gmail.com; ^2^School of Contemporary Sociology, Chukyo University, 101 Kaizutyou-tokodate, Toyota, Aichi, 470-0393, Japan.

**Aim:** We examined the extent to which nursery teachers’ development assessment of fine and gross movement predicts later psychosocial maladaptation and educational achievement in elementary schools. The Developmental Scale for Nursery Record (DSNR) we developed yielded nine subscales in principal component analysis, including fine and gross movement. We have already confirmed reliability and validity in this scale.

**Method:** A 7-year longitudinal investigation was conducted on 2,501 children (female 1,209, male 1,292) from all nursery and elementary schools in a suburban city. We used the NDSC for the development assessment by nursery teachers. Preschool children were assessed by their nursery teacher right before entering elementary school using the NDSC. We conducted multiple regression analysis on the collected data. The independent variables were fine and gross movement in the DSNR subscales. The dependent variable was later educational achievement (a deviation score on an academic achievement test), friendship, behavioral problems, and emotional problems (subscale scores on the Strengths and Difficulties Questionnaire).

**Results:** We found that fine movement suffers significantly under the influence of educational achievement. Furthermore, gross movement suffers significantly under the influence of friendship and emotional problems. There was no relationship between motor skills and behavioral problems.

**Discussion:** These findings indicated that fine motor skill predicts later educational achievement, and gross motor skill predicts later friendship and anxiety and/or depressive tendency. Thus, problems with motor skills in preschool children lead to the potential for lower educational achievement and psychosocial maladaptation. In conclusion, assessment of motor skills in preschool children may enable early detection and appropriate treatment of children who have special educational needs and/or psychosocial maladaptation. In addition, elementary schools need to contribute to educational planning after due consideration of motor skills in children.

**Keywords:** Fine motor skill; Gross motor skill; Psychosocial maladaptation; Educational achievement.

### Motor imagery in children with Developmental Coordination Disorder: a complex hand rotation task

S. Kerrigan^**1**^, J. Reynolds^**1**^, M. Licari^**1**^, C. Elliott^**2**^, B. Lay^**1**^ & J. Williams^**3**^

^1^School of Sport Science, Exercise and Health, The University of Western Australia, Perth, 6009, Australia. 20939831@student.uwa.edu.au; ^2^Faculty of Health Sciences, Curtin University, Perth, Australia; ^3^College of Sport and Exercise Science, Victoria University, Melbourne, Australia.

**Aim:** It has been hypothesised that deficits in the functioning of internal modelling may contribute to the motor impairments associated with DCD. This process can be explored behaviourally through motor imagery paradigms. This study examined motor imagery proficiency of children with and without DCD using a complex hand rotation task.

**Method:** 47 boys aged 7-13 yrs participated in this study, 25 with DCD (mean age=9.66yrs±1.56) and 22 controls (mean age=9.69yrs±1.61). Children were tested on the MABC-2, with children in the DCD group ≤16th percentile and controls ≥20th percentile. Motor imagery proficiency was measured using a complex hand rotation task administered via E-Prime 2.0. Images were presented in two rotational axes – palm view and back view, and eight 45° rotational steps between 0° and 360°. Participants completed the task twice: first with no instructions, and the second time with motor imagery instructions. To explore the effect of instructions and whether response patterns followed biomechanical constraints, response time (RT) and accuracy (ACC) data were submitted to separate RM-ANOVAs (2[instructions] x 2[view] x 2[laterality] x 2[group]). RM-ANOVAs (2[instructions] x 5[angle] x 2[group]) were then conducted to explore RT and ACC response patterns for back and palm view separately.

**Results:** Significant effects for instructions (p<0.001), view (p<0.001) and laterality (p<0.001) were revealed for both RT and ACC, indicating both groups were faster and more accurate with instructions, for back compared to palm view, and medial compared to lateral rotations. No significant group effects for RT (p=0.061) or ACC (p=0.076) were identified for responses to back view, although both approached significance. A significant group effect was revealed for palm view response accuracy (p=0.011), with children with DCD having lower accuracy levels than controls.

**Discussion:** There was partial support for the hypothesis that children with DCD would display atypical response patterns on the hand rotation task. A large degree of variation was observed within the DCD group, with a number falling within the control response range. It appears, however, that deficits in motor imagery proficiency may become more apparent as the task complexity increases, with group differences identified for palm view. A greater understanding of motor imagery performance in children with DCD has the potential to inform intervention programs.

**Keywords:** Motor imagery; Mental rotation, Internal modelling, Mirror neuron system.

### A preliminary study of the Movement Assessment Battery for Children-Second Edition on Japanese children: Age Band 2

Y. Kita^**1**^, S. Hirata^**2**^, K. Suzuki^**1**^, K. Sakihara^**3**^, M. Inagaki^**1**^ & A. Nakai^**4**^

^1^Department of Developmental Disorders, National Institute of Mental Health, National Center of Neurology and Psychiatry, 4-1-1 Ogawahigashi, Kodaira, Tokyo, 187-8553, Japan. kitay@ncnp.go.jp; ^2^Department of Special Needs Education, Chiba University, Japan; ^3^Department of Clinical Laboratory Science, Faculty of Medical Technology, Teikyo University, Tokyo, Japan; ^4^Hyogo Children’s Sleep and Development Medical Research Center, Kobe, Hyogo, Japan.

**Aim:** The Movement Assessment Battery for Children-Second Edition (MABC2) is a commonly used assessment of children’s motor skills in various countries. However, it has not been standardized in Japan; therefore, we have faced difficulties in diagnosing children with developmental coordination disorders. The present study aimed to examine whether norms of the MABC2 is also applicable to Japanese children to prepare for its standardization in Japan.

**Method:** We tested 132 typically developing Japanese children (mean age = 8.78 ± 1.19 yrs; boys = 84) with eight examination items for Age Band 2 of the MABC2. Their raw scores were transformed into scaled scores (SSs) based on the original norms (i.e., UK data), and we looked into sex differences in SSs of each examination items, three component scores, and a total score.

**Results:** Mean SSs of the Manual Dexterity and Balance were above 11 points, while that of the Aiming & Catching was below 10 points. We also found significant sex differences in the Manual Dexterity 1 (p < .05), Balance 1 (p < .001), and Balance 3 (p < .001), in which girls scored higher than boys. Moreover, SSs of the Manual Dexterity and Balance were higher in girls than boys, while that of the Aiming & Catching did not differ between the sexes.

**Discussion:** These results suggest that Japanese children have better manual skills and are better at balancing and jumping compared to children in the UK, particularly in girls. These findings also indicate necessity of setting a new norm for Japanese populations based on larger set of data collected from typically developing Japanese children.

**References**:

Henderson, S. E., Sugden, D. A., & Barnett, A. L. (2007). *Movement Assessment Battery for Children-Second Edition (Movement ABC-2)*. London, UK: Pearson.

**Keywords:** Movement Assessment Battery for Children-Second Edition (MABC2); Age Band2; Japanese; Sex differences.

### Autistic social communication symptoms in children with Developmental Coordination Disorder: a comparative study

J. Kruck & B. Rogé

Department of Psychology, The University Toulouse Jean Jaurès, CERPP laboratory, 5 allée A.Macahdo, 31058 Toulouse. jeanne.kruck@univ-tlse2.fr.

**Aim:** Few researches have attempted to establish a link between ASD and DCD. Some characteristics of children with DCD are also found in children with ASD. This is a disorder of motor development but the communicative and social skills appear altered. The aim of this study is to identify nature of characteristics of social communication functioning for autistic symptoms (using the Social Communication Questionnaire (SCQ) in a population of DCD Children.

**Method:** 88 children to 8-12 years old (30 ASD children (Asperger), 30 DCD children and 28 typical children diagnosed in reference center with ADI/ ADOS/ DSM IV/ CIM 10/M-ABC) have participated. Social communication questionnary (SCQ) (current form) have been completed by parents to different groups. WISC IV has allowed to check IQ level. SCQ total score was calculated as well as the scores in the 3 domains (social reciprocal interaction, communication and stereotyped and restricted behaviors) and compared between populations. All items was compared one by one. T-test were realized.

**Results:** ASD Children have a SCQ total score, communication score (COM) and stereotyped restricted behaviors (SRB) significatively higher than DCD children. Abnormalities in reciprocal social interactions (RSI) are have higher score than ADS children for items of idiosyncratic language, unappropriated questions, using body of other to communicate, pointing to express interest…Further studies are needed to investigate the quality of social communication abnormalities observed in DCD. Social cognition is deficient in children with ASD, a measure of its own cognitions in DCD will be our next study to establish links with the social adaptive functioning.

**References**:

McDuffie, A., Turner, L., Stone, W., Yoder, P., Wolery, M., & Ulman, T. (2007). Developmental correlates of different types of motor imitation in young children with autism spectrum disorders. *Journal of Autism and Developmental Disorders. 37*(3), 401-412.

Provost, B., Heimerl, S., & Lopez, B. R. (2007). Levels of gross and fine motor development in young children with autism spectrum disorder. *Physical Occupational Therapy in Pediatry. 27*(3), 21-36.

Stieglitz Ham, H., Bartolo, A., Corley, M., Rajendran, G., Szabo, A., & Swanson, S. (2011). Exploring the relationship between gestural recognition and imitation: Evidence of dyspraxia in autism spectrum disorders. *Journal of Autism and Developmental Disorders, 41*(1), 1-12. doi: 10.1007/s10803-010-1011-1.

**Keywords:** Developmental coordination disorder; Autism spectrum disorder; Social communication; Screening.

### Retrospective study of the use of the computer in class in a DCD context

P.-L. Lamonzie, C. Niquille & G. Timmerman

¹Centre Pédiatrique de Médecine Physique et de Rééducation St Jacques Roquetaillade, Montégut, 32550, France. roquetaillade.saintjacques@ordredemaltefrance.org

**Aim:** In order to find a solution, setting up computer tools is a measure which has developed. Until now, there has been no consensus^1^ about assessment^2^ or about setting up computer tools in class. After several years of our consultation functioning about learning disabilities, we felt the need to assess our work.

**Method:** A retrospective study about the reception of the DCD children from 2008 to 2014 has been conducted from a questionnaire that we created ourselves. This questionnaire is for the parents and the children of the consultation who had an assessment linked with the relevance of the computer solution. It is composed of several chapters. The first part is to be completed by all the people who were asked for; it deals with the instruction of the computer tool and the school context. The second part is for the ones who have started or totally integrated the tool in class. It is about the integration of the computer in the school years and includes a satisfaction survey.

**Results:** After sending 44 questionnaires, we received 30 answers. Three groups may be distinguished: the children who only made the assessment (30%), the children who were starting the training (13.3%) and the children who use the computer in their school years (56.7%). All the parents are in favor of the project whereas only 64% of the children are motivated. 95.2% of the children of groups 2 and 3 benefited from learning made by occupational therapists. The introduction of computer is supported by the ESS (Education Monitoring Staff) (64.7%) and the training of the environment. It is mainly set up through literary school subjects. Word processing and its extensions are used by all the children of the group. Only a third of the children is concerned by speech recognition.

**Discussion:** If the computer may appear as a simple, efficient and fast solution, the survey strengthens our practice showing that it is a precise assessment process which leads to an individualized suggestion and to a learning^3^ which brings together children, families and school environment^4^, all motivated by this project. As a first goal, it is an ideal tool for the production of texts and the personal organization.

**References**:

1. Freeman, A., Mackinnon, J., & Miller, L. (2005). Keyboarding for students with handwriting problems: a literature review. *Physical and Occupational Therapy in Pediatrics*, 25(1-2), 119-47.

2. Priest, N., & May, M., (2001). Laptop computers and children with disabilities: Factors influencing success. *Australian Occupational Therapy Journal, 48*, 11-23.

3. Guillermin, A-L., & Lévèque-Dupin, S. (2012). Comment l’ordinateur peut-il devenir un outil de compensation efficace de la dysgraphie pour la scolarité ? *Développements,* 12, 26-31.

4. Le Flem, A., & Gardi, C. (2011). Pertinence et limites des aménagements pédagogiques et des compensations pour l’enfant dyspraxique: du conseil pédagogique à l’utilisation de l’ordinateur en classe. *A.N.A.E.,* 111, 57-65.

**Keywords:** Developmental Coordination Disorder; Assistive technology; Laptop computers; Occupational therapist.

### The effects of psychomotor therapy in DCD children with or without comorbidities

A. Laurent^**1**^, J. Lareng-Armitage^**1**^, C. Lewandowski^**1,2**^, P. Abeilhou^**1,3**^, A.-C. Ballouard^**1**^, C. Chaffiotte^**4**^, C. Chignac^**1,5**^, A. Dardour^**6**^, C. Donnadieu^**2**^, C. Ducuing^**1**^, S. Guitard^**1,7**^, D. Innocent Mutel^**1**^, M.A. Pauc^**2**^, M. Salvan^**6**^, D. Toniatti^**8**^ & J.-M. Albaret^**1**^

^1^Psychomotricity Training Institute of Toulouse, University of Toulouse, 133 rte de Narbonne, 31062 Toulouse cedex 9, France. alaurent@adm.ups-tlse.fr; ^2^CMPP de l’Aveyron, PEP 12, France; ^3^Psychomotor therapy private practice, Albi, France; ^4^Psychomotor therapy private practice, Lavaur, France; ^5^Psychomotor therapy private practice, Cugnaux, France; ^6^Psychomotor therapy private practice, Saint-Jory, France; ^7^Psychomotor therapy private practice, Gaillac, France; ^8^Psychomotor therapy private practice, Bagnères de Bigorre, France.

**Aim:** Smits-Engelsman *et al*.^1^ meta-analysis of therapies for patients with DCD revealed that traditional therapy, including psychomotor training, have beneficial effects on patients. Their study however did not take into consideration the case of comorbidity.

The aim of this study is to evaluate the effects of comorbidity (SLI, Developmental Dyslexia, ADHD) on the efficiency and choice of techniques within psychomotor training for children with DCD.

**Method:** We compared the effects of psychomotor training, carried out between 2009 and 2015, in 2 groups of children from the Midi Pyrenees region: DCD children and children at risk of DCD, with (n=21) and without comorbidities (n=9). All children were assessed with the M-ABC (manual dexterity, ball skills, balance and total impairment score) and the BHK writing test (handwriting quality, handwriting speed) before and after psychomotor therapy. The different scores were submitted to ANOVAs (2[Group] x 2[Pre-Post therapy]. Qualitative (parts of different therapeutic methods) aspects of the training were also studied. Full Scale Intelligence Quotient (FSIQ) of the Wechsler scales were also examined.

**Results:** The average duration of psychomotor therapy was the same for both groups (20 months). Significant differences between pre and post therapy were revealed for the M-ABC: Manual dexterity (p<0.001); Ball skills, Balance and Total impairment score (p<0.0001), and for the BHK quality score (p<0.0001) indicating that both groups were improved by psychomotor therapy. No significant group effect nor interaction were found for these scores. Results revealed that DCD children with comorbidity performed worse than those without comorbidity (p<0.0001) on the handwriting speed task. When considering only DCD with (n=16) and without (n=8) comorbidities, the same results were found except for the handwriting speed with no significant effect. No differences were found for the therapeutic methods used between the two groups. Finally, DCD with comorbidity had lower FSIQ than DCD without (p<0.0001).

**Discussion:** Contrary to our expectations, both groups showed significant improvement after psychomotor therapy, using perceptual-motor training and/or cognitive and metacognitive approaches. The only difference between the two groups is in the FSIQ. At a cognitive level, comorbidity plays a role, but this does not have an adverse impact on the therapeutic process. More studies are needed to confirm these findings.

**References**:

Smits-Engelsman, B. C. M., Blank, R., Van Der Kaay, A.-C., Mosterd-Van Der Meijs, R., Vlugt-Van Den Brand, E., Polatajko, H. J., & Wilson, P. H. (2013). Efficacy of interventions to improve motor performance in children with developmental coordination disorder: a combined systematic review and meta-analysis. *Developmental Medicine & Child Neurology, 55*(3), 229-237.

**Keywords:** Developmental Coordination Disorder; Comorbidity; Psychomotor therapy.

### Driving behaviour and attitudes in young adults with coordination difficulties and Developmental Coordination Disorder

H.C. Leonard^**1**^, E.L. Hill^**1**^, R. Rowe^**2**^, M.A. Waszczuk^**3**^ & A.M. Gregory^**1**^

^1^Department of Psychology, Goldsmiths, University of London, London, SE14 6NW, UK. h.leonard@gold.ac.uk; ^2^Department of Psychology, University of Sheffield, Sheffield, UK; ^3^King’s College London, MRC Social, Genetic and Developmental Psychiatry Centre, Institute of Psychiatry, Psychology and Neuroscience, London, UK.

**Aim:** The study aimed to assess driving behaviour and attitudes of individuals with coordination difficulties and DCD, investigating whether these individuals reported being more cautious and making more errors while driving than those with typical coordination.

**Method:** Data were from a longitudinal study of twin pairs and siblings, the ‘Genesis 12-19 (G1219)’ project, when participants were aged 18-32 (*N*=862). Individuals were grouped based on reports of feeling: *very*, *somewhat*, *a little*, or *not at all* uncoordinated, and their responses were compared. A group of adults with a diagnosis of DCD are currently in the process of completing the same questionnaires, and these data will also be presented. Driving behaviour was measured using items from the Manchester Driver Behaviour Questionnaire (Lajunen et al., 2004) and relevant attitudes were measured using the Attitudes to Driving Violations Questionnaire (West & Hall, 1997).

**Results:** Preliminary analyses of the questionnaire data suggest that individuals who reported feeling very uncoordinated reported more lapses during driving compared to those reporting feeling *a little* and *not at all* uncoordinated. However, these individuals did not report more errors or violations than the other groups. The *very* uncoordinated group also tended to be less risky than the *not at all* uncoordinated group, although not significantly so.

**Discussion:** These data support previous reports of individuals with poor coordination and DCD having difficulties with driving, and extend current knowledge by highlighting specific problems in terms of lapses, compared to errors or violations. While lapses are likely to represent poor execution of planned behaviour, errors are related to plans that were poorly formed. This, as well as a tendency to less risky attitudes, could help explain why individuals with poor coordination and DCD do not tend to report more accidents despite reporting more difficulties driving. The analyses of the data of adults with a diagnosis of DCD will allow better understanding of these behaviours and attitudes in individuals with clinically-significant levels of motor difficulties. As individuals with Attention Deficit-Hyperactivity Disorder also demonstrate problems with driving, and given the high comorbidity with DCD, it will be useful to compare driving profiles across diagnostic groups and in those with co-occurring disorders in future research.

**References**:

Lajunen, T., Parker, D., & Summala, H. (2004). The Manchester Driver Behaviour Questionnaire: a cross-cultural study. *Accident Analysis and Prevention*, *36*, 231–238.

West, R., & Hall, J. (1997). The role of personality and attitudes in traffic accident risk. *Applied Psychology: An International Review, 46,* 253-264.

**Keywords:** Driving; Lapses; Errors; Coordination; DCD.

### Testing a revised cut-point on the Developmental Coordination Disorder Questionnaire-07 to screen for young children at risk for DCD

Y.C. Li^**1,2**^,****S. Veldhuizen^**2**^,****C. Missiuna^**3**^, B. Timmons^**4**^ & J. Cairney^**1,2**^

^1^Department of Kinesiology, McMaster University, Hamilton, L8P 0A1, Canada. Li427@mcmaster.ca; ^2^INfant and Child Health (INCH) Lab, Department of Family Medicine, McMaster University, Hamilton, Canada; ^3^School of Rehabilitation Science, McMaster University, Hamilton, Canada; ^4^Department of Pediatrics, McMaster University, Hamilton, Canada.

**Aim:** The Developmental Coordination Disorder Questionnaire-07 (DCDQ-07) is currently one of the most widely used tools for screening for DCD in children (Wilson *et al*., 2007). We previously examined the screening potential of the DCDQ-07 in children between 4 and 6 years, and found the recommended cut-point showed relatively poor agreement against the *Movement Assessment Battery for Children version 2* (MABC2) (Parmar *et al*., 2014). We also determined that a higher threshold score could be used to improve sensitivity. The purpose of this study was to investigate the revised cut-point in a new sample of children 4 and 5 years of age.

**Method:** Young children enrolled in the Coordination and Activity Tracking in CHildren (CATCH) study comprise the study sample (n=583). A two-step procedure was used to identify children at risk for DCD (DCDr). Parents completed the DCDQ-07 by telephone. Using the revised cut-point of 55, 146 children were identified as DCDr (DCDQ-07<55), and 437 children were considered typically developing (TD) (DCDQ-07 >55). DCDr children (n=111) and a random sample of TD children (n=42) were invited to the lab to complete the MABC2. Children scoring ≤16th %ile on the MABC2 test were classified as DCDr (n=77). Receiver-Operator Characteristics Curve (ROC) analysis was used to examine agreement.

**Results:** The overall sensitivity and specificity of the DCDQ-07 against the MABC2 were 85.7% (95% CI=75.5%-92.3%) and 40.8% (95% CI=29.8%-52.7%), respectively. The ROC curve analysis indicated the area under the curve (AUC) was .707 (95% CI=.623 to.791, p<.001). In the 4-year-old group (n=77), the sensitivity and specificity were 94.4% (95% CI=80.0%-99.0%) and 26.8% (95% CI=14.8%-43.2%), respectively; and 78.0% (95% CI=62.0%-88.9%) and 57.1% (95% CI=39.5%-73.2%), respectively, in the 5-year-old group. The AUC was .682 (95% CI=.564 to .800, p<.01) for 4-year-olds, and .716 (95% CI=.592 to .840, p<.01) for 5-year-olds.

**Discussion:** Overall, our revised cut-offs improved sensitivity of the DCDQ-07 to a point acceptable for recommended screening standards. However, due to a high number of false positives and a low number of true negatives, the overall diagnostic accuracy of the test is poor to fair. Even with a revised cut-point, we cannot recommend the DCDQ-07 for general screening for this developmental period. In situations where maximizing sensitivity is the goal (e.g. recruitment for research; two-step screening procedures), our revised cut-point can be recommended for use in this age group.

**References**:

Parmar, A., Kwan, M., Rodriguez, C., Missiuna, C., & Cairney, J. (2014). Psychometric properties of the DCD-Q-07 in children ages to 4-6. *Research in Developmental Disabilities, 3*5(2), 330-339.

Wilson, P. H., Kaplan, B. J., Crawford, S. G., & Roberts, G. (2007). *The Developmental Coordination Disorder Questionnaire 2007*. Calgary, Canada: Alberta Children’s Hospital Decision Support Research Team.

**Keywords:** Early Childhood; DCDQ-07; Screening; Assessment.

### Is the Movement Assessment Battery for Children – 2nd edition (MABC-2) valid for Brazilian children 4 to 8 years-old? A comparison between Brazil and UK

L.C. Magalhães^**1**^, C. Linhares^**2**^, A.L. Barnett^**3**^, B.L.C. Moraes^**4**^, C.G. Silva^**5**^, O.S. Agostini^**6**^ & A.A. Cardoso^**7**^

^1^Departamento de Terapia Ocupacional, Universidade Federal de Minas Gerais, Av. Antônio Carlos 6627, Belo Horizonte, MG, 31270-901, Brazil. liviacmag@gmail.com; ^2^Curso de Terapia Ocupacional, Instituto Federal do Rio de Janeiro, Rio de Janeiro, Brazil; ^3^Department of Psychology, Social Work & Public Health, Oxford Brookes University, Oxford, UK; ^4^Curso de Terapia Ocupacional, Universidade Federal de Minas Gerais, Belo Horizonte, Brazil; ^5^Departamento de Terapia Ocupacional, Universidade Federal de Pelotas, Pelotas, Brazil; ^6^Departamento de Terapia Ocupacional, Universidade Federal do Rio de Janeiro, Brazil; ^7^Departamento de Terapia Ocupacional, Universidade Federal de Minas Gerais, Belo Horizonte, Brazil.

**Aim:** The MABC-2 is being used in Brazil and although there are two validity studies, none of them compared actual data. The aim of this study was to compare the performance of children ages 4 to 8 years old from Brazil (BR) and the United Kingdom (UK) on individual items of the motor test component of the Movement Assessment Battery for Children 2nd edition (MABC-2).

**Method:** 887 typically developing children (396 BR; 491 UK) stratified by age and gender were assessed with the MABC-2. Data on UK children were obtained from the MABC-2 normative data set. Brazilian children were recruited and assessed in public and private schools, according to standardized procedures. Means and standard deviation were calculated for the raw score of each individual item for the ages 4, 5, 6,7, and 8 years old. ANOVA was used for group comparison (p≤.05).

**Results:** There was no significant age difference between groups on each age group. Concerning motor performance, there were scattered differences in means for different items and ages. On manual dexterity, UK children were better on Placing Pegs with preferred hand at age 7 (p=.001) and they committed less mistakes on the Drawing Trail at ages 5 (p=.001), 6 (p=.001), 7 (p=.016), and 8 (p=.007); there were no differences on the Threading tasks, and 7-y-old BR children were better on Placing Pegs with non-preferred hand (p=.002). On Ball Skills, there were no significant differences on Catching, but UK children were better on Throwing at ages 7 (p=.001) and 8 (p=.001). There were no differences on the Jumping and Hopping tasks, but 6-y-old BR were better on preferred (p = .001) and non-preferred leg (p=.001) One-Leg Balance, and UK were better on Heel-to-Toe walking at ages 6 (p=.004) and 7 (p=.023). Further analysis will investigate gender and socioeconomic issues.

**Discussion:** There were differences in several motor items at different ages and, overall, UK children tended to outperform Brazilians when differences were found. These differences could be related to cultural as well as to socioeconomic factors as half of the Brazilian sample was recruited in public schools, which have higher representation of children from socially disadvantaged backgrounds. Although Brazilians are recognized for their ball skills, they did not perform better on the MABC-2, maybe because most children are not familiar with the tennis ball, used in the test. The impact of these differences on the MABC-2 cut off scores will be discussed.

**References**:

Henderson, S. E., Sugden, D. A., & Barnett, A. L. (2007). *Movement assessment battery for children 2nd ed* (MABC-2). London: Harcourt Assessment.

**Keywords:** MABC-2; Children; Motor coordination; Cross cultural study.

### DCD: a failure to control the unexpected

M. Martel^**1,2**^, P. Fourneret^**3,4**^, L. Finos^**5**^, M. Vernet^**6**^, M.-C. Thiollier^**6**^, C. Schmitz^**2**^ & A.C. Roy^**1**^

^1^DDL, CNRS UMR5596, Université de Lyon, Lyon, France. marie.martel@isc.cnrs.fr; ^2^CRNL, Dycog Team, INSERM U1028 - CNRS UMR5292, Université de Lyon, Bron, France; ^3^Service Psychopathologie du Développement, HFME, Hospices civils de Lyon, France; ^4^L2C2, UMR5304, CNRS, Institut des Sciences Cognitives, Université de Lyon, Bron, France; ^5^Università di Padova, Padova, Italy; ^6^Réseau Dys/10, Lyon, France.

**Aim:** Developmental Coordination Disorder is characterized by poor motor skills. Motor control relies on two modes of control, a feedforward mode that anticipates the goal and system characteristics and a feedback mode that corrects on-line the residual errors. To determine the nature and the specificity of motor deficits in DCD, we examine both feedforward and feedback modes in Typically Developing (TD) children aged from 5 to 10 and in DCD children aged 9-10 years old.

**Method:** Children were required to reach and grasp an object in order to displace it to a lateral location. The objects, two visually identical opaque bottles, could be heavy or much lighter. We manipulated the child’s previous knowledge of the object weight: when known, participant could anticipate the consequences of the weight when reaching for the object, prior to contact with it, thus allowing for feedforward control. Conversely, when unknown prior to contact, participants had to cope with the object weight after contact (feedback control), in the displacing phase of the movement. Movement kinematics was recorded with a high-resolution optoelectronic motion tracking system.

**Results:** TD children were able to use weight information beforehand as early as 5 years old: they displayed later and smaller reaching parameters (i.e. wrist acceleration, velocity and deceleration) for heavy objects. More efficiently, 7- to 10-year-old children exhibited the opposite pattern to overcome weight effects (shorter latencies and higher peaks for heavy objects in the reaching phase). When unknown, the heavy object impacted the displacing phase only by slowing down the movement in 5- and 10-year-old children alike, whereas children aged 7-8 years old were less performant in applying online corrections. Preliminary results obtained on 10-year-old DCD children revealed an overall important slowness throughout the reaching and displacing phases. However, when required to reach for known heavy objects, DCD children tended to use the same strategy as 10-year-old TD children (e.g. going faster for a heavy object). In contrast, in the unknown weight condition, adjusting to the unexpected weight was time-consuming for DCD children.

**Discussion:** Our findings reveal that healthy children use a feedforward mode of control as young as 5 years of age, yet this control becomes optimal around 7 years old. This reorganization of motor control at 7 years old is further supported by the poor performance of the TD children aged 7 when online control was maximally taxed. Despite an important general sluggishness, DCD children tended to appropriately anticipate their movements as a function of the upcoming object weight, whereas their feedback control was affected. These findings shed light on the specificity of motor impairments in DCD, suggesting possible new avenues for rehabilitation approaches.

**Keywords:** Motor control; Feedforward; Feedback; Development; Kinematic.

### Intervention and support in DCD: from research to practice

V.A. McQuillan^**1,2**^, M.E. Chambers^**1**^, D.A. Sugden^**1**^ & R.A. Swanwick^**1**^

^1^School of Education, University of Leeds, LS2 9JT, UK. vickym@liv.ac.uk; ^2^School of Health Sciences, University of Liverpool, UK.

**Aim:** This project originates from our intervention studies, our current work examining children with DCD and the effects of associated difficulties over time, and work in the sporting arena notably “the aggregation of marginal gains”. Studies have shown that some children with DCD, with or without intervention, improve and sustain their motor performance, but it has been difficult to predict which children do this. Many children with DCD have associated difficulties of attention, language, social interaction and learning. However, few studies have examined the nature of their interaction and effect on outcomes over time. This paper firstly examines profiles of children with DCD with and without associated characteristics aged 8-16 years in relation to participation in functional outcomes over time. Secondly, implications for intervention are made based upon the dynamic variables noted.

**Method:** Children with DCD are identified according to DSM V. Assessment measures include the MABC2 to measure motor proficiency; DCDQ to assess the impact of motor ability on daily activities; CSAPPA to measure the children’s enjoyment of physical activity; CCC2 to indicate any pragmatic or other language difficulties. Children are monitored and assessed over a 24-month period with comparisons made between those with DCD, those with DCD plus associated characteristics and a group of typically developing peers. Some children, their parents and teachers are interviewed to provide case studies showing the nature of change.

**Results:** Four data points over two years are being analysed and the first two data points show the motor proficiency pattern and impact on participation, function and enjoyment for children with DCD and those with DCD plus associated characteristics and their typically developing peers. Case studies of children are presented identifying those that improve, those that remain the same and those that deteriorate, together with the dynamic variables affecting these outcomes.

**Discussion:** Intervention should recognize the total resources the child brings to the movement situation. We recommend an ecological perspective employing the concept of the aggregation of marginal gains, whereby several targeted areas in a child’s life are slightly modified by different individuals to improve their function in activities of daily living. The totality of these marginal gains leads to an overall improvement in the child’s participation and performance as illustrated in the case studies.

**Keywords:** DCD; Associated characteristics; Change; Intervention.

### The effect of a quiet eye training intervention on self-perceptions of adequacy, enjoyment and predilection of physical activity in children with DCD

C.A.L. Miles^**1,2**^, G. Wood^**2**^, S.J. Vine^**1**^, J.N.Vickers^**3**^ & M.R. Wilson^**1**^

^1^Department of Sport and Health Science, University of Exeter, St. Luke’s Campus, Heavitree Rd, Exeter, EX1 2LU, UK. milesc@hope.ac.uk; ^2^Department of Health Sciences, Liverpool Hope University, Hope Park, Liverpool, UK; ^3^Faculty of Kinesiology, University of Calgary, 2500 University Drive NW, Calgary, AB, Canada.

**Aim:** Children’s Self-Perceptions of Adequacy and Predilection for Physical Activity questionnaire (CSAPPA^1^) was used to assess the efficacy of a quiet eye training (QET) intervention for a throw and catch task in children with Developmental Coordination Disorder (DCD). The CSAPPA measures a child’s attitude to physical activity on a four point scale and has been validated as screening tool for DCD^2^.

**Method:** 30 children diagnosed with DCD completed the CSAPPA before being randomly allocated into a Traditional Training (TT) or QET intervention group. The training for both groups consisted of a single 60min session that included 3 short instructional videos based on national curriculum teaching strategies each followed by a set of catching practices. The QET group received additional emphasis relating to their gaze behaviour. Participants completed the CSAPPA immediately post-training and again 6-weeks later.

**Results:** A 2x3 mixed model ANOVA determined there was a significant interaction effect between test occasion and intervention group in perceived adequacy (*p* = .025). This effect was not observed in enjoyment (*p* = .728) or predilection (*p* = .629). The largest difference in scores was recorded immediately post-training where children who received QET reported higher levels of perceived physical activity adequacy, although this difference only approached significance (*p*= .096).

**Discussion:** QET increased perceived adequacy towards physical activity in children with DCD, which may be an important factor in encouraging these children to become more active.

**References**:

1. Hay, J. (1993). Predictive validity of the CSAPPA scale: A longitudinal investigatioin, *Peadiatric Exercise Sceince, 5,* 427.

2. Cairney, J., Veldhuizen, S., Kurdyak, P., Missiuna, C., Faught, B. E., & Hay, J. (2007). Evaluating the CSAPPA subscales as potential screening instruments for developmental coordination disorder, *Archives of Disease in Childhood, 92*(11), 987-991.

**Keywords:** Developmental Coordination Disorder; Perception; Intervention; QET; Physical activity.

### Innovations in management of Developmental Coordination Disorder in the schools: What does it take to shift occupational therapy practice?

C. Missiuna^**1,2**^, N. Pollock^**1,2**^, S.S. Whalen^**2**^, L. Dix^**1,2**^ & D. Stewart^**1,2**^

^1^School of Rehabilitation Sciences, McMaster University, 1400 Main Street West, Hamilton, ON, L8S 1C7. missiuna@mcmaster.ca; ^2^CanChild Centre for Childhood Disability Research, 1400 Main Street West, IAHS 408, Hamilton, ON.

**Aim:** In the Partnering for Change (P4C) implementation study, occupational therapists (OTs) are shifting practice for children with Developmental Coordination Disorder (DCD) from a direct, one-to-one, intervention model targeting motor impairment to a population-based approach, P4C builds the capacity of families and educators to promote the child’s successful participation and long-term health. OTs work collaboratively with educators in the classroom using principles of universal design for learning to create accessible curricula and environments; differentiate instruction for children experiencing challenges; and provide individual strategies for children who require accommodation. This significant shift in practice is challenging for OTs. The aim of this poster is to describe the strategies that the research team has used to support this change.

**Method:** Fifteen OTs are delivering P4C in 40 schools. Strategies to promote the shift in practice and to ensure fidelity to the P4C service delivery model have included: 1) targeted recruitment of therapists using self-evaluation; 2) training workshops; 3) completion of a series of online learning modules regarding DCD and co-morbidities, health promotion, collaborative consultation, capacity building and sustainability; 4) mentorship from expert practitioners. OTs completed pre-post knowledge questionnaires and participated in focus groups and interviews to enable understanding of their transition to this new model of care.

**Results:** Strategies aimed at developing OTs knowledge, skills and confidence have supported OTs’ transition to a capacity-building model for children with DCD. Pre-post questionnaires showed a significant increase in knowledge and perceived competence in many skill areas. Qualitative analysis of interview and focus group transcripts provided insight into the process of change and revealed OTs’ increased satisfaction with this model of service. Although OTs perceived the shift in practice to be challenging, belief in the effectiveness of the new model supported change.

**Discussion:** Making the shift to a very different model of intervention for children with DCD is a complex process. The research team used varied strategies to study and support the development of new knowledge and skills. Project findings support the need for strong leadership, ongoing mentorship and professional development opportunities to facilitate successful change in management for children with DCD.

**Keywords:** Intervention; Occupational therapy; Clinician education; Mentorship; Innovation.

### Adults with high functioning autism do not use vision for postural control

S. Morris, C.J. Foster, R. Parsons, M. Falkmer, T. Falkmer & S.M. Rosalie

Faculty of Health Sciences, Curtin University, Perth, Western Australia, 6102, Australia. s.morris@curtin.edu.au

**Aim:** Despite primarily a social disorder, most individuals with ASD also report difficulty with fundamental motor skills^1,2^. The development of motor skill relies on postural control and along with social skills is facilitated by the active use of visual information. The use of visual information for postural control in ASD has not been reported. The purpose of this study was to compare how adults with ASD and typically developed adults (TDA) use visual information to control posture during quiet standing.

**Method:** The study used intermittent (15off, 5on) posterior neck vibration during 100 seconds of quiet stance to induce a postural illusion. In typically developed adults and only in the absence of vision this protocol induces a forward body lean. Participants (12ASD, 20 TDA) undertook four conditions combining vibration and visual occlusion.

**Results:** Significant main effects were observed for group, *F*(1, 2355) = 5.50, *p*<.05, vibration illusion *F*(1, 2355) = 232.26, *p*<.0001, and visual occlusion condition *F*(3, 2355) = 4.22, *p*<.01. For both ASD and TDI the magnitude of postural movement during vibration under visual occlusion was in the order of between 7.8 and 10.2 mm. A significant interaction was also observed between group and visual occlusion condition *F*(3, 2355) = 10.11, *p*<.0001. The ASD group leaned forwards more than the TDI group when vision was either fully or partially available (EO-EO, EO-EC, EC-EO p<0.01); whereas, there was no difference between groups when vision was not available (EC-EC t=0.63 p=0.527). There were no differences in the postural position of the ASD group regardless of whether vision was fully, partially or not available (p>0.0335).

**Discussion:** Our findings indicate that the individuals with ASD do not use visual information to control standing posture. In light of evidence of that vision-for-perception is processed typically in ASD, our findings support a specific deficit in vision-for-action. These findings may explain why individual with ASD experience difficulties with both social and motor skills since both require vision-for-action. Further research needs to investigate the division of these visual learning pathways in order to provide more specific intervention opportunities in ASD.

**References**:

1. Ghaziuddin, M., & Butler, E. (1998). Clumsiness in autism and Asperger syndrome: a further report. *Journal of intellectual disability research: JIDR, 42*, 43-48.

2. Pan, C. Y., Tsai, C. L., & Chu, C. H. (2009). Fundamental movement skills in children diagnosed with Autism Spectrum Disorders and Attention Deficit Hyperactivity Disorder. *Journal of Autism and Developmental Disorders, 39*(12), 1694-1705.

**Keywords:** Autism; Vision; Vision for action; Postural control; Movement disorders.

### The Development of the Japanese version of the Adult Developmental Co-ordination Disorders/Dyspraxia Checklist (ADC)

A. Nakai^**1**^, K. Takayama^**2,3**^, M. Ohnishi^**4**^, Y. Mitsuhashi^**4**^ & A. Kirby^**5**^

^1^Hyogo Children’s Sleep and Development Medical Research Center, Kobe, 651-2181, Japan. anakai.kodomo@gmail.com; ^2^Nonprofit Organization Edison Club, Iruma, Japan; ^3^Graduate School of Pharmaceutical Sciences, Showa University, Tokyo, Japan; ^4^Faculty of Education and Regional Studies, University of Fukui, Fukui, Japan; ^5^Developmental Disorders in Education, The Dyscovery Centre, University of Wales, UK.

**Objectives:** Prevalence of developmental coordination disorder (DCD) is 6~10%. Until recently, it is believed that motor impairments in children were improved when children grew out. However, problems with coordinated movements in childhood continue through adolescence to adults in an estimated 50~70% of children, as described in DSM-5. Although there is no assessment tool, in the world, for screening DCD in adult, the adult developmental co-ordination disorders/dyspraxia checklist (ADC) has been developed by Kirby et al. (2010). The ADC has been translated into Hebrew, Dutch, Flemish and Taiwanese. Previous studies showed the high prevalence of comorbidity of DCD and AD/HD, at 30~50%, with poor psychosocial functioning. We have reported that all subscales and total scores of the DCDQ-J were significantly associated with the total and each subscales scores of ADHD-RS-J in the Japanese Children.

**Method:** We developed the Japanese version of the ADC (ADC-J) with the international collaborative study. The ADC consists of three subscales; the first relates to difficulties that the individual experienced as a child (subscale A-10 items) while the second (B-10 items) and third subscales (C-20 items) relate to current difficulties that the individual considers affect his performance. The questionnaire was translated according to the guidelines for cross-cultural translations of instruments (i.e., translation, back-translation and analysis by an expert committee). The adapted ADC-J has been administered to Japanese adults, as the pretest. We also employed the Japanese version of Adult ADHD Self-Report Scale (ASRS-v1.1).

**Results:** Data were collected from 360 Japanese adults (181 males; mean age 37.5, SD=5.77 ranged from 25 to 66, 179 female; mean age 35.8, SD=4.78 ranged 24 to 47). Cronbach’s coefficient alpha was calculated for the total and each of the three ADC subscales, and these are enough high as the original ADC, total 0.924, Subscale A 0.813, Subscale B 0.814, and Subscale C 0.886, respectively. Score of ADC-J and the scores of the Japanese version of ASRS were well correlated.

**Discussion:** The Japanese version of the ADC is expected to be a useful screening instrument to identify and assess motor coordination difficulties of adults in Japan, and it enables the cross-cultural comparison.

**Acknowledgement:** This study was supported, in part, by Scientific Research from the Japan Society for the Promotion of Science.

**Reference**:

Kirby, A., Edwards, L., Sugden, D., & Rosenblum, S. (2010). The development and standardization of the Adult Developmental Co-ordination Disorders/Dyspraxia Checklist (ADC). *Research in Developmental Disabilities, 31*, 131–139.

**Keywords:** Adult DCD; The adult developmental co-ordination disorders/dyspraxia checklist (ADC); Adult ADHD Self-Report Scale (ASRS); Comorbidity.

### Gait pattern evaluation in children with DCD

M. Nunzi^**1**^, M. Galli^**2**^ & G. Albertini^**1**^

^1^Department of Pediatric Neurorehabilitation, IRCCS San Raffaele Pisana, Rome, 00163, Italy; michela.nunzi@libero.it; ^2^Bioengineering Department, Politecnico di Milano, Milano, Italy.

**Aim:** The aim is to study the way of walking of children affected by Developmental Coordination Disorder through the Gait Analysis (GA) method.

**Method:** We analyzed 3 groups of 10 children each. The first group was populated with10 children affected by Developmental Coordination Disorder (DCD), the second group with children affected by Down Syndrome (DS) and the last group with children showing a Typical Development (TD). A clinical (orthopaedic and neurological) evaluation was performed on the children of all 3 groups. First of all the whole study population underwent a purely qualitative analysis of their gait (video recording). Subsequently, they were analyzed through the use of the two following measurement instruments: reflective skin markers were positioned on the patients following the Davis Protocol. A 8-Camera Optoelectronic System (ELITE, BTS) was used to capture the movements in order to reconstruct the 3D coordinates. Two force platforms (KISTLER) measuring the ground reaction forces during the walking. DCD is diagnosed to children with movement disorders: difficulties of coordination in various motor tasks (coarse and/or fine). Locomotion is an important milestone for the optimal development of the child. For this reason, a considerable amount of researches about the quality of locomotion, gait and standing, involved patients with Cerebral Palsy (PY) and Down Syndrome (DS), (e.g., Damiano & Abel, 1996; Massaad, Dierick, van den Hecke, & Detrembleur, 2004; Sutherland, 1978). It might be interesting to analyze the intrinsic characteristics of gait, locomotion and standing in children affected by DCD. To understand the underlying mechanism of the disease, it might be also interesting to compare gait, locomotion and standing of children affected by DCD with those affected by DS.

**Results:** The preliminary data show that children with DCD have a specific gait profile. The report stated that DCD patients, compared to TD patients, have an increased latency for both Distance and Temporal Gait Parameters (time stance and swing time, stride time). Distance and Temporal Gait Parameters were also different between the two legs. DS walking is characterized by a ‘’Chaplinesque’’ pattern and it shows similar temporal features of the DCD children. Both can have flat feet.

**Discussion:** The similarity of some intrinsic motor features between DCD and DS opens interesting questions about the aetiology of the DCD. These are still preliminary data but by July presumably this will be flushed out.

**References**:

Damiano, L.D., & Abel, M.F. (1996). Relation of gait analysis to gross motor function in cerebral palsy. *Developmental Medicine and Child Neurology, 38,* 389-396.

Galli, M., Albertini, G., Crivellini, M., & Tenore, N. (2001). Gait analysis in children with DS. *Progress Report- International Review of Medical Science, 13*, 21-28.

Massad, F., Dierick, F., Heck, A., & Detrembleur, C. (2004). Influence of gait pattern on the body’s centre of mass displacement in children with cerebral palsy. *Developmental Medicine & Child Neurology, 46*, 674–680.

Sutherland, D. H., & Cooper, L. (1978). The pathomechanics of progressive crouch gait in spastic diplegia. *Orthopedic Clinics of North America, 9*, 143-154.

**Keywords:** DCD; Gait analysis; Locomotion.

### The “Make My Day” evaluation tool aimed to gather information about daily routines from a young child and his parents- construct validity

L. Or & T. Ricon

Department of occupational therapy, The University of Haifa, 31905 Israel. lironor@gmail.com

**Aim:** This research verified and examined the reliability of a new tool, named “Make My Day”, whose objective is to provide information on the daily routine of a child directly from the child and from his/her parents. The tool studies the child’s daily routine, the quality of his/her performance of everyday activities, the level of his/her independence in performing these activities and satisfaction from the performance. In addition, the tool enables studying the degree of correspondence between the reports of the child and the parents. The evaluation tool “Make My Day” was developed with the aim of meeting the need for an up to-date evaluation tool that enables obtaining information on the daily routine of a child aged 4 – 7, adapted to his/her level of development, from the child and from his/her parents. The tool is based on the “Family-Centered” approach, which has become the preferred approach amongst occupational therapists in recent years and promotes the involvement of the client and his/her family in the entire therapeutic process, from the initial evaluation, through the treatment program, to the final assessment of the outcome.

**Method:** Verification and reliability assessment will be performed by comparing “Make My Day” with other evaluation tools. The studied population comprised 75 normative Jewish children from the Jerusalem area, aged 4 – 7, for whom the parents’ consent to participate in the research was obtained in advance. In the analysis of the results, a number of details, regarding which under 10% of the participants responded, were removed and appeared as missing data. In addition, some of the statistical analysis was performed on a larger sample of children that included data from a preceding study of the tool that was conducted with normative 4 – 7 year-old Arab children.

**Results:** The results of the research indicate that “Make My Day” is a tool that enables collecting data from children as young as 4 on the basis of their own and their parents’ reports regarding activities that comprise their daily routine; this is based on correlation with CPQ and PEGS questionnaires. The internal reliability of all the research variables, tested on the entire sample (Jews and Arabs), was found to be medium and above, with α=0.65 – 0.89. The internal reliability within the current sample (Jews) was also found to be medium and above, with α=0.55 – 0.93. In the validity test, medium to high correlation was found between the evaluation tool “Make My Day” and the PEGS evaluation tool, as expressed by an analysis of the results of the current sample for the children’s version (r = 0.30 – 0.65) and for the parents’ version (r = 0.28 – 0.58). Likewise, medium to high correlation was found between the evaluation tool “Make My Day” for the parents’ version and the CPQ evaluation tool (r = 0.10 – 0.60). The findings of the current research, similarly to the preceding study performed amongst the Arab population, indicate differences between the children’s and the parents’ reports, in that the children assess their abilities as better than their parents’ assessment of them.

**Discussion:** The evaluation tool “Make My Day” has been found, in the preliminary studies conducted in the preceding research within the Arab population (Ricon, Hen & Keaden-Hardan, 2013) and in the current research, efficient for the purpose of research and, in the future, for clinical use (with various of clinical population such as DCD). With this tool it is possible to obtain reports from the child and his/her parents regarding his/her capabilities in performing the everyday tasks that make up his/her routine from a pleasant conversation adapted to his/her level of development. The findings from similar research projects have indicated greater success in therapy when the children are actively involved in the therapeutic process and in determining the objectives of the treatment themselves and in cooperation with them.

**References**:

Missiuna, C. & Pollock, N. (2000). Perceived efficacy and goal setting in young children. *Canadian Journal of Occupational Therapy, 67*, 101-109.

Rosenberg, L., Jarus, T. & Bart, O. (2010). Development and initial validation of the Children Participation Questionnaire (CPQ). *Disability and Rehabilitation, 32*(20), 1633-1644.

**Keywords:** Daily routines; “Make my day”; Evaluation; Young children.

### Normative data for lower limb peak mechanical power in children aged 7 to 11 years old

N.J. Owen^**1**^, W. Griffiths^**2**^ & J. Watkins^**1**^

^1^Applied Sports Technology, Exercise and Medicine Research Centre, Swansea University, Swansea, SA2 8PP, UK. n.j.owen@swansea.ac.uk; ^2^Neath Port Talbot Education Authority, Civic Centre, Port Talbot, UK.

**Aim:** There are currently about 45 published instruments for the assessment of motor development in children. However, all of the tests lack robust evidence of reliability and validity^1^. A high levels of lower limb peak muscular power (Pmp) is widely considered a key determinant of athletic performance^2^; conversely it is reasonable to assume that poor physical performance, characterised by poor coordination, would be associated with low levels of Pmp. However there are currently limited valid data on normative values of Pmp in children. The aim of this study was to report valid normative data for Pmp in children.

**Method:** Children 7 to 11 years old (n= 791, age 9.26 ± 1.20 decimal years, stature = 1.338 ± 0.094 m, body mass = 34.7 ± 9.7 kg) of mixed gender were randomly selected from schools in South Wales. Each child performed one countermovement jump (CMJ) off a force platform with their hand held on their hips to isolate the lower limbs. The ground reaction force was recorded and the momentum impulse principle was used to derive a criterion measure of lower limb peak instantaneous power (Pp)3. A pilot study had shown good reliability for Pp in children in this age range (ICC > 0.92). Participants were grouped in school years (Y) and comparisons were made between genders and year groups for Pp.

**Results:** There was no significant difference in Pp between genders for year group (p = 0.05). Combined gender groups for each school year produced Pp that were normally distributed and had the following values (mean Pp, standard deviation), Y3 = 905 ± 191 W, Y4 = 1047 ± 233 W, Y5 = 1230 ± 258 W, Y6 = 1367 ± 326 W. A significant difference in Pp was found between successive mixed gender year groups (p= 0.01). Y3 (n= 190) to Y4 (n = 182), t = 6.01, p < 0.001, Y4 to Y5 (n = 215), t = 7.67, p < 0.001, Y5 to Y6 (n = 204), t = 3.94, p < 0.001.

**Discussion:** This study indicates that Pp produced in a single-trial CMJ has the potential to provide information on the coordination of children aged 7 – 11 years, with a high level of discrimination. For example, in Y3 (aged 7-8 years) there is a 12% increase in Pp between the 5th and 10th percentile. Pp as measured in a CMJ has the potential to augment tests like Movement Assessment Battery for Children-2. However more study is needed regarding the potential benefits of allometric scaling of Pp and further splitting school year groups into 3 month groups and comparison with other tests of physical ability.

**References**:

1. Brown, T., & Lalor, A. (2009). The Movement Assessment Battery for Children - Second Edition (MABC-2): a review and critique. *Physical & Occupational Therapy in Pediatrics, 29*(1), 86-103.

2. Cronin, J. B., & Hansen, K. T. (2005). Strength and power predictors of sports speed. *Journal of Strength and Conditioning Research, 19*, 349–357.

3. Owen, N. J., Watkins, J., Kilduff, L. P., Bevan, H. R., & Bennett, M. A. (2014). Development of a criterion method to determine peak mechanical power output in a countermovement jump. *Journal of Strength and Conditioning Research, 28*(6), 1552-1558.

**Keywords:** Power; Coordination; Vertical jumping; Children; Force platform.

### Specificity of motor abnormalities in DCD in the following children with Autism Spectrum Disorder using a standardized neuro-psychomotor assessment

A. Paquet^**1,3,4**^, B. Olliac^**4,5**^, B. Golse^**2,3,6**^ & L. Vaivre-Douret^**2,6,7**^

^1^Department of Psychology, University of Paris Descartes, Sorbonne Paris City 75006, Paris. France aude.paquet@etu.parisdescartes.fr; ^2^Department of Medicine, University of Paris Descartes, Sorbonne Paris City, Paris, France; ^3^INSERM, UMR 1178, University of Paris Sud and Paris Descartes, Paris, France; ^4^University Pole of Psychiatry of the Child and the Teenager, Limoges, France; ^5^UMR 1094, University of Limoges, Limoges, France; ^6^Department of Child Psychiatry, AP-HP Necker-Enfants Malades University Hospital, IMAGINE affiliation, Paris, France; ^7^Department of Pediatrics, AP-HP Paris Center Port Royal-Cochin Hospital, Paris, France.

**Aim:** Decreased motor performance was described in Autism Spectrum Disorders (ASD) with a disturbance in walking, posture, coordination or arm movements. There is evidence in favour of comorbidity between Developmental Coordination Disorder (DCD) and ASD, including impairment of voluntary actions or locomotion. Never the less some individuals with ASD show no decrease of the driving performance. Although there are difficulties in defining the DCD in the studies in children with ASD, it is estimated that there is a high prevalence of classic motor symptoms of DCD in these children. Objective: To highlight the semiology of movement disorders among children with ASD, using a neurodevelopmental assessment tool.

**Method:** Thirty-five children with ASD are recruited in a child psychiatry service (Limoges) and Autism Resource Centers (Bordeaux, Limoges, Toulouse). Evaluations of the first instances (psychiatric/ ADI; psychological/ KABC-II/ Rey’s figure/ London tower; understanding/NEEL; psychomotor/ M-ABC) were supplemented by a standardized assessment battery of neuro-developmental psychomotor functions (NP-MOT).

**Results:** From the NP-MOT test, the tests of two-hand and finger praxis are largely failed(33% and 60%). Manual and digital gnosopraxis are deficient (72% and 65%). Postural deficit was found in both the static equilibrium tests (60%) and dynamic (53%). There is also the difficulties of coordination between the upper and lower limbs in 55% of children. We find a failure to the M-ABC (79%). Spatial orientation is deficient in 47% and visuospatial structuring in 56% of cases. Children with Asperger’s Syndrome (AS) were better on perceptual-visuo-spatial tests.

**Discussion:** We can emphasize in the light of the results of manual and digital gnosopraxis tests a planning deficit in children with autism. A gesture programming deficit is also highlighted by the poor results in the tests of two-hand praxis NP-MOT, by failure to M-ABC tests despite the training of the child and by the visuospatial test. These planning and programming difficulties gesture increase a mixed type of dyspraxia (ideomotor/visiospatial and constructive) of this DCD in children with ASD. However it appears that children with AS have a better overall motor coordination and we did not notice any perceptual and visuospatial disorder in these children. And children with AS would present a particular type of ideomotor typology of DCD. The use of neurodevelopmental tool allows to refine the semiology of motor abnormalities in the DCD.

**References**:

Paquet, A., Olliac, B., Golse, B., & Vaivre-Douret, L. (2014). *Etat des connaissances sur les troubles moteurs des enfants porteurs de Trouble du Spectre Autistique et apport de l’évaluation neuro-psychomotrice standardisée*. Les Entretiens de Bichat, Paris.

Vaivre-Douret, L. (2006). *Batterie d’évaluation des fonctions neuro-psychomotrices de l’enfant*. Paris: ECPA.

Vaivre-Douret, L. (2014). Developmental coordination disorders: State of art. *Neurophysiologie Clinique/Clinical Neurophysiology, 44*(1), 13-23.

**Keywords:** Autism Spectrum Disorders; Motor skills; Neuro-psychomotor functions; Developmental Coordination Disorder.

### Adaptation and preliminary testing of the developmental coordination disorder questionnaire (DCD-Q’07) for children in India

P. Patel^**1**^, C. Gabbard^**1**^ & B.N. Wilson^**2**^

^1^Department of Health and Kinesiology, Texas A&M University, USA. priya_20@tamu.edu; ^2^Department of Pediatrics, University of Calgary, Canada

**Aim:** Whereas Developmental Coordination Disorder (DCD) has gained worldwide attention among the medical and research communities, in India it is relatively unknown. One of the most utilized screening tools is the revised DCD-Questionnaire (DCD-Q’07)^1^. Our aim was to translate the instrument into Hindi language (DCDQ-I), test its basic psychometric properties, and provide a preliminary report of probable DCD prevalence in India based on its use.

**Method:** The translation and back- translation of the DCDQ-I was conducted using a group of outside experts and guidelines developed by Beaton *et al.* (2000)^2^ for cross cultural adaptation of instruments. To determine the psychometric properties of the DCDQ-I and probable prevalence of DCD, parents of 955 children ages 5- to 15 years, representing 5 public schools in Western India were involved in the study. Of the 955, 60 were retested randomly after 3 weeks from initial screening for test- retest reliability. The total data set was divided into 3 age groups (years 5 – 7, 8 – 10, and 11 – 15). Internal consistency was examined using Cronbach’s α and corrected item-total correlations. Confirmatory factor analysis (CFA) was performed based on the DCD-Q’07 3-factor solution to test structure of the DCDQ-I.

**Results:** Mean scores across three age groups ranged from 49 to 54 with 74 the maximum score. Cronbach’s α was high (α = 0.86) and 14 of 15 items showed moderate correlation values ranging from 0.40 to 0.59. Test- retest results indicated acceptable reliability (0.73). CFA results indicated that the 3-factor model, namely, ‘Control during Movement’, ‘Fine Motor/ Handwriting’, and ‘General Coordination’ was the best model for the DCDQ-I. The % probable DCD using the DCD-Q’07 cutoff scores of ≤ 57 ranged from 22- to 68%. When using more stringent cutoffs of ≤ 36, percentages were reduced substantially to 5- to 9%. One-way ANOVA and *t* test results for age group and gender effects respectively showed no significant differences. Two-way ANOVA with percentage scores of the DCDQ-I subsets (namely: Control During Movement, Fine-Motor/ Handwriting, General Coordination) considering effect of gender showed significant difference between boys and girls, *p* = 0.01, with boys performing significantly better than girls in subset 1.

**Discussion:** The DCDQ-I reveals promise for initial identification of Hindi speaking Indian children for DCD. Confirmatory factor analysis indicated that the instrument is compatible with the DCD-Q’07. The relatively high difference in the estimated prevalence rates as predicted by two different cut off scores suggest that the DCD-Q’07 cut off scores should be modified in order to be more sensitive in predicting those children at risk for DCD. In terms of gender effect, results were not significant for total scoring, but were significant for subset scores; findings similar to that of the DCD-Q’07. Based on our preliminary findings using the more stringent cut-off scores, the *‘probable prevalence’* of DCD in India appears to be around 6 – 7%; numbers similar to those in the USA for those diagnosed with DCD. Research with a larger sample and comparison with the MABC-2 or equivalent is needed.

**References**:

1. Wilson, B. N., Crawford, S. G., Green, D., Roberts, G., Aylott, A., & Kaplan, B. J. (2009). Psychometric properties of the Revised Developmental Coordination Disorder Questionnaire. *Physical & Occupational Therapy in Pediatrics, 29*(2), 182-202.

2. Beaton, D. E., Bombardier, C., Guillemin, F., & Ferraz, M. B. (2000). Guidelines for the process of cross-cultural adaptation of self-report measures. *Spine*, *25*(24), 3186-3191.

**Keywords:** DCD; DCD-Q’07; India; Children.

### The use of the Movement Assessment Battery for Children (MABC) in Brazil: a literature review

C.L. Pinheiro^**1**^, A.P. Silva^**1**^, A.A. Cardoso^**2**^ & L.C. Magalhães^**2**^

^1^Occupational Therapy Course, Federal Institute of Rio de Janeiro, Rua Professor Carlos Wenceslau, 343, Realengo, Rio de Janeiro, RJ, 25715-000, Brazil. carolinnelinhares@yahoo.com.br; ^2^Department of Occupational Therapy, Federal University of Minas Gerais, Belo Horizonte, Brazil.

**Aim:** To analyze and synthesize research studies regarding the use of the Movement ABC (MABC), first and second editions, to assess the motor performance of Brazilian children.

**Method:** Literature review based on search in electronic databases – MedLine, Lilacs (Brazilian) and Scielo (South America) – according to the following criteria: research articles published in Portuguese, English or Spanish, observational and experimental studies reporting data on the use of the MABC^1^ or MABC-2^2^ with Brazilian children.

**Results:** From the 360 articles located in the searches, following exclusions, 23 were selected for the review, most of them were published in the last four years (N=18), including two studies on the validity of the MABC-2 for the Brazilian children. The MABC and MABC-2 were used by different professionals, especially physical educators and occupational therapists. The studies report data on 5.613 children for the motor test, considering both versions, and 565 for the checklist. The studies included children from 3 to 13 years old, but most publications aimed to children 7 to 10 years old and their results indicate the MABC and MABC-2 are useful to identify mild motor difficulties among Brazilian children. No study point to the need to adapt the motor test, however, the checklist seems to need some adjustments to be used in Brazil.

**Discussion:** The growing number of studies published in the last four years might be associated to increased awareness about Developmental Coordination Disorder (DCD) and its consequences on the children´s development and daily life. The fact that most studies were focused on MABC-2 age band two is consistent with the age in which the school tasks become more demanding making the repercussions of the motor impairment more evident. The motor test´s utility, confirmed in studies, encourages investment in its validation for the Brazilian children. Although there are two recent studies on the validity of the motor test and the checklist for children from the southern of Brazil, with results suggesting that while the MABC-2 Checklist may need minor modification, the motor test and its original normative data could be used with these children, further validity studies are needed, as Brazil is a continental country.

**References**:

1. Henderson, S. E., & Sugden, D. A. (1992). *Movement assessment battery for children manual*. London: The Psychological Corporation Ltd.

2. Henderson, S. E., Sugden, D. A., & Barnet, A. (2007). *Movement assessment battery for children 2nd ed (MABC-2)*. London: Harcourt Assessment.

**Keywords:** MABC; MABC-2; Assessment; Motor coordination; Children; Brazil.

### Bibliographic analysis of Brazilian literature about Developmental Coordination Disorder

T.B. Pontes^**1**^, C.R.S. Araújo^**2**^, A.L. de Oliveira Monteiro^**1**^, P.H.T.Q. de Almeida^**1**^ & L.M. Vendrusculo-Fangel^**1**^

^1^Faculdade de Ceilândia, Universidade de Brasília, 72220-900 Brasília, Brazil. tatianapontes@unb.br; ^2^Departamento de Terapia Ocupacional, Universidade Federal da Paraíba, João Pessoa, Brazil.

**Aim:** To analyze the quantity and quality of studies on Development Coordination Disorders published by Brazilians researchers

**Method:** The bibliographical study comprised two steps: in the first part an extensive literature research was performed to obtain all the publications about the topic. In the second stage, a systematic review of articles that addressed the interventions with children diagnosed with DCD was performed. Research was performed in SciELO, Lilacs and Medline electronic databases, without a publishing date restriction. Keywords “Disorders”, “Development”, “Coordination” and “DCD” were used both in English and Portuguese, in order to characterize Brazilian studies. A critical review was conducted using the Occupational Therapy Systematic Evaluation of Evidence

**Results:** 23 articles were found. Results showed few studies in the area in Brazil and publications with low to median quality.

**Discussion:** The reduced amount of studies on DCD indicates the little number of researches conducted in the area at the country suggests the relevance of scientific investigation of the disorder, since this condition can interfere not only in the lives of children, but also persist through adolescence and adulthood, generating a significant impact on social and academic environments.

**References**:

1. Sherrington, C., Herbert, R. D., Maher, C. G., & Moseley, A. M. (2000). PEDro. A database of randomized trials and systematic reviews in physiotherapy. *Manual Therapy, 5*(4), 223-226.

2. Zwicker, J. G., Missiuna, C., Harris, S. R., & Boyd, L. A. (2012). Developmental coordination disorder: A review and update. *European Journal of Paediatric Neurology, 16*(6), 573-581.

**Keywords:** Development Coordination Disorder; Systematic Review; Scientific Production.

### The relationship between hot and cold executive function in Developmental Coordination Disorder

S. Rahimi-Golkhandan^**1**^, B. Steenbergen^**1,2**^, J.P. Piek^**3**^, K. Caeyenberghs^**1**^ & P.H. Wilson^**1**^

^1^Australian Catholic University, Melbourne, Australia. shahin.rahimi@acu.edu.au; ^2^Behavioural Science Institute, Radboud University Nijmegen, Netherlands; ^3^Curtin University, Bentley, Australia

**Aim:** Developmental coordination disorder (DCD) has been linked to deficits of cold and hot executive function (EF). Requiring clarification is the critical relationship between cold and hot EF in this cohort. At the level of individual differences, our aim was to investigate whether children with DCD who show reduced hot EF also show deficits of cold EF.

**Study 1 - Method:** 36 children (14 DCD) aged 6 to 12 years completed two cold EF tasks (i.e., One-Back Task (OBT), and Groton Maze Learning Test (GMLT)) and one hot EF task based on the Iowa Gambling Task, the Hungry Donkey Task (HDT).

**Results:** First, we identified those children who scored either within or outside the 95% confidence interval (CI) of the controls on each task. Six (43%) of the DCD group performed worse than controls on both the hot EF task and at least one cold EF task. Another seven (50%) showed deficits in one domain, only, and only one child scored within the 95% CI of the controls on all the three tasks. There was no significant correlation between the HDT and the cold tasks.

**Study 2 - Method:** 36 children (12 DCD) were examined on a facial go/no-go task tapping hot EF; here neutral facial expressions were paired with either happy or sad faces. Facial expressions were used as both go and no-go targets in different blocks. The cold EF tasks were the same ones used in study 1.

**Results:** Four (33%) of the DCD group showed reduced hot EF as well as poor performance on at least one of the cold tasks, five (42%) had scores outside the 95% CI of the controls on either the hot task or one of the cold ones, and three performed as well as controls on all three tasks. Commission errors on the go/no-go task were correlated positively with errors on the GMLT, and negatively with accuracy of the OBT.

**Discussion:** Critically, our findings support the view that DCD is a heterogeneous disorder at the level of cognition; a proportion of children show no deficits on either hot or cold EF tasks, and a smaller number perform at age appropriate levels on all EF tasks. Developmental data show moderate correlations between different EF abilities, perhaps a reflection of common (underlying) processing abilities like inhibitory control. However, our findings do accord with recent neuroimaging studies that point to some dissociation between abilities under conditions of varying emotional investment. This has important implications for the remediation of motor and EF deficits in this cohort, and underlies the need to tailor EF training to the individual needs of each child with DCD.

**Keywords:** Executive function; Cognitive control; Working memory; Delay of gratification; Developmental Coordination Disorder.

### Comorbid Developmental Coordination Disorder in semi-hospitalized children with psychiatric disorders

M. Ramos Mari Dreyer^**1**^, M.A. Boarati^**2**^, T. Pantano^**2**^, S. Scivoletto^**2**^, C. Castanho de Almeida Rocca^**1**^

^1^Psychology and Neuropsychology Units, Department and Institute of Psychiatry, University of São Paulo Medical School, rua Dr. Ovidio Pires de Campos, 785- 4 floor, 05403-903, São Paulo, Brazil. mrmdreyer@gmail.com; ^2^Department and Institute of Psychiatry, University of São Paulo Medical School, Brazil.

**Aim:** Developmental Coordination Disorder (DCD), affects between 5 and 6% of children between 5 and 11 years old, (APA, 2013) and recent studies show its impact through adulthood. The object of this study was to investigate the presence of psychomotor dysfunctions in a sample of children and adolescents presenting psychiatric disorders.

**Method:** A sample of 58 children between 8 and 17 years old undergoing the rehabilitation program of the Day Hospital of Institute and Department of Psychiatry, of the University of São Paulo School of Medicine (IPq-HC-FMUSP) was investigated. DSM-5 criteria was used for diagnostic purposes and the Movement Assessment Battery for Children-2nd. Edition (M ABC-2) was used for the motor competence assessment. Total impairment score > 16 percentile on the M ABC 2 was the cut-off set to support the diagnosis of DCD. Other tools as the standard anamnesis interview from the Unit of Child and Adolescent Psychiatry, from IPq-HCFMUSP (Andrade & Boarati, 2011), the Kiddie Schedule for Affective Disorders and Schizophrenia for School-Age Children-Present and Lifetime (K-SADS-PL) and the Child Behaviour Checklist (CBCL), both validated for Brazilian population (Brasil, 2003; Duarte & Bordin, 2000) were also administered. Parents signed written informed consent from the Psychology and Neuropsychology units from HC-FMUSP prior to testing. A descriptive method was used for the data analysis and presentation of the results.

**Results:** DCD was found in children with the following diagnosis: 72% of Attention deficit hyperactivity disorder (ADHD), (8 out of 11 cases), in 65% of early onset bipolar disorder and in severe depressive episodes (11 out of 17 cases), 67% of psychosis (2 out of 3 cases), 62% of anxiety disorder (5 out of 8 cases), 100% of autism spectrum disorder (ASD), 6 out of 6 cases, 100% of somatoform disorder and dissociative conversion disorder (2 out of 2 cases) and in conduct disorder (1 case). DCD was not observed in any of individuals with emotionally instable personality disorder symptomatology, presenting disruptive behaviour and exhibit symptoms suggesting the development of a personality disorder (5 cases).

**Discussion:** The results showed a high prevalence of developmental coordination disorder in a group of children along with disruptive behaviour and psychiatric disorders. An already solid literature describes that the presence of psychomotor dysfunctions are expect in neurodevelopmental diagnosis such as ADHD (50%), learning disorders and ASD. Few studies are dedicated to investigate the incidence of the comorbid psychomotor disorder in children and adolescents presenting mood, anxiety or psychotic symptomatology. Thus further research should be conducted to investigate the correlation between psychomotor disorders and those conditions. Such evidence also justifies the importance of psychomotor assessment followed by intervention as standard practice for the treatment of early onset psychiatric disorders.

**References**:

Dewey, D., Kaplan, B. J., Crawford, S. G., & Wilson, B. N. (2002). Developmental coordination disorder: Associated problems in attention, learning, and psychosocial adjustment. *Human Movement Science, 21*, 905–918.

Ekorna?s, B., Lundervold, A. J., Tjus, T., & Heimann, M. (2010). Anxiety disorders in 8-11-year-old children: motor skill performance and self-perception of competence. *Scandinavian Journal of Psychology, 51*(3), 271-277.

Emck, C., Bosscher, R. J., Van Wieringen, P. C., Doreleijers, T., & Beek, P. J. (2011). Gross motor performance and physical fitness in children with psychiatric disorders. *Developmental Medicine and Child Neurology, 53*(2), 150-155.

Hill, E.L. & Brown, D. (2013). Mood impairments in adults previously diagnosed with Developmental Coordination Disorder. *Journal of Mental Health, 22*(4), 334-340.

Kirby, A., Sugden, D., & Purcell, C. (2014). Diagnosing developmental coordination disorders. *Archives of Disease in Childhood, 99*(3), 292-296.

**Keywords:** Psychomotor disorder; Motor abilities; Developmental coordination disorder; Paediatric psychiatric disorder.

### Inter-language reliability of the European-French Developmental Coordination Disorder Questionnaire’07 (DCDQ’07)

S. Ray-Kaeser^**1**^, E. Thommen^**2**^, R. Martini^**3**^ & A.M. Bertrand^**4**^

^1^Department of Occupational Therapy, School of Social Work & Health Sciences, EESP, University of Applied Sciences and Arts Western Switzerland, 1110 Lausanne, Switzerland. sylvie.ray@eesp.ch; ^2^Department of Social Work, School of Social Work & Health Sciences, Lausanne, Switzerland; ^3^Department of Occupational Therapy Program, Faculty of Health Sciences, University of Ottawa, Canada; ^4^Department of Occupational Therapy, School of Social Work & Health Sciences, Lausanne, Switzerland.

**Aim:** Developmental Coordination Disorder (DCD) is a condition whereby coordination difficulties significantly interfere with the performance and participation in daily activities. Guidelines for identifying DCD recommend the use of a culturally adapted, reliable and valid parent questionnaire, such as the DCDQ’07, to measure the child’s performance in daily activities (Blank *et al*., 2011). The DCDQ’07 is a Canadian-English parent-report questionnaire that assesses children’s motor performance with respect to the three factors that consider impairment in DCD: “general coordination”, “fine motor/handwriting”, and “control during movement”. The European-French DCDQ (DCDQ-EF) was produced following Beaton *et al.* (2000) guidelines for cross-cultural adaptation (Ray-Kaeser *et al.*, 2015). While modifications followed these standard procedures, the reliability of the DCDQ-EF should not be assumed without assessment of its psychometric properties. The aims of this study were to: (1) assess the inter-language reliability between the DCDQ-EF and the DCDQ’07 (2) assess the internal consistency of the DCDQ-EF and (3) estimate its predictive validity.

**Method:** The participants (n=30) were all living in the French-speaking part of Western Switzerland. They were French and English-speaking parents of children (non-clinically-referred: n=22; clinically-referred: n=8), ranging in age from 5 to 14 years (Mean age (SD)= 8.8 (2.8)). Parents completed both the DCDQ’07 and the DCDQ-EF (random order) at approximately 1-month interval.

**Results:** The inter-language reliability of the DCDQ-EF was moderate to excellent for each item and the two factors “general coordination” and “control during movement” (ICC=0.63-0.92). However, there was a systematic difference between the mean of the original questionnaire and that of the translated version for the factor “fine motor/handwriting” (diff.=0.80, p=0.03) and for the total (diff.=2.3, p=0.03). Cronbach’s Alpha coefficient for the 15-item was high (0.93). All item-total correlations were moderate to high (0.46-0.88). Using the cut-off scores established by Wilson *et al.* (2009) for the DCDQ’07, the overall DCDQ-EF sensitivity was 100% and specificity was 95% with this sample.

**Discussion:** The DCDQ-EF showed fairly similar internal consistency to the original DCDQ’07 and Canadian-French DCDQ, attesting to the homogeneity of all items. Furthermore, no item seemed to be problematic. However, given the systematic difference between the total score of the DCDQ-EF and that of the DCDQ’07, the appropriateness of the original cut-off scores may need further examination for use in French-speaking Switzerland.

**References**:

Beaton, D. E., Bombardier, C., Guillemin, F., & Ferraz, M. B. (2000). Guidelines for the Process of Cross-Cultural Adaptation of Self-Report Measures. *Spine 25*(24), 3186-3191.

Blank, R., Smits?Engelsman, B., Polatajko, H., & Wilson, P. (2011). European Academy for Childhood Disability (EACD): Recommendations on the definition, diagnosis and intervention of developmental coordination disorder (long version). *Developmental Medicine & Child Neurology*, *54*(1), 54-93.

Ray-Kaeser, S., Satink, T., Andresen, M., Martini, R., Thommen, E. & Bertrand Leiser, M. (accepted). European-French Cross-cultural Adaptation of the Developmental Coordination Disorder Questionnaire and Pretest in French-speaking Switzerland. *Physical & Occupational Therapy in Pediatrics.*

Wilson, B. N., Crawford, S. G., Green, D., Roberts, G., Aylott, A., & Kaplan, B. J. (2009). Psychometric properties of the revised developmental coordination disorder questionnaire. *Physical & Occupational Therapy in Pediatrics*, *29*(2), 182-202.

**Keywords:** DCDQ’07; Psychometric assessment; Reliability.

### Imitation of complex gestures in children with Developmental Coordination Disorder

J. Reynolds^**1**^, M. Licari^**1**^, B. Lay^**1**^, J. Williams^**2**^ & C. Elliott^**3**^

^1^School of Sport Science, Exercise and Health, The University of Western Australia, Perth, 6009, Australia. jessica.reynolds@research.uwa.edu.au; ^2^College of Sport and Exercise Science, Victoria University, Melbourne, 3011, Australia; ^3^Faculty of Health Sciences, Curtin University, Perth, 6102, Australia.

**Aim:** It has been hypothesised that children with DCD have deficits in the functioning of the mirror neuron system (MNS). Deficits in MNS function may impair imitation, which is our primary method of learning motor skills. Previous research provides support for difficulties while imitating meaningful learned gestures, however, only a small number of studies have explored imitation of unknown gestures in children with DCD. The present study examined the imitation ability of children with and without DCD using unlearned complex gestures.

**Method:** 53 boys aged 6-13 years participated in this study, 29 with DCD (mean age = 9.29yrs ± 1.85) and 24 typically developing controls (mean age = 9.49yrs ± 1.78). Children were tested on the MABC-2, with children in the DCD group ≤16th percentile (mean percentile = 5.69 ± 5.28, range = 0.5-16) and typically developing controls ≥20th percentile (mean percentile = 68.13 ± 20.32, range = 25-98). Measures of imitation were collected using the standardized Postural Praxis and Sequencing Praxis components of the Sensory Integration and Praxis Tests (Ayres, 1989). Assessments were scored by two assessors (one blinded to group).

**Results:** Consistent with previous research, children with DCD displayed imitation deficits. Children with DCD had significantly lower scores than typically developing controls on the postural praxis (DCD median = 21, control median = 27.5, U = 69.5, p < 0.001). Similarly, children with DCD scored lower on the sequencing praxis (DCD median = 68, control median = 96.5, U = 118.0, p < 0.001), and in particular displayed difficulty with finger sequencing gestures (DCD median = 17, control median = 34, U = 170.50, p < 0.001).

**Discussion:** Initial evidence supports imitation deficits in children with DCD. This provides behavioural level support for a deficit in MNS functioning. Given that visual learning, and learning by imitation is our primary modality of learning skills through modelling behaviour and actions, deficits in praxis have the potential to contribute to the motor deficits associated with DCD. A greater understanding of imitation performance in children with DCD has the potential to inform intervention programs for this population.

**References**:

Ayres, A. J., (1989). *Sensory integration and praxis tests: SIPT manual*. Los Angeles: Western Psychological Services.

**Keywords:** Imitation; Gestures; Sensory Integration and Praxis Tests; Mirror neuron system (MNS).

### Can a Little instrument make a big noise? A cross-cultural collaboration for identifying motor delay in young preschoolers

**T. Rihtman^1^, B.N. Wilson^2^, S. Cermak^3^, S. Rodger^4^, A. Kennedy-Behr^5^, L. Snowdon^5^, M.M. Schoemaker^6^, M. Cantell^6^, S. Houwen^6^, M. Jover^7^, J-M. Albaret^8^, S. Ray-Kaeser^9^, L. Magalhães^10^, A.A. Cardoso^10^, H. Van Waelvelde^11^, D. Hultsch^12^, S. Vincon^13^, M.H. Tseng^14^, A. Pienaar^15^, D. Coetzee^15^, A. Nakai^16^, R. Martini^17^, J. Tercon^18^, D. Green^19^, E. Imperatore (Blanche)^20^, J. Diaz^3^ & S. Parush^21^**

^1^Hebrew University, Jerusalem, Israel & Coventry University, United Kingdom. tanya@tanya-branko.com; ^2^Alberta Children’s Hospital and the University of Calgary, Calgary, Canada; ^3^University of Southern California, United States of America; ^4^University of Queensland, Australia; ^5^University of the Sunshine Coast, Australia; ^6^University of Groningen, The Netherlands; ^7^Aix-Marseille Université, Aix en Provence, France; ^8^University of Toulouse III Paul Sabatier, France; ^9^Haute école de travail social et de la santé, Lausanne, Switzerland; ^10^Universidade Federal de Minas Gerais, Brazil; ^11^Artevelde University College and Ghent University, Belgium; ^12^University of Gie?en, Germany; ^13^Child Centre Maulbronn, Germany; ^14^National Taiwan University, Republic of China; ^15^North West University, Potchefstroom, South Africa; ^16^Hyogo Children’s Sleep and Development Medical Research Center, Kobe, Japan; ^17^University of Ottawa, Canada; ^18^Community Health Centre Ljubljana & University of Ljubljana, Slovenia; ^19^Oxford Brookes University, United Kingdom; ^20^University of Southern California, United States of America and Universidad de Chile, Chile; ^21^Hebrew University, Jerusalem, Israel.

**Aim:** Even though Developmental Coordination Disorder (DCD) is typically not diagnosed before 5 years, identification and monitoring of younger preschool children at risk of being diagnosed with DCD may mitigate secondary complications and participation difficulties, through the provision of early support. Screening tools to identify motor difficulties are needed, but instruments developed in one country may not be psychometrically sound when shared between cultures. This study aimed to collaboratively develop the Little Developmental Coordination Disorder Questionnaire (LDCDQ) (a screening instrument to identify motor difficulties in young preschoolers) between several countries, while ensuring numerous psychometrically sound, comparable versions of the tool. This innovative project in the field of DCD will enable the analysis and comparison of different patterns of motor development and/or delay in different cultures.

**Method:** Based on a similar screening instrument for older children, the Little DCDQ was developed in Hebrew and psychometrically tested. After generating an English Little DCDQ (following recommended guidelines), 27 researchers from 17 sites adapted and psychometrically tested the instrument with their local cultures and languages. Thereafter, each collaborator used their local Little DCDQ to assess 40 children aged 3-4.11 (20 typically developing and 20 with suspected motor difficulties) following the same protocol, and the data was compared to assess motor development across cultures.

**Results:** The process of the first phase of this collaboration will be briefly described and initial cross-cultural comparative results will be reported based on data collected to date. Within most countries, significant differences in motor performance between referred and non-referred children were found; sub-scores in which differences have not been identified may be due to specific cultural characteristics. When comparing between countries, significant differences were more noticeable for non-referred than referred children; trends in high- and low-scoring means will be discussed.

**Discussion:** This study has important implications for DCD research and practice. This is the first attempt to develop an instrument with the aim of facilitating cross-cultural comparison of DCD in young preschoolers, which will enable a unified language for researchers investigating typical motor development and motor delay in this population.

**Keywords:** Developmental Coordination Disorder; Motor development; Young Preschoolers; Cross-cultural assessment.

### Lessons learned using an eye-tracker in school-aged children with Developmental Coordination Disorder performing a real-world visual-motor task

L.M. Rivard^**1**^, L.R. Wishart^**2**^, T.D. Lee^**3**^ & C. Missiuna^**4**^

^1^School of Rehabilitation Science and CanChild Centre for Childhood Disability Research, McMaster University, 1400 Main St. W., Hamilton, Ontario L8S 1C7, Canada. lrivard@mcmaster.ca; ^2^School of Rehabilitation Science, McMaster University, Canada; ^3^Department of Kinesiology, McMaster University, Canada; ^4^School of Rehabilitation Science and CanChild Centre for Childhood Disability Research, McMaster University, Canada.

**Aim:** Eye tracking has become an innovative technique for measuring cognitive processes in children with neurodevelopmental disorders but this method has been employed infrequently with children with developmental coordination disorder (DCD). Eye tracking use may improve understanding of the cognitive processes that influence motor performance in DCD but it is not yet known whether this is feasible. Comorbid conditions may impact the reliable use of eye trackers with this population. The purpose of this study was to investigate the feasibility of using an eye tracker with children with DCD performing a real-world visual motor task.

**Method:** As part of a larger study investigating selective visual attention, 12 children with DCD (11 M, 1F; mean age (range): 10.5 yrs (6.7)) wore a mobile eye tracker while pouring water from 3 glass ‘pouring’ cups into 3 colour-matched plastic ‘filling’ cups sequentially (single trial). Children performed 12 trials of increasing difficulty (position of filling cups was altered on every 3rd trial). Each trial lasted 30-60 seconds; total testing time was 15-30 minutes. Parents completed the DCDQ’07 and the 45-item Conners-3 Parent Rating Scales of attention. Children’s coordination was assessed using the Movement Assessment Battery for Children-2 (MABC-2). Following task completion, gaze overlay videos were extracted and analysed.

**Results:** Eye tracking was feasible and reliable for most children with DCD (n=9); 3 children had poor gaze fixation for calibration affecting the reliability of their data. These children had visual impairments and significantly lower MABC-2 percentiles (M=0.67 (0.29)) than the group without calibration difficulties (M=3.33 (2.86); t(10)=2.75, p <.05). Attention difficulties were formally diagnosed and/or reported by parents in 9 children, including 2 of the 3 children with poor calibration; however, no differences were found between groups with and without poor calibration based on Conners-3 data. Difficulties maintaining posture, sensory challenges, fit of the eye tracking glasses, and corrective eyewear were additional factors hindering data collection.

**Discussion:** Eye tracking appears to be a feasible approach to use with most children with DCD during a real-world visual motor task. However, the nature and heterogeneity of DCD, as well as practical challenges, may limit this approach. Recommendations for overcoming challenges in eye tracking research with children with DCD will be provided.

**Keywords:** Eye tracking; Feasibility; Comorbidity; Attention; Visual difficulties.

### Relationship between foot posture and balance in young children at risk for Developmental Coordination Disorder: Preliminary results

C. Rodriguez^**1**^, C. Missiuna^**2**^, B. Timmons^**3**^ & J. Cairney^**1**^

^1^Department of Family Medicine, McMaster University, Hamilton, L8P 0A1, Canada; odrigmc@mcmaster.ca; ^2^School of Rehabilitation Science, McMaster University, Hamilton, Canada; ^3^Department of Pediatrics, McMaster University, Hamilton, Canada.

**Aim:** Some authors have shown that foot posture, specifically foot pronation (or flat feet), is associated with poor balance in young adults and older adults. Children with DCD are known to have impaired motor coordination, including impaired balance, and have also been shown to have flat feet^1^, yet foot posture is rarely assessed in children with DCD. We wish to examine the relationship between foot posture and balance in a group of children at risk for DCD (aDCD) and those who are typically developing (TD) to determine whether foot posture contributes to the impaired balance commonly observed in children with DCD.

**Method:** Young children aged 4 and 5 years who are enrolled in a longitudinal cohort study (Coordination and Activity Tracking in CHildren [CATCH]) comprise the study sample. Balance as well as fine and motor gross motor skills were assessed using the *Movement Assessment Battery for Children version 2* (MABC2). Foot posture was assessed using the *Foot Posture Index* (FPI) which is a clinical tool comprised of 6 items assessing the rear and forefoot. Children scoring at or below the 16th percentile (overall) on the MABC2 are considered to be aDCD. Two raters independently completed the FPI; the average total score for each the left and right foot was used. Children with both MABC2 and FPI total scores from two raters were included in the analysis (n=71).

**Results:** FPI total score was transformed to logit values (Keenan et al 2007). FPI logit total score was not different between the aDCD (n=46) and TD (n=25) groups (F(1,69)=0.003, p=0.96) nor between the right and left foot (F(1,69)=3.41, p=0.07). We also examined the relationship using 1.0 SD cutpoints for pronation: published cutpoint for minors (Redmond et al 2008) and a cutpoint derived from our data. No children in our sample fell below the published 1.0 SD cutpoint. Using the derived 1.0 SD cutpoint, the proportion of children with potentially abnormal pronation did not differ between the aDCD or TD groups (left foot: Fisher’s Exact test p>0.05, right foot Chi-Square=1.24, df=1, p=0.26).

**Discussion:** The results suggest that foot posture does not contribute to the impaired balance observed in young children at risk for DCD; however, given the young age of the children in the present study, it is possible that the relationship between foot posture and balance may emerge later when other differences become apparent between these two groups of children (eg, BMI, etc).

**References**:

1. Kirby, A., & Davies, R. (2006). Developmental Coordination Disorder and Joint Hypermobility Syndrome – overlapping disorders? Implications for research and clinical practice. *Child: Care, Health and Development, 325*, 513-519.

**Keywords:** Foot posture; Balance; Developmental coordination; Etiology.

### The unique characteristics of students with learning disabilities and Developmental Coordination Disorders

S. Rosenblum & K. Sharfi

The Laboratory of Complex Human Activity and Participation (CHAP), Department of Occupational Therapy, University of Haifa, Israel. rosens@research.haifa.ac.il

**Aim:** The literature has indicated a high percentage of comorbidity between learning disabilities and Developmental Coordination Disorders (DCD). However, works describing personal, health and executive control characteristics, as related to daily function and quality of life among adults who are diagnosed with both LD and DCD are scarce^1,2^. The study aim was to find unique personal, health, and executive control characteristics and their relationships with daily function and life quality characteristics among adults diagnosed with LD and DCD, compared to those of controls.

**Methods:** The participants were 60 adults aged 21 to 50. Thirty adults diagnosed with LD and DCD and 30 controls matched for age, gender, socio-economic-status and years of education, volunteered for the study. Students with LD were diagnosed by a professional expert as having DCD based on the Adult Developmental Coordination Disorders checklist (ADC)^3^. Participants completed the following questionnaires: (a) demographic and health questionnaire (b) Body function - Behavioral Rating Inventory of Executive Functions (BRIEF-A) (c) Activity and Participation - Time Organization and Participation (TOPS) and) d( Life quality - World Health Organization Quality Of Life questionnaire (WHOQOL-BREF).

**Results:** Adults with LD and DCD significantly differed from controls in various *personal factors*; for example, significantly more participants were single and lived with their parents attended more than one high school and graduated with bad feelings. They reported a significantly deficient mental and emotional health condition, including a higher percentage of current use of medication and health care services, compared to controls. Their executive control was significantly different from that of controls as was their organization in time abilities and life quality. Regression analysis indicated that 54% of the variance of their psychological quality of life was predicted by specific EF domains (BRIEF A) and by their organization in time ability.

**Discussion:** Adults with LD and DCD have unique characteristics concerning personal factors, health, executive control and activity performance. This may partially explain the difficulties they experience in daily function and influence their life quality. The implications of these results to future theoretical and clinical developments for this population will be discussed.

**References**:

1. Gerber, P. J. (2012). The Impact of learning disabilities on adulthood. A review of the evidenced-based literature for research and practice in adult education. *Journal of Learning Disabilities*, *45*(1), 31-46.

2. Gilger, J. W., & Kaplan, B. J. (2001). Atypical brain development: a conceptual framework for understanding developmental learning disabilities. *Developmental Neuropsychology*, *20*(2), 465-481.

3. Kirby, A., Edwards, L., Sugden, D., & Rosenblum, S. (2010). The development and standardization of the adult Developmental Co-ordination Disorders/dyspraxia checklist (ADC). *Research in Developmental ­Disabilities*, *31*(1), 131-139.

**Keywords:** Personal factors; Health condition; Executive control.

### Kinematic assessment of the catching movement of a child with motor difficulties: A single case study

Y. Sawae^**1**^, Y. Murakami^**1**^ & A. Sugiyama^**2**^

^1^Faculty of Health and Sport Sciences, University of Tsukuba. JAPAN. sawae.yukinori.ka@u.tsukuba.ac.jp; ^2^Graduate School of Comprehensive Human Sciences, University of Tsukuba. JAPAN

**Aim:** It is known that when catching a ball in one hand, the level of motor skill required as school-aged children, some children with motor difficulties place the non-catching arm at the side of the body. This study hypothesized that such catching movements are due to limited motor experiences caused by repeated their failure events and the children’s attempts to avoid failure. As a first step toward testing this hypothesis, a single case study was carried out in which an individualized intervention sought to modify catching movement.

**Methods:** The participant of this study was a male child with motor difficulties (red zone of M-ABC 2nd)^1^ who began an instruction program within the MDCG (Movement Development Clinic Group) of the authors’ university. At the beginning of the program, he was 11 years old and a general elementary school student in Japan. Throughout the study, the participant received individualized instruction in ball skills (for one year) based on a 12-session task-oriented approach which, for example, participant was requested to throw balls against the target board and tilt it back third round of throwing, and during each session, his one-hand catching movement was video recorded and subjected to kinematic analysis of the knee joint, elbow joint, and waist angles.

**Results:** The participant’s post-catching movement showed characteristic variation as follows: 1) The non-catching arm, which braced the upper limb at the side of the body to limit motion during the early part of the intervention, played a more functional role in his catching movement during the latter stages of the study. 2) The range of waist motion during his post-catching movement increased in comparison with his pre-catching movement (i.e., the pre-catching angle of his waist was M = 169.3, SD = 10.8; the post-catching angle was M = 145.5, SD = 20.9).

**Discussio**n**:** In the early stages, the participant was unable to perform the catching movement without limitations in his waist and joint motion (Newell & Vaillancort, 2001). After the intervention, however, he could perform the movement without these limitations. The findings suggest that it might be possible to develop better catching movement among children with motor difficulties through an intervention based on proactive repetitive motion.

**Reference**:

1. Henderson S.E., Sugden, D. A., & Barnett, A. (2007). *Movement Assessment Battery for Children*. London: Pearson.

**Keywords:** Kinematic assessment; Catching movement; Motor difficulties; Case study.

### Psychometric item analysis of the Assessment of Motor Coordination and Dexterity (ACOORDEM) for 4 years old Brazilian children

C.G. Silva^**1**^, A.M.V.N. Van Petten^**2**^, E. Harsányi^**2**^, A.A. Cardoso^**2**^, M.B. Rezende^**2**^ & L.C. Magalhães^**2**^

^1^Departamento de Terapia Ocupacional, Universidade Federal de Pelotas, Av. Duque de Caxias, 250, Pelotas, RS, 96030-001, Brazil. cynthiagirundi@gmail.com; ^2^Departmento de Terapia Ocupacional, Universidade Federal de Minas Gerais, Av. Antônio Carlos 6627, Belo Horizonte, MG, 31270-901, Brazil.

**Aim:** There are several assessment tools to identify children with Developmental Coordination Disorder (DCD). However, they were created abroad, are costly and do have normative data for the Brazilian children. The Assessment of Motor Coordination and Dexterity (ACOORDEM) was create to assess aspects of motor development and functional performance relevant for school performance and social participation in 4-8 years old Brazilian children. Continuing ACOORDEM´s validation process, the aim of this study was to investigate the validity and reliability of the test for 4 years old children.

**Method:** Eighty children 4 years old, divided into equal groups by gender and type of school (public or private) were evaluated. Children were assessed at school with ACOORDEM and the MABC-2. Children´s parents responded to ACOORDEM parents´ questionnaire and the DCDQ-Little. Retest was completed with 10 participants.

**Results:** In the MABC-2 and DCDQ-Little there were no significant differences by gender or type of school. Concerning ACOORDEM, only three (5.8%) of the 51 items presented statistically significant gender difference, with girls performing better in all of them. Considering the type of school, statistically significant difference was observed in seven items (13.7%) with private school children performing better than public school. Regarding test-retest reliability, 10 (30.3%) out of 33 quantitative items presented statistically significant differences on test and retest; the remaining items presented similar means. For the qualitative items, ICC identified 17 (94.4%) out of 18 items with moderate to excellent reliability.

**Discussion:** Few quantitative and qualitative items presented statistically significant differences associated with gender and type of school. Concerning gender, differences in performance were not expected since younger children are acquiring skills and may not show differentiation in motor behavior. Regarding type of school, better performance of private school children in some items seems to be influenced by environmental and socio cultural issues. Test-retest reliability showed good indices for quantitative and qualitative items. In general the results are consistent with previous studies with children 6-8 years old, showing that the test was well accepted by the children, has acceptable psychometric qualities, however, some items need adjustment or to be eliminated for 4 years old children, to reduce the length of the test.

**References**:

Henderson, S. E., Sugden, D. A., & Barnet, A. (2007) *Movement assessment battery for children 2nd ed (MABC-2)*. San Antonio, TX: The Psychological Corporation.

Magalhães, L. C., Rezende, M. B., Cardoso, A. A. (2014). *Avaliação da Coordenação e Destreza Motora – ACOORDEM - Versão 5*. Belo Horizonte: Departamento de Terapia Ocupacional, UFMG. Manuscrito não publicado.

Rihtman, T., Wilson, B. N., & Parush, S. (2011). Development of the Little Developmental Coordination Disorder Questionnaire for Preschoolers. *Research in Developmental Disabilities, 32*(4), 1378-1387.

**Keywords:** Assessment; Reliability; Validity; ACOORDEM; Developmental Coordination Disorder.

### Systematic Detection of Writing Problems (SOS-2): a valid and easy to use instrument

B. Smits-Engelsman^**1**^, I. van Bommel-Rutgers^**2**^ & H. van Waelvelde^**3**^

^1^Department Kinesiology, Movement Control & Neuroplasticity Research Group, University of Leuven, Belgium. Department of Health and Rehabilitation Sciences, University of Cape Town, South Africa; ^2^De Kinderpraktijk Meppel/Ruinerswold and Docent Avans+ University Breda, The Netherlands. vanbommelingrid@gmail.com; ^3^Department Rehabilitation Sciences and Physiotherapy, Ghent University, Belgium.

**Aim:** The SOS-2 has been developed in 2014 to detect handwriting problems and to measure writing speed in children. The purpose was to make an easy and fast instrument (scoring takes max 10 minutes) that can reliably detect poor handwriting. The SOS-2 contains criteria that are described in the literature as critical for poor writing (e.g. increased variation in size, dysfluencies, poor letterform). Results of earlier studies of the SOS already indicated that the psychometric properties were adequate to identify children with handwriting problems. Purpose of this study was the develop norm scores for children between 6 and 10 years of age and to determine face validity of the SOS-2.

**Method:** Data for the Dutch version were gathered from children in elementary schools between 2009 and 2013. Norms were developed for children (grade 1 to 4) who could not yet write with connecting strokes (printed letters) and for cursive writing (n=938). The writing speed norms (n=3586) have been collected for grade 1 to 4. For face validity, the SOS scores were compared to teachers’ and therapists’ classification of anonymous handwriting samples. These data were also used to determine the cut off values for good, doubtful and poor handwriting.

**Results:** Norms are presented in a Dutch and English manual “Systematic Detection of Writing Problems” (SOS-2-NL). SOS scores were significantly different for the classifications of the teachers (good, doubtful, poor). The SOS score for handwriting samples judged as “poor handwriting” was 5.1; for samples judged as “doubtful” the average SOS score is 4.2 and for “good handwriting” the average SOS score was 3.4 (F (2,537) =16.39, p< 0.001). High agreement between the handwriting judgment of the therapists and the SOS indicated good face validity (rs=0.89). Therapists reported the SOS easy to administer and the total time needed for scoring was <10 minutes.

**Discussion:** The SOS-2 seems a valid instrument to detect handwriting problems in an easy way. Once a writing problem has been detected further assessment is necessary into the possible underlying causes of the handwriting problems. In accordance with the Belgian study (Van Waelvelde et al. 2012) we showed that the SOS-2 test has good psychometric properties. Norm values have been developed for Dutch, German and Flemish children who write cursive. The Dutch version has also norms for children who do not (yet) use connecting strokes between letters. The test has recently translated into English and there is also a special version of the SOS for patients with Parkinson’s disease (SOS-PD), which has been developed for research purposes. No standardized scores for English-speaking countries have been collected yet.

**Reference**:

Van Waelvelde, H., Hellinckx, T., Peersman, W., & Smits-Engelsman, B. C. M. (2012). SOS: A screening instrument to identify children with handwriting impairments. *Physical & Occupational Therapy in Pediatrics, 32*(3), 306-319.

**Keywords:** Handwriting; Assessment; Measurement.

### Evaluation of handwriting speed for Quebec francophone students in elementary school (grades 3 to 6)

L. St-Denis^**1**^, S. Corriveau^**1**^, D. Giguère^**1**^, J. Santagata^**1**^, A.A. Trudeau^**1**^, M. Coallier^**2**^, M.-F. Morin^**2**^, M. Couture^**1,3**^ & E. Jasmin^**1,3**^

^1^Faculté de Médecine et des Sciences de la Santé, École de Réadaptation, Université de Sherbrooke, Sherbrooke, J1H 5N4, Québec, Canada. Emmanuelle.Jasmin@USherbrooke.ca; ^2^Chaire de recherche sur l’apprentissage de la lecture et de l’écriture chez le jeune enfant, Département d’études sur l’adaptation scolaire et sociale, Faculté d’éducation, Université de Sherbrooke, Canada; ^3^Centre de recherche clinique Étienne-Lebel, Centre hospitalier universitaire de Sherbrooke, Canada.

**Aim:** Handwriting difficulties are common in children with DCD, regardless of their language or culture. However, there is no complete handwriting assessment tool adapted for Quebec francophone students. The Handwriting Assessment Protocol (HAP) is a tool that evaluates the handwriting of children. The five tasks that it contains are writing from memory, near point copying, far point copying, dictation and composition. The aims of this study were (1) to adapt and validate the tasks from this tool for Quebec francophone students in grades 3 to 6 and (2) to develop reference values of handwriting speed for this population.

**Method:** Many sentences for creating this French version were submitted to one speech-language pathologist, three occupational therapists and eight primary school teachers. They chose the sentences that were most appropriate for each grade. Then, four primary schools in Quebec were visited in order to administer the five tasks (writing from memory, near point copying, far point copying, dictation and composition) of the French version of the Handwriting Assessment Protocol. This representative sample involved 201 students in 12 classrooms (grade 3: 47 students; grade 4: 50 students; grade 5: 46 students; grade 6: 58 students). The students were aged between 8 and 12 years old. The schools were chosen using a convenience sampling. Data were analyzed to determine a mean and standard deviation for each school grade. Effects of age and gender were also examined.

**Results:** Reference values (mean value with their standard deviation, percentile) were obtained for handwriting speed of the near point copying and far point copying tasks for each grade (3 to 6). Handwriting speed increases with school grade, both for near point copying and far point copying. Students were faster in far point copying than near point copying.

**Discussion:** These results will facilitate the assessment of the handwriting of Quebec francophone students in grade 3 to 6 by comparing their handwriting speed with the reference values. This way, the needs of students with handwriting difficulties will be better targeted and treated.

**Reference**:

Pollock, N., Lockhart, J., Blowes, B., Semple, K., Webster, M., Farhat, L. et al. (2009). The McMaster handwriting assessment protocol, from http://www.canchild.ca/en/measures/resources/HandwritingProtocolAugust2009FINAL.pdf (Retrieved January 28^th^ 2013).

**Keywords:** Handwriting Assessment Protocol; French adaptation; Handwriting speed; Quebec francophone students; School-aged children.

### Partnering for Change: An innovative and evidence-based model for managing children with Developmental Coordination Disorder in school settings

D. Stewart^**1,2**^, C. Hecimovich^**3**^, N. Pollock^**1,2**^, W. Campbell^**1,2**^, C. Camden^**4,2**^, S. Bennett^**5,2**^, R. Gaines^**6,2**^, D. McCauley^**2**^, J. Cairney^**7,2**^, D. O’Reilly^**8**^, K. Floyd^**3**^, D. Haughton^**9**^, K. Wlodarczyk^**2**^, L. Dix^**1,2**^, C. DeCola^**2**^ & C. Missiuna^**1,2**^

^1^School of Rehabilitation Science, McMaster University, Hamilton, ON, L8S 1C7, Canada. stewartd@mcmaster.ca; ^2^CanChild Centre for Childhood Disability Research, McMaster University, Hamilton, ON, Canada; ^3^Central West Community Care Access Centre, Brampton, ON, Canada; ^4^Centre de recherche du CHUS & School of Rehabilitation, Université de Sherbrooke, Canada; ^5^Department of Teacher Education, Brock University, St. Catharines, ON, Canada; ^6^Children’s Hospital of Eastern Ontario Research Institute, Ottawa, ON, Canada; ^7^Department of Family Medicine, McMaster University, Hamilton, ON, Canada; ^8^Department of Clinical Epidemiology & Biostatics, McMaster University, Hamilton, ON, Canada; ^9^Hamilton, Niagara, Haldimand Brant Community Care Access Centre, Hamilton, ON, Canada.

**Aim:** Evidence indicates children with DCD are at high risk for secondary physical and mental health issues. Early identification is required to enhance children’s participation in school; however, in Ontario, Canada, 85% of children with DCD are placed on lengthy waitlists for occupational therapy (OT) services. Clearly another approach is needed. This poster presents evidence about an innovative model of school-based OT services for children with DCD.

**Method:** A participatory action research approach has been used over 6 years with key stakeholders from government, health care, service provider organizations, schools and families to develop, demonstrate and evaluate a new OT service delivery model, Partnering for Change (P4C). The development phase focused on identifying the key goals of the model: early identification of DCD; chronic disease management by families; prevention of secondary consequences; and a school-based service accessible to all children, whether or not they have been identified or diagnosed with DCD. Instead of 1:1 remediation, the school is the target of intervention. Capacity-building is emphasized and occurs through collaboration and coaching of educators and families. The current research utilizes mixed methods for evaluation, including standardized measures and qualitative interviews of key stakeholders.

**Results:** In one year, 15 OTs offering service 1 day/week in 40 schools (1207 workdays) provided: 3329 individual strategies for 592 children with significant motor challenges; 2980 sessions to screen small groups of children or trial different types of instruction; 704 activities with whole classes of children; 385 educator in-services. Education about how to manage the children’s issues at home was also provided to families. Waitlists were eliminated in all schools in which P4C service delivery was implemented. Interviews indicated educators had a better understanding of DCD and could identify strategies to use to support children with coordination difficulties in their classroom. Individual outcome data for 392 research participants identified with DCD are still being collected.

**Discussion:** P4C is a model of OT service delivery that may be applicable in many countries as a way to manage the needs of children with DCD. Emerging evidence about this model indicates capacity-building with children, families and educators leads to better understanding and management of the needs of children with DCD within the school context.

**Keywords:** Management; Intervention; Service delivery model; Secondary consequences; Early identification.

### Oculomotor function in children with and without Developmental Coordination Disorder

E. Sumner, H.C. Leonard & E.L. Hill

Department of Psychology, Goldsmiths, University of London, New Cross, London, SE14 6NW, United Kingdom. e.sumner@gold.ac.uk

**Aim:** Vision supports almost every aspect of movement by helping individuals to make accurate judgments about their environment. Children learn to stabilize gaze and to control their eye movements to track moving objects or to move the eyes smoothly and quickly from one object to another. Thus, oculomotor control has a primary role in perception and is key to completing general motor tasks. Yet, surprisingly very little research has assessed oculomotor function in children with Developmental Coordination Disorder (DCD). The aim of this study was to employ a short battery of tests that examine basic oculomotor control in children with and without DCD.

**Method:** Twenty-five children, aged 7-10 years, who met the formal diagnosis for DCD (DSM-5; American Psychiatric Association, 2013) participated in this study. A control group of 25 typically-developing children were matched by age and gender to the DCD group. Eye movements were recorded using Eyelink 1000, with the camera as a desktop mount, as children completed four tasks: a fixation task, horizontal smooth pursuit (e.g. tracking a red circle moving across the screen), and a pro- and anti-saccade task. The distinction between the latter tasks is that children follow the target with their eyes when it moves from the central point (pro-saccade) either left or right; or they must inhibit a reflexive saccade and look in the opposite hemifield (anti-saccade) as quickly as possible when the target moves from the centre of the screen.

**Results:** Preliminary analyses of 15 participants per group revealed significant differences in fixation stability. Children with DCD had poorer fixation stability and more frequent drifts away from the visual target than their peers. They also made a higher number of saccades during smooth pursuit. Children with DCD had similar pro- and anti-saccade latencies to their peers, but they found the anti-saccade task very difficult, completing less than a quarter of the trials on average due to many anti-saccade errors. These errors mean that children with DCD repeatedly looked in the same direction as the moving target, instead of looking in the opposite direction as instructed.

**Discussion:** Oculomotor paradigms gain insight into executive control skills. While children with DCD did not present with difficulties in oculomotor response preparation, in comparison to their peers they demonstrated a less efficient visual system for fixating and tracking a target. Children with DCD also had difficulty with the more cognitively challenging anti-saccade task. Further examination of oculomotor dysfunction may help to identify underlying processes of DCD.

**Reference**:

American Psychiatric Association. (2013). *Diagnostic and statistical manual of mental disorders* (DSM, 5^th^ ed.). Arlington, VA: American Psychiatric Association.

**Keywords:** Developmental Coordination Disorder; Oculomotor control; Saccades; Smooth pursuit.

### Using eye tracking methods to investigate action prediction skills in children with Developmental Coordination Disorder

E. Sumner, H.C. Leonard & E.L. Hill

Department of Psychology, Goldsmiths, University of London, New Cross, London, SE14 6NW, United Kingdom. e.sumner@gold.ac.uk

**Aim:** Research using eye tracking methods has shown that typically developing infants and adults naturally anticipate the goal of an observed action (Ambrosini, Costantini & Sinigaglia, 2011). The direct matching hypothesis postulates that when we watch someone pick up or manipulate an object, we activate our own motor representation for that action. The aim of the current study is two-fold. 1) To examine whether children with Developmental Coordination Disorder (DCD) successfully predict the end goal of an observed action, in comparison to children with age-appropriate motor abilities. 2) To identify the information that children rely on to predict the end goal of an action (i.e. hand grasp or trajectory).

**Method:** Data collection is ongoing at the time of writing. Twenty-five children, aged 7-10 years, that meet the DSM-5 criteria (American Psychiatric Association, 2013) for DCD will participate in the present study, alongside 25 typically developing age-matched controls. Eye movements are recorded, using Eyelink 1000, while children observe an actor reach forward and grasp one of two objects (one large, one small). The actor uses either a whole hand grasp or precision grasp when reaching forward to the object. Congruent and incongruent trials are presented.

**Results:** Group comparisons will be made in relation to accuracy of predictive gaze, and onset and arrival times of eye movements towards the correct target. Performance will be compared across congruent and incongruent trials to determine the role of hand-shape on anticipatory gaze. Correlational analyses will consider the relationship between general motor skill and action prediction performance.

**Discussion:** Findings will be discussed in relation to the direct matching hypothesis and whether children with and without DCD activate the motor system to predict the goal of an observed action. Information about whether children with DCD recruit the motor system in this way will contribute to our theoretical understanding of the underlying processes of DCD.

**References**:

Ambrosini, E., Costantini, M., & Sinigaglia, C. (2011). Grasping with the eyes. *Journal of Neurophysiology, 106*, 1437-1442.

American Psychiatric Association. (2013). *Diagnostic and statistical manual of mental disorders* (DSM, 5^th^ ed.). Arlington, VA: American Psychiatric Association.

**Keywords:** Action prediction; Developmental Coordination Disorder; Eye-tracking.

### A preliminary study of the Movement Assessment Battery for Children-Second Edition on Japanese children: Age band 1

K. Suzuki^**1**^, S. Hirata^**2**^, Y. Kita^**1**^, K. Sakihara^**3**^, M. Inagaki^**1**^ & A. Nakai^**4**^

^1^Department of Developmental Disorders, National Institute of Mental Health, National Center of Neurology and Psychiatry, 4-1-1 Ogawahigashi, Kodaira, Tokyo 187-8553, Japan. kt.susuki@ncnp.go.jp; ^2^Department of Special Needs Education, Chiba University, Japan; ^3^Department of Clinical Laboratory Science, Faculty of Medical Technology, Teikyo University, Japan; ^4^Hyogo Children’s Sleep and Development Medical Research Center, Kobe, Japan.

**Aim:** Movement Assessment Battery for Children-Second Edition (MABC2) has been used in various counties to diagnose developmental coordination disorders (DCD). However, since MABC2 has not been standardized in the Japanese population, we were unable to conduct norm referenced assessments of motor skills in Japanese children. The purpose of the present study is to examine whether the original norms for Age Band 1 of the MABC2 are also applicable to Japanese children, in order to prepare for its standardization in Japan.

**Method:** Eighty-one typically developing Japanese children (age range = 4-6 yrs, boys = 37, left-handed = 9) participated in this study. They completed eight examination items for Age Band 1 of the MABC2. Their raw scores were transformed into scaled scores (SSs) based on the original norms (i.e., UK data), and we looked into sex differences in SSs of each examination items, three component scores, and a total score.

**Results:** Mean SSs of three components scores and a total score ranged from 9 to 11. However, when taking a look at each examination items separately, mean SSs of MD3 and Bal2 were below 9 and that of Bal1 was above 11. In addition, boys tended to score higher than girls in Manual dexterity (p = .06). The sex difference was most prominent in the SSs of MD1 (boys: 11.41, girls: 9.41, p = .01).

**Discussion:** Although the components scores and the total score of Age Band 1 in the Japanese population were similar to those in the UK population, differences were found at a level of individual examination items. These differences might be associated with cultural backgrounds. Therefore, further investigation with a larger sample size is necessary to obtain the Japanese version of normative data. In addition, our results suggest that Japanese boys tend to be better at manual skills than girls.

**Keywords:** Movement Assessment Battery for Children-Second Edition (MABC2); Age Band 1; Japanese; Sex differences.

### Participation and quality of life of young adults with Developmental Coordination Disorder: A longitudinal study

M. Tal Saban^**1**^, A. Ornoy^**2,3**^ & S. Parush^**1**^

^1^School of Occupational Therapy, Hebrew University Hadassah Medical School, Jerusalem, Israel. miri.tal-saban@mail.huji.ac.il; ^2^Hebrew University Hadassah Medical School, Jerusalem, Israel; ^3^Israeli Ministry of Health, Jerusalem, Israel.

**Aim:** The purpose of this paper is to present a longitudinal study that designed to assess the continuing influence of DCD on quality of life and participation.

**Method:** The study used a randomized cohort (*N*=429) of young adults with DCD (n= 135), borderline DCD (*n*= 149) and control (*n*= 145), from a previous study. This initial cohort was asked to participate in a longitudinal follow-up study three to four years after the initial study. This study consisted of 96 individuals: 25 DCD (mean age = 24.35 years [SD=0.88]; 52% males); 30 borderline DCD (mean age = 24.48 years [SD=0.98]; 43.3% males) and 41 typical individuals (mean age = 25.82 [SD=1.91]; 48.8% males). Subjects completed the Participation in Every Day Activities of Life (PEDAL), the Life-Satisfaction Questionnaire (Li-Sat 9) and the World Health Organization Quality of Life instrument (WHOQOL-BREF).

**Results:** A MANOVA revealed a statistically significant between-group difference (*F*[7,95]=2.89; *p*=0.001; η=.173) and post-hoc analyses revealed that individuals in the DCD and borderline DCD groups received lower overall scores for participation, quality of life and life satisfaction. Pearson correlation coefficients yielded moderate to high correlations between scores. Linear regression found the psychological domain of the WHOQOL-BREF to be a significant predictor of life satisfaction (*B*=0.533; *p*=0.001).

**Discussion:** The results of this study indicate that the effects of DCD do not dissipate with age and continue to effect in young adulthood. Young adults with DCD are less satisfied with their quality of life and life in general and continue to report decreased participation in daily life activities.

**References**:

Hill, E. L., Brown, D., & Sorgardt, K. S. (2011). A preliminary investigation of quality of life satisfaction reports in emerging adults with and without developmental coordination disorder. *Journal of adult development, 18,* 130-134.

Kirby, A., Edwards, L., & Sugden, D. (2011). Emerging adulthood in developmental co –ordination disorder: parent and young adult perspectives. *Research in Developmental Disabilities, 32,* 1351-1360.

Tal- Saban, M., Ornoy, A., & Parush, S. (2014). Young adults with developmental coordination disorder: A longitudinal study. *American Journal of Occupational Therapy, 68*, 1–10.

**Keywords:** Participation; Quality of life; Longitudinal study; Young adults.

### Slovenian adaptation of the Developmental Coordination Disorder Questionnaires

J. Tercon^**1**^, T. Rihtman^**2**^ & B. N. Wilson^**3**^

^1^Faculty of Education, University of Ljubljana & Community Health Centre Ljubljana, Ljubljana, 1000, Slovenia. jerneja.tercon@pef.uni-lj.si; ^2^Hebrew University of Jerusalem, Jerusalem, 9190501, Israel & Coventry University, Coventry, CV1 5FB, United Kingdom; ^3^Alberta Health Services & University of Calgary, Calgary, AB T2N 1N4, Alberta, Canada.

**Aim:** There are several instruments to screen for children with developmental coordination disorder (DCD). Considering that proxy questionnaires (parental or teacher assessment of *child* abilities or behaviour) represent a simple and effective data collection method, Slovenian adaptations of the Developmental Coordination Disorder Questionnaire – DCDQ (Wilson & Crawford, 2007) and the Little Developmental Coordination Disorder Questionnaire – LDCDQ (Rihtman, Parush, & Wilson, 2011) were made.

**Method:** The purpose of this study was to assess the use of both Slovenian adaptations of the DCD questionnaires (DCDQ-SI and LDCDQ-SI) using the MABC or MABC-2 as a reference standard. The use of the DCDQ-SI was tested in two separate studies. In the first study, the DCDQ-SI was administered to 196 preschool teachers of five-year-old children who were assessed with the MABC. In the second study, the DCDQ-SI was completed by 135 parents of children aged 5 to 15 years, assessed by the MABC-2. Finally, the LDCDQ-SI was administered to 43 parents of kindergarten children aged 3 and 4, tested with the MABC-2.

**Results:** Based on the MABC/MABC-2 criteria for the identification of DCD, results showed statistically significant differences between the DCD and the non-DCD groups in both DCDQ-SI studies. Furthermore, cut-off scores that were made in both studies were determined higher than the cut-off scores proposed by the original version of the questionnaire. Preliminary results in the LDCDQ-SI analysis showed good prediction of motor difficulties in young children. Further research will be conducted on a larger sample, and the *psychometric properties* of the instrument will be established in the near future.

**Keywords:** Developmental Coordination Disorder; Screening; Cultural adaptation; Questionnaire.

### Measuring fatigue and related symptoms in adults with Developmental Coordination Disorder (DCD)

M. Thomas^**1**^, G. Christopher^**2**^, N. Williams^**1**^ & R. Heirene^**1**^

^1^The Dyscovery Centre, University of South Wales, Caerleon Campus, Newport, NP18 3QT, UK. marie.thomas3@southwales.ac.uk; ^2^Department of Health and Social Sciences, University of the West of England, Bristol, UK.

**Aim:** Although a normal result of over exertion, lack of sleep or disease states, fatigue is usually resolved by rest. Though, when fatigue is prolonged and unresolved it can be extremely debilitating. This preliminary study aims to be the first of its kind to investigate the level and nature of the fatigue experienced by adults with DCD. The study utilises the expertise of the co-authors (Thomas & Christopher) in the field of Chronic Fatigue Syndrome (CFS) research to investigate fatigue in DCD.

**Method:** Support groups for individuals with DCD and CFS (national and international) were contacted for their assistance in recruitment. Staff and students from the university were recruited as a typically developing (TD) non-CFS comparison group. Participants completed a batch of standardised measures known to be important markers in previous CFS research^1,2^ including: a) fatigue, b) measures of anxiety, depression and mood, c) self-esteem, e) cognitive failures, f) measures of symptoms and health behaviours, and g) perceived stress. Data were captured using the online resource Survey Monkey™. Ethical approval for the study was obtained from the university Research Ethics Committee.

**Results:** So far 39 adults with DCD, 51 with CFS, 6 with a dual diagnosis of DCD and CFS, and 21 TD non-CFS adults have taken part in this on-going study. Preliminary results indicate that the DCD adults have significantly higher levels of cognitive difficulties (p<0.001) and failures (p<0.001), fatigue (p<0.01), anxiety (p<0.01), depression (p<0.05) and symptoms (p<0.001) together with significantly lower levels of self-esteem (p<0.05) than the TD group. In terms of cognitive difficulties, fatigue and symptoms, the DCD groups reported significantly lower levels of each measure than the CFS group (p<0.05, p<0.001 and p<0.001 respectively). No significant differences were identified between those with DCD and those with both DCD and CFS on any measures; though mean scores were higher for the dual diagnosis group on measures of symptoms, anxiety, cognitive failures and difficulties, perceived stress, and fatigue.

**Discussion:** The aim of this ongoing study is to describe the level and nature of fatigue reported (anecdotally) by adults with DCD. Preliminary findings show adults with DCD report significantly greater levels of fatigue than controls, supporting previous research which has found those with DCD may fatigue earlier than their TD counterparts. This may be attributed to inefficient motor patterns – as suggested by Silman et al.^3^- or from elevated oxygen requirements^4^. Interestingly, 6 adults have taken part in the study with a dual-diagnosis of DCD and CFS, suggesting some overlap between the conditions. These findings have important clinical implications in terms of supporting those with DCD and suggest the interventions used to treat fatigue in those with CFS may also be efficacious in alleviating the fatigue experienced by those with DCD.

**References**:

1. Thomas, M., & Smith, A. (2006). An investigation of the long-term benefits of anti-depressant medication in the recovery of patients with chronic fatigue syndrome. *Clinical & Experimental, 21*, 1-8.

2. Fleming, J., James, S., & Watts, W. A. (1980). The dimensionality of self-esteem: some results from a college sample. *Journal of Personality and Social Psychology, 39,* 921-29.

3. Silman, A., Cairney, J., Hay, J., Klentrou, P., & Faught, B. E. (2011). Role of physical activity and perceived adequacy on peak aerobic power in children with developmental coordination disorder. *Human Movement Science, 30*, 672-681.

4. Faught, B. E., Rivilis, I., Klentrou, P., Cairney, J., Hay, J., & Liu, J. (2013). Submaximal oxygen cost during incremental exercise in children with developmental coordination disorder. *Research in Developmental Disabilities, 34*, 4439-4446.

**Keywords:** DCD; Chronic Fatigue Syndrome; fatigue; cognition; symptoms.

### Characteristics of motor overflow in children with Developmental Coordination Disorder

A. Thornton^**1**^, S. Reid^**1**^, C. Elliott^**2,3**^& M. Licari^**1**^

^1^School of Sport Science, Exercise and Health, The University of Western Australia, Perth, Australia; ^2^Department of Paediatric Rehabilitation, Princess Margaret Hospital for Children, Perth, Australia; ^3^Curtin University, Faculty of Health Sciences, Perth, Australia.

**Aim:** Children with Developmental Coordination Disorder (DCD) commonly present with unintentional motor activity, referred to as motor overflow, which accompanies the performance of voluntary movement. The relevance of these movements, interference with the execution of motor tasks and their diagnostic value in this population is poorly understood. This study aimed to investigate the degree and frequency of these movements.

**Method:** Forty three male and nine female children with DCD aged 8-10 years (*x^−^* = 9y1m+9m) participated in this study. Three subtypes of motor overflow were investigated: contralateral, bilateral and mimic using tasks from the Zurich Neuromotor Assessment (ZNA) and previous research. Where appropriate, motor overflow scores were compared to the ZNA normative sample. Additionally children were compared across three movement classifications established using the Movement Assessment Battery for Children -2; children with confirmed proficiency issues (<5th percentile) and children falling into borderline groups (6-10th percentile and 11-15th percentile).

**Results:** Statistical analysis revealed that overall, children with DCD displayed more severe motor overflow for both degree and frequency compared to the ZNA normative sample across all tasks, except for walking on toes. The most contralateral motor overflow was seen on pegboard task (*x^−^* = 2.00 + 0.78), closely followed by finger sequencing (*x^−^* = 1.94 + 0.65). Walking with feet externally rotated elicited the greatest bilateral motor overflow (*x^−^* = 2.58 + 0.61). When children were separated into motor proficiency classifications, those ranked in the lowest percentile displayed higher degree and frequency of motor overflow compared to borderline groups.

**Discussion:** Children with DCD consistently displayed motor overflow levels in excess of typically developing children. They displayed higher amplitudes of displacement and greater frequency. The presence of these movements is likely to reflect deficits in the development of inhibitory control mechanisms and is likely to compromise movement proficiency in this population.

**Keywords:** Motor overflow; Movement proficiency; Inhibitory control.

### Pilot Study: Assessing the usefulness of the Japanese Playful Assessment for Neuropsychological Abilities (JPAN) for identifying movement impairment in children with autism spectrum disorder

A. Tokunaga^**1**^, T. Higashionna^**1**^, A. Nakai^**2**^, K. Tanaka^**1**^, H. Nakane^**3**^, G. Tanaka^**1**^ & R. Iwanaga^**1**^

^1^Division of Physical Therapy and Occupational Therapy Sciences, Graduate School of Biomedical Science, Nagasaki University, 1-7-1 Sakamoto Nagasaki, 852-8120, Japan. akiko0923@nagasaki-u.ac.jp; ^2^Director, Department of Pediatric Neurology, Department of Pediatrics, & Department of Child and Adolescent Psychiatry, Hyogo Children’s Sleep and Development Medical Research Center, Nishi-ku, Kobe, Japan; ^3^Unit of Rehabilitation Sciences, Graduate School of Biomedical Science, Nagasaki University, Japan.

**Aim:** The aim of this study is to examine the usefulness of the Japanese Playful Assessment for Neuropsychological Abilities (JPAN) for assessment of movement skills in children with Autism Spectrum Disorder (ASD) compared to the Movement Assessment Battery for Children-2 (MABC-2).

**Method:** We tested 13 children with autism spectrum disorder (aged 6 to 12 years old), using the JPAN and the MABC-2.

**Results:** The results of this study showed that 4 children scored below -1SD on the JPAN subscale for Posture and equilibrium, 9 for Somatosensory, 5 for Hand-eye coordination and visual perception, and 5 for Praxis. On the MABC-2, the number of children scoring below -1SD was 1 for Manual Dexterity, 4 for Aiming & Catching, and 0 for the Balance component. The number of children who scored below -1SD was larger for JPAN total scores than for MABC-2 total scores. There were significant correlations; between total score on the MABC-2 and all domain scores of the JPAN, between total score on the Manual Dexterity component of the MABC-2 and all 4 domains of the JPAN, between Aiming & Catching components on the MABC-2 and Posture and equilibrium and Somatosensory on the JPAN, and between the Ball skills component of the MABC-2 and all domains of the JPAN except for Praxis.

**Discussion:** Although the JPAN has fine motor test components, which include Visual perception and Praxis, there is no independent domain for fine motor such as Manual Dexterity on the MABC-2. Therefore, to compare the scores of both tests is difficult. Only the Balance test on the MABC-2 and Posture and equilibrium on the JPAN might evaluate similar abilities. In the results, both of these test scores were significantly correlated. Furthermore, the number of children scoring below -1SD on the JPAN Posture and equilibrium was larger than Balance on the MABC-2. These differences might be caused by differences in test methods. Posture and equilibrium on the JPAN includes ‘Standing balance with eyes closed’ and ‘One hand one leg balance’ which is not in the MABC-2. Since the scores of these tests for most participants were below -1SD, low Posture and equilibrium scores on the JPAN may be a result of the larger number of components. Children with ASD are more likely to score poorly on these tests. Posture and equilibrium on the JPAN might evaluate similar skills to Balance on the MABC-2, and there is possibility that the JPAN is more sensitive to identifying balance problems. The large number of children with scores below -1SD on Praxis suggests that praxis problems may occur frequently in children with ASD. Since Praxis on the JPAN includes some fine motor tests and significantly correlates with Manual Dexterity on the MABC-2, lower Praxis scores might reflect not only praxis skills, but also fine motor dysfunction. Although the JPAN has different tests and evaluates motor function in a different way compared to the MABC-2, it may be able to detect balance and praxis problems in children with ASD as well as the MABC-2. Further studies with a larger sample size should be conducted to validate this initial research.

**Reference**:

The Japanese Academy of Sensory Integration (2011). *Japanese Playful Assessment for Neuropsychological Abilities*. Japan: Pacific Supply Co. (in Japanese)

**Keywords:** Movement Assessment Battery for Children-2; Japanese Playful Assessment for Neuropsychological Abilities; Autism spectrum disorder.

### Deficits of visuospatial attention with reflexive orienting in children with Developmental Coordination Disorder: A time-frequency EEG study

C.L. Tsai^**1**^, C.H. Wang^**1**^, C.Y. Pan^**2**^, F.C. Chen^**3**^, Y.H. Lo^**1**^ & W.K. Liang^**4**^

^1^Institute of Physical Education, Health & Leisure Studies, National Cheng Kung University, Taiwan, NO. 1, University Road, Tainan City 701, Taiwan. andytsai@mail.ncku.edu.tw; ^2^Department of Physical Education, National Kaohsiung Normal University, Taiwan; ^3^Department of Recreational Sport and Health Promotion, National Pingtung University of Science and Technology, Taiwan; ^4^Institute of Cognitive Neuroscience, National Central University, Taiwan.

**Aim:** The purpose of the present study was to explore and compare the behavioural performance and electrophysiological correlates of attentional orienting in children with developmental coordination disorder (DCD) and typically developing (TD) children when performing the visuospatial attention task with reflexive/automatic (exogenous) orienting.

**Method:** Twenty children with DCD and 20 TD children were recruited, and their behavioral performance and brain activity were recorded during a variant of the visuospatial attention paradigm which relies on mechanisms associated with reflexive/automatic shifts of attention, while simultaneously considering the behavioural and neural oscillations.

**Results:** Although no significant difference was found in the response accuracy between the two groups, children with DCD exhibited longer reaction time across conditions and larger attentional cost when compared to TD children. In addition, the TD group showed stronger theta power under both valid and invalid conditions relative to that exhibited in the neutral condition. However, such performances were found to be absent in the DCD group.

**Discussion:** The current study reveals the task-related modulation of theta power and demonstrates that the poor behavioural (e.g., RTs) performance along with weaker theta power may account for the deficit of the ventral attention network in DCD children, implying that the medial frontal theta oscillation appears to be a good index to investigate the deficit of attentional orienting in children with DCD.

**Keywords:** Attention; Developmental Coordination Disorder; Behaviour; Neural oscillations.

### Importance of multidimensional developmental assessments to define subtypes and specific impairments of Developmental Coordination Disorder

L. Vaivre-Douret^**1,2,3,4**^, C. Lalanne^**5**^ & B. Golse^**1,2,4**^

^1^Department of Medicine, University of Paris Descartes, Sorbonne Paris City 75006, Paris, France; ^2^INSERM UMR 1178, University of Paris Sud and Paris Descartes Paris, France. laurence.vaivre-douret@inserm.fr; ^3^Department of Pediatrics, AP-HP Paris Center Port Royal-Cochin Hospital, Paris, France; ^4^Department of Child Psychiatry, AP-HP Necker-Enfants Malades University Hospital, IMAGINE affiliation, Paris, France; ^5^Patient-Centered Outcomes Research, Paris Sorbonne Cité, EA 7334 (REMES), University of Paris Diderot and Saint-Louis Hospital, Paris, France.

**Aim:** Developmental Coordination Disorder (DCD) involve a marked impairment in the development of motor coordination although visuo-spatial, digital and visuo-motor perception, qualitative and quantitative developmental measures^1^ of neuromuscular tone, gross and fine motor coordination-related impairments might be used to isolate three main subtypes of DCD/dyspraxia: ideomotor, visuo-spatial and constructional, and a mixed group (MX) sharing common impairments with additional comorbidities^2,3^. The MX group appears as an umbrella of motor disorders, found in clusters studies. This study focus on isolating specific diagnosis markers with high predictive discriminatory power of MX vs. pure form of DCD/dyspraxia.

**Methods:** Data were collected on 63 children with DCD aged 5-15 years (median 8.1), enrolled on DSM-5 criteria with a strict inclusion (full term, free of remediation and of medical abnormalities). Each subject underwent a neuropsychological, neuro-psychomotor (NP-MOT developmental battery), and neurovisual testing battery totalling 49 milestones assessment scored as pass/fail variables. A classification tree incorporating bagging was used to rank those variables, either alone or in interaction with other variables, by their relative importance in classification accuracy of clinical subgroups. Model calibration (number of leaves and number of trees) was done on a training sample through nested repeated 5-fold cross-validation while predictive performance was assessed on a held-out validation sample, using a split ratio of 0.7/0.3.

**Results:** The most salient markers with respect to the three subtypes studied in this sample are digital praxia, imitation of gestures, digital perception, visual-motor integration, manual dexterity, visual spatial structuration, coordination between upper and lower limbs, and lego blocks. Specific interactions among those predictors and other impairments (motor pathway, visual evoked potential, language) were shown to provide additional insights into DCD subtyping. The Mix group shows specific difficulties in coordinating lower and upper limbs or poor manual dexterity.

**Discussion:** Taylored follow-up of patients presenting with DCD should consider the specificity of neuro-sensory-motor, visuo-spatial, and neuropsychological impairments of which co-occurrence allows to define different subtypes of DCD. Less than 15 milestone tests might be required to provide a sensitive and specific diagnostic of DCD subtypes, and isolated markers allow a better understanding of DCD. The five most important predictors appeared to be Imitation gestures, Digital praxia, Arithmetic, Visuo-motor integration and Digital perception. Indeed, the choice of appropriate measures has an impact on understanding of the nature and etiology of disorders. Investigations involving standardized neuro-developmental assessments with qualitative and quantitative measures are necessary.

**References**:

1. Vaivre-Douret, L. (2006). *Batterie d’évaluation des fonctions neuro-psychomotrices (NP-MOT)*. Paris: Editions du Centre de Psychologie Appliquée.

2. Vaivre-Douret, L., Lalanne, C., Ingster-Moati, I., Boddaert, N., Cabrol, D., Dufier, J.-L. (2011). Subtypes of Developmental Coordination Disorder: Research on their nature and etiology. *Developmental Neuropsychology, 36*(5), 614-643.

3. Vaivre-Douret, L., Lalanne, C., Cabrol, D., Ingster-Moati, I., Falissard, B., & Golse, B. (2011). Identification de critères diagnostiques des sous-types de troubles de l’acquisition de la coordination (TAC) ou dyspraxie développementale. *Neuropsychiatrie de l’Enfance et de l’Adolescence, 59*(8), 443-453.

**Keywords:** Developmental Coordination Disorder; Dyspraxia; Developmental assessment; Subtypes; Diagnosis markers.

### Intervention effect in fundamental motor skills of children with Developmental Coordination Disorder and at risk, and typically developing children

N.C. Valentini^**1**^, L.W. Zanella^**1**^, M.S. de Souza^**1**^& M.J. Kim^**2**^

^1^School of Physical Education, Federal University of Rio Grande do Sul, Porto Alegre, 91760210m, Brazil. nadia.cristina@ufrgs.br; ^2^Institute of Health Science, Korea University, Seoul, Korea.

**Aim:** The purpose of the study was to investigate the effect of a motor skill intervention (MSI) on the fundamental motor skills (FMS), coordination, and strength and agility in children with DCD and risk of DCD (r-DCD), and typically developing children (TD).

**Method:** 48 children (5 to 7 year-old) were randomly assigned to intervention (IG: N=24) and control groups (CG: N=24). MABC-2 was used to assess motor development and categorize groups and TGMD-2 and BOT-2 were adopted d to assess intervention effect. The MSI implemented a Mastery Motivational Climate using the TARGET (Task, Authority, Recognition, Group, Evaluation, Time). MSI was consisted of 32 lessons, 2 lessons per week during 16 weeks and researchers provided 70-minute lesson for each lesson. CG participated in physical education classes focusing on low oriented fun activities.

**Results:** There were significant interactions in locomotor skills (LOC: p<.001), object control skills (OC: p<.001), motor quotient (MQ: p<.001), manual coordination (MC: p=.006), body coordination (BC: p=.045), strength and agility (SA: p=.031), and total motor composite (TMC: p<.001). Nothing was found in fine motor coordination (FMC). Post hoc tests suggested that: (1) IG with DCD showed superior performance than CG with DCD at the post test in LOC (p=.016) and MQ (p=.013), and progressed in LOC (p=.009), OC (p=.003) and MQ (p=.021). IG with DCD showed declines in MC (p=.003) over time, whereas CG children with DCD showed declines in QM (p=.010) and MC (p<.001) over time; (2) IG children at r-DCD showed superior performance at the post test in LOC (p=.040) and OC (p=.001) compared to CG at r-DCD. Also IG at r-DCD progressed in the LOC (p=.015), OC (p=.011), MQ (p<.001), and TMC (p=.005), and showed decline in MC (p=.002) over time. However, no positive changes were observed for the CG children at r-DCD (p<.05); (3) TD from IG showed superior performance than TD from CG in LOC (p<.001), OC (p<.001), MQ (p<.001), FMC (p=.036), MC (p=.004), SA (p=.014), and TMC (p<.001), progressed in LOC (p<.001), OC (p<.001), MQ (p<.001), and TMC (p=.001), and decline MC (p<.001) over time. Whereas TD from CG showed decline in LOC (p=.016), MQ (p=.016), MC (p<.001), SA (p=.011), and TMC (p=.009).

**Discussion:** The recent results suggested that MSI for children should be consistently organized in the tasks, implemented high levels of challenge, engagement children in the program organization and protocols in order to promote motor skill and coordination development, and improvement children strength and agility.

**References**:

Pless, M., & Carlsson, M. (2000). Effects of motor skill intervention on developmental coordination disorder: A meta-analysis. *Adapted Physical Activity Quarterly*, *17*(4), 381-401.

Wright, H. C., & Sugden, D. A. (1998). A school based intervention programme for children with developmental coordination disorder. *European Journal of Physical Education*, *3*(1), 35-50.

**Keywords:** Intervention; Motor skill intervention; Developmental coordination disorder; at riks of DCD.

### Implicit motor timing in children with Autism Spectrum Disorder

H. Van Waelvelde, L. de Caluwé, T. Taymans & J. Debrabant

Department of Rehabilitation Sciences and Physiotherapy, Ghent University, De Pintelaan 185, 9000 Gent, Belgium. hilde.vanwaelvelde@ugent.be

**Aim:** A dysfunction in implicit motor timing has already been identified as an underlying mechanism of motor problems in children with DCD. Implicit motor timing refers to the implicit acquisition of a precise timing of visuo-motor responses at a steady rhythm. The current study aims to confirm the relationship between motor problems and implicit motor timing in a group of children clinically diagnosed as children with Autism Spectrum Disorder (ASD).

**Method:** Participants were 23 children with ASD and a matched control group of 21 typically developing (TD) children between 8- and 12-years-old. Motor tests (M-ABC-2, VMI copying, VMI tracing, VMI visual perception, KTK (jumping item) and the short-form of the WISC-III IQ test were administered next to the visuo-motor reaction time task (VRT). The VRT requires speeded index responses at a visual stimulus, a red blowfish cartoon, by pressing a laptop space button. In a regularly visual pacing block, 12 stimuli with fixed inter stimulus interval (ISI) of 1200ms were presented whereas in an irregularly paced block, stimuli were presented with random ISIs (900 – 1050 – 1200 – 1350 - 1500 ms). The average presentation rate is identical in both visual blocks, i.e. one stimulus every 1200 ms. Children are not informed about the difference between both conditions. Outcome measures of VRT are mean reaction times in series of regularly paced stimuli (fixed condition) and series of irregularly paced stimuli (random condition) and percentages anticipatory responding (RT<100ms) in both conditions. A GLM for Repeated Measurements was used to compare conditions and groups. Post-hoc paired T-Tests were used to compare conditions within groups. VRT measures were correlated with motor and visual perception indices and IQ.

**Results:** Fifteen children with ASD scored at or below Pc 16 and 2 TD children scored at the 16th Pc on M-ABC-2. IQ scores did not differ significantly between groups. In contrast to TD children, the children with ASD did not show reaction time advantage in the fixed condition and their anticipatory reactions did not differ significantly across both conditions. Within the group of children with ASD the difference in mean RT between both conditions was not significantly correlated with KTK jumping side to side or VMI visual perception, but it was significantly correlated with VMI copy task (r=0.72) and VMI tracing task (r=0.42), as it was with IQ (r =0.42).

**Discussion:** Children with ASD show impaired implicit motor timing abilities which are supposed to contribute to their motor problems and more specifically to complex visual spatial motor tasks. But this timing ability seems also to be related to IQ. The strong correlation between the VMI copy task and implicit motor timing is a result confirming the findings of previous studies in children with DCD and in children with Neurofibromatosis-1. More research is necessary to unravel the meaning of implicit motor timing in human behavior.

**Keywords:** Implicit; Timing; Autism spectrum disorder.

### Criterion validity of the german Bruininks-Oseretsky Test of Motor Proficiency-2nd edition

S. Vinçon^**1,2**^, D. Green^**3**^, R. Blank^**4,5**^ & E. Jenetzky^**4,6,7**^

^1^Department of Occupational Therapy, Child Centre Maulbronn, 75433 Maulbronn, Germany. s.vincon@kize.de; ^2^Department of Sport and Health Sciences, Oxford Brookes University, United Kingdom; ^3^Centre for Rehabilitation, Oxford Brookes University, United Kingdom; ^4^Department of Pediatrics, Child Centre Maulbronn, Germany; ^5^University of Heidelberg, Germany; ^6^Divison of Clinical Epidemiology and Aging Research, German Cancer Research Center, Heidelberg, Germany; ^7^Department of Child and Adolescent Psychiatry, University Medicine Mainz, Germany.

**Aim:** The Bruininks-Oseretsky Test of Motor Proficiency-2 (BOT-2) is an established assessment for measuring the motor abilities of children and youth for clinical and research purposes. A version, standardized for German speaking countries (BOT-2 G) is now available. The motor proficiency of children develops through performance of daily activities, which require fine and gross motor abilities. Assuming that the motor capability of children is linked with fine and gross motor activities, there should be a relationship between performance of results of the BOT-2 G and various daily motor activities. Therefore the aim of the study is to evaluate the criterion validity of the BOT-2 G for six daily motor activities.

**Method:** Data from 1177 children and their parents, were obtained from the BOT-2 G standardization study. Purposive questions were included to determine parental ratings of their child’s general fine and gross motor abilities and specific skills in six fine and gross motor activities (drawing, writing, arts and crafts, driving scooter/bicycle, ball games, general sports). Parental ratings across skills seen in daily activities at home and school will be contrasted to corresponding subtests and motor areas of the BOT-2 G. Analysis of Variance (ANOVA) between the three subgroups of children rated as worse, same or better than peers by their parents in their motor abilities, will be used to consider the extent to which the BOT-2 G total scores or domain scores reflect abilities seen in daily life activities.

**Results:** The results of this study will ascertain whether there is a relationship between the BOT-2 G and the perceived competence in typical daily activities as evaluated by parents. It is hypothesized that scores on the motor scales of the BOT-2 G will show significant associations between parents ratings, but the associations may vary due to the different contexts in which these activities are performed in daily life (e.g. additional social challenges).

**Discussion:** Information regarding the criterion validity of the BOT-2 G will assist diagnostic formulation of Developmental Coordination Disorder.

**References**:

Blank, R., Jenetzky, E., & Vinçon, S. (Ed.) (2014). *Bruininks-Oseretzky Test der motorischen Fähigkeiten – Zweite Ausgabe (BOT-2) nach R.H. Bruininks und B.D. Bruininks*. Frankfurt: Pearson Assessment.

Bruininks, R. H., & Bruininks, B. D. (2005). *Bruininks-Oseretsky Test of Motor Proficiency – Second Edition (BOT-2)*. NCS Pearson, Inc.

**Keywords:** BOT-2; German BOT-2; Criterion validity.

### Norm values for writing speed in German pupils

S. Vinçon^**1,2**^, B. Smits-Engelsman^**3**^, B. Schleifer^**4**^, R. Blank^**4,5**^ & E. Jenetzky^**5,6,7**^

^1^Department of Occupational Therapy, Child Centre Maulbronn, 75433 Maulbronn, Germany. s.vincon@kize.de; ^2^Department of Sport and Health Sciences, Oxford Brookes University, Oxford, United Kingdom; ^3^University of Leuven, Faculty of Kinesiology and Rehabilitation Science, Leuven, Belgium; ^4^University of Heidelberg, Heidelberg, Germany; ^5^Department of Pediatrics, Child Centre Maulbronn, Maulbronn, Germany; ^6^Divison of Clinical Epidemiology and Aging Research, German Cancer Research Center, Heidelberg, Germany; ^7^Department of Child and Adolescent Psychiatry, University Medicine Mainz, Mainz, Germany.

**Aim:** Handwriting is one of the most complex of fine motor skills and therefore often results in activity and participation problems for children with DCD. Besides problems with legibility, writing speed is often limited. The Dutch assessment ‘Systematische opsporing van schrijfmotorische problemen’ (SOS-2) reflects both aspects of handwriting. Until now no assessment for German speaking countries has been available, therefore a German adaptation called ‘Systematische Erfassung motorischer Schreibstörungen’ (SEMS) was created. This study aimed to analyze underlying factors of writing speed and to develop normative values for German children between 6 and 14 years.

**Method:** Comparable to the Dutch version, a German version of the SOS-2 was created. For this purpose a structured German text was developed, including all letters of the alphabet, to be copied in five minutes. The study sample was part of the German BOT-2 standardization. 40% of this subsample came from South-West of Germany, and half of this subsample were primary school pupils. Possible factors predicting the writing speed were analyzed.

**Results:** In total 262 healthy children (49% male) between 6 and 14 years without specific disabilities performed this test. Due to strong linear relation between age and writing speed, linear transformation was possible. Age explained up to 72% of variance; with speed increasing about 50 letters each year in strong linear manner. In direct comparison with the Dutch norm values, the German median values are only marginal faster. Adjusted for age, girls were about 29 letters faster than boys in the younger age group (6-10 years; p=0.002) and 54 letters faster in the older age group (11-14 years; p=0.001). In this older age group results differ regarding secondary school types (three levels in Germany). Children aged 11-14 years from level 1 secondary school were up to 57 letter (p=0.01) faster than children (11-14 years) from level 3 secondary school. The strongest correlation (r=0.37) was found with the third BOT-2 subtest (manual dexterity) using gender specific norms explaining 14% of variance.

**Discussion:** The first German norm values regarding normal writing speed development are now available. Age is the most important factor of writing speed with a strict linear development. This strict linear relationship allows smaller sample sizes for norm values. But as well other predicting factors like gender and level of school ability should be considered. In direct comparison with BOT-2 composite fine manual control, motor seem to be independent from writing speed. The strongest correlation was found with speed depended assessments. Writing speed is not well considered in most motor assessments, hence additional standardized assessments are needed.

**Keywords:** Handwriting; Norm values; SOS-2; SEMS.

### How do symbolic and non-symbolic numerical magnitude processing and working memory relate to mathematical skills of children with Developmental Coordination Disorder?

M.J.M. Volman

Department of Special Education, Utrecht University, Heidelberglaan 1, 3534CS, Utrecht, the Netherlands. m.volman@uu.nl

**Aim:** It is well known that children with DCD often have co-morbid learning disabilities including mathematical learning problems. It has been suggested that mathematical skills are influenced by domain-general (e.g., working memory) and by domain-specific (e.g., numerical magnitude processing) factors. Numerical magnitude processing is considered an important building block for higher-level numerical skills and was found to relate to the level of achievement in typical and atypical mathematical development^1^. In children with DCD, working memory appeared to be significantly associated with numeracy^2^. The aim of the present study is to investigate the role of symbolic and non-symbolic numerical magnitude processing and working memory on the mathematical skills of children with DCD and age-matched peers.

**Method:** Twenty-five children with DCD (7-12 years) and 48 age-matched peers participated. Math performance was assessed with a Dutch math skills test. Didactic age (DA) and didactic age equivalent (DAE) score were expressed in a Math Learning Efficiency percentage score (*MLE* = DAE/DA*100). Numerical magnitude processing skills were assessed with a non-symbolic magnitude comparison task (Panamath) and a symbolic number line estimation task. Visuo-spatial and verbal working memory were measured with the Automated Working Memory Assessment (AWMA) Battery. Hierarchical regression analysis was applied with domain-general variables (working memory) and domain-specific variables (magnitude processing) entered, respectively.

**Results:** Fifteen children with DCD (60%) and 8 control children (16%) were lagging behind on math skills for more than one year. The DCD group scored significantly lower on math skills (*p* < .01), on visuo-spatial and verbal working memory (both *p* < .01), and on symbolic and non-symbolic numerical magnitude comparison (both *p* < .05) compared to the control group. Only in the DCD group, the *MLE* score was significantly related to visuospatial and verbal working memory and to symbolic and non-symbolic magnitude processing. Hierarchical regression analyses revealed that visuospatial working memory was a significant predictor of *MLE* score of children with DCD (*R*^2^ = .25), and that symbolic and non-symbolic magnitude comparison significantly contributed to the explained variance in the *MLE* score (*p* < .05) (*R*^2^change = .20).

**Discussion:** Children with DCD have relatively more problems with mathematical skills compared to age-matched peers. Both domain-general working memory processes and domain-specific numerical magnitude processing contribute to these poorer mathematical skills in children with DCD.

**References**:

1. De Smedt, B., Noël, M. P., Gilmore, C., & Ansari, D. (2013). The relationship between symbolic and non-symbolic numerical magnitude processing and the typical and atypical development of mathematics: Evidence from brain and behavior. *Trends in Neuroscience and Education, 2,* 48–55.

2. Alloway, T. P. (2007). Working memory, reading, and mathematical skills in children with developmental coordination disorder. *Journal of Experimental Child Psychology, 96*(1), 20–36.

**Keywords:** Learning disabilities; Mathematical skills; Numerical magnitude processing; Working memory.

### Motor coordination and emotional behavioural problems in 4- and 5-year old children

T.J. Wade^**1**^, C. Rodriguez^**2**^, C. Missiuna^**3**^, B. Timmons^**4**^ & J. Cairney^**2**^

^1^Department of Health Sciences, Brock University, 500 Glenridge Ave, St. Catharines, Ontario, L2S 3A1, Canada. twade@brocku.ca; ^2^Department of Family Medicine, McMaster University, Hamilton, Canada; ^3^School of Rehabilitation Science, McMaster University, Hamilton, Canada; ^4^Department of Pediatrics, McMaster University, Hamilton, Canada.

**Aim:** Developmental Coordination Disorder (DCD) has been linked to higher levels of behavioural problems among middle school age children and adolescents. The current study examines the link between children at risk for DCD (DCDr) and behavioural problems among younger children. Furthermore, we examine whether DCDr children have an increased likelihood of having multiple emotional/behavioural problems.

**Method:** Participants were a part of the larger Coordination and Activity Tracking in CHildren (CATCH) study (n=148; 65% male; 50% DCDr). ANOVA and MANOVA as well as contingency tables and logistic regression were used to compare mean differences as well as likelihood of reaching clinical thresholds across groups, respectively.

**Results:** DCDr children had significantly higher scores on CBCL syndrome scales: withdrawn (p=0.02), emotionally reactive (p=0.03), aggression (p=0.02), attention problems (p<.01), sleep problems (0.04) and other behavioural problems (p<0.01) as well as on the overall internalizing (p=0.04), externalizing (p<0.01) and total scales (p<0.01). Examining clinical cut-offs, the DCDr group were significantly more likely to have either borderline clinical or clinical scores on withdrawn (OR=9.0; 95%CI=1.1, 74.8; p=0.04), attention (OR=5.9, 95%CI=1.6-21.7; p<.01) and aggression (OR=8.8; 95%CI=1.0-75.0; p=0.047) subscales, as well as the total CBCL scale (OR=12.9; 95%CI=1.5-108.1; p=0.02) adjusting for age and sex. With respect to DSM-V criteria, the DCDr group had significantly higher scores on depression problems (p=0.01), autism spectrum problems (p=0.02), oppositional defiant problems (p=0.04) and attention deficit/hyperactivity problems p<0.01). There was no difference between groups on anxiety problems. Using clinical cut-offs, the DCDr group was significantly more likely to have borderline clinical or clinical scores on depression (FE<0.01). Likelihood of being identified clinically approached significance (p<.10) for autism spectrum problems and attention deficit/hyperactivity problems. There were no differences between groups for anxiety problems or oppositional defiance problems. A final analysis examined the cumulative clinical scoring across both CBCL syndrome scales and DSM-V oriented scales across groups. For both sets of measures, DCDr children were significantly more likely to have multiple clinical and borderline clinical indications.

**Discussion:** These results indicate that the co-occurrence between DCDr children and behavioural concerns is manifested in children as young as 4 years of age compared to their TD peers. The higher likelihood of having multiple co-morbid behavioural problems among those with DCDr early in life raises concerns about long-term emotional health and development in this population.

**References**:

Cairney, J., Veldhuizen, S., & Szatmari, P. (2010). Motor coordination and emotional–behavioral problems in children. *Current Opinion in Psychiatry, 23*, 324–329 [Electronic version]

King-Dowling, S., Missiuna, C., Rodriguez, M. C., Greenway, M., & Cairney, J. (2015). Co-occuring motor, language, and emotional-behavioral problems in children 3-6 years of age. *Human Movement Science, 39*, 101-108.

**Keywords:** Developmental Coordination Disorder; Early childhood; Preschool; Comorbidity; Co-occurrence; Behaviour; CBCL.

### Familial and nonshared environmental influences on the association between coordination difficulty and anxiety and depression symptoms in adult twins and siblings

M.A. Waszczuk^**1**^, H.C. Leonard^**2**^, E.L. Hill^**2**^, R. Rowe^**3**^ & A.M. Gregory^**2**^

^1^MRC Social Genetic and Developmental Psychiatry Centre, Institute of Psychiatry, Psychology and Neuroscience, King’s College London, De Crespigny Park, SE5 8AF, London, UK. monika.waszczuk@kcl.ac.uk; ^2^Department of Psychology, Goldsmiths, University of London, New Cross, London, UK; ^3^Department of Psychology, University of Sheffield, Western Bank, Sheffield, UK.

**Aim:** Increased levels of anxiety and depression have been reported in individuals with Developmental Coordination Disorder (DCD). However, little is known about the aetiology of these associations. The current study aimed to assess genetic, shared and nonshared environmental influences on the association between poor coordination and symptoms of anxiety and depression using a sample of adult twin and sibling pairs.

**Method:** Data were from a longitudinal twin and sibling study, Genesis 12-19, when participants were aged 22-32 (N=858). Participants completed questionnaires which included a question about their coordination (in which they could report feeling very, somewhat, a little, or not at all uncoordinated) and the adult versions of the Revised Child Anxiety and Depression Scale, and the Short Mood and Feelings Questionnaire. Data were analysed using multivariate quantitative genetic models.

**Results:** Coordination difficulty was due to 50% genetic influences (A), 1% shared environmental influences (C) and 49% nonshared environmental influences (E), but due to low power, the A and C influences were non-significant. To address this limitation and aid interpretability, AE models were fitted where A was significant and should be interpreted as familial liability (A and C). The phenotypic association between coordination and anxiety and depression symptoms was largely underpinned by shared familial liability for the three traits (rA=.58-.79, CI: .36-1.00) that accounted for most of the phenotypic correlation (A=.65-.74, CI: .36-1.00). The nonshared environmental influences on coordination difficulty and anxiety/depression symptoms were moderately correlated (rE=.18-.29) and accounted for about a third of the phenotypic association (E=.26-.35).

**Discussion:** The results show that both familial (genetic and shared environmental) and nonshared environmental influences play an important role in the aetiology of coordination difficulty. The results support previous research suggesting an association between coordination and symptoms of anxiety and depression, and extend it by highlighting a substantial shared familial liability for the three traits. Future research should examine the potential biological and environmental pathways shared between these symptoms.

**Keywords:** Anxiety; Depression; Familial; Environmental; Coordination; DCD.

### A secondary analysis of attention during motor tasks in children with Developmental Coordination Disorder (DCD) - with and without comorbid Attention Deficit Hyperactivity Disorder (ADHD)

K. Wlodarczyk^**1,2**^, J.G. Zwicker^**2,3**^, D. McCauley^**2**^, J. Cairney^**2,4**^ & C. Missiuna^**1,2**^

^1^School of Rehabilitation Science, McMaster University, Hamilton, Ontario, L8S 1C7, Canada. wlodarka@mcmaster.ca; ^2^CanChild, McMaster University, Hamilton, Ontario, Canada; ^3^Department of Occupational Science and Occupational Therapy, University of British Columbia, Vancouver, British Columbia, Canada; ^4^Department of Family Medicine, McMaster University, Hamilton, Ontario, Canada.

**Aim:** DCD may occur in the absence of other neurological disorders but is often comorbid with ADHD. To date, there is insufficient knowledge about the neurological underpinnings that mediate the symptoms of DCD, let alone explain the comorbidity. Similar neural structures have been implicated in fine and gross motor tasks for children with DCD^1^ and ADHD^2^ relative to their typically developing peers. Therefore, it is unclear whether ADHD is secondary, co-occurring or comorbid with DCD. Understanding the challenges children with DCD have during attention and motor tasks, relative to children with a comorbid ADHD diagnosis, may help define the role attention plays in DCD and provide insight to the neural correlates of behaviour. This paper examined attention as a secondary mechanism of a motor response during standardized testing.

**Method:** Secondary data analysis was conducted with 122 children (9–13 years) from a non–clinical population–based study who met diagnostic criteria for DCD (*n* = 54 ADHD; 68 DCD only). The Movement Assessment Battery for Children was used to determine whether group differences existed for motor skills

**Results:** Independent samples t-tests were conducted. Significant group differences were not found for overall scores; however, group differences existed for manual dexterity subtests. More specifically, children with DCD (*M* = 2.13, *SD* = 1.98) performed significantly better than students with a combined diagnosis (*M* = 3.02, *SD* = 1.87) on flower trails (*t*1,120 = 2.54, *p* = .012). A trend was identified for the shifting pegs tasks where children with DCD performed slightly worse (*M* = 3.60, *SD* = 2.17) than peers with a comorbid diagnosis (*M* = 2.94, *SD* = 1.96; *t*1,120 = 1.743, *p* = .084). These findings may suggest that some motor problems in children with a comorbid diagnosis may be accounted for by attention, specifically for tasks that involve fine motor and cognitive control.

**Discussion:** The impact of ADHD on specific motor tasks in children with DCD will be discussed. These results highlight performance differences when a greater demand for attention is required for fine motor tasks. Improved understanding of the influence attention has on motor control and motor learning will facilitate future research on the underlying neural mechanisms responsible for behaviour, and may assist in identifying rehabilitative strategies for children with DCD.

**References**:

1. Wilson, P. H., Ruddock, S., Smits-Engelsman, B., Polatajko, H., & Blank, R. (2013). Understanding performance deficits in developmental coordination disorder: a meta-analysis of recent research. *Developmental Medicine and Child Neurology*, *55*(3), 217–28. doi:10.1111/j.1469-8749.2012.04436.x

2. Halperin, J. M., & Schulz, K. P. (2006). Revisiting the role of the prefrontal cortex in the pathophysiology of attention-deficit/hyperactivity disorder. *Psychological Bulletin*, *132*(4), 560–81. doi:10.1037/0033-2909.132.4.560

**Keywords:** ADHD; Attention; M–ABC; Neural correlates; Motor control.

### Classroom Strategies and Support for Children with Developmental Coordination Disorder

K. Wlodarczyk^**1,2**^, S. Bennett^**1,2,3**^, N. Pollock^**1,2**^, L. Dix^**1,2**^, W. Campbell^**1,2**^, S. Sahagian Whalen^**1**^ & C. Missiuna^**1,2**^

^1^School of Rehabilitation Science, McMaster University, Hamilton, Ontario, L8S 1C7, Canada. wlodarka@mcmaster.ca; ^2^CanChild, McMaster University, Hamilton, Ontario, Canada; ^3^Faculty of Education, Brock University, St. Catharines, Ontario, Canada.

**Aim:** In Canada, children with developmental coordination disorder (DCD) attend mainstream schools and do not typically receive any special education support. Given that 1 to 2 children in every class may have DCD, evidence-based management is required to ensure that educators are informed about and can accommodate children with DCD. In this study, occupational therapists (OTs) worked collaboratively with educators to build knowledge and improve children’s outcomes by using problem-solving strategies. The purpose of this paper is to examine the type and frequency of strategies and accommodations that OTs shared with educators to encourage differentiated instruction for these children.

**Method:** Fifteen OTs worked closely with over 300 educators in classrooms (1 day/week per school) to identify issues and develop strategies to accommodate the learning and environmental needs of children with DCD. In the first year, OTs provided service to nearly 600 children with probable DCD and prepared summary reports for each child. Using a systematic chart review, data from the reports of 392 children participating in an evaluation of this service was used to quantify and categorize the strategies that were found to improve their participation at school.

**Results:** Environmental and compensatory strategies were identified in the domains of self–care, written productivity, leisure/sports and environment. With respect to improving ability in these domains, preliminary analyses indicate that OTs had fewer overall recommendations for self–care; strategies were primarily recommended in the areas of printing, gym class and seating arrangements. OTs recommended more child specific strategies to improve motor function at school than at home with the parent or child. Ongoing OT support and services to address motor concerns were encouraged for the second year.

**Discussion:** Specific strategies recommended by OTs are geared primarily to, and are important for, all educators. Modifying tasks, altering expectations, teaching strategies, changing the environment, or helping by understanding the child’s needs (e.g., MATCH) are ways to differentiate instruction and improve functional outcomes, not only for children who have DCD, but for all children who experience fine and gross motor challenges in mainstream classrooms.

**Keywords:** Management; School–based intervention; School, Occupational Therapy; Strategies.

### Sleep characteristics of children with movement impairments

K.E. Wright, A.L. Thornton, M.K. Licari, S.L. Reid & B.J. Furzer

UWA Paediatric Exercise Health Research Group, School of Sport Science, Exercise and Health, The University of Western Australia, WA, Australia. kemi.wright@uwa.edu.au

**Aim:** Sleep has been identified as a key element to the healthy growth and development of children. Disturbances in sleep have been associated with cognitive deficits, poorer academic performance, behavioural problems and mood disturbances. There has also been emerging evidence that sleep plays a vital role in motor skill learning and memory consolidation. There has been limited research into the sleep characteristics of children with movement impairments. Preliminary studies suggest children with movement impairments have greater sleep disturbance than typically developing children however, to date, sleep has not been objectively measured in this population. The aim of this pilot study is to determine the feasibility of objectively measuring the sleep characteristics of children with motor impairments using tri-axial accelerometers.

**Method:** 20 children aged 7-12 years old with were recruited from community based exercise programs. Children were assessed for motor proficiency using the Movement Assessment Battery for Children-2 (MABC-2), those who fell below the 15th percentile were considered to have movement impairments. Sleep characteristics were assessed via Actigraph monitors, which were worn on the wrist for a period of 7 days, with a parent/guardian completing sleep behaviour diaries on behalf of their child. Outcome measures were sleep duration, sleep onset latency, sleep efficiency and wake time after sleep onset. Additionally, compliance and feasibility with the use of Actigraph monitors was assessed through parent report and analysis of Actigraph data.

**Results & Conclusion:** Data collection and analysis are currently in progress. We hypothesize the sleep profiles of children with movement impairments will differ from normal values. Comprehensive results and conclusions from this study will be presented at the conference.

**Keywords:** Movement impairments; Sleep; Accelerometry.

### The relationship among motor development, BMI, gender, age and daily activities in children with DCD and at risk of DCD, and typically developing children

L.W. Zanella^**1**^, M.S. de Souza^**1**^, M.J. Kim^**2**^ & N.C. Valentini^**1**^

^1^School of Physical Education, Federal University of Rio Grande do Sul, Porto Alegre, 91760210m, Brazil. nadia.cristina@ufrgs.br; ^2^Institute of Health Science, Korea University, Seoul, Korea.

**Aim:** The purpose of the present study was to investigate the relations among important factors regarding to motor development (MD), BMI age, gender and levels of daily activities of children with DCD and at risk of DCD (r-DCD) and typically developing children (TD) at pre and post intervention.

**Method:** 48 children (5 to 7 year-old) were randomly assigned to intervention (IG: N=24) and control group (CG: N=24). They were assessed by MABC-2, test and checklist: Section A (SA), Section B (SB) and BMI (using the Center of Disease Control’ curves). A motor skill intervention (MSI) was implemented with the mastery motivational climate using the TARGET (Task, Authority, Recognition, Group, Evaluation, Time) structure. MSI for IG was consisted of 32 lessons, 2 lessons per week during 16 weeks and researchers provided 70-minute lesson for each lesson. The CG participated in regular physical education classes.

**Results:** Pearson correlations indicated that: (1) at pre test for children with DCD, MD has been shown significant and positive correlation regarding to age (*r*=.63, *p*=.008) and BMI(*r*=-.78, *p*<.001); (2) at the post test for children with TD, significant and positive correlations were found between MD and gender (r=-.47 p=.003). The results from the linear regression indicated that: (1) for children with DCD at the pre test, a significant model was found [*r*^2^=.71, F_(5,8)_=4.02, p=.040]. It seems that BMI might be the only variable to significantly predict MD (p=.019); (2) non significant models were observed for children at r-DCD (*r*^2^=.624, F_(2,6)_=4.97, p=.053) and TD (*r*^2^=.14, F_(1,23)_=3.77,p=.064); (3) for children TD at the post test, significant model was found (*r*^2^= .36, F_(5,26)_=2.96, p=.030), SB might be interpreted as a significant predictor of MD (p=.034); (4) for children with DCD at the post test, non significant models were found (*r*^2^=.40, F_(3,9)_=1.99, p=.19); and, (5) for children at r-DCD, a model was not observed.

**Discussion:** The present results suggested that MD of the children with DCD was influenced by the age and BMI at the pre test. Age and BMI are frequently reported as risk factors for children with DCD. Interestingly, however, at the post-test those variables were not significantly influenced in MD. MSI probably have positive effect on those relationships. For children with TD, boys participated more vigorously in physical activity than girls and this phenomenon might reflect different levels of participation with respect to gender.

**References**:

Cairney, J., Hay, J. A., Faught, B. E., & Hawes, R. (2005). Developmental coordination disorder and overweight and obesity in children aged 9–14 y. *International Journal of Obesity*, *29*(4), 369-372.

Cairney, J., Kwan, M. Y., Hay, J. A., & Faught, B. E. (2012). Developmental coordination disorder, gender, and body weight: Examining the impact of participation in active play. *Research in Developmental Disabilities*, *33*(5), 1566-1573.

**Keywords:** Developmental coordination disorder; at risk of DCD; BMI; Age, Motor skill intervention.

